# Pathways Activated during Human Asthma Exacerbation as Revealed by Gene Expression Patterns in Blood

**DOI:** 10.1371/journal.pone.0021902

**Published:** 2011-07-14

**Authors:** Unnur S. Bjornsdottir, Stephen T. Holgate, Padmalatha S. Reddy, Andrew A. Hill, Charlotte M. McKee, Cristina I. Csimma, Amy A. Weaver, Holly M. Legault, Clayton G. Small, Renee C. Ramsey, Debra K. Ellis, Conor M. Burke, Philip J. Thompson, Peter H. Howarth, Andrew J. Wardlaw, Phillip G. Bardin, David I. Bernstein, Louis B. Irving, Geoffrey L. Chupp, George W. Bensch, Gregory W. Bensch, Jon E. Stahlman, Monroe Karetzky, James W. Baker, Rachel L. Miller, Brad H. Goodman, Donald G. Raible, Samuel J. Goldman, Douglas K. Miller, John L. Ryan, Andrew J. Dorner, Frederick W. Immermann, Margot O'Toole

**Affiliations:** 1 Department of Allergy/Clinical Immunology, University of Iceland, Reykjavík, Iceland; 2 University of Southampton, Southampton, United Kingdom; 3 Pfizer, Cambridge, Massachusetts, United States of America; 4 Wyeth Research, Cambridge, Massachusetts, United Sates of America; 5 James Connolly Memorial Hospital, Dublin, Ireland; 6 Lung Institute of WA and Centre for Asthma, Allergy & Respiratory Research, University of Western Australia, Crawley, Australia; 7 University of Leicester, Leicester, United Kingdom; 8 Monash University and Medical Centre, Melbourne, Australia; 9 University of Cincinnati College of Medicine, Cincinnati, Ohio, United States of America; 10 Royal Melbourne Hospital, Parkville, Australia; 11 Yale University School of Medicine, New Haven, Connecticut, United States of America; 12 Bensch Clinical Research, Stockton, California, United States of America; 13 Allergy and Asthma Center, Conyers, Georgia, United States of America; 14 Newark Beth Israel Medical Center, Newark, New Jersey, United States of America; 15 Baker Allergy, Asthma and Dermatology, Lake Oswego, Oregon, United States of America; 16 Columbia University Medical Center, New York, New York, United States of America; 17 Coastal Allergy and Asthma, Savannah, Georgia, United States of America; Centro de Investigación Príncipe Felipe, Spain

## Abstract

**Background:**

Asthma exacerbations remain a major unmet clinical need. The difficulty in obtaining airway tissue and bronchoalveolar lavage samples during exacerbations has greatly hampered study of naturally occurring exacerbations. This study was conducted to determine if mRNA profiling of peripheral blood mononuclear cells (PBMCs) could provide information on the systemic molecular pathways involved during asthma exacerbations.

**Methodology/Principal Findings:**

Over the course of one year, gene expression levels during stable asthma, exacerbation, and two weeks after an exacerbation were compared using oligonucleotide arrays. For each of 118 subjects who experienced at least one asthma exacerbation, the gene expression patterns in a sample of peripheral blood mononuclear cells collected during an exacerbation episode were compared to patterns observed in multiple samples from the same subject collected during quiescent asthma. Analysis of covariance identified genes whose levels of expression changed during exacerbations and returned to quiescent levels by two weeks. Heterogeneity among visits in expression profiles was examined using K-means clustering. Three distinct exacerbation-associated gene expression signatures were identified. One signature indicated that, even among patients without symptoms of respiratory infection, genes of innate immunity were activated. Antigen-independent T cell activation mediated by IL15 was also indicated by this signature. A second signature revealed strong evidence of lymphocyte activation through antigen receptors and subsequent downstream events of adaptive immunity. The number of genes identified in the third signature was too few to draw conclusions on the mechanisms driving those exacerbations.

**Conclusions/Significance:**

This study has shown that analysis of PBMCs reveals systemic changes accompanying asthma exacerbation and has laid the foundation for future comparative studies using PBMCs.

## Introduction

While asthma is a chronic inflammatory disorder of the conducting airways causing variable airflow obstruction, sudden deterioration of asthma control in the form of exacerbations, even in the presence of adequate controller therapy, creates a major unmet clinical need. At worst, exacerbations can lead to death, and at best to unscheduled healthcare interventions accounting for most hospital admissions for asthma. Asthma exacerbations are caused by a wide variety of different factors acting singly or together including inadequate controller therapy, exposure to environmental insults (especially allergens, infectious agents, air pollutants, irritant chemicals, and certain drugs) as well as endogenous factors such as changes in sex hormones and psychological stress [1]. The frequency and severity of asthma exacerbations can be reduced by adherence to asthma management guidelines and adequate use of controller therapy. Indeed the most recent guidelines (GINA, BTS and US) advocate control of baseline asthma and prevention of exacerbations as the optimal targets of therapy. While some causes of exacerbating asthma (such as those resulting from inadequate baseline therapy, poor compliance and allergen exposure) can be effectively modified by increasing anti-inflammatory therapy, especially inhaled corticosteroids, others (such as those triggered by virus infection or air pollution episodes) are much less responsive. Indeed, clinical trials of doubling the dose of inhaled corticosteroids at the onset of a naturally occurring exacerbation have shown no beneficial effect [2], [3], although higher doses and oral corticosteroids are more effective [4]. Adequate doses of inhaled corticosteroids alone or in combination with long-acting β_2_-adrenoceptor agonists, leukotriene receptor antagonists and/or anti-IgE monoclonal antibody result in reduced number and severity of exacerbations. These effects of therapy are likely due to a combination of reduced baseline airway inflammation, bronchodilatation and variable suppression of the underlying cellular mechanisms that drive the exacerbation itself [5]. However, even in the case of biologics that target highly selected pathways such as monoclonal antibodies against IgE (omalizumab) and/or IL5, their effectiveness will be limited to exacerbation subtypes that utilize these inflammatory pathways [6], [7].

Although much is now known about the immunological, inflammatory cells and mediators involved in different asthma subtypes, it is surprising that almost nothing is known about the mechanisms involved in exacerbations other than that they are triggered by inadequate controller therapy, respiratory viral infection and allergen exposure. A dominant eosinophil or mixed eosinophil and neutrophil response in blood, sputum and bronchoalveolar lavage and release of a range of inflammatory mediators, cytokines and chemokines during exacerbations strongly supports the existence of heterogeneous mechanisms [8]. Difficulty in obtaining airway tissue and lavage samples during naturally occurring exacerbations has greatly hampered the study of their underlying mechanisms. However, in one bronchial biopsy study of severe asthma exacerbations, there was a similar increase in the number of mucosal eosinophils and neutrophils that was accompanied by increased expression of mRNA for the chemokines CXCL5 (epithelial cell-derived neutrophil-activating peptide-78) and CXCL8 (IL8) and their receptors CXCR1 and CXCR2, but the mechanisms involved are unknown [9].

Based on the paucity of mechanistic information on asthma exacerbations, the aim of the current study was to determine if mRNA profiling of peripheral blood mononuclear cells (PBMCs) could provide new insights into the systemic molecular pathways involved during naturally asthma exacerbations in patients with a range of asthma severity. Some of the results of these studies have been previously reported in the form of an abstract [10].

## Materials and Methods

This was a prospective, multi-center non-interventional study conducted in Australia, Iceland, Ireland, U.K., and USA, and approved by the respective Institutional Review Boards or Ethics Committees. The names of the institutional review boards that approved this study are: Research Ethics Committee, Royal Adelaide Hospital, Adelaide, Australia, The Sir Charles Gardiner Hospital Human Research Ethics Committee, Nedlands, Australia, Sothern Health Human Research Ethics Committee, Monash Medical Center, Clayton, Victoria, Australia, Human research Ethics Committee, The Royal Melbourse Hospital, Parkville, Australia, Western Institutional Review Board, Olympia, Wahington, USA, Quorum Review Inc, Seattle, Washington, USA, Sterling Institutional Review Board, Atlanta, Georgia, USA, Yale University Human Investigation Committee, New Haven, Connecticut, USA, Institutional Review Board, Saint Barnabus Health Care System, Newark, Newjersey, USA, Medical Ethics Committee, Northern Health Board, Dubline, Ireland, Southampton &South West hants Local research Ethics Committee, Southampton, UK, Leicestershir, Northamptonshire and Rutland Health Authority Committee on the Ethics of Clinical Research Investigation, Leicerster, UK, National Bioethics Committee, Reykjavik, Iceland. All subjects gave their written informed consent, were aged ≥18 years and had a confirmed diagnosis of mild persistent, moderate persistent, or severe persistent bronchial asthma according to the 1997 Guidelines for the Diagnosis and Management of Asthma [11] Subjects were stratified by disease severity and had to have demonstrated an improvement in forced expiratory volume in 1 second (FEV_1_) of ≥12% from the baseline in response to an inhaled short-acting β_2_-adrenoceptor agonist within 12 months of screening and/or a provocative concentration of methacholine causing a 20% fall in FEV_1_ (PC_20_) of <8 mg/mL. Exclusion criteria included an active infection, major intercurrent illness, allergen immunotherapy, pregnancy or lactation. At screening, baseline information collected included a detailed medical and asthma history, medication use, physical examination and spirometry. The pattern of asthma over the 12 month observation period is supplied in Text S1.

Subjects attended the clinic every 3 months throughout the course of the 12-month study with asthma assessments performed at each visit. In addition, at the first sign of an exacerbation attack subjects were asked to attend the assessment clinic as soon as feasible, and again within two weeks of recovery from exacerbation. At each visit, venous blood samples were collected. Thus there were three types of blood samples collected: 1) Q*uiet* - during stable disease at approximately 3-month intervals, 2) E*xacerbation* - during a 14 day window during which subjects were experiencing symptoms of exacerbation attack and 3) *Follow-up* - within a 14 day window following cessation of the exacerbation attack. *Exacerbation* samples were collected while the subjects were experiencing one or more of the following symptoms – increases in wheezing, chest tightness, and/or shortness of breath. There was no restriction on medication use in the management of these patients either when stable or during exacerbations.

### Analytical Samples

PBMCs from asthma subjects were isolated from whole blood samples (8 ml×6 tubes) collected into cell purification (CPT) tubes (Becton Dickinson, Franklin Lakes, NJ) according to the manufacturer's recommendations. All samples were shipped at room temperature in a temperature controlled box overnight from the clinical site, cell differential counts taken, PBMCs purified according to CPT manufacturer instructions, (Table S1), and cell pellets stored at −80°C pending RNA purification. RLT lysis buffer (with 0.1% β-mercaptoethanol) was added to frozen pellets, RNA isolated using RNeasy Mini Kit (Qiagen, Valencia, CA) and DNase treated (Qiagen RNase-free DNase Kit). Eluted RNA was quantified using a Spectramax96 well plate UV reader (Molecular Devices, Sunnyvale, CA, USA) monitoring A260/280 OD values. The quality of each RNA sample was assessed by the integrity of the 28S and 18S peaks by capillary electrophoresis alongside an RNA molecular weight ladder on the Agilent 2100 bioanalyzer (Agilent Technologies, Palo Alto, CA, USA). RNA was quantified using Spectramax96 (Molecular Devices, Sunnyvale, CA).

### Determining RNA Expression Level

Labelled targets for oligonucleotide arrays were prepared using 2 µg of total RNA according to the protocol provided by Affymetrix (Santa Clara, CA). Biotinylated cRNA was hybridized to the HG-U133A Affymetrix GeneChip Array® which interrogates 23,283 probe sets. Raw intensity values were processed using Affymetrix MAS 5.0 software, which calculated signal expression levels and present/absent calls for each probe set. More detailed descriptions of sample preparation, mRNA expression measurements using the Affymetrix U133A GeneChip and quality control acceptance criteria for GeneChip data are given in the Table S2. Gene expression data for all arrays run has been submitted to NCBI GEO, accession number 19301. Expression levels of polymorphic HLA DQA1 and HLADQB1, and a Y-chromosome specific transcript, RPS4Y1 were checked for each sample from each donor to ensure that each sample was associated with the correct donor, i.e. erroneous sample switching had not occurred.

### ANCOVA and K-Means Analyses

The multiple samples drawn from a given subject during periods of quiet asthma served as the control comparators for samples drawn from the same patient during exacerbations. This repeated-measures study design provided the power to detect changes associated with exacerbation across the large number of subjects analyzed. There were at least 3 *quiet* samples for 85% of the 118 subjects in the study. The percentage of subjects with 1, 2, 3, 4 or 5 *quiet* samples is shown in Figure S1. Levels and variability of gene expression during *quiet* visits in each subject was calculated using all *quiet* samples from the subject.

For each probe set, mean expression levels during *quiet*, *exacerbation*, and *follow-up* visits were compared using repeated-measures analysis of covariance (ANCOVA), (see Text S1 for more detailed description). Heterogeneity of expression during *quiet* states was factored into the ANCOVA, and only exacerbation-associated differences that fell significantly outside the levels observed during *quiet* periods were examined in further detail. In these analyses, log_2_-transformed signal was the response variable, and asthma severity, sex, age category, race, geographical location, visit type, corticosteroid exposure, leukotriene receptor antagonist use, RNA quality and monocyte to lymphocyte ratio were the explanatory variables. To adjust for multiplicity of testing, false discovery rates (FDRs) were calculated across all probe sets, separately for each term in the ANCOVA model or pair-wise contrast using SAS version 9.1 [12]. Additional information is provided in Text S1.

An initial ANCOVA compared mean log_2_ expression levels during exacerbations with levels during *quiet* visits. All genes with mean differences between *exacerbation* and *quiet* visits that were statistically significant at the 0.05 level (unadjusted p-value <0.05) were identified. Heterogeneity in the expression profiles of this set of genes among samples was evaluated using K-means clustering [13]. Specifically, the input to the K-means analysis was the difference between the log_2_ expression level of each *exacerbation* visit and the mean log_2_ expression level of *quiet* visits for the same subject. The K-means clustering partitioned samples solely on the basis of similarity in gene expression profile, i.e. in the absence of additional sample-related information supplied by the investigator. K-means clustering was executed using the R software package (version 2.1.1; www.r-project.org). To estimate the number of *exacerbation* sub-groups that were present within the dataset, repeated K-means cluster analyses were run, setting K (the number of subgroups) to each possible value between 2 and 8. For each number of subgroups, we assessed the separability and robustness of the resulting clusters. Higher separability and robustness reproducibly revealed distinguishable subgroups of exacerbation responses. Separability and robustness were measured using the silhouette statistic (SW) [14] and a simulation-based robustness index (R) [15]. For the robustness index calculation, Gaussian random noise with zero mean and realistic amplitude (a standard deviation of 0.3) was computationally added to the observed log-ratios to simulate biological replication. For each of 100 realizations of the noisy data, K-means clustering was executed as described above, and the co-clustering of all donor pairs recorded. The resulting co-clustering matrix was then divided by the number of realizations (100) to yield a symmetrical matrix of cluster co-occurrence fractions for every sample-pair in the dataset. Additional details describing the evidentiary support for dividing the *exacerbation* samples into 3 subgroups are provided in Text S1.

Based on the K-means assignments of *exacerbation* visits to three subgroups, an ANCOVA was performed to compare log_2_ mean expression for *exacerbation* visits within each subgroup with mean expression during the *quiet* visits. The *exacerbation* versus *quiet* comparison was calculated separately for each exacerbation-associated gene. Since these ANCOVAs were performed using *exacerbation* visits grouped on the basis of similarities in expression pattern, the resulting FDR adjusted p-values must be regarded as relative and not as unconditional probabilities indicating the significance of association with exacerbation in general. Nevertheless, we report these “relative FDR p-values” because they help identify significant changes between *quiet* and *exacerbation* observed within subgroups, and because the values are useful for assessing the relative strength and rank of each association.

### Principal Components (PCA) Analysis

Principal components analysis (PCA) was used to display the relationships among donor-visits in the 3 K-means clusters. PCA was executed in Spotfire DecisionSite 9.0 (TIBCO, Palo Alto, CA).

### Exacerbation-Associated Probe Sets Selection Criteria

We set the cut-offs for association with *exacerbation* within each subgroup at relative FDR p-value <0.05 and an absolute fold change with *exacerbation* >1.2 fold. This fold change cut-off was lower than conventional thresholds. Studies on selection of appropriate fold change cut-offs in gene expression studies have shown that decisions on cut-offs should take the characteristics of individual dataset into account [16], [17], [18], [19]. The relatively large sample sizes in this study and the other considerations described in the discussion are felt to justify the lower than conventional 1.2-fold-cutoff.

### Pathway analysis

Pathway analysis was performed using Ingenuity Pathways Analysis (IPA) (www.ingenuity.com, Ingenuity Systems, Redwood City, CA). Canonical pathways are shown as depicted by IPA, or as expanded using the literature-based pathway building tools in IPA. A right-tailed Fisher's Exact Test was used to identify over-represented functions or pathways in IPA. The p-values derived through these analyses were based on: 1) number of functions/canonical pathway eligible molecules that participate in that annotation, 2) total number of knowledge base molecules known to be associated with that function, 3) total number of functions/canonical pathways eligible molecules, and 4) total number of genes in the reference set (https://analysis.ingenuity.com/pa/info/help/help.htm#ipa_help.htm).

### TaqMan® PCR

We have previously performed extensive analysis comparing expression level data obtained using the Affymetrix U133A GeneChip and TaqMan® PCR and shown highly concordant results. One of these platform concordance studies has been reported previously [20]. The results of another study, using data from some of the *quiet* asthma samples reported in this study, are described in the Figure S2, and Text S1. The overall Pearson correlation coefficient (for measurements of expression differences by the two platforms) was 0.86. Due to this strong concordance, we did not perform a GeneChip and TaqMan® PCR platform concordance analysis for the results reported here. However we did use TaqMan® PCR to measure levels of three specific genes, IFNα1, IFNβ1, and IFNγ because these genes were expressed at levels too low to be detected by the GeneChip, but were functionally related to many genes identified from our GeneChip data. We also measured IL13 by TaqMan® PCR TaqMan® because of the association between the IL13 pathway and asthma. Expression levels of these genes and ZNF592 (used for normalization) were measured using primers, probes and instructions from Applied Biosystems (Table S3 and Text S1). ZNF592 was selected as a normalizer gene based on a survey of oligonucleotide array expression data for 44,928 transcripts across a compendium of 9,270 hybridizations, including multiple studies involving different types of cells and tissues. In this broad survey, ZNF592 had substantially less variability than other commonly utilized endogenous controls. Specifically, ZNF592 had a coefficient of variation which was at the 0.01-th percentile of variation among all surveyed transcripts.

## Results

### Study Population

**Figure 1:**
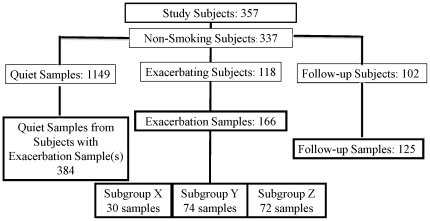
Description of Study Samples. Of 357 subjects enrolled in the study, the 337 non-smoking subjects were selected for analyses of gene expression patterns associated with exacerbation attack. A total of 118 subjects experienced at least one exacerbation attack, and 166 samples were evaluated from these subjects. Analyses were performed comparing expression levels of each probe set in each *exacerbation* sample to the average of *quiet* visits from the same subject.

A total of 357 subjects were enrolled and are described in Table 1 and Table S4. The mean FEV1 for each disease strata (mild, moderate and severe) are shown in Table 2. Detailed information on the enrolled subjects is reported in Text S1 with the following parameter reported in tabulated form: assessment of asthma control (Table S5), healthcare resource use (Table S6), atopy status at screening (Table S7), body mass index and reflus disease (Table S8), history of reflux disease (Table S9), change in asthma severity by visit (Table S10), use of concomitant anti-asthmatic medication (Table S11), use of anti-asthmatic medication by country (Table S12), healthcare resource use during course of study (Table S13), precipitating and aggravating factors by visit (Table S14), adverse events (Table S15), most common respiratory adverse events (Table S16), and mean FEV1 (% predicted) at scheduled non-exacerbation visits (Table S17). To avoid potential confounding effects of smoking, subjects who smoked (n = 20) were excluded from the analyses reported here. Of 337 non-smoking subjects (64.4% female, 87.1% white) enrolled, at least one evaluable *exacerbation* sample was collected from each of 118 subjects, and there were 37 subjects from whom more than one *exacerbation* sample was collected. The total number of exacerbation samples analyzed was 166. From the 118 subjects from whom at least one exacerbation sample was collected, a total of 394 *quiet* samples and 125 *follow-up* samples were also collected (Figure 1 and Figure S1). The vast majority of *exacerbation* samples were from severe (55%) and moderate (41%) asthmatics. The interval between exacerbation onset and collection of the *exacerbation* sample was unavoidably variable. Subjects were requested to go to their doctor's office within 3 days of the onset of the attack, with 72% of samples being collected within this window. For the 166 *exacerbation* samples reported in this study, 25% were collected on the day of onset and 16%, 17%, 14%, 24% and 4% collected on days 1,2, 3, 4−9, and 10−14 post-exacerbation onset respectively.

**Table 1:** Demographic and Baseline Characteristics by Asthma Severity.

Characteristic	Mild	Moderate	Severe	Total
	(n = 36)	(n = 149)	(n = 172)	(N = 357)
Mean age (yr)	41.14	43.4	47.37	45.08
S.D.	12.85	15.18	14.71	14.88
Female, n (%)	26 (72.2)	98 (65.8)	106 (61.6)	230 (64.4)
Male, n (%)	10 (27.8)	51 (34.2)	66 (38.4)	127 (35.6)
Asian, n (%)	0 (0)	4 (2.7)	6 (3.5)	10 (2.8)
Black, n (%)	1 (2.8)	11 (7.4)	24 (14.0)	36 (10.1)
White, n (%)	35 (97.2)	134 (89.9)	142 (82.6)	311 (87.1)
Hispanic, n (%)	0 (0)	3 (2.0)	10 (5.8)	13 (3.6)
Non-Hispanic, n (%)	36 (100)	146 (98.0)	162 (94.2)	344 (96.4)
Mean Weight (Kg)	78.03	82.01	80.87	81.06
S.D.	13.29	18.83	19.88	18.86
Mean Height (cm)	167.37	169.54	166.95	168.07
S.D.	10.39	10.08	9.47	9.88

Subjects were followed for 12 months according to site standard of care. Twenty-seven subjects (7.6%) did not complete the study. The most common reason overall for early withdrawal from the study was failure to return. The most common reason for exclusion was FEV1 reversibility. The reasons for patient exclusion tended to be site-specific rather than country-specific.

**Table 2:** Mean FEV_1_ (% Predicted) at Scheduled Non-Exacerbation Visits.

Exacerbation Status		Asthma Severity		
		Mild	Moderate	Severe
Never	n	81	295	300
	**Mean**	**95.01**	**85.51**	**74.85**
	*P*-value^a^	<0.0001		
At Least 1	n	33	181	244
	**Mean**	**90.27**	**86.33**	**74.15**
	*P*-value^a^	<0.0001		
	*P*-value^b^	0.0479	0.5675	0.6851
All Subjects	n	114	476	544
	**Mean**	**93.64**	**85.83**	**74.54**
	*P*-value^a^	<0.0001		

a *P*-value indicates test for differences among asthma severity groups

b *P*-value indicates test for difference between exacerbation status groups (never had an exacerbation versus had at least 1 exacerbation) within an asthma severity category.

Abbreviations: FEV_1_ = forced expiratory volume in 1 second.

### Partitioning Exacerbation Samples into Three Subgroups

Gene expression levels in each subject in multiple *quiet* samples were compared to levels in the same patient during individual *exacerbations* by ANCOVA performed to determine the association of each probe set with *exacerbation*. There were 1079 probe sets with an unadjusted p-value association with *exacerbation* ranging from 5.33×10^−10^ to 5×10^−2^ (0.05). Upon adjustment for multiplicity of testing, however, the association with *exacerbation* was at an unacceptably low confidence level for the majority of probe sets. To gain an overview of the large dataset, we generated a heat map of the difference between the log_2_ expression level during an *exacerbation* visit and the mean log_2_ expression level during *quiet* visits for the same subject for each of these 1079 probe sets for each of the 166 *exacerbation* samples. As we had expected, this analysis revealed significant heterogeneity of *exacerbation*-associated gene expression patterns among the samples.

**Figure 2:**
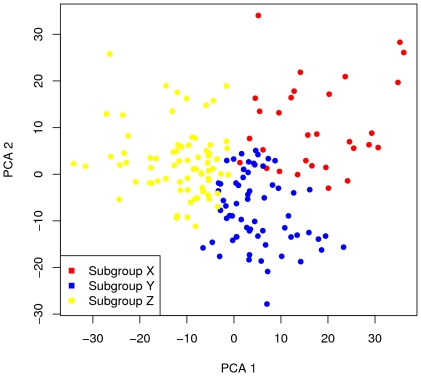
Principal Component Analysis Showing Separation of Subgroups X, Y and Z. PCA was performed on the log-ratios of exacerbation to quiet expression levels for 1,572 probesets measured in 166 donor-visits. The cumulative percent of variation explained by principal components 1, 2, and 3 was 23%, 37%, and 43% respectively; components 1 and 2 are shown in the figure.

K-means clustering was performed to group the samples on the basis of similarities in *exacerbation*-related differences. Since the K-means algorithm partitions samples into the number of subgroups stipulated by the investigator, procedures were performed to determine the number of robust subgroups within the dataset. We assessed the robustness of K-means clusters using both the silhouette statistic (SW)[14] and a simulation-based robustness index (R), similar to the approach of McShane et al.[15]. Figure S3 shows silhouette statistics for K = 2, 3, 4, and 8 clusters. There is an “elbow” in the slope of the SW curve at K = 3, indicating that further increases in K have diminishing benefits in distinguishing distinct groups (Text S1). Figure S4 shows a clear and robust separation into three subgroups (K = 3 clusters, SW = 0.08, R = 0.88). With more than 3 subgroups, the SW and R measures of subgroup robustness declined, indicating no more than three well defined subgroups of samples. Combining these observations with the imperative to use the simplest model that is consistent with the data, we selected K = 3 (text S1). *Exacerbation* samples were therefore partitioned by the K-means algorithm into three subgroups designated X, Y and Z. Each *exacerbation* was assigned by the algorithm to one of these three subgroups with the subgroups comprising 18%, 38% and 43% of *exacerbation* samples respectively. ANCOVA was performed and 1572 probe sets that met the criteria for significant exacerbation association within any subgroup were identified (FDR<0.05 and |fold change| >1.2 in at least one subgroup). Separation of the subgroups using principal component analysis is shown in Figure 2, and a heat map representation ordered by bispectral clustering is shown in Figure S5.

**Figure 3:**
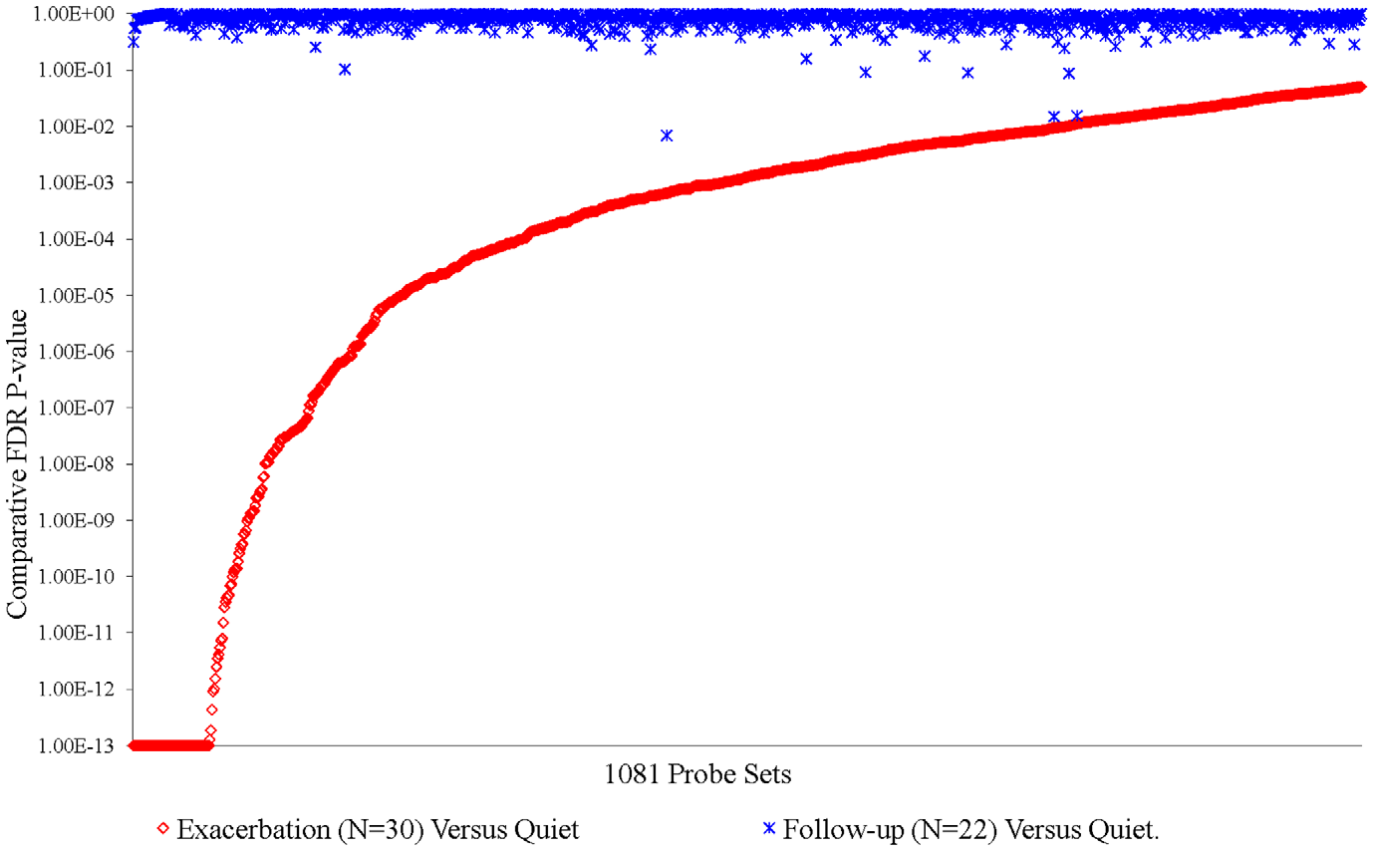
Association of Subgroup X Probe Sets with Exacerbation. Relative FDR p-value for 1081 probe sets meeting selection criteria in ANCOVA on 30 Subgroup X *exacerbation* samples compared to the average of their corresponding *quiet* samples. To clarify the visual representations, probe sets have been ordered by descending relative FDR p-value in the comparison of *quiet* versus *exacerbation* samples. The metrics associated with each of the 1081 probe sets are given in the Table S18. See Figure S6 for results obtained from ANCOVA comparing *quiet* and *exacerbation* using only the 22 *exacerbation* samples with corresponding *follow-up* sample.

**Figure 4:**
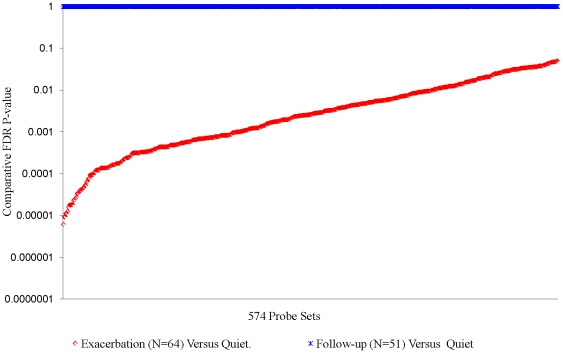
Association of Subgroup Y Probe Sets with Exacerbation. Relative FDR for 574 probe sets meeting selection criteria in ANCOVA of 64 Subgroup Y *exacerbation* samples and the average of their corresponding *quiet* samples. See Table S18 for metrics for each individual probe set, and Figure S6 for results obtained comparing *quiet* and *exacerbation* using only the 55 *exacerbation* samples with corresponding *follow-up* sample.

**Figure 5:**
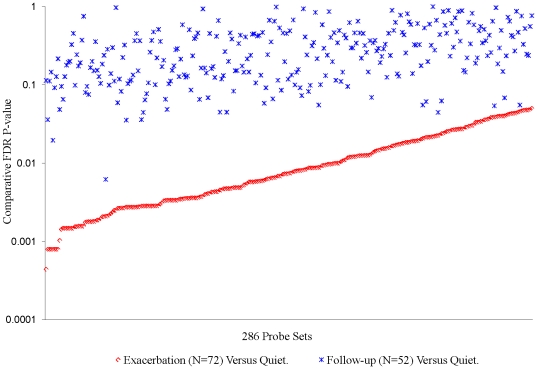
Association of Subgroup Z Probe Sets with Exacerbation. Relative FDR for 286 probe sets meeting selection criteria in ANCOVA of 72 Subgroup Z *exacerbation* samples and the average of their corresponding *quiet* samples. See Table S18 for metrics on individual probe sets and Figure S6 for results comparing *quiet* and *exacerbation* using only the 52 *exacerbation* samples with corresponding *follow-up* sample.

For Subgroups X, Y and Z, there were 1081, 574 and 286 probe sets respectively that met the criteria for significant association with *exacerbation*. The FDR adjusted p-values for association each of these subgroups are summarized in Figures 3, 4, 5, and Figure S6 of Text S1. Table S18 gives the identity of each gene, the significance of the association with exacerbation, and the log_2_ fold change with exacerbation. Separate ANCOVAs comparing mean expression levels in *quiet* and *follow-up* samples showed that, with a very small number (4%) of exceptions in Subgroup Z, *exacerbation*–associated probe sets were not different (relative FDR p-value>0.05) from *quiet* levels two weeks after cessation of an *exacerbation* (Figures 3,4, and 5, and Figures S6). These results show that: a) significant differences were detected in gene expression in the blood of asthmatics during *quiet* and *exacerbation* periods of disease, b) no significant differences were identified when samples from individuals who were not experiencing exacerbation (follow-up samples) were compared to the *quiet* sample dataset using identical procedures used in the analysis with *exacerbation* samples. This latter point establishes that the identification of differences in the *quiet* versus *exacerbation* comparison cannot be attributed to unknown artifact(s) introduced by conducting the analyses as described.

### Detection of Exacerbation-Associated Genes and Biological Pathways

**Figure 6:**
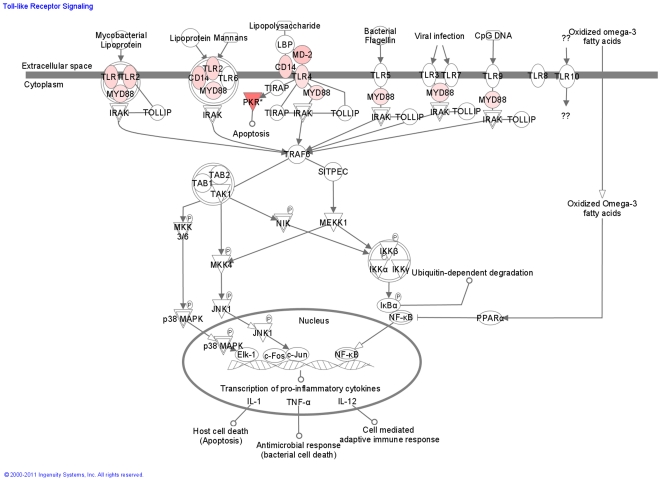
Activation of the TLR Pathway in Subgroup X. Genes that are significantly upregulated in exacerbation are shown in red.

**Figure 7:**
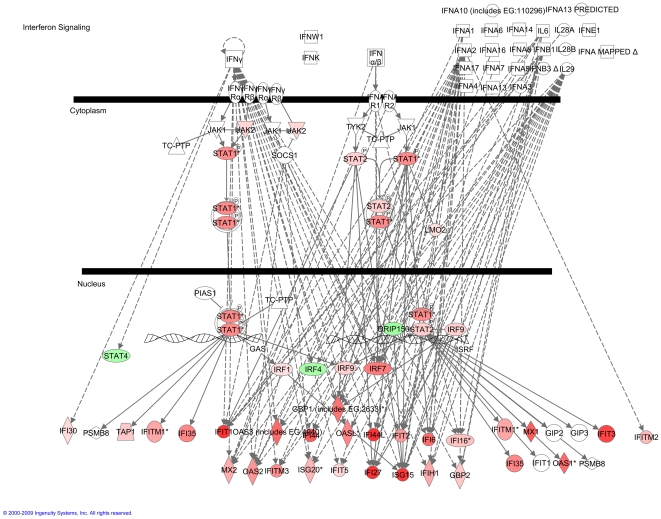
Activation of the Interferon Pathway in Subgroup X. Type I and Type II interferon-induced genes including IRFs that are involved in the regulation of interferon response, and the JAK-STAT signaling components of the IFN signaling pathway that are upregulated in Subgroup X exacerbations are shown in red.

**Figure 8:**
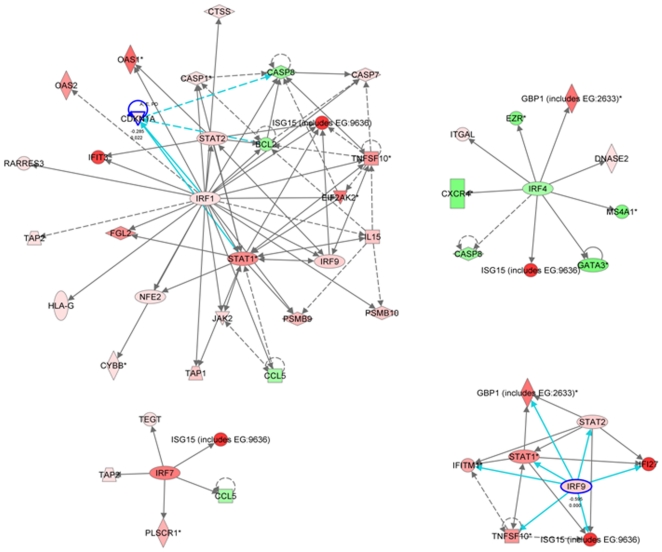
Activation of the Interferon Response Factors in Subgroup X. Several genes upregulated in Subgroup X exacerbations are controlled by IRFs and are shown in red. In addition to IRF1, IRF7 and IRF9 are up-regulated in exacerbation along with the target genes that these IRFs regulate. IRF4 is down-regulated (as shown in green) and this change is consistent with the changes observed in IRF4-target genes. IRF1 appears to be the major driver of gene expression changes in Subgroup X. IRF1 is also involved in the regulation of IL15 expression.

Among Subgroup X genes, components of the toll-like receptor (TLR) and interferon response signaling pathways were significantly over-represented (Figure 6). P-values determined by Ingenuity Pathway Analysis for significance of over-representation of interferon and TLR pathways are = 4.23×10^−8^ and 2.66×10^−2^ respectively. Interferon-inducible genes that were over-expressed included the interferon regulatory factors (IRFs) −1, −7 and −9 (Figure 7). IRF-4 was down-regulated as were its target genes (Figure 8). Pathway analysis indicated a role for either IFNα_1_, IFNβ_1_, and IFNγ in *exacerbation*, but these three genes were below detectable levels using the HG-U133A Affymetrix GeneChip Array®^.^ As a result, expression levels of these three genes were measured TaqMan® PCR. In Subgroup X, significant elevation of mRNA for type I interferons IFNα_1_ (p = 4.7×10^−3^) and IFNβ_1_ (p = 3.1×10^−3^) was observed, while the association with the type II interferon (IFNγ)was not significant, indicating that the activation of IFN associated genes was being driven by type I interferon.

**Figure 9:**
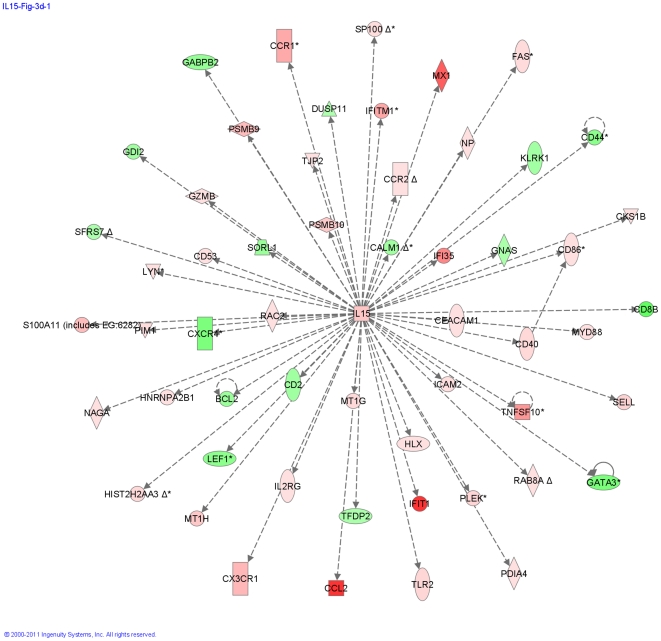
Modulation of the IL15 Pathway in Subgroup X. Many IL15-induced genes are significantly modulated in Subgroup X, and this network is also identified by Ingenuity Pathway analysis as a significant pathway among Subgroup X genes. Red indicates genes up-regulated in exacerbation and green indicate genes down-regulated in exacerbation. See Table S5 for a complete list of IL15 pathway genes associated with exacerbation.

One of the genes regulated by IRF7 is IL15, and a network centered on IL15 was found to be highly significant in Subgroup X. Ingenuity Pathway Analysis determined the significance of over-representation of IL15-regulated genes at p = 4×10^−13^, indicating a role for this cytokine in the expression of several exacerbation-related genes (Figure 9 and Table S19). Consistent with the TCR-independent activation of T cell by IL15 [21], there was down-regulation of the TCR activation pathway in Subgroup X (Text S1, Table S18). These results support the dominant involvement of innate immune pathways in Subgroup X.

**Figure 10:**
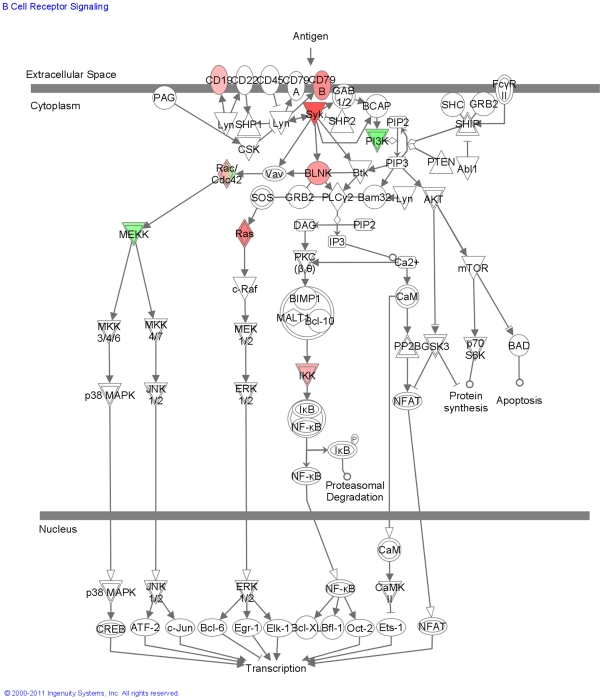
Modulation of the B Cell Antigen Receptor Pathway in Subgroup Y. Shown in red are B-cell receptor signaling pathway components that are up-regulated in Subgroup Y exacerbations.

**Figure 11:**
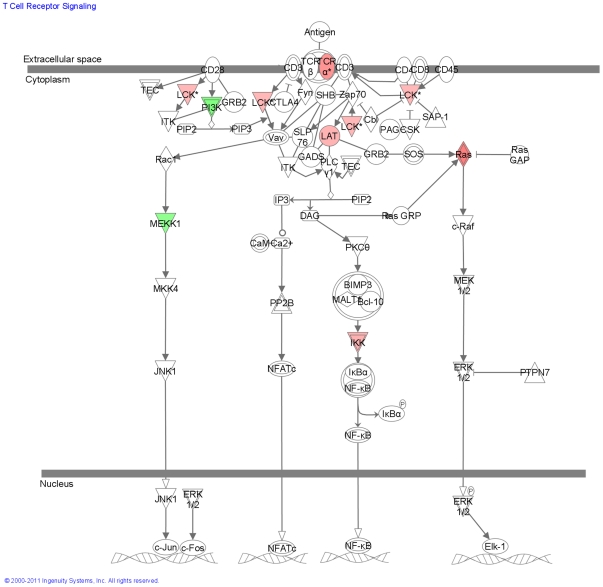
Modulation of the T Cell Antigen Receptor in Subgroup Y. Shown in red are T-cell receptor signaling pathway components that are up-regulated in Subgroup Y exacerbations.

In contrast to the dominant signatures of innate immunity detected in Subgroup X, the gene expression patterns in Subgroup Y were indicative of a dominant role of antigen driven pathways of adaptive immunity. Genes associated with the activation of B cells through the B cell antigen receptor were more significantly upregulated in Subgroup Y than in the other subgroups (Figure 10). Ingenuity Pathway Analysis determined the significance of over-representation of B cells antigen receptor pathway in Subgroup Y at p = 3.54×10^−3^. While genes of the T cell receptor-dependent pathway were down-regulated in Subgroup X, the same genes were significantly up-regulated in Subgroup Y (Figure 11). Ingenuity Pathway Analysis determined the significance of over-representation of T cells receptor pathway genes in Subgroup Y at p = 1.5×10^−2^. Genes of the IL4 pathway, a pathway with well established links to asthma [22], [23], were also over-represented in Subgroup Y (p = 6.2×10^−3^). Because IL13 was not detectable by GeneChip and is known to be an important mediator of lung inflammation and IgE production [24], [25], [26], [27], IL13 expression levels were measured in a small subset of samples by TaqMan® PCR. We did not detect a significant difference between *quiet* and *exacerbation* samples within any of the subgroups.

Compared to the other subgroups, the genes associated with *exacerbation* within subgroup Z were far fewer and of less statistical significance (Figures 5). There were 674 probe sets in Subgroup X and 110 in Subgroup Y that exhibited more significant associations with *exacerbation* than the most significant associations seen in Subgroup Z. Since Subgroup Z is the largest of the 3 subgroups in terms of number of samples, the comparatively weak signature cannot be attributed to lack of statistical power. Of the 286 probe sets in Subgroup Z with significantly altered expression relative to *quiet* visits, only 26% (75) of the 286 probe sets were unique to Subgroup Z, and this relatively short list of genes was not sufficient for an informative pathway analysis. In the majority of cases where Subgroup Z genes overlapped with those of Subgroup X and/or Y, the association with *exacerbation* in Subgroup Z was much less significant, again indicating that the Subgroup Z expression pattern is weak by comparison to the other subgroups.

### Search for Subgroup Assignment and Clinical Parameters Associations

We examined whether multiple *exacerbations* from the same subject were assigned to the same subgroup, and for 19 subjects this was not the case. Therefore, subgroup assignment was not solely attributable to an invariant parameter associated with the subject. The time between exacerbation onset and collection of the *exacerbation* sample varied from 0 to 14 days, but the subgroups did not differ with respect to this parameter (Table 3, Figure S7 and Text S1). Due to the non-normal distribution of days from start, a non-parametric version of a standard ANOVA was run, and no evidence of differences among clusters in mean days from start was observed (p-value = 0.49 in test for differences of means among clusters, calculated from a one-way ANOVA run on the ranks.) Multiple analyses were performed to identify any associations between subgroup assignment and respiratory function and no associations were identified. For these analyses respiratory function was assessed by FEV1 predicted (Table S20), FEV1 change from baseline (Table S21), FVC predicted (Table S22), FVC change from baseline (Table S23), FEF 25–75% predicted (Table S24), FEF 25–75% change from baseline (Table S25), PEF predicted (Table S26), PEF change from baseline (Table S27) or relevant respiratory infection (Table S28). There was also no association between cluster assignment and disease severity (Table S29). Cluster assignment also did not show an association with use of medications such as systemic corticosteroids (Table S30), inhaled corticosteroids (Table S31), intranasal corticosteroids (Table S32) leukotriene antagonists (Table S33), any GI medication use (Table S34), any PPI medication use (table S35), or any histamine H2 antagonist (Table S36). Analysis also failed to identify an association between subgroup assignment and sex (Table S37), race (Table S38), location of sample processing (Table S39), country (Table S40), atopy status (Table S41), fasting status (Table S42), IgE levels (Table S43) or history of acid reflux (Table S44).

**Table 3:** Subgroup Assignment and Days Between Exacerbation Onset and Collection of *Exacerbation* Sample.

	Subgroup		
	X	Y	Z
Number of *exacerbation* visit samples	30	64	72
Median number of days between onset and sample collection	2	2	2
Minimum number days	0	0	0
Maximum number of days	9	14	12
Quartile 1	0	0	1
Quartile 3	2.8	4.0	3.3

Of the 27 analyses run in an effort to identity associations between subgroup assignment and other parameters, two analyses identified significant associations. Mean BMI was statistically significantly lower (p = 0.006) in Subgroup X than Subgroup Y, and statistically suggestively lower (p = 0.0501) in Sub-group Z than in Subgroup Y (Table S45). This finding indicates that those with the highest BMI tended to be preferentially assigned to Subgroup Y. As discussed below, this finding is notable in light of the report from Haldar et al. [28] that obese non-eosinophilic asthmatics constitute a cluster of asthmatics that differ from other types of asthmatics with respect to clinical response to treatment. The other parameter that showed a significant association with subgroup assignment was the time between quiet visit and subsequent exacerbation visit (Table 4 and Table S46). There was a significantly shorter time between a quiet visit and the subsequent exacerbation visits for samples in Subgroups X and Y (median days 40.5 and 40 respectively) than for samples in Subgroup Z (median days 69).

**Table 4:** Association Between Subgroup Assignment and Days Since Exacerbation Visit and Previous Quiet Visit.

	Subgroup based on 1079 probeset clustering		
Statistic	Subgroup X	Subgroup Y	Subgroup Z
N	30	64	71
Mean	48.4	62.7	79.6
Median	40.5	40	69
S.D.	45.3	55.3	64.0
CV	93.6	88.3	80.5
5^th^ percentile	9	7	11
95^th^ percentile	91	181	211
Missing values	0	0	1

Differences between nodes in mean number of days since quiet visit. *Exacerbation* visits occur sooner after a *quiet* visit in some nodes than in others. Statistically significant difference between Subgroups 1 and 3 (p = 0.014), difference between Subgroups 2 and 3 is statistically suggestive (p = 0.091. p-value from test for differences of means among Subgroups = 0.03.

## Discussion

The elucidation of mechanisms that drive naturally occurring human asthma exacerbations presents a considerable translational medicine challenge in this area of unmet medical need. We have conducted a multinational study and searched for changes in PBMC gene expression coincident with asthma exacerbation. The approach we have taken was intended to test whether advances in understanding could be made in the absence of pre-conceived bias by using the broad net of expression profiling and bioinformatics approaches. The strengths of our study are its large size, longitudinal design, recruitment of patients from multiple sites in the Northern and Southern Hemisphere and collection of samples from individual subjects during multiple *quiet*, naturally occurring *exacerbation* and *follow-up* periods in real-life settings.

From the start, it was recognized that, even if exacerbation associated gene expression patterns existed in the blood, they were likely to be heterogeneous. This prediction was based on the known complexity and heterogeneity of asthma and asthma exacerbations, the multiple triggers associated with natural exacerbations, and the knowledge that many sources of variability could not be controlled in a study of naturally occurring exacerbations. Adding to these challenges was the realization that, prior to study initiation, we had little information on what to base predictions of the strength signal(s) or the degree of sample heterogeneity. Therefore, the study design and data analysis plans contained elements aimed at maximizing the chances of detecting changes in gene expression that could, with a high level of confidence, be attributed to exacerbation. First, the number of subjects enrolled was as large as we could accommodate in a study which depended on high content oligonucleotide arrays. From the total of 337 enrolled subjects, 166 *exacerbation* samples from 118 subjects who had at least one exacerbation attack were collected. Secondly, the average number of *quiet* samples collected over the course of a year from each of these exacerbating subjects was 3.3, enabling determination of the variability in level of expression of each gene in each subject during quiescent asthma over the course of approximately one year. These multiple *quiet* samples for each subject served as the control comparators for a sample or samples drawn from the same subject during *exacerbation*s. ANCOVA probed for differences in each *exacerbation* sample as compared to the levels observed in *quiet* samples from the same subject. Covariate analysis adjusted for effects associated with many covariates including steroid use, age, sex, and cell differentials. Thirdly, data-driven clustering algorithms that operate without investigator bias were employed to characterize the heterogeneity of exacerbation-related expression patterns, and determine the number of well defined subgroups within the dataset.

The study design enabled the identification of changes in PBMC gene expression coincident with asthma exacerbation. Our first pass analysis on the complete sample set indicated an exacerbation-associated gene expression pattern encompassing a large number of genes most of which, on an individual basis, were associated with exacerbation at an unacceptably low confidence level. We examined the expression pattern of all samples using the 1079 probe sets associated with exacerbation with a low level of confidence (unadjusted p-value<0.05), and used that data to examine the heterogeneity among samples. The heterogeneity revealed by this analysis indicated that the relative statistical weakness of the associations identified by ANCOVA was explained by differences among samples with respect to exacerbation-associated fold change in gene expression. Clearly, the heterogeneity within the group of *exacerbation* samples as a whole masked much of the information that could be garnered by analyzing the less heterogeneous subgroups of samples.

It must be recognized that it would not be possible to avoid many of the sources of variability inherent in a yearlong observational study of naturally occurring exacerbation in subjects living their normal lives. For example the interval between exacerbation onset and sample collection varied from 0 to 14 days. A number of other variables would be predicted to affect results including, but not limited to, severity of underlying disease, severity of exacerbation attack, patient disease phenotype, timing and type of medication, type of trigger, and level of exposure to trigger. The interactions between these variables would also be predicted to affect results. Each *exacerbation* sample should therefore be viewed as a single time point “snap-shot” of a disease state that is influenced by many covariates and waxes and wanes due to the natural course of exacerbation resolution and the effects of therapy. It was our hypothesis that, if *exacerbation*-associated expression changes occurred in blood, heterogeneity of expression patterns would be observed. The size of the study reflected this hypothesis, and we took the risk that the study was sufficiently large to allow an examination of heterogeneity.

We proceeded with K-means clustering to partition samples on the basis of similarity of *exacerbation*-related gene expression pattern. Because the number of groups into which K-means partitions samples is specified by investigator, we performed a series of analyses to ascertain separability and robustness, which measure how distinct the subgroups are from each other. Based on the outcome of these analyses, we concluded that the samples should be subdivided into three groups. K-means analysis was then used to assign each of 166 *exacerbation* samples to 1 of 3 subgroups. The direct consequence of grouping samples on the basis of similarity in *exacerbation* related changes was a reduction in variability within each subgroup. As a direct consequence of the reduced variability, ANCOVA run on samples categorized by subgroup identified many more genes that were associated with *exacerbation* within an acceptable confidence limit (relative FDR<0.05). The *exacerbation* samples in subgroups X and Y showed many robust differences with the *quiet* samples. The *exacerbation* samples in Subgroup Z (44% of *exacerbation* samples) showed much less profound differences with *quiet*. These results suggested that the relatively weak *exacerbation*-associated expression pattern in Subgroup Z greatly diminished the ability to detect *exacerbation*-associated genes when all the samples were analyzed as a whole.

Choosing gene selection criteria in studies using oligonucleotide arrays poses significant challenges that cannot be met simply by using cut-offs adopted by convention [16], [17], [18], [19]. While the 1.2 fold change cut-off chosen was low in comparison to convention, the large size of study reported here provided better statistical power than usually available in GeneChip-based translational medicine studies of mixed cell populations. Our choice of the 1.2 fold change cut-off was based on the following three considerations. First, we examined the relationship between fold change and relative FDR p-value in this particular dataset and observed that a high proportion of probe sets with fold change between 1.2 and 1.3 also had a relative FDR p-value <0.05. This was not the case for probe sets with fold change between 1.1 and 1.2. For example, in the analyses of Subgroup X, 70% of probe set with fold change between 1.2 and 1.3 were significantly associated with *exacerbation* (relative FDR p-value <0.05). Exclusion of these probe sets from the analysis of biological pathway involved would have diminished the information on the representation of various pathways within the dataset. Our goal was to address the biology and probe the pathways that are dysregulated in exacerbation, and slight but significant fold changes of several genes within a given pathway provided cumulative evidence implicating the pathway. Consistent with this analytical approach we note that any impact of false positive identifications would be mitigated by a lack of cumulative evidence provided by functionally related genes. A second consideration in setting the fold change cut-off at 1.2 was that statistically significant but small changes in magnitude were of biological interest. The profiles were generated using the mixed population of cells in the periphery, and therefore a large magnitude change in one, perhaps minority, cell population would be expected to have a diluted impact on the average fold change observed in the population as a whole. The third source of support for the 1.2 fold cut-off decision was obtained from a GeneChip study comparing *quiet* asthma samples to healthy volunteer samples. We selected 24 pairs of samples and used a custom low density TaqMan® array to measure the fold change of 192 genes by TaqMan® PCR. The fold change between samples by GeneChip was below 1.2 for a significant number of gene/sample pair combinations. We compared the log_2_ signal differences obtained by GeneChip to the delta CT differences obtained by TaqMan®. Results between platforms were concordant in 87% of the comparisons performed, and lack of concordance was associated with low expression level, and not with low fold change (O'Toole, Burczynski et al. unpublished data). We recognize that by imposing the fold change filter in addition to the relative FDR p-value criteria some true positives have most likely been excluded (especially among the few probe sets with fold change <1.2 and very low relative FDR p-values) and some false positives included (especially among probe sets with low fold change and relative FDR p-value close to the 0.05 cut-off), but based on the three considerations described here, the 1.2 fold cut-off was the most appropriate cut-off for this study.

The size of the study and distribution of the expression values were sufficient to allow definition of three robust subgroups of *exacerbation*-associated gene expression profiles. Key validation that analytical methods and selection criteria used had identified genes associated with exacerbation in each of the individual subgroups was obtained when the intra-subgroup ANCOVA comparison of *quiet* and follow-up samples did not identify significant differences. Thus, ANCOVA did not merely identify highly variable genes that, by random chance, differed significantly between the *exacerbation* set of samples and *quiet* set of samples.

Examination of the known biological links between *exacerbation*-associated genes led to our conclusions that systemic immune pathways are extensively activated during asthma exacerbations. Innate and antigen-independent immune pathways were predominantly activated in subgroup X, with toll-like receptors TLR1, TLR2 and TLR4 being significantly elevated (Figure 6). Cell activation through TLRs is a well established driver of type I interferon responses. Because interferon genes were not themselves detectable by GeneChip, TaqMan® PCR assays were done to confirm significant elevation of Type I interferon genes. The importance of this innate immune response was reinforced by the finding of extensive interferon pathway activation associated with Subgroup X and extensive activation of many interferon inducible genes such as OAS1, OAS3, MX1, and IFITM3 as well as the interferon regulatory factors IRF 1,7 and 9 (Figure 7). Taken together these data indicate that in Subgroup X, TLR activation leads to induction of a systemic type I interferon response.

It is well established that infection triggers activation of the innate immunity pathways, and that type I interferon response is closely linked to viral infection via activation of TLR-3 and -7/-8 by ss- and ds-RNA respectively [29], [30]. An important question, but one beyond the scope of this study, is how do the peripheral blood expression profile changes seen in asthmatics during respiratory infection differ from the changes seen in non-asthmatics during respiratory infection? Since respiratory infections are a common trigger of asthma exacerbations [31], a study that identifies differences between asthmatics and non-asthmatics in pathways activated during respiratory infections could advance understanding of the disease. Among the utilities of this study is that it has laid a foundation showing the feasibility and likely fruitfulness of such a study.

It has been reported in normal subjects that during viral upper respiratory tract infection in the absence of lower respiratory tract involvement, there is no systemic interferon response [32]. This finding contrasts with our results on robust systemic interferon activation signature in the blood of exacerbating asthmatics - both infected and without symptomatic evidence of infection. One possible explanation for these differing results is that homing processes may be at least somewhat abnormal in asthmatics. Inappropriate homing could promote virus proliferation in the lower airways, cytotoxic injury and entry into the circulation as has recently been shown in childhood asthma exacerbation [33]. A recent study using Illumina Human Bead Chip arrays applied to PBMCs compared expression during exacerbations in dust mite sensitive asthmatic children to expression in stable asthma and normal controls. That study found that many of the asthma exacerbation related genes were involved in defense responses and responses to external stimuli, but these associations disappeared after excluding infection related genes [34]. However, this study was much smaller (N = 12 exacerbation samples) than the study reported here (n = 166 *exacerbation* samples).

An unexpected finding was that a large proportion of the *exacerbation* samples with the robust signature of innate immune activation were from patients for whom symptoms of respiratory infection were not reported by the patient nor noted by the physician. Among possible explanations for this are: a) pathogen load too low to result in commonly recognized symptoms of infection but sufficient to trigger innate immunity in asthmatics, or b) triggering through TLR ligands such as reactivated bacteria [35], resident viruses [36], biologically active allergens such as Der P2 [37] or ambient air pollutants as encountered in an air pollution episode [38], [39]. Indeed many endogenous molecules that are increased with inflammation are TLR ligands and agonists [40], [41], [42], [43], [44], and in mice the immunostimulatory activity of lung surfactant protein A is TLR4-dependent [45]. Therefore the molecules that activate innate immunity may be of either pathogen or host origin, and the strong signature of innate immunity implicates innate immunity in *exacerbation* even in the absence of symptoms of respiratory infection. This interpretation fits with the accumulating evidence that innate immunity plays an important role in asthma [46], [47], [48], [49], [50], [51] and with the findings of association between asthma and single nucleotide polymorphisms in TLRs [52], [53], [54], [55], [56] and associated molecules [57], [58], [59], [60], [61].

Another prominent characteristic of Subgroup X exacerbations was a highly significant representation of IL15-pathway genes. IL15 production is known to be strongly induced by interferons [62], transcriptionally activated by IRF-1 [63], supports a non-TCR–mediated T-cell response [21] and results in activation of CD8 T cells [64]. IL15 has also been linked to asthma and allergy by DNA polymorphism association [65], [66]. These reports are consistent with our findings on the down-regulation of the TCR pathway in Subgroup X. This study implicates IL15 as a bridge between innate and adaptive immune responses in asthma exacerbation.

Subgroup Y genes that increased during exacerbation included those involved in B-cell activation pathway through B-cell antigen receptor (BCR) and the IL4 signalling pathway involved in inducing and maintaining pro-allergic Th-2 cell and IgE responses [22], [23]. These responses are linked to a strong adaptive allergen-driven immune response, and distinguish Subgroup Y *exacerbation*s from the innate immunity pathways that dominated the gene signature of Subgroup X. While the signatures of innate immunity did not predominate in Subgroup Y as they did in Subgroup X, NK signalling was significant in both subgroups, with more activation detected in Subgroup Y. Overlap with Subgroup X was observed for 24% of Subgroup Y probe sets, and for all but 3% (centered on antigen receptor mediated pathways), the direction of change with *exacerbation* was the same in Subgroups X and Y. These observations, together with the important role innate immunity is known to play in priming adaptive immunity [67], suggest a complex interplay between both these arms of the immune system during the course of an asthma exacerbation. Consistent with this view are the examples in this study of predominance of innate immune pathways at a single sampling point during one exacerbation, and predominance of adaptive immune pathways at a single sampling point during a different exacerbation from the same patient.

Subgroup Z comprised the largest number of samples, but also contained the *exacerbation* samples that differed least from the *quiet* samples, both in terms of the number of differentiating probe sets (286) and the significance of the detected differences. A number of the probes-sets identified in Subgroup Z overlapped with probe sets in the other subgroups, but the significance of the association was almost always much less in subgroup Z. Also the direction of change was often in opposite direction, perhaps suggesting that these exacerbations were sampled at a time when homing between periphery and tissue was at a different phase. Pathway analysis on the 75 genes uniquely identified in Subgroup Z unfortunately did not identify any dominant biological processes. We did not seek to determine if reduction in stringency of selection criteria would have pointed towards particular pathway(s). Such a relaxation of the standards might have given hints implicating various biological pathways, but would also have resulted in identification of a large number of confounding false positives.

Extensive analysis was conducted in a search for parameters associated with the assignment by K- means of a particular *exacerbation* sample to a particular subgroup. *Exacerbation* samples from a single donor were not necessarily assigned to the same subgroups, indicating that subgroup assignment cannot be solely determined by an invariant characteristic of the patient. However, the relatively stable patient characteristic of BMI had a significant association with subgroup assignment. Mean BMI is statistically significantly lower (p = 0.006) in Subgroup X than Subgroup Y, and is statistically suggestively lower (p = 0.0501) in Sub-group Z than in Subgroup Y. These results suggest that subjects with lowest BMI tended to have the most pronounced pro-inflammatory gene expression profile. The gene expression changes observed in the group with the significantly higher BMI tended to be of less magnitude, and with less evidence of involvement of the innate immune system than those in Subgroup X. Haldar et al [28] have reported that those of an obese non-eosinophilic asthmatic phenotype tended to cluster based on similarities in clinical parameters such as response to therapy. The authors suggested that, based on differences between the obese and other groups observed in the parameters they studied, the difference between the obese group and the other groups “may provide a reliable framework for exploratory molecular and genetic studies, presently undermined by population heterogeneity”. The patients in Subgroup Y of this study have lower mean BMI (32.4) than the obese group in the Haldar et al study (36.2), but the finding of a BMI influence on asthma phenotypes is common to both studies.

The comparative weakness of the gene expression signature in Subgroup Z led us to a number of hypotheses which were then tested by calculating the significance of association between subgroup assignment and a given clinical parameter. For instance there was no association between subgroup assignment and: 1) severity of exacerbation as indicated by spirometry, 2) asthma severity level, 3) time between exacerbation onset and sample collection, 4) physician noted symptoms of respiratory infection, 5) medication use 6) ethnicity, or 7) country of residence. A hypothesis to explain subgroup assignment that is not ruled out by the available data is that some combination of these covariates acts together to influenced subgroup assignment. We did not have information on some potentially relevant covariates such as the level of exposure to exacerbation trigger and the degree of sensitivity to various triggers. The study was not large enough to support combinatorial analyses on the data available for other covariates. The available information has not provided insight into how to predict which exacerbation-associated gene expression pattern described here would be expressed by any particular *exacerbation* sample. One covariate identified as significantly associated with subgroup assignment was the mean number of days between *quiet* and *exacerbation* visits. As shown in Table 4, there was a shorter time between a *quiet* visit and the subsequent *exacerbation* visit for samples in Subgroups X and Y (median days 40.5 and 40 respectively) than for samples in Subgroup Z (median days 69). The time interval between visits for Subgroup Z (median 69 days, average 79.6 days) indicates that many Subgroup Z exacerbations occurred within the window of a scheduled *quiet* visit. It therefore seems likely that the explanation for this longer interval in Subgroup Z was that these exacerbation samples came from patients less likely to have sought urgent care for the exacerbation, but nevertheless met the study criteria for *exacerbation* visit. We hypothesize that, although the severity of these exacerbations did not differ with respect to the objective measures captured in the database, the patients felt less impacted by Subgroup Z exacerbations than by exacerbations assigned to either of the other two subgroups. This hypothesis is consistent with the molecular profile showing a much diminished *exacerbation* molecular signature in Subgroup Z. Also consistent with this interpretation of the data is that Subgroup Z exacerbations may represent a type of exacerbation that slowly worsens, while Subgroup X and Y exacerbation more acutely impact the patient, and have a shorter “build-up” phase.

This study has provided proof of concept that systemic changes associated with asthma exacerbation can be studied in the blood. In addition to showing that the involvement of biological processes with well-established roles in asthma can be detected in the blood, the study has also provided new insights such as the significant involvement of the IL15 pathway, and activation of innate immune pathways in the absence of apparent symptoms of respiratory infections. Perhaps the greatest impact of our study will come from the foundation it has laid for future studies, in particular comparative studies between healthy and asthmatic subjects during the course of respiratory infection. Further investigation could also be aimed at an understanding of transitions in gene activation that occur over the course of an exacerbation, from initiation to resolution. These type of data could be targeted at a selected set of genes, and seek to distinguish between processes that exacerbate disease, and processes that are actually associated with the resolution of such exacerbations.

## Supporting Information

Figure S1 **Distribution of 384 Quiet Samples from 118 Subjects.** Three or more *quiet* samples were analyzed from the majority (84%) of the 118 subjects with *exacerbation* samples, with 3 samples from 38% of subjects, 4 samples from 40% of subjects, and 5 samples analyzed from 6% of subjects. Two quiet samples were analyzed from 12% of the subjects, and only 1 quiet sample was available for the remaining 3%.
(DOC)

Figure S2 **Concordance of Results Using GeneChip and TaqMan® Platforms.** A strong correlation was observed between expression levels as measured by Affymetrix U133A GeneChip and as measured by TaqMan® Low Density Array. Differences in expression between paired samples as observed in the two platforms are shown. Signal sample pair differences (log 2 from GeneChip) are shown on the X axis, and delta CT sample pair differences (from TaqMan®) on the Y axis.
(DOC)

Figure S3 **Silhouette Statistic.** The silhouette statistics for K = 2, K = 3, K = 4 and K = 8 are shown.
(DOC)

Figure S4 **Robustness Statistics.** The larger drop in robustness statistic (R) from K = 3 to K = 4, compared to either the K = 1–2 or K = 4–8 drops, is shown indicating that increasing from 3 to 4 clusters markedly reduced the robustness of the cluster assignments to simulated experimental noise.
(DOC)

Figure S5 **Visual Representation (Heat Map) Of Exacerbation Related Gene Expression differences.** Color representation of differences between gene expression levels in each of 166 *exacerbation* samples and the average of *quiet* samples from the same patient. Intensity of color indicates magnitude of *exacerbation*/average *quiet* log ratios. Red color indicates elevation in expression in *exacerbation*, and green represents a decrease.
(DOC)

Figure S6 **Relative FDR p-value Obtained From ANCOVA.** A. Subgroup X Samples Using Only Exacerbation Samples with Corresponding Follow-up Sample. Comparison of relative FDR p-values for association with exacerbation obtained using N = 30 *exacerbation* samples and N = 22 *exacerbation* samples for which a follow-up sample was available. As expected, there is in general a small reduction in significance with the smaller sample number, but relative FDR p-values are very similar. B. Relative FDR p-value Obtained From ANCOVA On Subgroup Y Samples Using Only Exacerbation Samples with Corresponding Follow-up Sample.Comparison of relative FDR p-values for association with exacerbation obtained using N = 64 *exacerbation* samples and N = 51 *exacerbation* samples for which a follow-up sample was available. As expected, there is in general a small reduction in significance with the smaller sample number, but relative FDR p-values are very similar. C. Relative FDR p-value Obtained From ANCOVA On Subgroup Z Samples Using Only Exacerbation Samples with Corresponding Follow-up Sample. Results of ANCOVA indicate the lack of a robust gene expression pattern (in comparison to Subgroups X and Y) associated with Subgroup Z exacerbations. In the analysis using the 52 exacerbation samples for which a corresponding follow-up sample was available, the FDRs in the *Quiet* versus *Exacerbation* analysis is, as expected, less significant than the FDRs obtained with the larger sample set (N = 72).
(DOC)

Figure S7 **Subgroup Assignment and Days Between Exacerbation Onset and Exacerbation Sample Collection.** Results (in box plot format) of analysis showing lack of association between days between exacerbation onset and collection of *exacer*bation sample.
(DOC)

Table S1 **Post-CPT purification monocyte and lymphocyte percent in** ***quiet*** **and** ***exacerbation*** **visits.**
(DOC)

Table S2 **Quality control criteria for inclusion of GeneChip in analysis.**
(DOC)

Table S3 **Genes analyzed by Taqman with assay identification.**
(DOC)

Table S4 **Demographic and baseline characteristics by asthma severity.**
(DOC)

Table S5 **Global assessment of asthma control by the subject and by the investigator at screening.**
(DOC)

Table S6 **Reported asthma healthcare resource use before enrollment.**
(DOC)

Table S7 **Atopy status at screening.**
(DOC)

Table S8 **Body mass index and gastrointestinal reflux disease.**
(DOC)

Table S9 **History of reflux disease.**
(DOC)

Table S10 **Subjects with change in asthma severity by visit.**
(DOC)

Table S11 **Number (%) subjects who used concomitant anti-asthmatic medications by asthma severity**.
(DOC)

Table S12 **Number (%) of subjects who used concomitant anti-asthmatic medications by country.**
(DOC)

Table S13 **Reported asthma healthcare resource use during the study.**
(DOC)

Table S14 **Asthma precipitating or aggravating factors.**
(DOC)

Table S15 **Number (%) of subjects experiencing adverse events.**
(DOC)

Table S16 **Most common (≥10% of subjects in any severity group) respiratory adverse events, number (%) of subjects.**
(DOC)

Table S17 **Mean FEV1 (% predicted) at scheduled non-exacerbation visits.**
(DOC)

Table S18 **ANCOVA results. A: subgroup X. B: subgroup Y C: subgroup Z.**
(DOC)

Table S19 **IL15 pathway genes associated with exacerbation in subgroup X.**
(DOC)

Table S20 **Lack of subgroup association with FEV1 predicted.**
(DOC)

Table S21 **Lack of subgroup association with FEV1 (predicted) change from baseline.**
(DOC)

Table S22 **Lack of subgroup association with FVC (predicted).**
(DOC)

Table S23 **Lack of subgroup association with FVC (predicted) change from baseline.**
(DOC)

Table S24 **Lack of subgroup association with FEF 25–75% (predicted).**
(DOC)

Table S25 **Lack of subgroup association with FEF 25–75% (predicted) change from baseline.**
(DOC)

Table S26 **Lack of subgroup association with PEF (predicted) change from baseline.**
(DOC)

Table S27 **Lack of subgroup association with PEF (predicted) change from baseline.**
(DOC)

Table S28 **Lack of subgroup association with relevant respiratory infection.**
(DOC)

Table S29 **Lack of subgroup association with disease severity.**
(DOC)

Table S30 **Lack of subgroup association with use of medication: systemic corticosteroids.**
(DOC)

Table S31 **Lack of subgroup association with use of medication: inhaled corticosteroids.**
(DOC)

Table S32 **Lack of subgroup association with use of medication: intranasal corticosteroids.**
(DOC)

Table S33 **Lack of subgroup association with use of medication: leukotriene antagonists.**
(DOC)

Table S34 **Lack of subgroup association with use of medication: Any GI non-study medication** **use.**
(DOC)

Table S35 **Lack of subgroup association with use of medication: Any PPI non-study medication use.**
(DOC)

Table S36 **Lack of subgroup association with use of medication: any histamine H2 antagonist non-study medication use.**
(DOC)

Table S37 **Lack of subgroup association with sex.**
(DOC)

Table S38 **Subgroup assignment is not associated with race.**
(DOC)

Table S39 **Subgroup assignment is not associated with laboratory in which the samples were processed.**
(DOC)

Table S40 **Subgroups assignment is not associated with patients' country of residence.**
(DOC)

Table S41 **Subgroup assignment is not associated with atopy status.**
(DOC)

Table S42 **Subgroup assignment is not associated with fasting status.**
(DOC)

Table S43 **Subgroup assignment is not associated with IgE titers.**
(DOC)

Table S44 **Subgroups assignment is not associated with medical history of acid reflux.**
(DOC)

Table S45 **Association between BMI (at screening) and subgroup assignment.**
(DOC)

Table S46 **Association between subgroup assignment and days since quiet visit.**
(DOC)

Text S1 **Extensive details of the study are provided in this 101 page document.**
(DOC)
