# Supplementary material for: Pathways Activated during Human Asthma Exacerbation as Revealed by Gene Expression Patterns in Blood
Source: PLoS One. 2011 Jul 14;6(7):e21902. doi: 10.1371/journal.pone.0021902 (PMC3136489; doi:10.1371/journal.pone.0021902)
Supplement: Figure S1 — Distribution of 384 Quiet Samples from 118 Subjects. Three or more quiet samples were analyzed from the majority (84%) of the 118 subjects with exacerbation samples, with 3 samples from 38% of subjects, 4 samples from 40% of subjects, and 5 samples analyzed from 6% of subjects. Two quiet samples were analyzed from 12% of the subjects, and only 1 quiet sample was available for the remaining 3%. (DOC) [file pone.0021902.s001.doc]

## Online Supporting Information Figure S1: Distribution of 384 Quiet Samples from 118 Subjects

Three or more *quiet* samples were analyzed from the majority (84%) of the 118 subjects with *exacerbation* samples, with 3 samples from 38% of subjects, 4 samples from 40% of subjects, and 5 samples analyzed from 6% of subjects. Two quiet samples were analyzed from 12% of the subjects, and only 1 quiet sample was available for the remaining 3%.
