# Supplementary material for: Pathways Activated during Human Asthma Exacerbation as Revealed by Gene Expression Patterns in Blood
Source: PLoS One. 2011 Jul 14;6(7):e21902. doi: 10.1371/journal.pone.0021902 (PMC3136489; doi:10.1371/journal.pone.0021902)
Supplement: Figure S2 — Concordance of Results Using GeneChip and TaqMan® Platforms. A strong correlation was observed between expression levels as measured by Affymetrix U133A GeneChip and as measured by TaqMan® Low Density Array. Differences in expression between paired samples as observed in the two platforms are shown. Signal sample pair differences (log 2 from GeneChip) are shown on the X axis, and delta CT sample pair differences (from TaqMan®) on the Y axis. (DOC) [file pone.0021902.s002.doc]

## Online Supporting Information Figure S2: Concordance of Results Using GeneChip and Taqman Platforms


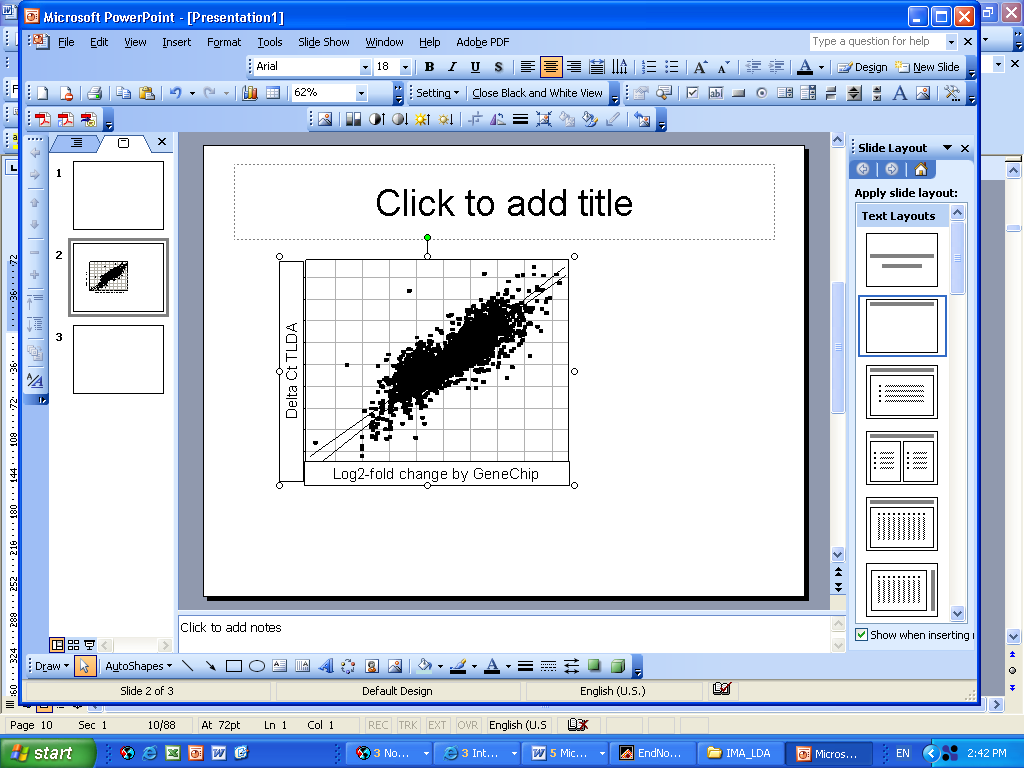


A strong correlation was observed between expression levels as measured by Affymetrix U133A GeneChip and as measured by TaqMan®  Low Density Array. Differences in expression between paired samples as observed in the two platforms are shown. Signal sample pair differences (log 2 from GeneChip) are shown on the X axis, and delta CT sample pair differences (from TaqMan®) on the Y axis.
