# Supplementary material for: Pathways Activated during Human Asthma Exacerbation as Revealed by Gene Expression Patterns in Blood
Source: PLoS One. 2011 Jul 14;6(7):e21902. doi: 10.1371/journal.pone.0021902 (PMC3136489; doi:10.1371/journal.pone.0021902)
Supplement: Figure S3 — Silhouette Statistic. The silhouette statistics for K = 2, K = 3, K = 4 and K = 8 are shown. (DOC) [file pone.0021902.s003.doc]

## Online Supporting Information Figure S3. Silhouette Statistic for K = 2 through K = 8 Clusters


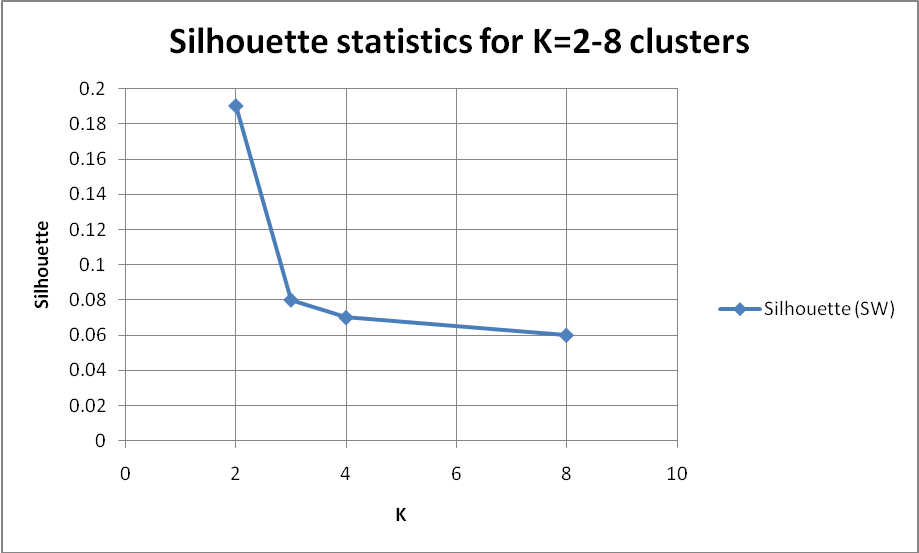


The silhouette statistic for K = 2, K = 3, K = 4 and K = 8 is shown.
