# Supplementary material for: Pathways Activated during Human Asthma Exacerbation as Revealed by Gene Expression Patterns in Blood
Source: PLoS One. 2011 Jul 14;6(7):e21902. doi: 10.1371/journal.pone.0021902 (PMC3136489; doi:10.1371/journal.pone.0021902)
Supplement: Figure S4 — Robustness Statistics. The larger drop in robustness statistic (R) from K = 3 to K = 4, compared to either the K = 1–2 or K = 4–8 drops, is shown indicating that increasing from 3 to 4 clusters markedly reduced the robustness of the cluster assignments to simulated experimental noise. (DOC) [file pone.0021902.s004.doc]

## Online Supporting Information Figure S4. Robustness statistics for K = 2 through K = 8


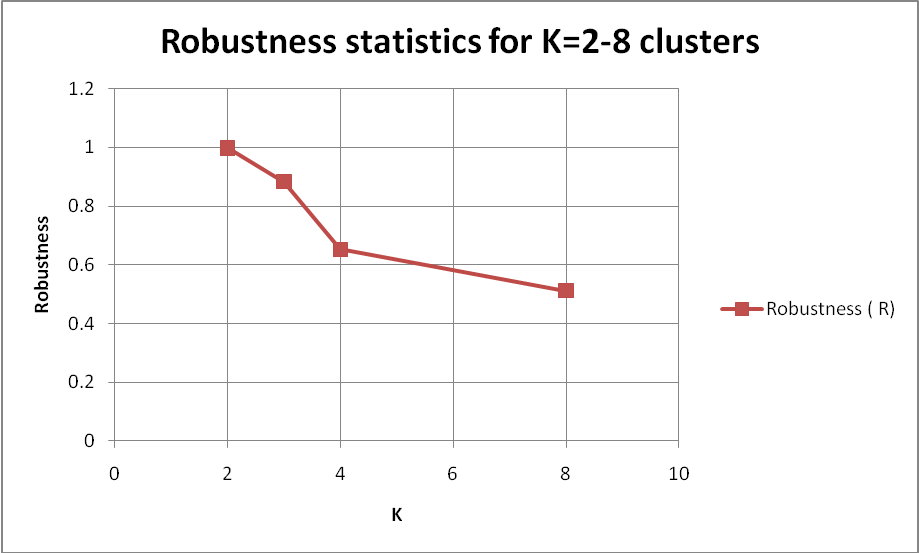


The larger drop in robustness statistic ( R) from K=3 to K=4, compared to either the K=1-2 or K=4-8 drops, is shown indicating that increasing from 3 to 4 clusters markedly reduced the robustness of the cluster assignments to simulated experimental noise.
