# Supplementary material for: Pathways Activated during Human Asthma Exacerbation as Revealed by Gene Expression Patterns in Blood
Source: PLoS One. 2011 Jul 14;6(7):e21902. doi: 10.1371/journal.pone.0021902 (PMC3136489; doi:10.1371/journal.pone.0021902)
Supplement: Figure S5 — Visual Representation (Heat Map) Of Exacerbation Related Gene Expression differences. Color representation of differences between gene expression levels in each of 166 exacerbation samples and the average of quiet samples from the same patient. Intensity of color indicates magnitude of exacerbation/average quiet log ratios. Red color indicates elevation in expression in exacerbation, and green represents a decrease. (DOC) [file pone.0021902.s005.doc]

## Online Supporting Information Figure S5: Visual Representation of Differences Between Gene Expression Levels in Each of 166 Exacerbation Samples and the Average of Quiet Samples from the Same Patient.


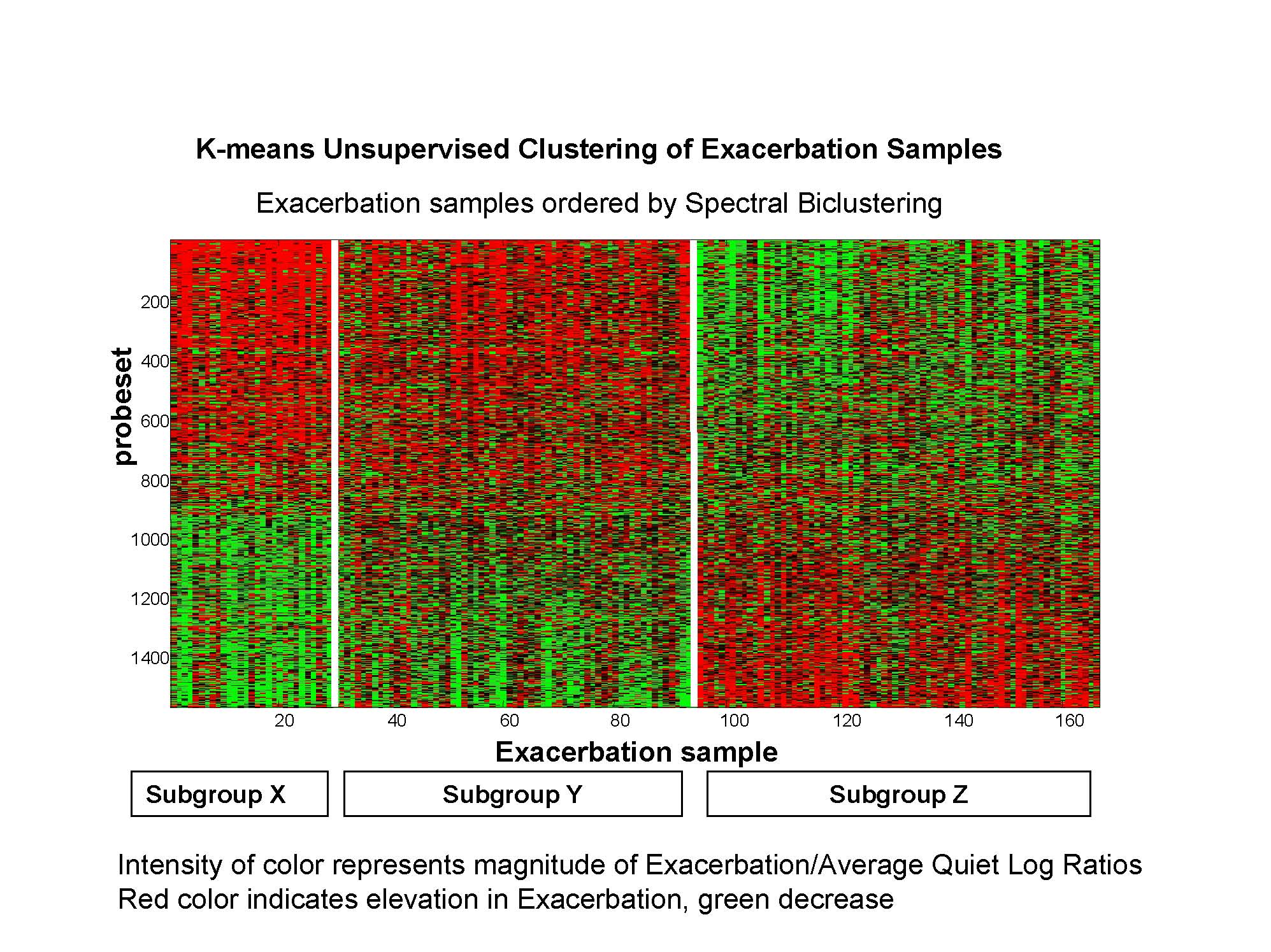


Color representation of differences between gene expression levels in each of 166 *exacerbation* samples and the average of *quiet* samples from the same patient. Intensity of color indicates magnitude of *exacerbation*/average *quiet* log ratios. Red color indicates elevation in expression in *exacerbation*, and green represents a decrease
