# Supplementary material for: Pathways Activated during Human Asthma Exacerbation as Revealed by Gene Expression Patterns in Blood
Source: PLoS One. 2011 Jul 14;6(7):e21902. doi: 10.1371/journal.pone.0021902 (PMC3136489; doi:10.1371/journal.pone.0021902)
Supplement: Figure S6 — Relative FDR p-value Obtained From ANCOVA. A. Subgroup X Samples Using Only Exacerbation Samples with Corresponding Follow-up Sample. Comparison of relative FDR p-values for association with exacerbation obtained using N = 30 exacerbation samples and N = 22 exacerbation samples for which a follow-up sample was available. As expected, there is in general a small reduction in significance with the smaller sample number, but relative FDR p-values are very similar. B. Relative FDR p-value Obtained From ANCOVA On Subgroup Y Samples Using Only Exacerbation Samples with Corresponding Follow-up Sample.Comparison of relative FDR p-values for association with exacerbation obtained using N = 64 exacerbation samples and N = 51 exacerbation samples for which a follow-up sample was available. As expected, there is in general a small reduction in significance with the smaller sample number, but relative FDR p-values are very similar. C. Relative FDR p-value Obtained From ANCOVA On Subgroup Z Samples Using Only Exacerbation Samples with Corresponding Follow-up Sample. Results of ANCOVA indicate the lack of a robust gene expression pattern (in comparison to Subgroups X and Y) associated with Subgroup Z exacerbations. In the analysis using the 52 exacerbation samples for which a corresponding follow-up sample was available, the FDRs in the Quiet versus Exacerbation analysis is, as expected, less significant than the FDRs obtained with the larger sample set (N = 72). (DOC) [file pone.0021902.s006.doc]

# Figure S6: Relative FDR p-value Obtained From ANCOVA

# Subgroup X Samples Using Only Exacerbation Samples with Corresponding Follow-up Sample


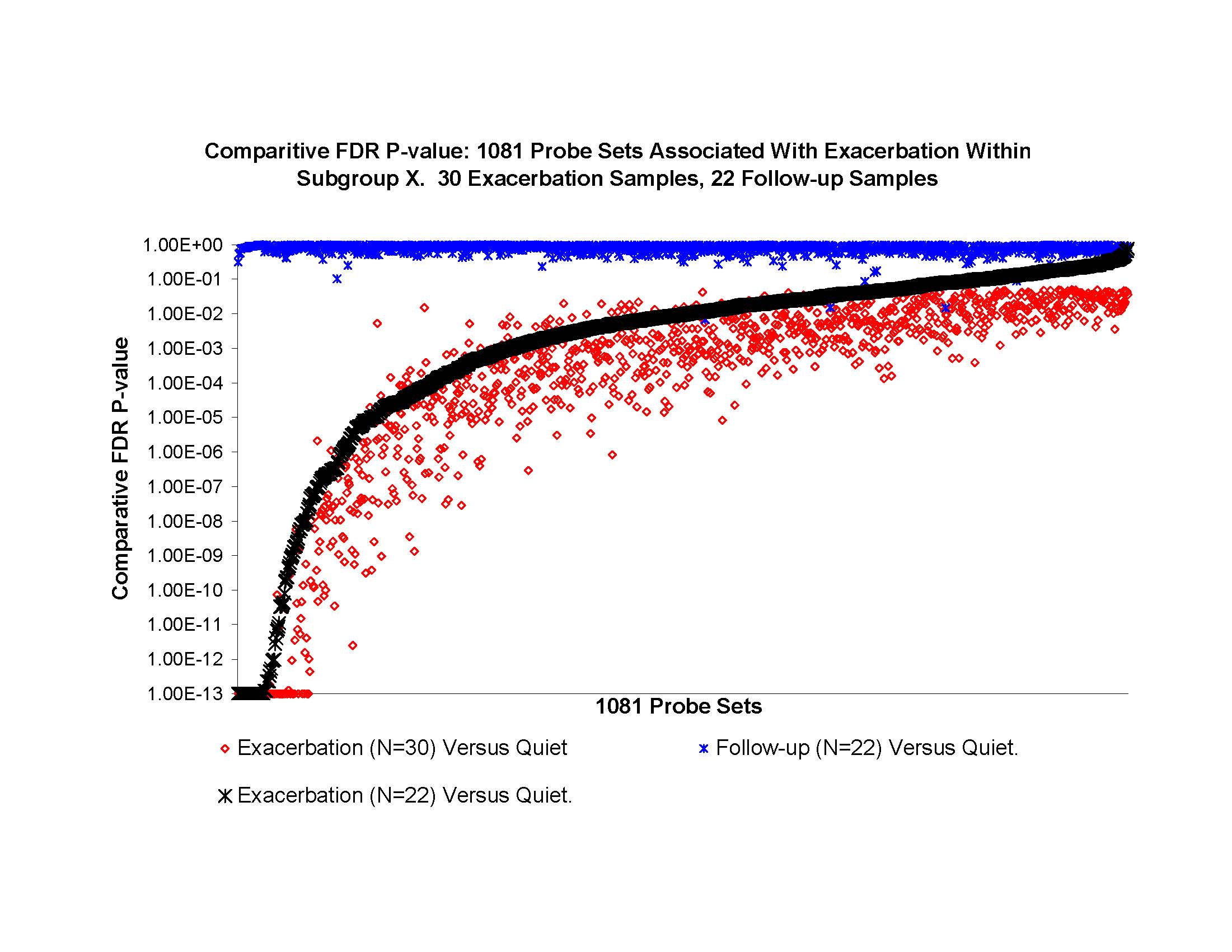


Comparison of relative FDR p-values for association with exacerbation obtained using N= 30 *exacerbation* samples and N = 22 *exacerbation* samples for which a follow-up sample was available. As expected, there is in general a small reduction in significance with the smaller sample number, but relative FDR p-values are very similar.

# Subgroup Y Samples Using Only Exacerbation Samples with Corresponding Follow-up Sample


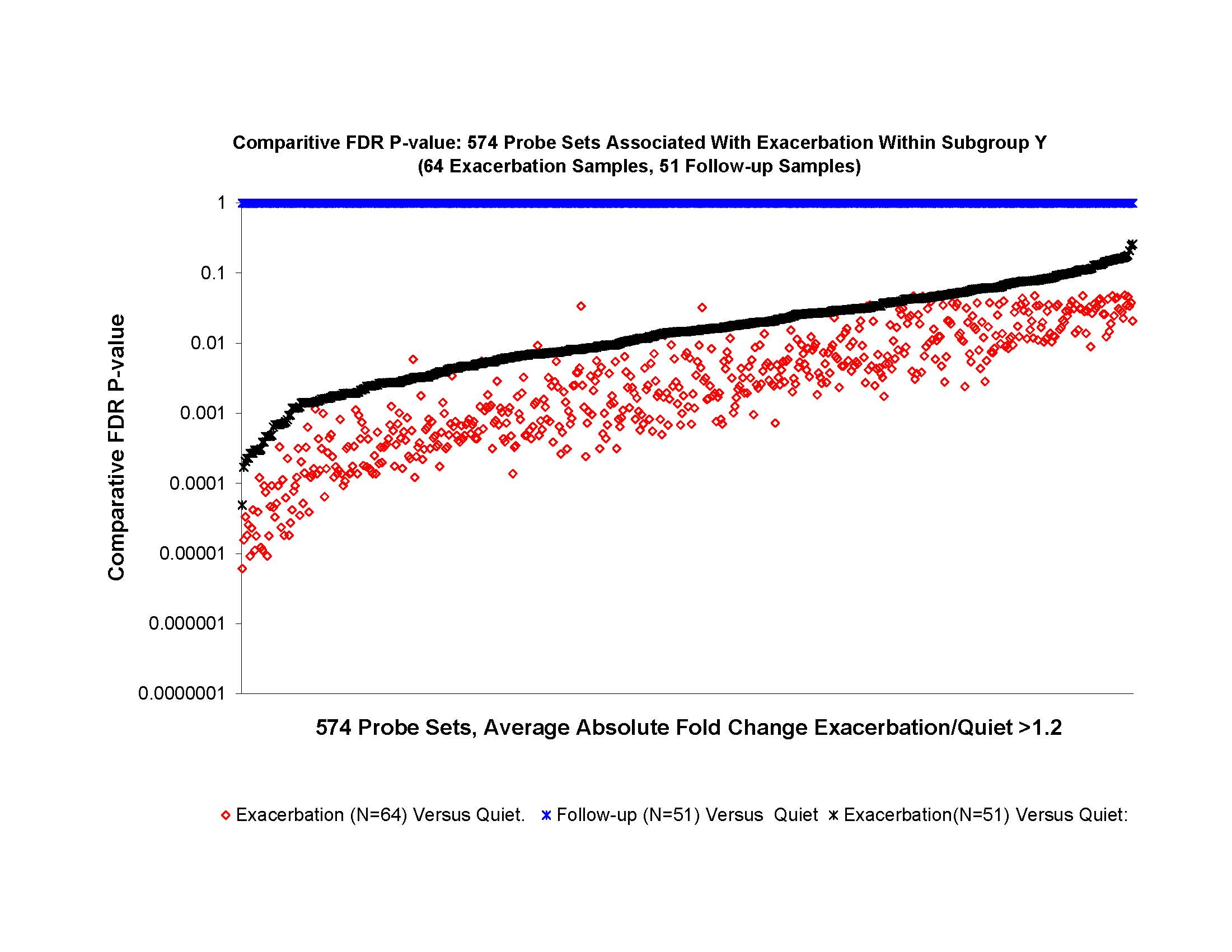


Comparison of relative FDR p-values for association with exacerbation obtained using N= 64 *exacerbation* samples and N = 51 *exacerbation* samples for which a follow-up sample was available. As expected, there is in general a small reduction in significance with the smaller sample number, but relative FDR p-values are very similar.

# Subgroup Z Samples Using Only Exacerbation Samples with Corresponding Follow-up Sample.


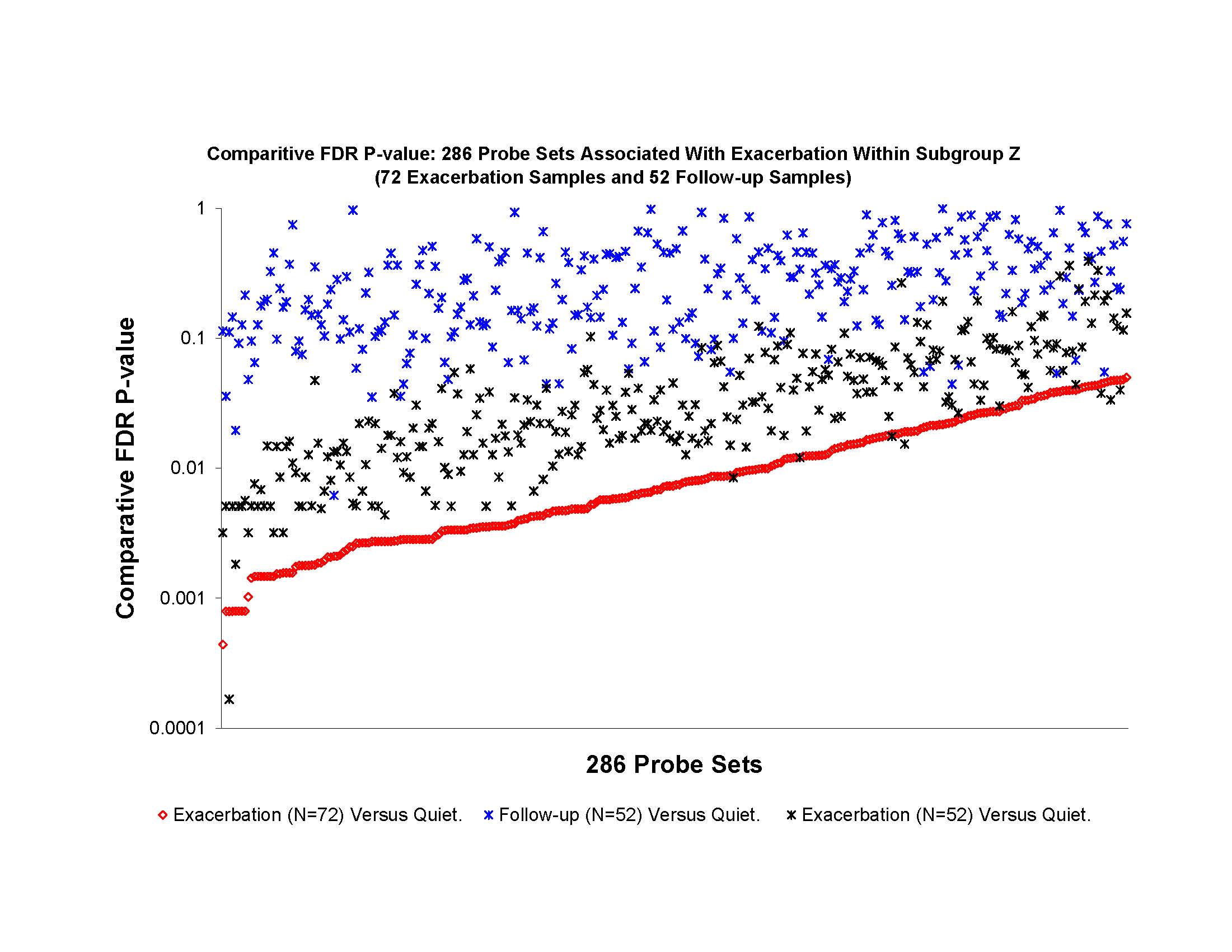


Results of ANCOVA indicating the lack of a robust gene expression pattern (in comparison to Subgroups X and Y) associated with Subgroup Z exacerbations. In the analysis using the 52 exacerbation samples for which a corresponding follow-up sample was available, the FDRs in the *Quiet* versus *Exacerbation* analysis is, as expected, less significant than the FDRs obtained with the larger sample set (N = 72).
