# Supplementary material for: Pathways Activated during Human Asthma Exacerbation as Revealed by Gene Expression Patterns in Blood
Source: PLoS One. 2011 Jul 14;6(7):e21902. doi: 10.1371/journal.pone.0021902 (PMC3136489; doi:10.1371/journal.pone.0021902)
Supplement: Figure S7 — Subgroup Assignment and Days Between Exacerbation Onset and Exacerbation Sample Collection. Results (in box plot format) of analysis showing lack of association between days between exacerbation onset and collection of exacerbation sample. (DOC) [file pone.0021902.s007.doc]

## Online Supporting Information Figure S7. Subgroup Assignment and Days Between Exacerbation Onset and Exacerbation Sample Collection


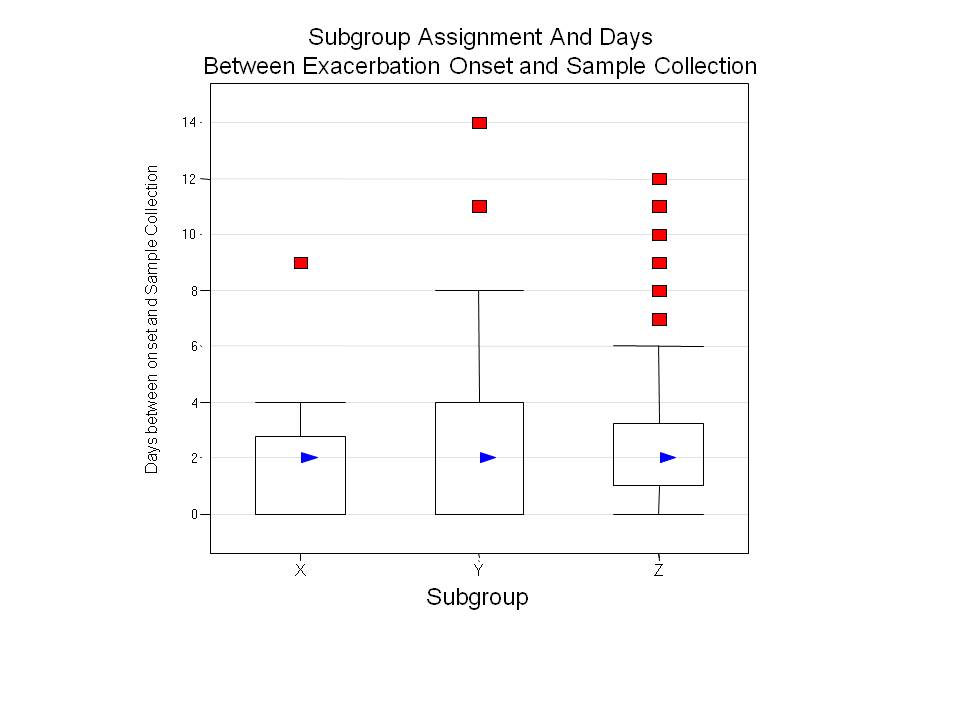


Results of analysis showing lack of association between days between exacerbation onset and collection of *exacerbation* sample.
