# Supplementary material for: Pathways Activated during Human Asthma Exacerbation as Revealed by Gene Expression Patterns in Blood
Source: PLoS One. 2011 Jul 14;6(7):e21902. doi: 10.1371/journal.pone.0021902 (PMC3136489; doi:10.1371/journal.pone.0021902)
Supplement: Table S1 — Post-CPT purification monocyte and lymphocyte percent in quiet and exacerbation visits. (DOC) [file pone.0021902.s008.doc]

## Online Supporting Information Table S1: Post-CPT Purification Monocyte and Lymphocyte Percent in Quiet and Exacerbation Visits.

|  | Quiet | | | Exacerbation | | |
| --- | --- | --- | --- | --- | --- | --- |
|  | mean | s.d. | n | mean | s.d. | n |
| Monocyte percent | 20.97 | 12.49 | 309 | 26.14 | 13.13 | 134 |
|  |  |  |  |  |  |  |
| Lymphocyte percent | 64.79 | 18.52 | 309 | 60.02 | 17.53 | 134 |
