# Supplementary material for: Pathways Activated during Human Asthma Exacerbation as Revealed by Gene Expression Patterns in Blood
Source: PLoS One. 2011 Jul 14;6(7):e21902. doi: 10.1371/journal.pone.0021902 (PMC3136489; doi:10.1371/journal.pone.0021902)
Supplement: Table S2 — Quality control criteria for inclusion of GeneChip in analysis. (DOC) [file pone.0021902.s009.doc]

## Online Supporting Information Table S2: Quality Control Criteria for Inclusion of GeneChip in Analysis

| 1 | Defect on visual inspection |  |
| --- | --- | --- |
| 2 | Bactin Gapdh Freq Avg Exp | > 0.6 |
| 3 | Genechip Raw Q Exp | < 7 |
| 4 | Qc P Prob Freq Exp | < 20 |
| 5 | Qc P Prob Avg Diff Exp | < 205 |
| 6 | Qc Sensitivity Exp | < 6.1 |
| 7 | Scale Factor Exp | < 4 and > 0.25 |
|  |  |  |
| 1 | Defect on visual inspection: Patterns in chip fluorescence visible after the chip has been run that reveal scratches, uneven staining or other defects. |  |
| 2 | Ratio of signal portion of the gene. A measure of the integrity of the RNA sample. |  |
| 3 | Raw Q: measure of the noise level of the array, it is the degree of pixel-to-pixel variation among the probe cells used to calculate the background. |  |
| 4 | QCP probability average difference: signal value for which there is a 70% probability of a Present call. |  |
| 5 | QCP probability frequency: QCP probability average difference expressed in ppm units. |  |
| 6 | Chip sensitivity: concentration level, in ppm, at which there is a 70% probability of obtaining a Present call. |  |
| 7 | Scale factor: the value required to obtain a trimmed mean intensity indicated by the target value. For all data in this study, the target value was set to a value of 100 and the scale factor was determined by dividing the trimmed mean of all probe sets by the target value. |  |
