# Supplementary material for: Pathways Activated during Human Asthma Exacerbation as Revealed by Gene Expression Patterns in Blood
Source: PLoS One. 2011 Jul 14;6(7):e21902. doi: 10.1371/journal.pone.0021902 (PMC3136489; doi:10.1371/journal.pone.0021902)
Supplement: Table S3 — Genes analyzed by Taqman with assay identification. (DOC) [file pone.0021902.s010.doc]

## Online Supporting Information Table S3: Genes Analyzed by Taqman.

| **Gene**  **Symbol** | **Applied Biosystems, Catalog #** | **Interrogated**  **Sequence** | **Exon Boundary** | **Probe**  **Binding Location** | **Tissue source for standard curve** |
| --- | --- | --- | --- | --- | --- |
| IFNα1 | Hs00256882_s1 | NM_024013 | 1-1 | 649 | Cervix tumor,  Ambion |
| IFNβ1 | Hs00277188_s1 | NM_002176 | 1-1 | 637 | Human monocyte, activated at Wyeth |
| IFNγ | Hs00174143_m1 | NM_000619 | 1-2 | 242 | Human PBMC activated at Wyeth |
| IL-13 | Hs00174379_m1 | NM_002188 | 1-2 | 192 | Thymus Ambion |
