# Supplementary material for: Pathways Activated during Human Asthma Exacerbation as Revealed by Gene Expression Patterns in Blood
Source: PLoS One. 2011 Jul 14;6(7):e21902. doi: 10.1371/journal.pone.0021902 (PMC3136489; doi:10.1371/journal.pone.0021902)
Supplement: Table S4 — Demographic and baseline characteristics by asthma severity. (DOC) [file pone.0021902.s011.doc]

| Online Supporting Information Table S4: Demographic and Baseline Characteristics by Asthma Severity | | | | | |
| --- | --- | --- | --- | --- | --- |
|  |  | Asthma Severity | | |  |
| Characteristic | *P*-value | Mild (n=36) | Moderate (n=149) | Severe (n=172) | Total (N=357) |
|  |  |  |  |  |  |
| n |  | 36 | 149 | 172 | 357 |
| Age (yr) Mean | 0.014[a](#aoneway) | 41.14 | 43.40 | 47.37 | 45.08 |
| Standard deviation |  | 12.85 | 15.18 | 14.71 | 14.88 |
| Sex n (%) | 0.449[b](#bfishers) |  |  |  |  |
| Female |  | 26 (72.2) | 98 (65.8) | 106 (61.6) | 230 (64.4) |
| Male |  | 10 (27.8) | 51 (34.2) | 66 (38.4) | 127 (35.6) |
| Race n (%) | 0.125[b](#bfishers) |  |  |  |  |
| Asian |  | 0 | 4 (2.7) | 6 (3.5) | 10 (2.8) |
| Black |  | 1 (2.8) | 11 (7.4) | 24 (14.0) | 36 (10.1) |
| White |  | 35 (97.2) | 134 (89.9) | 142 (82.6) | 311 (87.1) |
| Ethnicity n (%) | 0.120[b](#bfishers) |  |  |  |  |
| Hispanic |  | 0 | 3 (2.0) | 10 (5.8) | 13 (3.6) |
| Non-Hispanic |  | 36 (100 ) | 146 (98.0) | 162 (94.2) | 344 (96.4) |
| Weight (kg) |  |  |  |  |  |
| n |  | 36 | 149 | 170 | 355 |
| Mean | 0.518[a](#aoneway) | 78.03 | 82.01 | 80.87 | 81.06 |
| Standard deviation |  | 13.29 | 18.83 | 19.88 | 18.86 |
| Height (cm) |  |  |  |  |  |
| n |  | 36 | 149 | 172 | 357 |
| Mean | 0.058[a](#aoneway) | 167.37 | 169.54 | 166.95 | 168.07 |
| Standard deviation |  | 10.39 | 10.08 | 9.47 | 9.88 |
| a One‑way analysis of variance with severity category as factor.  b Fisher's exact test *P*-value (2-tail) for comparison across asthma severity groups. | | | | | |
