# Supplementary material for: Pathways Activated during Human Asthma Exacerbation as Revealed by Gene Expression Patterns in Blood
Source: PLoS One. 2011 Jul 14;6(7):e21902. doi: 10.1371/journal.pone.0021902 (PMC3136489; doi:10.1371/journal.pone.0021902)
Supplement: Table S5 — Global assessment of asthma control by the subject and by the investigator at screening. (DOC) [file pone.0021902.s012.doc]

| Online Supporting Information Table S5: Global Assessment of Asthma Control by the Subject and by the Investigator at Screening | | | | |
| --- | --- | --- | --- | --- |
|  | Asthma Severity | | |  |
| Characteristic | Mild (n=36) | Moderate (n=149) | Severe (n=172) | Total (N=357) |
| Subject’s Assessment^a^ n (%) |  |  |  |  |
| Excellent | 13 (36.1) | 32 (21.5) | 24 (14.0) | 69 (19.3) |
| Good | 18 (50.0) | 77 (51.7) | 87 (50.6) | 182 (51.0) |
| Fair | 5 (13.9) | 33 (22.1) | 48 (27.9) | 86 (24.1) |
| Poor | 0 | 6 (4.0) | 13 (7.6) | 19 (5.3) |
| Investigator’s Assessment n (%) |  |  |  |  |
| Excellent | 16 (44.4) | 31 (20.8) | 19 (11.0) | 66 (18.5) |
| Good | 19 (52.8) | 85 (57.0) | 86 (50.0) | 190 (53.2) |
| Fair | 1 (2.8) | 30 (20.1) | 52 (30.2) | 83 (23.2) |
| Poor | 0 | 3 (2.0) | 15 (8.7) | 18 (5.0) |
| a The assessment was not done for 1 subject. | | | | |
