# Supplementary material for: Pathways Activated during Human Asthma Exacerbation as Revealed by Gene Expression Patterns in Blood
Source: PLoS One. 2011 Jul 14;6(7):e21902. doi: 10.1371/journal.pone.0021902 (PMC3136489; doi:10.1371/journal.pone.0021902)
Supplement: Table S6 — Reported asthma healthcare resource use before enrollment. (DOC) [file pone.0021902.s013.doc]

| Online Supporting Information Table S6: Reported Asthma Healthcare Resource Use Before Enrollment | | | | | | |
| --- | --- | --- | --- | --- | --- | --- |
|  |  | Asthma Severity | | | |  |
| Characteristic | *P*-value^a^ | Mild (n=36) | Moderate (n=149) | Severe (n=172) | Total (N=357) | |
| **Number of ER Visits n (%)** | 0.01 |  |  |  |  | |
| None |  | 32 (88.9) | 129 (86.6) | 117 (68.0) | 278 (77.9) | |
| 1-3 Times |  | 4 (11.1) | 18 (12.1) | 40 (23.3) | 62 (17.4) | |
| 4-6 Times |  | 0 | 1 (0.7) | 10 (5.8) | 11 (3.1) | |
| >6 Times |  | 0 | 1 (0.7) | 5 (2.9) | 6 (1.7) | |
| **Number of Exacerbations n (%)** | 0.009 |  |  |  |  | |
| None |  | 15 (41.7) | 55 (36.9) | 41 (23.8) | 111 (31.1) | |
| 1-3 Times |  | 15 (41.7) | 62 (41.6) | 67 (39.0) | 144 (40.3) | |
| 4-6 Times |  | 3 (8.3) | 19 (12.8) | 27 (15.7) | 49 (13.7) | |
| >6 Times |  | 3 (8.3) | 13 (8.7) | 37 (21.5) | 53 (14.8) | |
| **Oral Steroid Taper n (%)** | <0.001 |  |  |  |  | |
| None |  | 26 (72.2) | 78 (52.3) | 61 (35.5) | 165 (46.2) | |
| 1-3 Times |  | 10 (27.8) | 53 (35.6) | 66 (38.4) | 129 (36.1) | |
| 4-6 Times |  | 0 | 14 (9.4) | 21 (12.2) | 35 (9.8) | |
| >6 Times |  | 0 | 4 (2.7) | 24 (14.0) | 28 (7.8) | |
| **Near-Fatal Episode due to Asthma n (%)** | 0.023 |  |  |  |  | |
| None |  | 33 (91.7) | 116 (77.9) | 112 (65.1) | 261 (73.1) | |
| 1-3 Times |  | 3 (8.3) | 27 (18.1) | 51 (29.7) | 81 (22.7) | |
| 4-6 Times |  | 0 | 4 (2.7) | 4 (2.3) | 8 (2.2) | |
| >6 Times |  | 0 | 2 (1.3) | 5 (2.9) | 7 (2.0) | |
| **Intubation n (%)** | 0.010 |  |  |  |  | |
| None |  | 36 (100) | 143 (96.0) | 150 (87.2) | 329 (92.2) | |
| 1-3 Times |  | 0 | 6 (4.0) | 19 (11.0) | 25 (7.0) | |
| 4-6 Times |  | 0 | 0 | 3 (1.7) | 3 (0.8) | |
| **Hospital Admissions for Asthma n (%)** | <0.001 |  |  |  |  | |
| None |  | 26 (72.2) | 88 (59.1) | 68 (39.5) | 182 (51.0) | |
| 1-3 Times |  | 6 (16.7) | 32 (21.5) | 49 (28.5) | 87 (24.4) | |
| 4-6 Times |  | 3 (8.3) | 15 (10.1) | 20 (11.6) | 38 (10.6) | |
| >6 Times |  | 1 (2.8) | 14 (9.4) | 35 (20.3) | 50 (14.0) | |
| a Fisher's exact test *P*-value (2-tail).  Abbreviations: ER = emergency room | | | | | | |
