# Supplementary material for: Pathways Activated during Human Asthma Exacerbation as Revealed by Gene Expression Patterns in Blood
Source: PLoS One. 2011 Jul 14;6(7):e21902. doi: 10.1371/journal.pone.0021902 (PMC3136489; doi:10.1371/journal.pone.0021902)
Supplement: Table S7 — Atopy status at screening. (DOC) [file pone.0021902.s014.doc]

| Online Supporting Information Table S7: Atopy Status at Screening | | | | | |
| --- | --- | --- | --- | --- | --- |
|  |  | Asthma Severity | | |  |
| Characteristic | *P*-value | Mild (n=36) | Moderate (n=149) | Severe (n=172) | Total (N=357) |
| Atopy Status (history, total IgE) n (%) | 0.488^a^ |  |  |  |  |
| Atopic |  | 23 (63.9) | 100 (67.1) | 116 (67.4) | 239 (66.9) |
| Nonatopic |  | 5 (13.9) | 23 (15.4) | 34 (19.8) | 62 (17.4) |
| Atopy Status (Skin Test/RAST) n (%) | 0.017^a^ |  |  |  |  |
| Atopic |  | 14 (38.9) | 71 (47.7) | 59 (34.3) | 144 (40.3) |
| Nonatopic |  | 14 (38.9) | 52 (34.9) | 91 (52.9) | 157 (44.0) |
|  |  |  |  |  |  |
| Unknown |  | 8 (22.2) | 26 (17.4) | 22 (12.8) | 56 (15.7) |
| a Fisher's exact test *P*-value (2-tail) for comparison across asthma severity groups. | | | | | |
