# Supplementary material for: Pathways Activated during Human Asthma Exacerbation as Revealed by Gene Expression Patterns in Blood
Source: PLoS One. 2011 Jul 14;6(7):e21902. doi: 10.1371/journal.pone.0021902 (PMC3136489; doi:10.1371/journal.pone.0021902)
Supplement: Table S8 — Body mass index and gastrointestinal reflux disease. (DOC) [file pone.0021902.s015.doc]

## Online Supporting Information Table S8: Body Mass Index and Gastrointestinal Reflux Disease:

When demographic characteristics were analyzed by sex , body mass index in men varied significantly (*P*=0.037) by asthma severity.

| Body mass index |
| --- |

| **BMI (men)** |  |  |  |  |  |
| --- | --- | --- | --- | --- | --- |
| n | p | 10 | 51 | 65 | 126 |
| Mean | 0.037[a](#aoneway) | 26.01 | 27.73 | 29.58 | 28.55 |
| Standard deviation |  | 2.97 | 4.98 | 5.19 | 5.07 |
| **BMI (Women)** |  |  |  |  |  |
| n |  | 26 | 98 | 105 | 229 |
| Mean | 0.907[a](#aoneway) | 28.82 | 28.87 | 28.49 | 28.69 |
| Standard deviation |  | 5.95 | 6.18 | 6.72 | 6.39 |
| a One‑way analysis of variance with severity category as factor.  b Fisher's exact test *P*-value (2-tail) for comparison across asthma severity groups.  Abbreviations: BMI = body mass index | | | | | |
