# Supplementary material for: Pathways Activated during Human Asthma Exacerbation as Revealed by Gene Expression Patterns in Blood
Source: PLoS One. 2011 Jul 14;6(7):e21902. doi: 10.1371/journal.pone.0021902 (PMC3136489; doi:10.1371/journal.pone.0021902)
Supplement: Table S9 — History of reflux disease. (DOC) [file pone.0021902.s016.doc]

| Online Supporting Information Table S9: History of Reflux Disease | | | | | |
| --- | --- | --- | --- | --- | --- |
| Category | Overall *P*‑Value | Asthma Severity | | | Total (N=357) |
|  |  | Mild (n=36) | Moderate (n=149) | Severe (n=172) |  |
| Subjects with History of Reflux n (%) | 0.0232 | 4 (11.1) | 39 (26.2) | 56 (32.6) | 99 (27.7) |
| Men with Medical History Data |  | 10 | 51 | 66 | 127 |
| Men with History of Reflux n (%) | 0.6402 | 1 (10.0) | 14 (27.5) | 18 (27.3) | 33 (26.0) |
| Women with Medical History Data |  | 26 | 98 | 106 | 230 |
| Women with History of Reflux n (%) | 0.0302 | 3 (11.5) | 25 (25.5) | 38 (35.8) | 66 (28.7) |
| a Overall *P*-value: Fisher’s exact test P-value (2-tail) for comparison across severity groups. | | | | | |
