# Supplementary material for: Pathways Activated during Human Asthma Exacerbation as Revealed by Gene Expression Patterns in Blood
Source: PLoS One. 2011 Jul 14;6(7):e21902. doi: 10.1371/journal.pone.0021902 (PMC3136489; doi:10.1371/journal.pone.0021902)
Supplement: Table S10 — Subjects with change in asthma severity by visit. (DOC) [file pone.0021902.s017.doc]

| Online Supporting Information Table S10: Subjects with a Change in Asthma Severity by Visit | | | | | |
| --- | --- | --- | --- | --- | --- |
| Asthma Severity at Screening | Status | Visit 3 n (%) | Visit 4 n (%) | Visit 5 n (%) | Visit 6 n (%) |
| Mild (n=36) | Same | 27 (79.4) | 30 (88.2) | 26 (76.5) | 29 (90.6) |
|  | Changed | 7 (20.6) | 4 (11.8) | 8 (23.5) | 3 (9.4) |
| Moderate (n=149) | Same | 48 (33.6) | 52 (36.1) | 41 (29.1) | 42 (29.4) |
|  | Changed | 95 (66.4) | 92 (63.9) | 100 (70.9) | 101 (70.6) |
| Severe (n=172) | Same | 44 (26.8) | 37 (22.7) | 33 (20.6) | 31 (19.3) |
|  | Changed | 120 (73.2) | 126 (77.3) | 127 (79.4) | 130 (80.7) |
