# Supplementary material for: Pathways Activated during Human Asthma Exacerbation as Revealed by Gene Expression Patterns in Blood
Source: PLoS One. 2011 Jul 14;6(7):e21902. doi: 10.1371/journal.pone.0021902 (PMC3136489; doi:10.1371/journal.pone.0021902)
Supplement: Table S11 — Number (%) subjects who used concomitant anti-asthmatic medications by asthma severity. (DOC) [file pone.0021902.s018.doc]

| Online Supporting Information Table S11: Number (%) of Subjects Who Used Concomitant Anti-asthmatic Medications by Asthma Severity | | | | | |
| --- | --- | --- | --- | --- | --- |
|  |  | Asthma Severity | | |  |
| Characteristic | *P*-value^a^ | Mild (n=36) | Moderate (n=149) | Severe (n=172) | Total (N=357) |
| All Visits |  |  |  |  |  |
| Systemic corticosteroids | <0.0001 | 5 (13.9) | 63 (42.3) | 91 (52.9) | 159 (44.5) |
| Inhaled corticosteroids | <0.0001 | 30 (83.3) | 147 (98.7) | 168 (97.7) | 345 (96.6) |
| Intranasal corticosteroids | 0.3876 | 18 (50.0) | 79 (53.0) | 78 (45.4) | 175 (49.0) |
| Leukotriene antagonists | <0.0001 | 3 (8.3) | 36 (24.2) | 71 (41.2) | 110 (30.8) |
| Scheduled Non-Exacerbation Visits | | | | | |
| Systemic corticosteroids | <0.0001 | 0 | 33 (22.2) | 70 (40.7) | 103 (28.9) |
| Inhaled corticosteroids | <0.0001 | 29 (80.6) | 147 (98.7) | 168 (97.7) | 344 (96.4) |
| Intranasal corticosteroids | 0.2819 | 18 (50.0) | 79 (53.0) | 76 (44.2) | 173 (48.5) |
| Leukotriene antagonists | <0.0001 | 3 (8.3) | 35 (23.5) | 69 (40.1) | 107 (30.0) |
| a Chi-square test for comparison across asthma severity groups. | | | | | |
