# Supplementary material for: Pathways Activated during Human Asthma Exacerbation as Revealed by Gene Expression Patterns in Blood
Source: PLoS One. 2011 Jul 14;6(7):e21902. doi: 10.1371/journal.pone.0021902 (PMC3136489; doi:10.1371/journal.pone.0021902)
Supplement: Table S12 — Number (%) of subjects who used concomitant anti-asthmatic medications by country. (DOC) [file pone.0021902.s019.doc]

| Online Supporting Information Table S12: Number (%) of Subjects Who Used Concomitant Anti-asthmatic Medications by Country | | | | | | |
| --- | --- | --- | --- | --- | --- | --- |
|  |  | Country | | | | |
| Characteristic | *P*-value | Australia (n=73) | G Britain (n=51) | Ireland (n=35) | Iceland (n=61) | USA (n=137) |
| **All Visits** |  |  |  |  |  |  |
| Systemic corticosteroids | 0.0635^a^ | 28 (38.4) | 20 (39.2) | 13 (37.1) | 37 (60.7) | 61 (44.5) |
| Inhaled corticosteroids | 0.1030^b^ | 72 (98.6) | 48 (94.1) | 35 (100.0) | 61 (100.0) | 129 (94.2) |
| Intranasal corticosteroids | <0.0001^a^ | 19 (26.0) | 15 (29.4) | 18 (51.4) | 53 (86.9) | 70 (51.1) |
| Leukotriene antagonists | <0.0001^a^ | 6 (8.2) | 5 (9.8) | 14 (40.0) | 22 (36.1) | 63 (46.0) |
| **Scheduled Non-Exacerbation Visits** | | | | | | |
| Systemic corticosteroids | 0.8955 ^a^ | 18 (24.7) | 15 (29.4) | 10 (28.6) | 17 (27.9) | 43 (31.4) |
| Inhaled corticosteroids | 0.1770^b^ | 71 (97.3) | 48 (94.1) | 35 (100.0) | 61 (100.0) | 129 (94.2) |
| Intranasal corticosteroids | <0.0001^a^ | 18 (24.7) | 15 (29.4) | 17 (48.6) | 53 (86.9) | 70 (51.1) |
| Leukotriene antagonists | <0.0001^a^ | 5 (6.9) | 5 (9.8) | 14 (40.0) | 22 (36.1) | 61 (44.5) |
| a Chi-square test for comparison across asthma severity groups  b Fisher’s exact test for comparison across asthma severity groups | | | | | | |
