# Supplementary material for: Pathways Activated during Human Asthma Exacerbation as Revealed by Gene Expression Patterns in Blood
Source: PLoS One. 2011 Jul 14;6(7):e21902. doi: 10.1371/journal.pone.0021902 (PMC3136489; doi:10.1371/journal.pone.0021902)
Supplement: Table S13 — Reported asthma healthcare resource use during the study. (DOC) [file pone.0021902.s020.doc]

| Online Supporting Information Table S13: Reported Asthma Healthcare Resource Use During the Study (number of events reported) | | | | | |
| --- | --- | --- | --- | --- | --- |
|  |  | Asthma Severity | | |  |
| Characteristic | *P*-value^a^ | Mild (n=34) | Moderate (n=147) | Severe (n=169) | Total (N=350) |
| **Number of ER Visits n (%)** | 0.0690 | 0 | 7 (4.8) | 16 (9.5) | 23 (6.6) |
| 1-3 Times |  | 0 | 7 | 26 | 33 |
| **Number of Exacerbations n (%)** | 0.0043 | 12 (35.3) | 63 (42.9) | 99 (58.6) | 174 (49.7) |
| 1-3 Times |  | 22 | 128 | 243 | 393 |
| 4-6 Times |  | 0 | 2 | 2 | 4 |
| >6 Times |  | 0 | 2 | 0 | 2 |
| **Oral Steroid Taper n (%)** | 0.0028 | 5 (14.7) | 56 (38.1) | 76 (45.0) | 137 (39.1) |
| 1-3 Times |  | 8 | 85 | 180 | 273 |
| 4-6 Times |  | 0 | 0 | 2 | 2 |
| >6 Times |  | 0 | 1 | 5 | 6 |
| **Near-Fatal Episode n (%)** | 0.5932 | 0 | 0 | 2 (1.2) | 2 (0.6) |
| 1-3 Times |  | 0 | 0 | 2 | 2 |
| Intubation n (%) | 1.0000 | 0 | 0 | 1 (0.6) | 1 (0.3) |
| 1-3 Times |  | 0 | 0 | 1 | 1 |
| **Hospital Admissions n (%)** | 0.2243 | 0 | 6 (4.1) | 12 (7.1) | 18 (5.1) |
| 1-3 Times |  | 0 | 6 | 17 | 23 |
| a Fisher's exact test *P*-value (2-tail) for comparison across asthma severity groups.  room  Abbreviations: ER = emergency Source: | | | | | |
