# Supplementary material for: Pathways Activated during Human Asthma Exacerbation as Revealed by Gene Expression Patterns in Blood
Source: PLoS One. 2011 Jul 14;6(7):e21902. doi: 10.1371/journal.pone.0021902 (PMC3136489; doi:10.1371/journal.pone.0021902)
Supplement: Table S14 — Asthma precipitating or aggravating factors. (DOC) [file pone.0021902.s021.doc]

| Online Supporting Information Table S14: Asthma Precipitating or Aggravating Factors by Visit | | | | | |
| --- | --- | --- | --- | --- | --- |
| Characteristic | Visit 1 | Visit 3 | Visit 4 | Visit 5 | Visit 6 |
|  | Screening (N=357) | Wk 1-13 (N=341) | Wk 14-26 (N=341) | Wk 27-39 (N=335) | Wk 40-52 (N=335) |
| **Viral Respiratory Infections n (%)** |  |  |  |  |  |
| Yes | 325 (91.0) |  |  |  |  |
| No | 30 (8.4) |  |  |  |  |
| No Change Since Last Visit |  | 337 (98.8) | 340 (99.7) | 334 (99.7) | 335 (100) |
| **Changes in Weather, Exposure to Cold Air n (%)** |  |  |  |  |  |
| Yes | 307 (86.0) |  |  |  |  |
| No | 46 (12.9) |  |  |  |  |
| No Change Since Last Visit |  | 338 (99.1) | 341 (100) | 334 (99.7) | 334 (99.7) |
| **Exercise n (%)** |  |  |  |  |  |
| Yes | 290 (81.2) |  |  |  |  |
| No | 64 (17.9) |  |  |  |  |
| No Change Since Last Visit |  | 338 (99.1) | 341 (100) | 334 (99.7) | 334 (99.7) |
| **Environmental Allergens n (%)** |  |  |  |  |  |
| Yes | 283 (79.3) |  |  |  |  |
| No | 72 (20.2) |  |  |  |  |
| No Change Since Last Visit |  | 341 (100) | 341 (100) | 334 (99.7) | 335 (100) |
| **Smoke n (%)** |  |  |  |  |  |
| Yes | 278 (77.9) |  |  |  |  |
| No | 74 (20.7) |  |  |  |  |
| No Change Since Last Visit |  | 340 (99.7) | 339 (99.4) | 333 (99.4) | 335 (100) |
| **NSAIDs or Aspirin n (%)** |  |  |  |  |  |
| Yes | 46 (12.9) |  |  |  |  |
| No | 289 (81.0) |  |  |  |  |
| No Change Since Last Visit |  | 340 (99.7) | 341 (100) | 334 (99.7) | 335 (100) |
| Note: Subjects without a “yes” or “no” response were not exposed or exposure status was unknown (unknown was categorized as not exposed).  Abbreviations: NSAIDs = nonsteroidal antiinflammatory drugs; Wk = Weeks | | | | | |
