# Supplementary material for: Pathways Activated during Human Asthma Exacerbation as Revealed by Gene Expression Patterns in Blood
Source: PLoS One. 2011 Jul 14;6(7):e21902. doi: 10.1371/journal.pone.0021902 (PMC3136489; doi:10.1371/journal.pone.0021902)
Supplement: Table S15 — Number (%) of subjects experiencing adverse events. (DOC) [file pone.0021902.s022.doc]

| Online Supporting Information Table S15: Number (%) of Subjects Experiencing Adverse Events | | | | | |
| --- | --- | --- | --- | --- | --- |
| Adverse Event | Overall *P*‑Value^a^ | Asthma Severity | | | Total (N=357) |
|  |  | Mild (n=36) | Moderate (n=149) | Severe (n=172) |  |
| Any AE | 0.020* | 27 (75.0) | 126 (84.6) | 157 (91.3) | 310 (86.8) |
| Any study‑related AE | 0.365 | 0 | 2 (1.3) | 0 | 2 (0.6) |
| Any grade 3 or 4 AE^b^ | 0.035* | 1 (2.8) | 20 (13.4) | 32 (18.6) | 53 (14.8) |
| Any SAE | 0.169 | 2 (5.6) | 23 (15.4) | 31 (18.0) | 56 (15.7) |
| a Overall *P*-value: Fisher's exact test *P*-value (2-tail) for comparison across asthma severity groups. Statistical significance at the 0.05, 0.01, 0.001 levels is denoted by *, **, ***, respectively.  b Severe or life-threatening  Abbreviations: AE = adverse event; SAE = serious adverse event | | | | | |
