# Supplementary material for: Pathways Activated during Human Asthma Exacerbation as Revealed by Gene Expression Patterns in Blood
Source: PLoS One. 2011 Jul 14;6(7):e21902. doi: 10.1371/journal.pone.0021902 (PMC3136489; doi:10.1371/journal.pone.0021902)
Supplement: Table S16 — Most common (≥10% of subjects in any severity group) respiratory adverse events, number (%) of subjects. (DOC) [file pone.0021902.s023.doc]

| Online Supporting Information Table S16: Most Common (≥10% of Subjects in Any Severity Group) Respiratory Adverse Events, Number (%) of Subjects | | | | | |
| --- | --- | --- | --- | --- | --- |
|  | Overall P‑Value^a^ | Asthma Severity | | | Total (N=357) |
|  |  | Mild (n=36) | Moderate (n=149) | Severe (n=172) |  |
| Respiratory system | 0.033* | 22 (61.1) | 102 (68.5) | 135 (78.5) | 259 (72.5) |
| Cough increased | 0.868 | 13 (36.1) | 59 (39.6) | 71 (41.3) | 143 (40.1) |
| Dyspnea | 0.048* | 9 (25.0) | 50 (33.6) | 75 (43.6) | 134 (37.5) |
| Wheezing | 0.032* | 9 (25.0) | 36 (24.2) | 64 (37.2) | 109 (30.5) |
| Rhinitis | 0.716 | 11 (30.6) | 41 (27.5) | 43 (25.0) | 95 (26.6) |
| Pharyngitis | 0.234 | 9 (25.0) | 21 (14.1) | 24 (14.0) | 54 (15.1) |
| Upper respiratory infection | 0.625 | 3 (8.3) | 20 (13.4) | 26 (15.1) | 49 (13.7) |
| Sinusitis | 0.532 | 4 (11.1) | 22 (14.8) | 18 (10.5) | 44 (12.3) |
| Pulmonary physical finding | 0.359 | 1 (2.8) | 13 (8.7) | 18 (10.5) | 32 (9.0) |
| Sputum increased | 0.014* | 1 (2.8) | 6 (4.0) | 21 (12.2) | 28 (7.8) |
| a Overall *P*-value: Fisher's exact test *P*-value (2-tail) for comparison across asthma severity groups. Statistical significance at the 0.05, 0.01, 0.001 levels is denoted by *, **, *** respectively. The incidence of AEs reported for all other body systems was less than 20. | | | | | |
