# Supplementary material for: Pathways Activated during Human Asthma Exacerbation as Revealed by Gene Expression Patterns in Blood
Source: PLoS One. 2011 Jul 14;6(7):e21902. doi: 10.1371/journal.pone.0021902 (PMC3136489; doi:10.1371/journal.pone.0021902)
Supplement: Table S17 — Mean FEV1 (% predicted) at scheduled non-exacerbation visits. (DOC) [file pone.0021902.s024.doc]

| Online Supporting Information Table S17: Mean FEV1 (% Predicted) at Scheduled Non-Exacerbation Visits | | | | |
| --- | --- | --- | --- | --- |
| Exacerbation Status | Statistic | Asthma Severity | | |
|  |  | Mild | Moderate | Severe |
| Never | n | 81 | 295 | 300 |
|  | **Mean** | **95.01** | **85.51** | **74.85** |
|  | *P*-value^a^ | 0.0000 |  |  |
| At Least 1 | n | 33 | 181 | 244 |
|  | **Mean** | **90.27** | **86.33** | **74.15** |
|  | *P*-value^a^ | 0.0000 |  |  |
|  | *P*-value^b^ | 0.0479 | 0.5675 | 0.6851 |
| All Subjects | n | 114 | 476 | 544 |
|  | **Mean** | **93.64** | **85.83** | **74.54** |
|  | *P*-value^a^ | 0.0000 |  |  |
| a *P*-value indicates test for differences among asthma severity groups  b *P*-value indicates test for difference between exacerbation status groups (never had an exacerbation versus had at least 1 exacerbation) within an asthma severity category  Abbreviations: FEV_1_ = forced expiratory volume in 1 second | | | | |
