# Supplementary material for: Pathways Activated during Human Asthma Exacerbation as Revealed by Gene Expression Patterns in Blood
Source: PLoS One. 2011 Jul 14;6(7):e21902. doi: 10.1371/journal.pone.0021902 (PMC3136489; doi:10.1371/journal.pone.0021902)

## Online Supporting Information Table S18A: ANCOVA Results Subgroup X
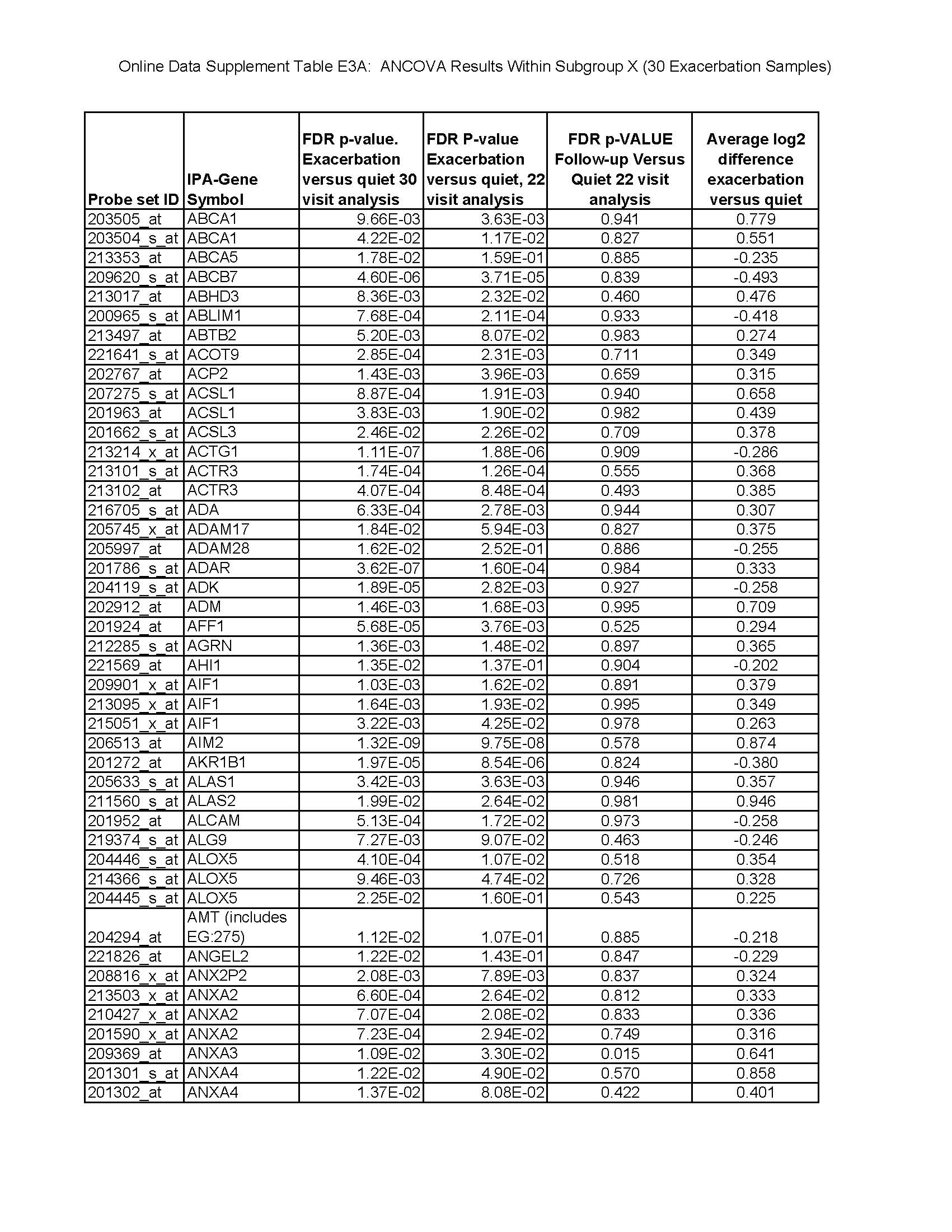


Table S18A: ANCOVA Results Subgroup X, continued
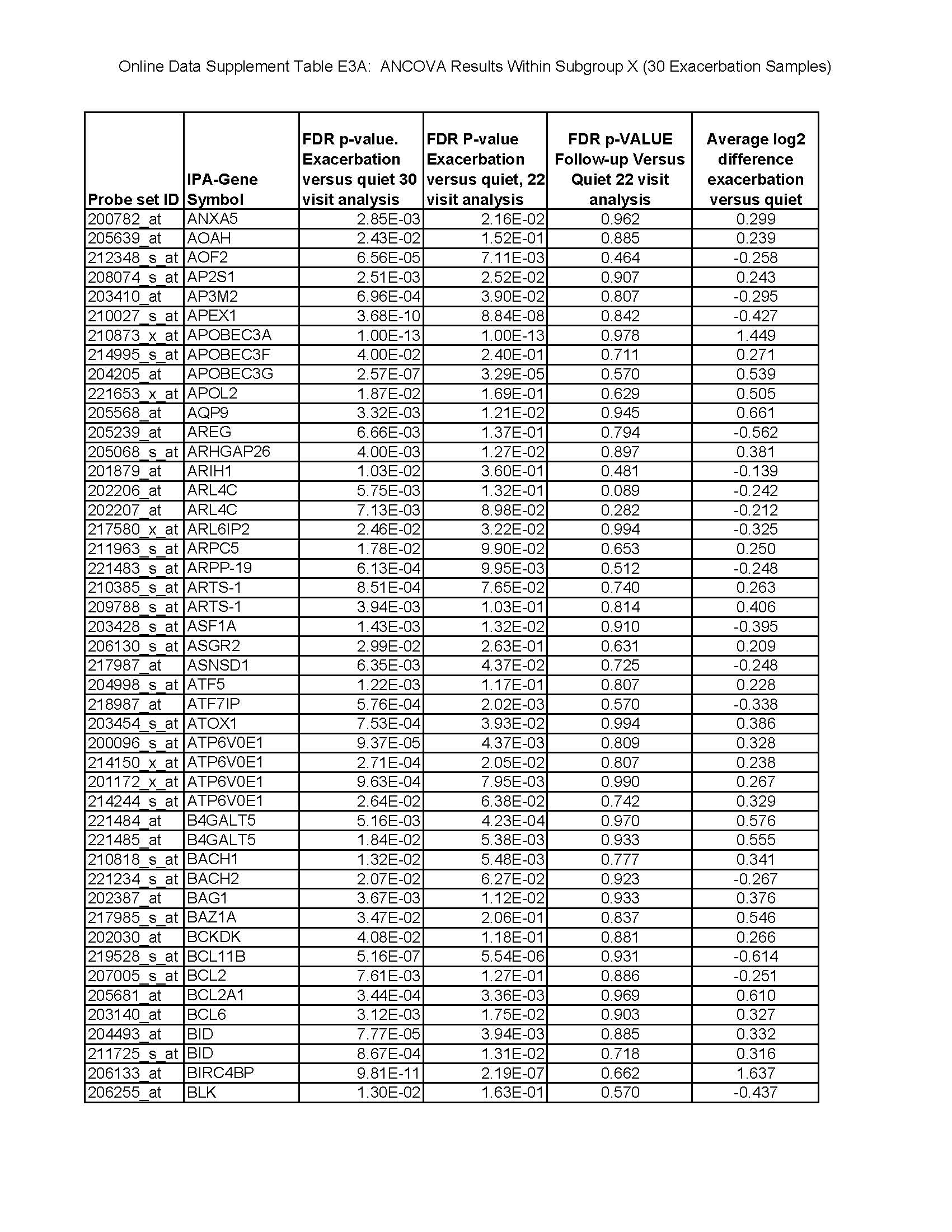


Table S18A: ANCOVA Results Subgroup X, continued
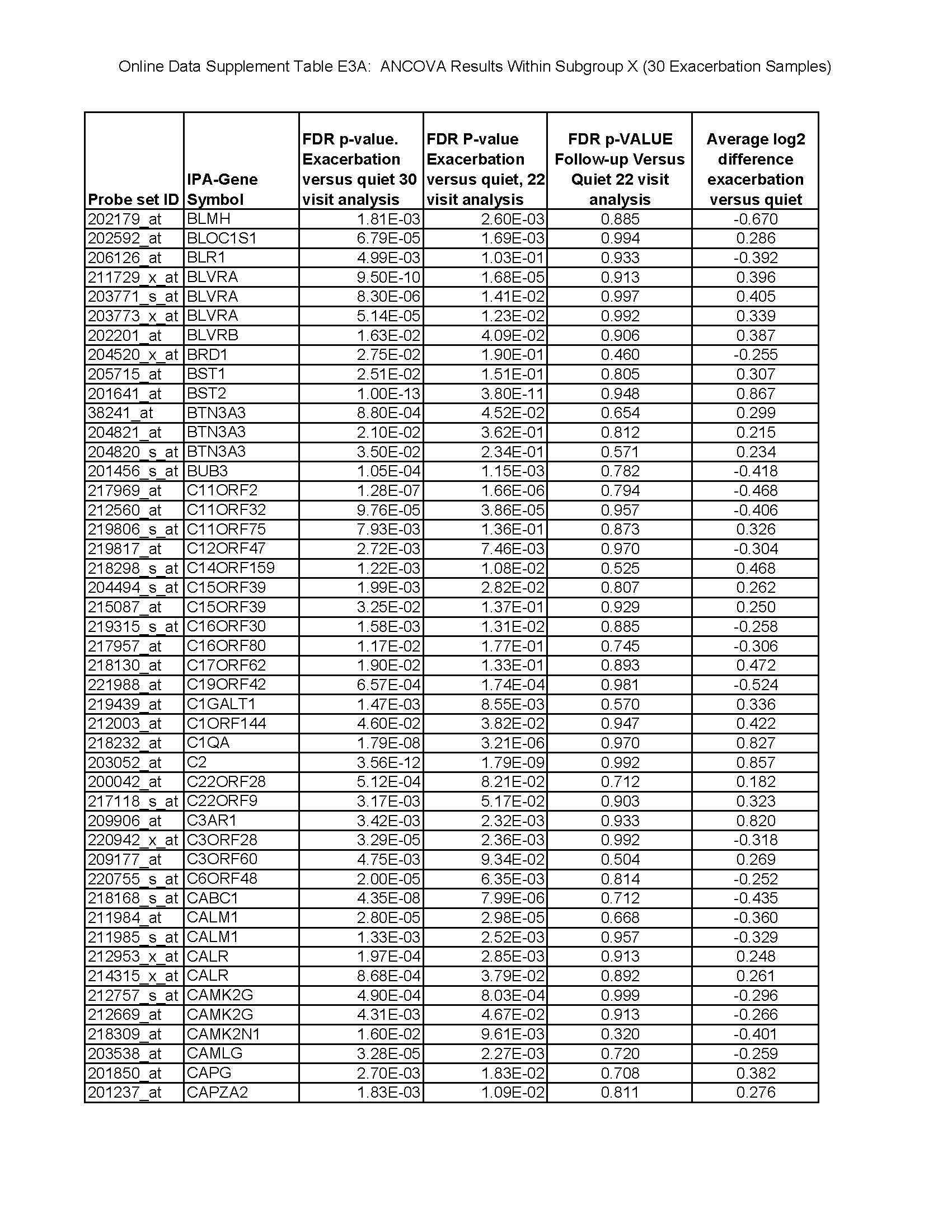


Table S18A: ANCOVA Results Subgroup X continued \
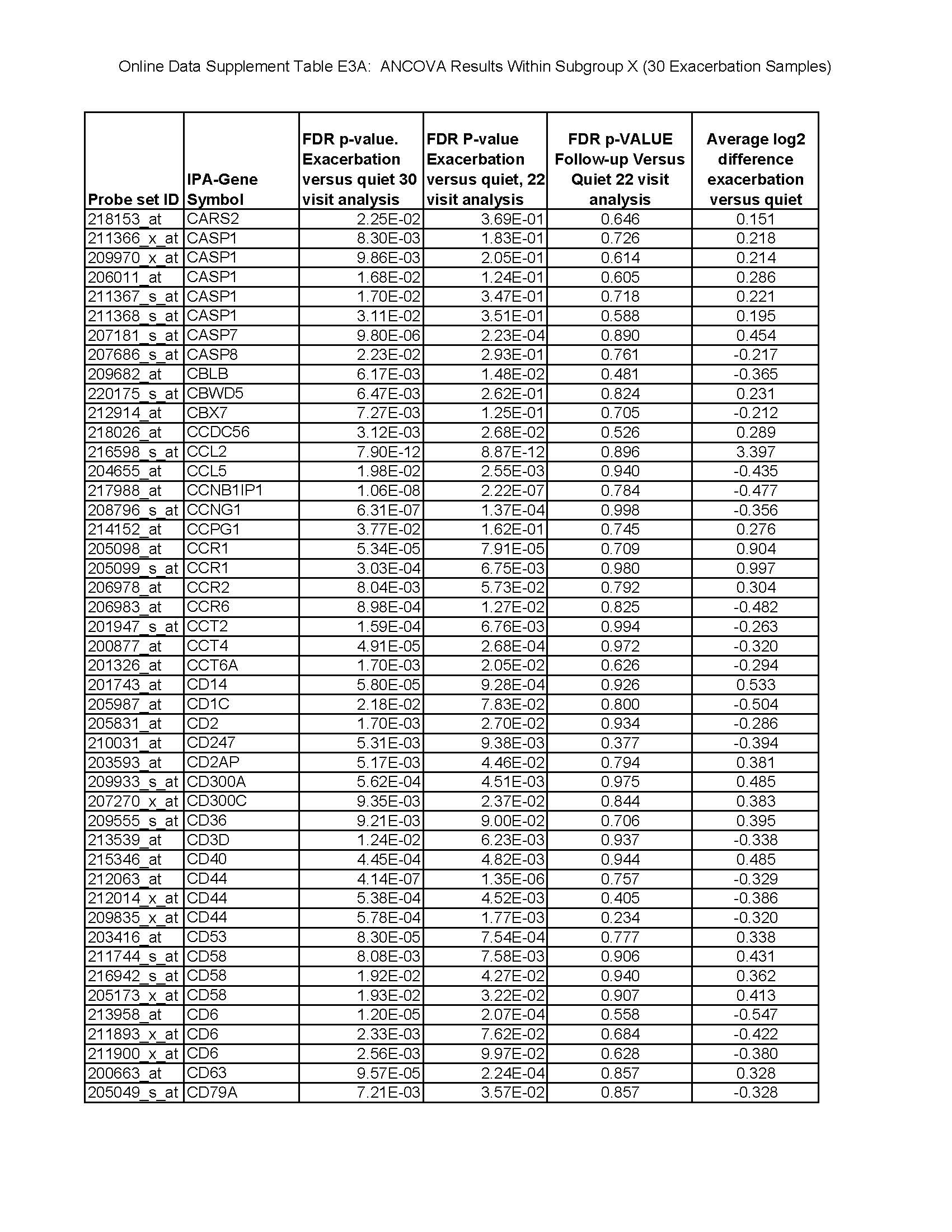


Table S18A: ANCOVA Results Subgroup X continued
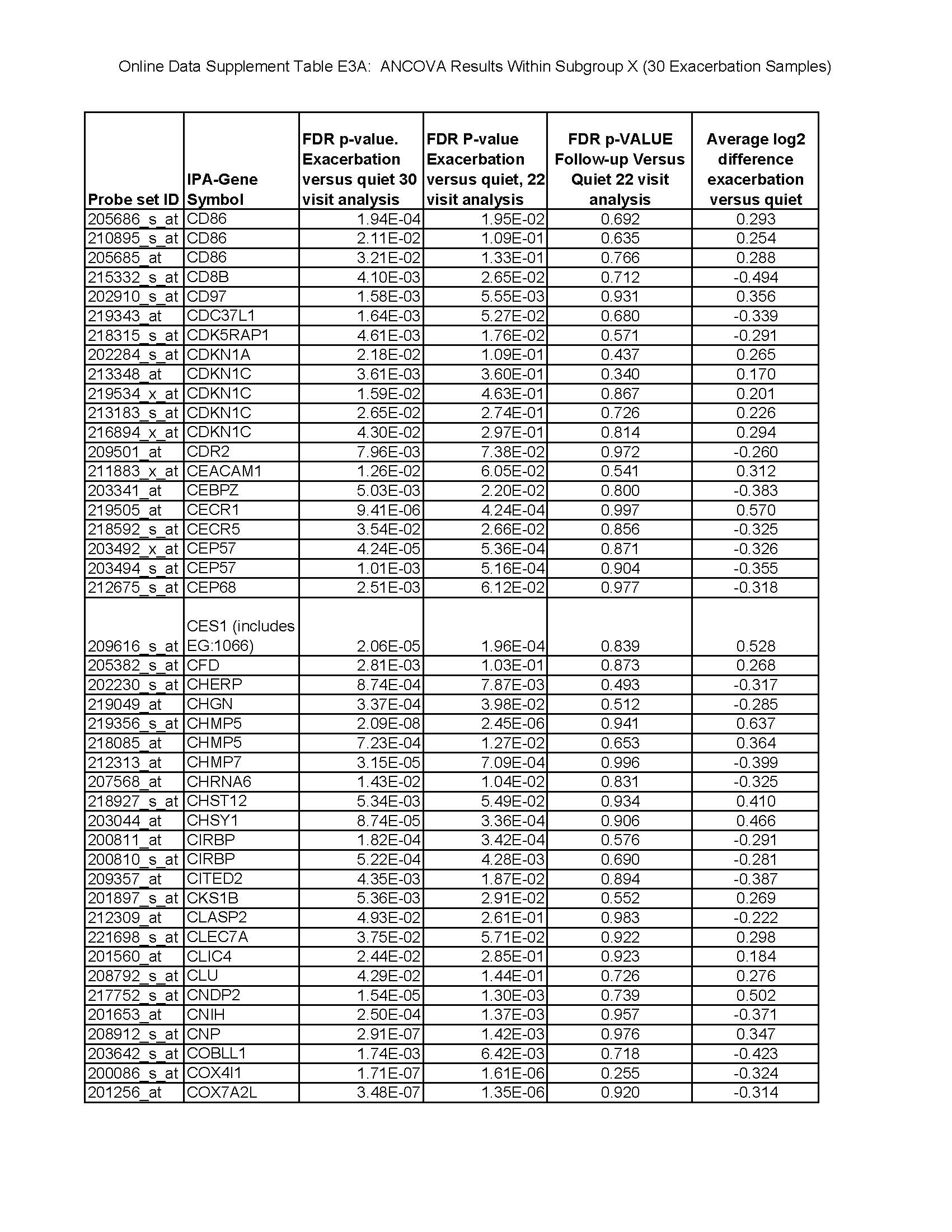


Table S18A: ANCOVA Results Subgroup X continued
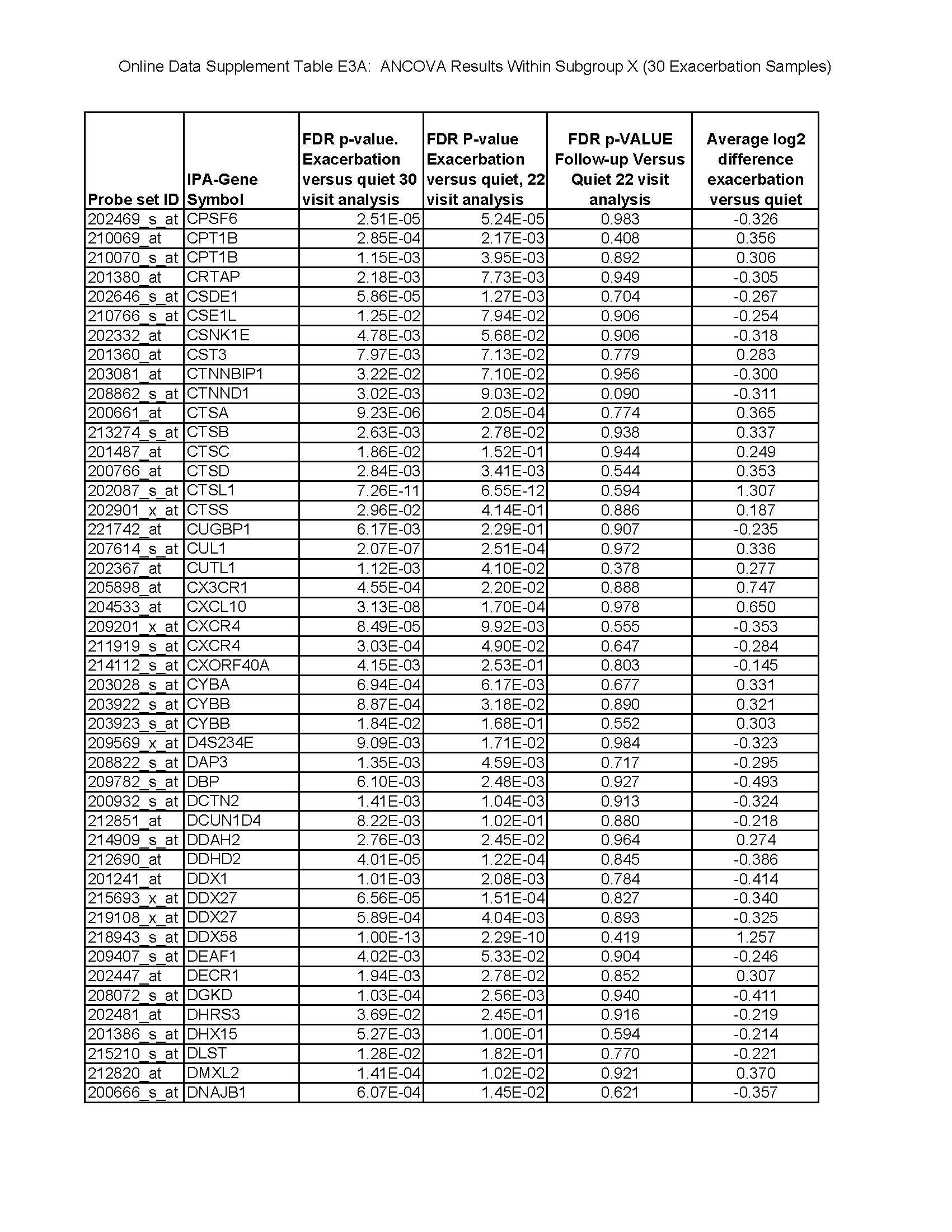


Table S18A: ANCOVA Results Subgroup X continued
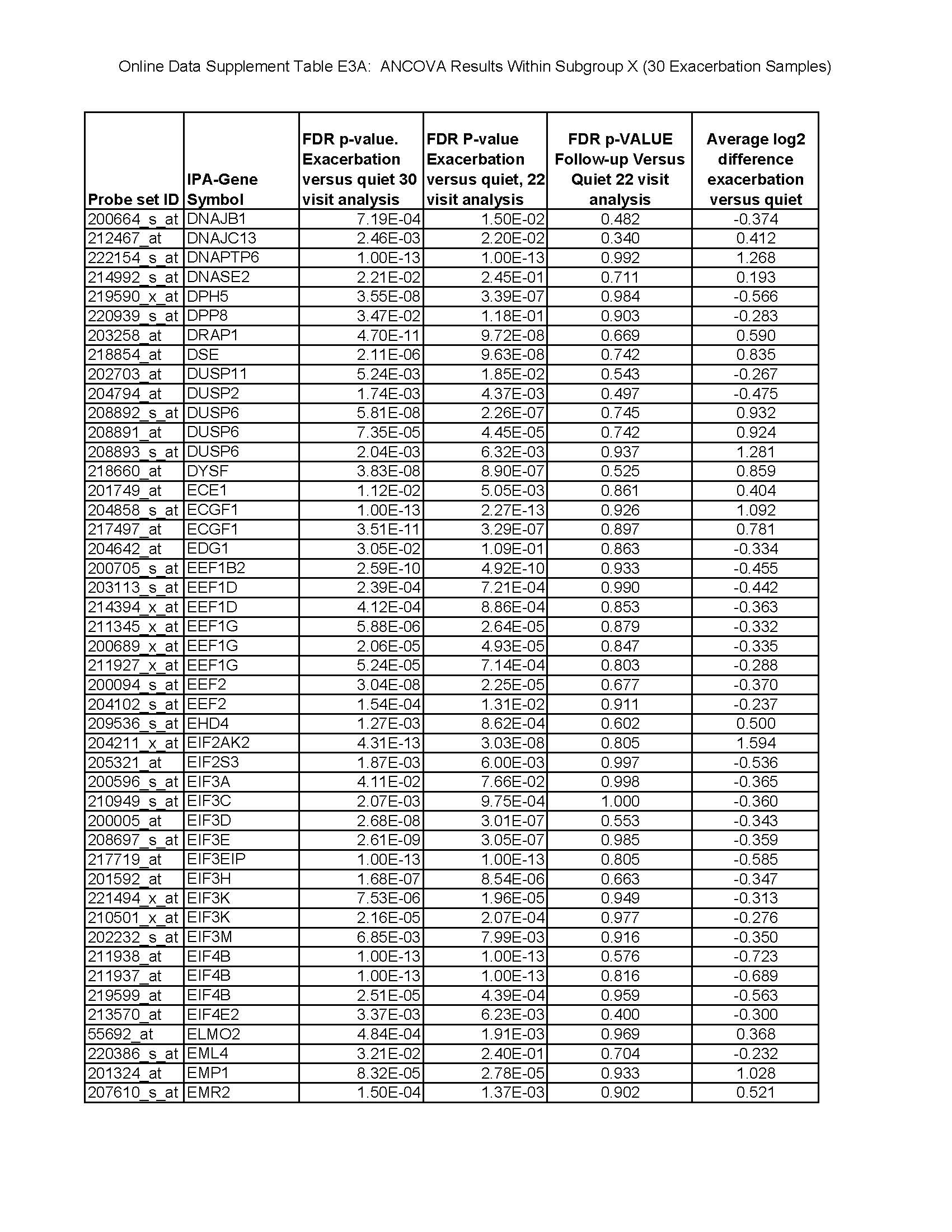


Table S18A: ANCOVA Results Subgroup X continued
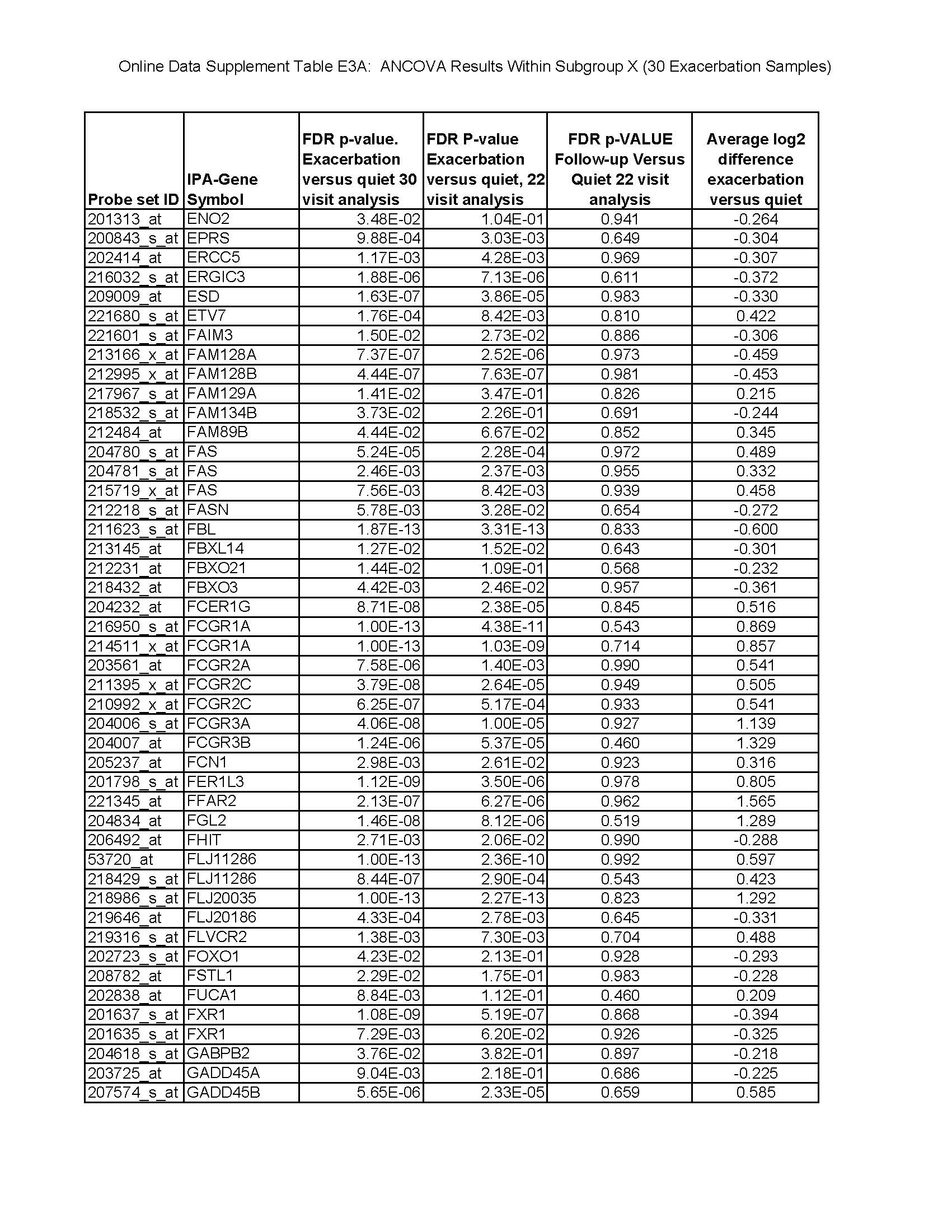


Table S18A: ANCOVA Results Subgroup X continued
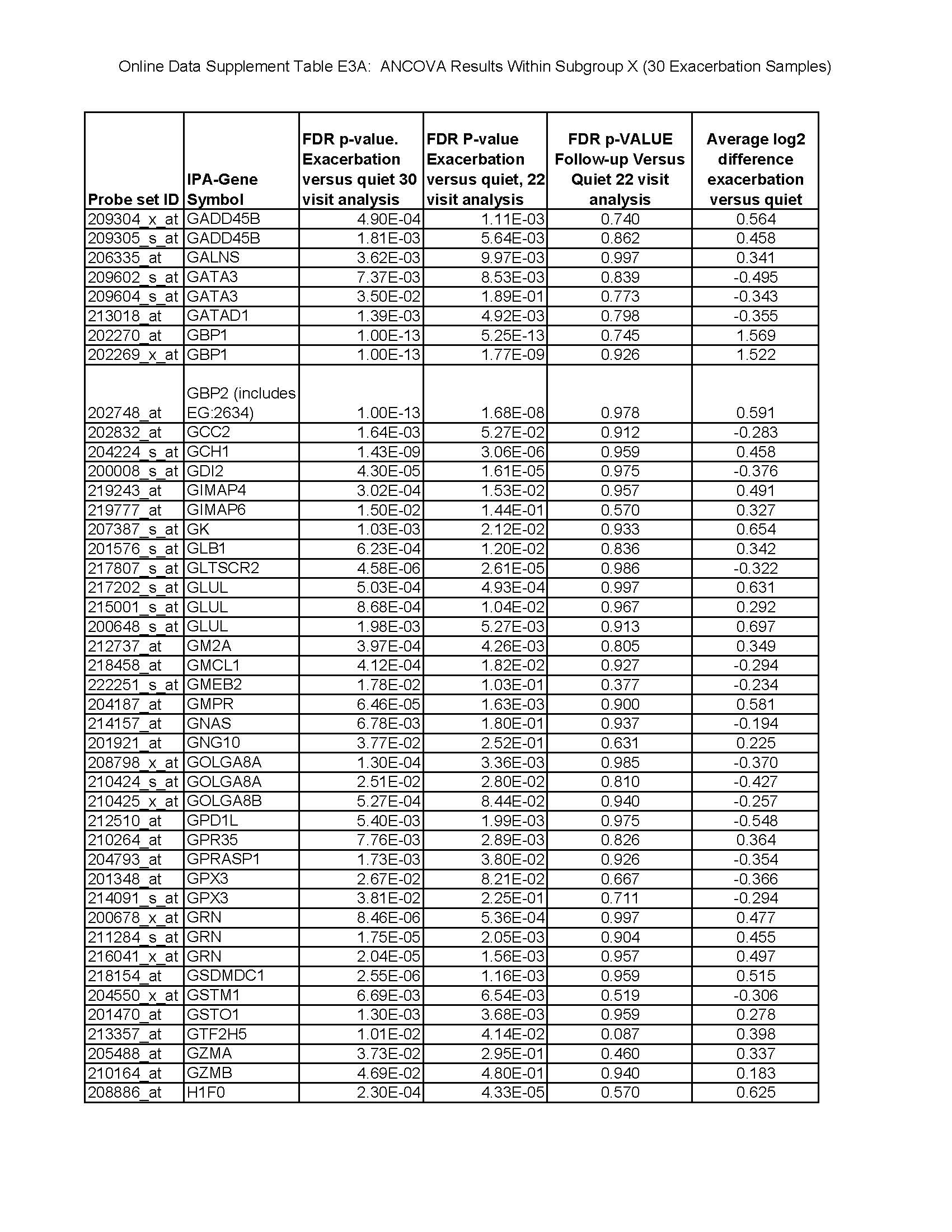


Table S18A: ANCOVA Results Subgroup X continued
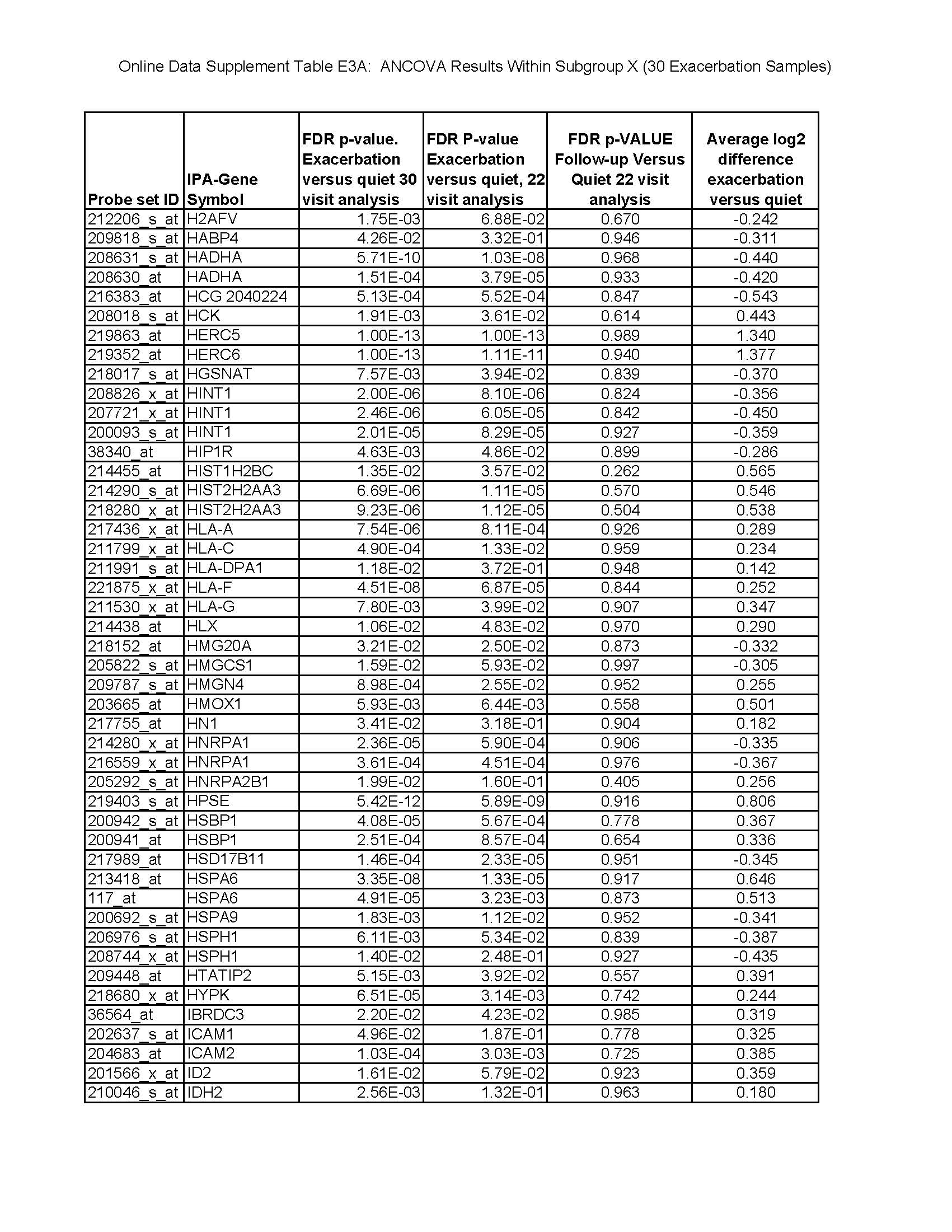


Table S18A: ANCOVA Results Subgroup X continued
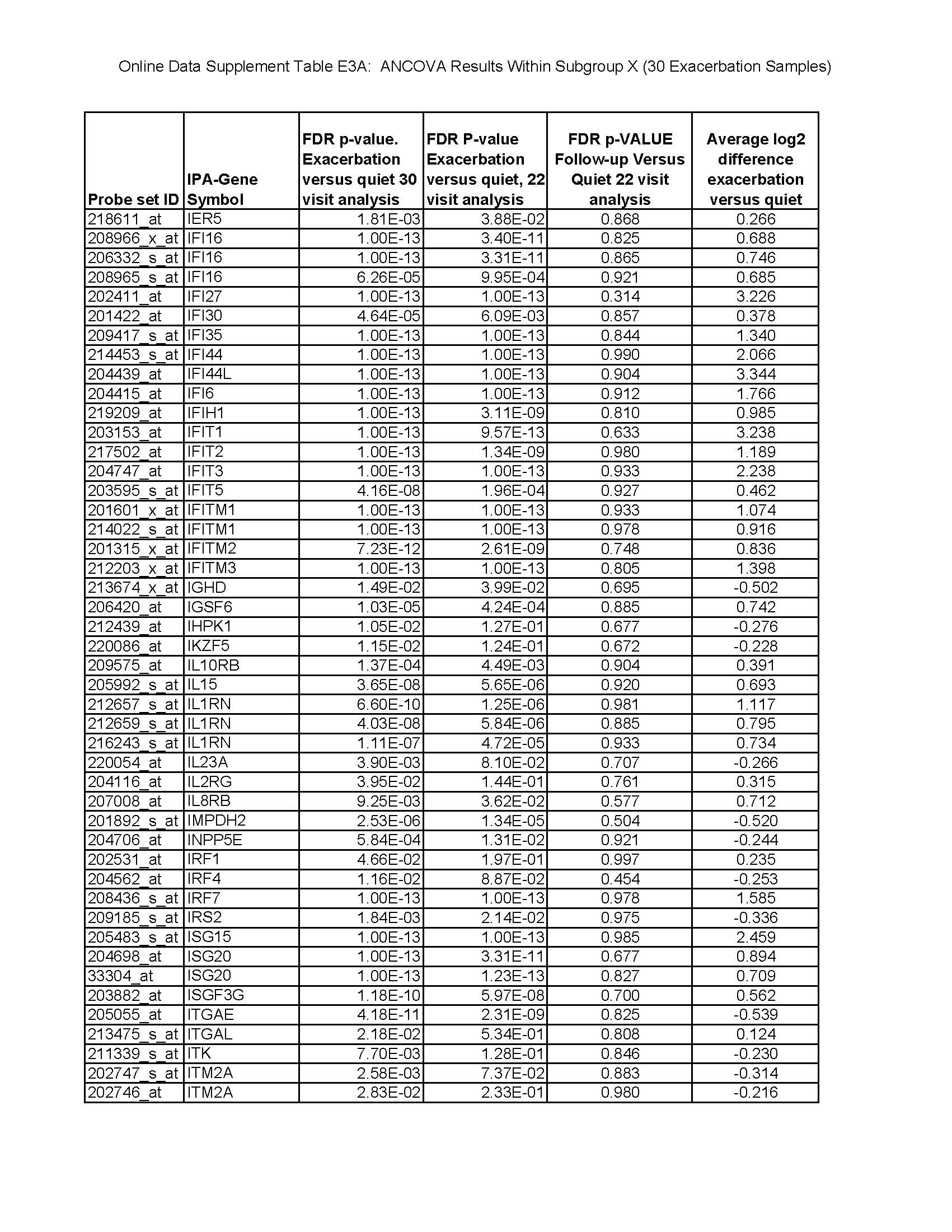


Table S18A: ANCOVA Results Subgroup X continued
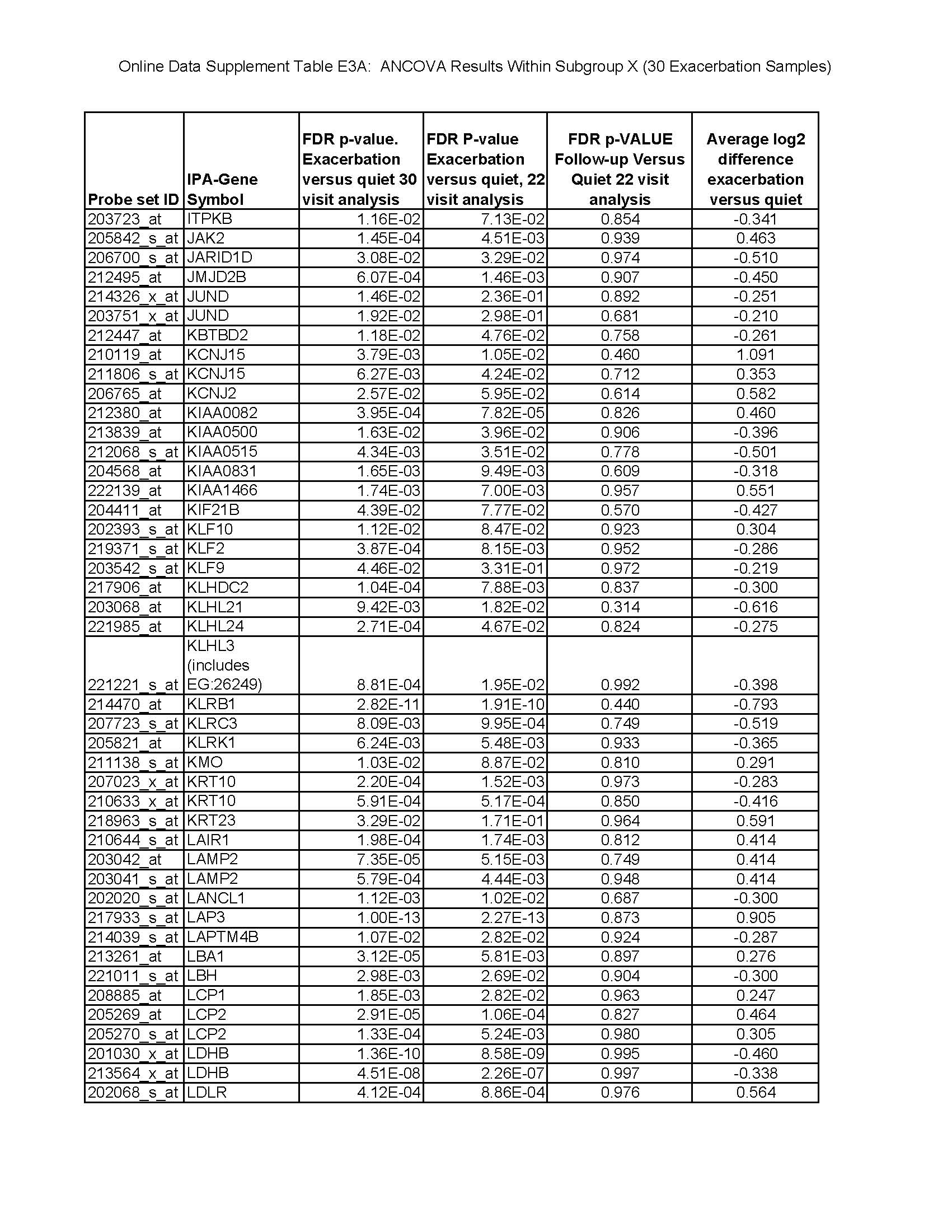


Table S18A: ANCOVA Results Subgroup X continued
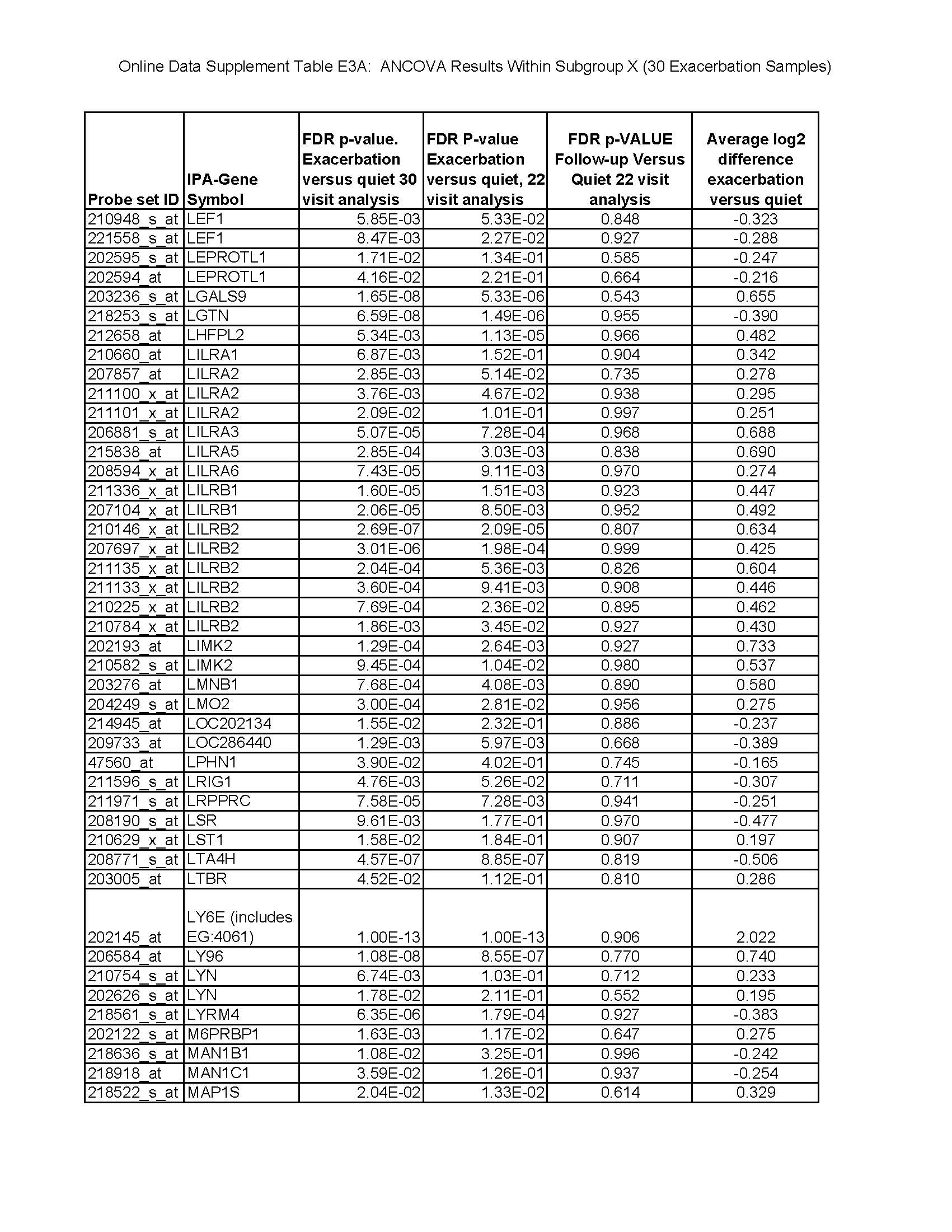


Table S18A: ANCOVA Results Subgroup X continued
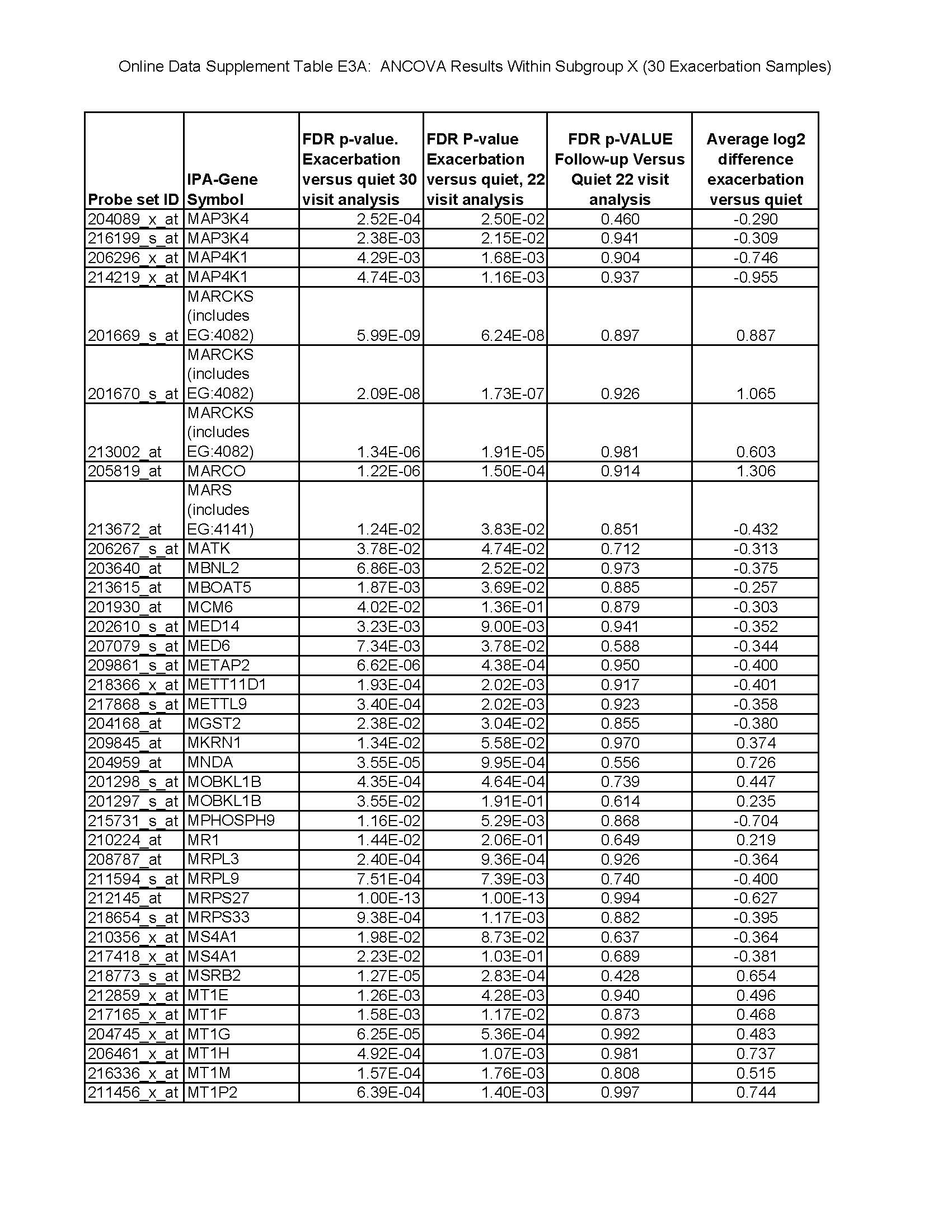
 Table S18A: ANCOVA Results Subgroup X continued
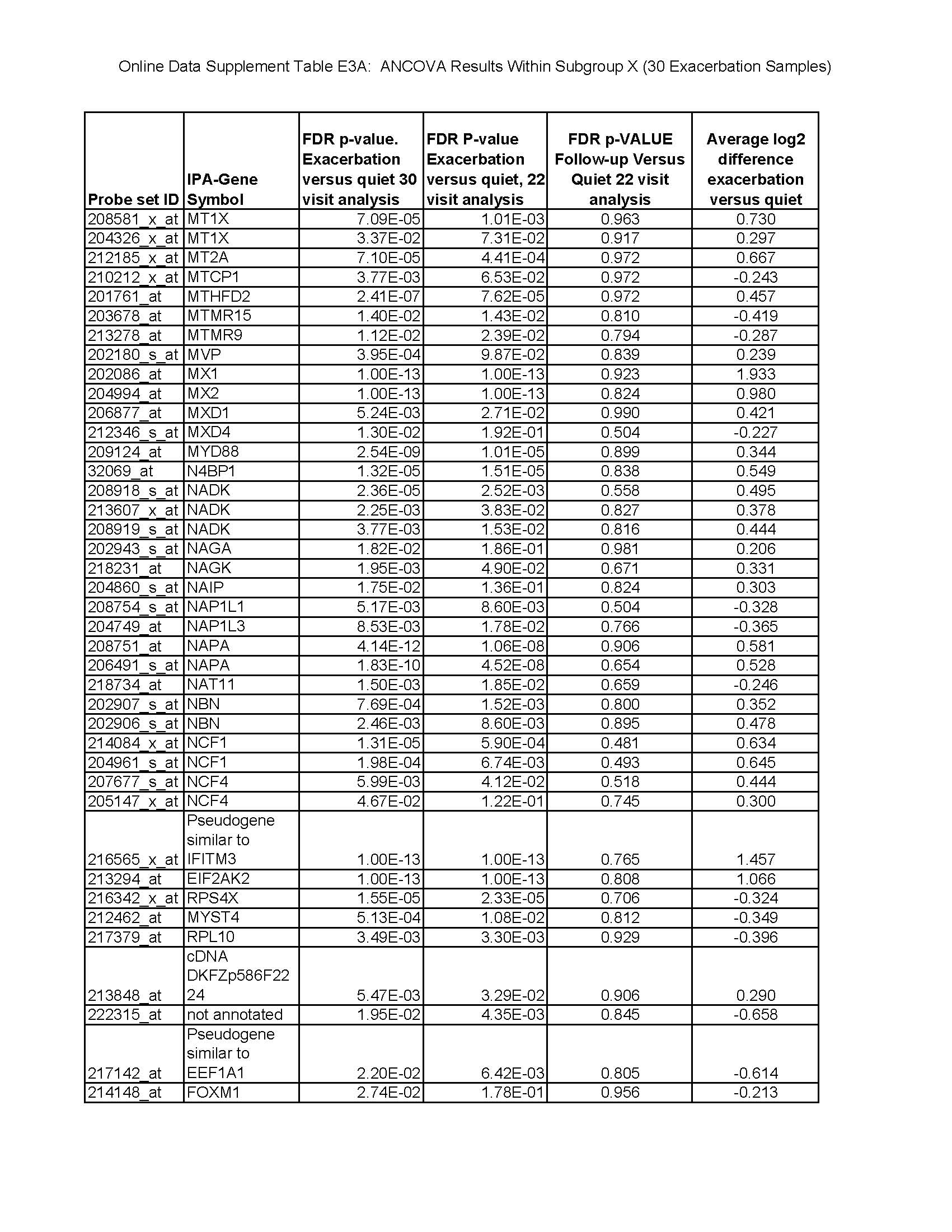


Table S18A: ANCOVA Results Subgroup X continued
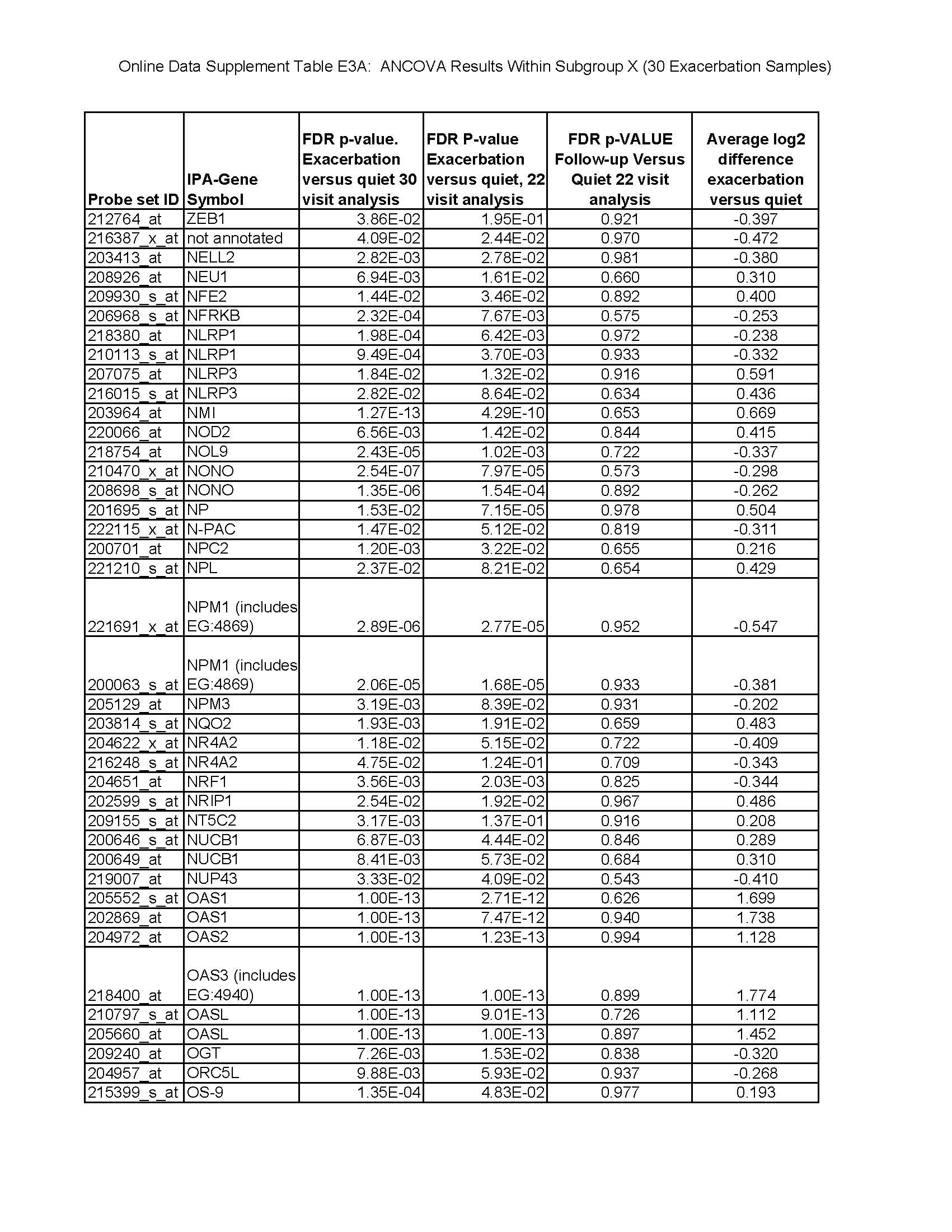


Table S18A: ANCOVA Results Subgroup X continued
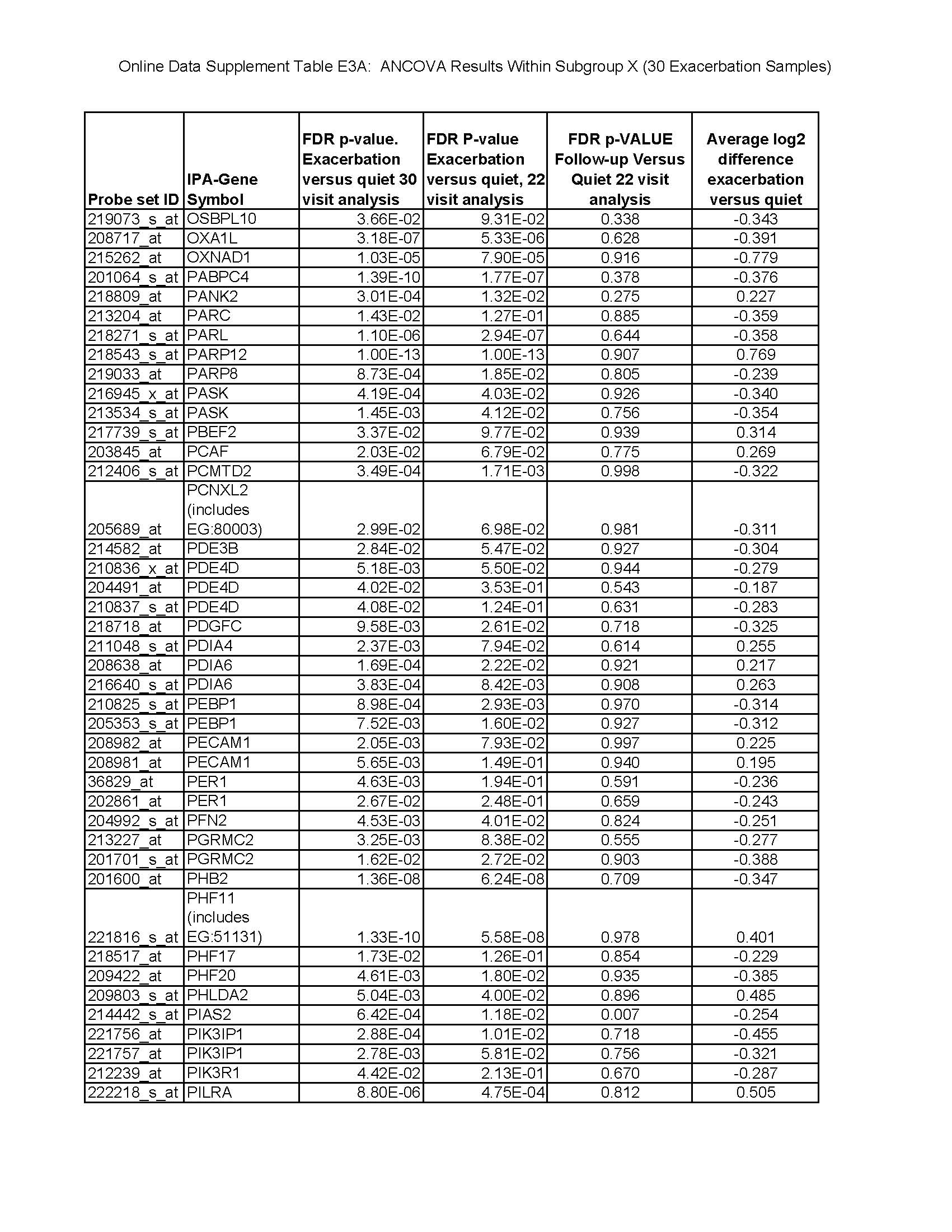


Table S18A: ANCOVA Results Subgroup X continued
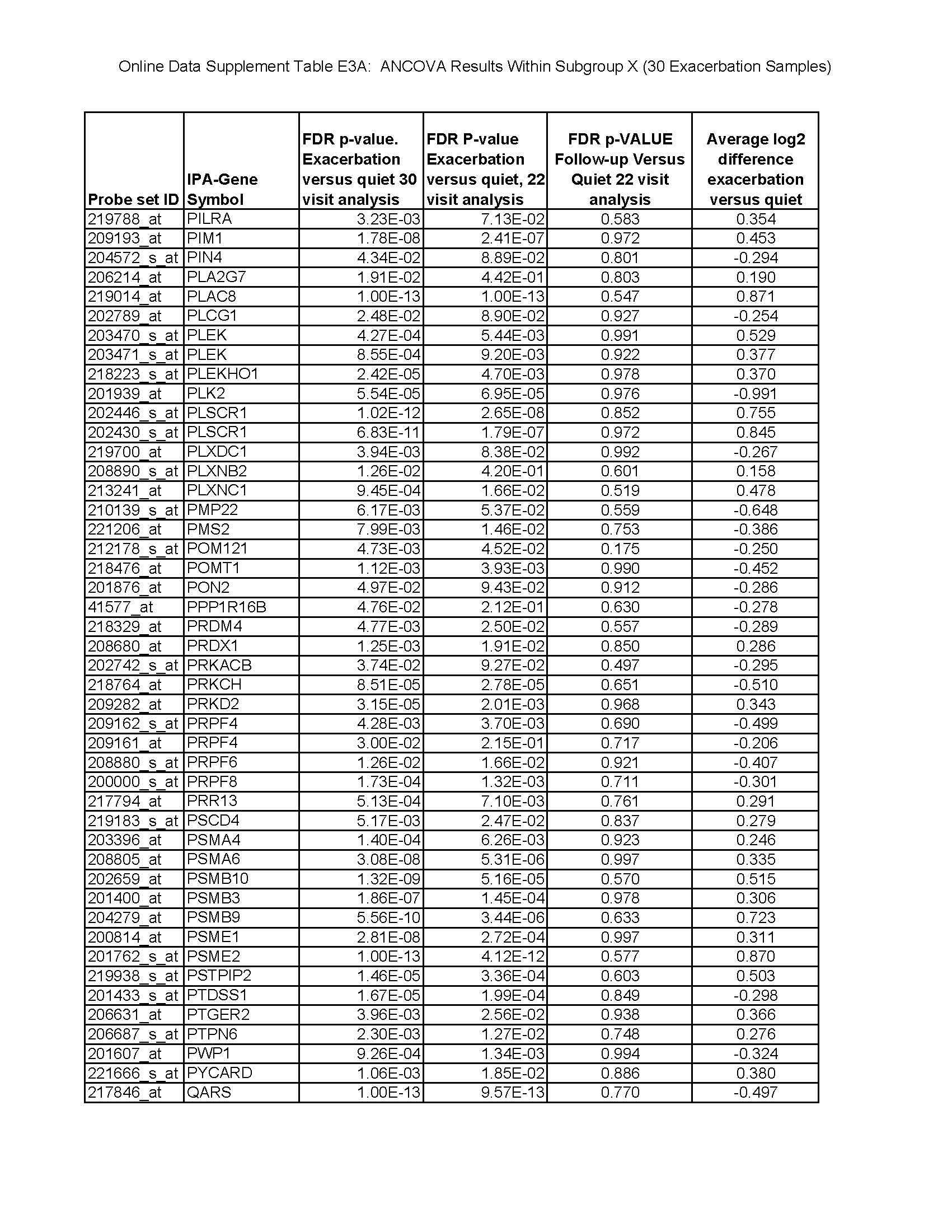


Table S18A: ANCOVA Results Subgroup X continued
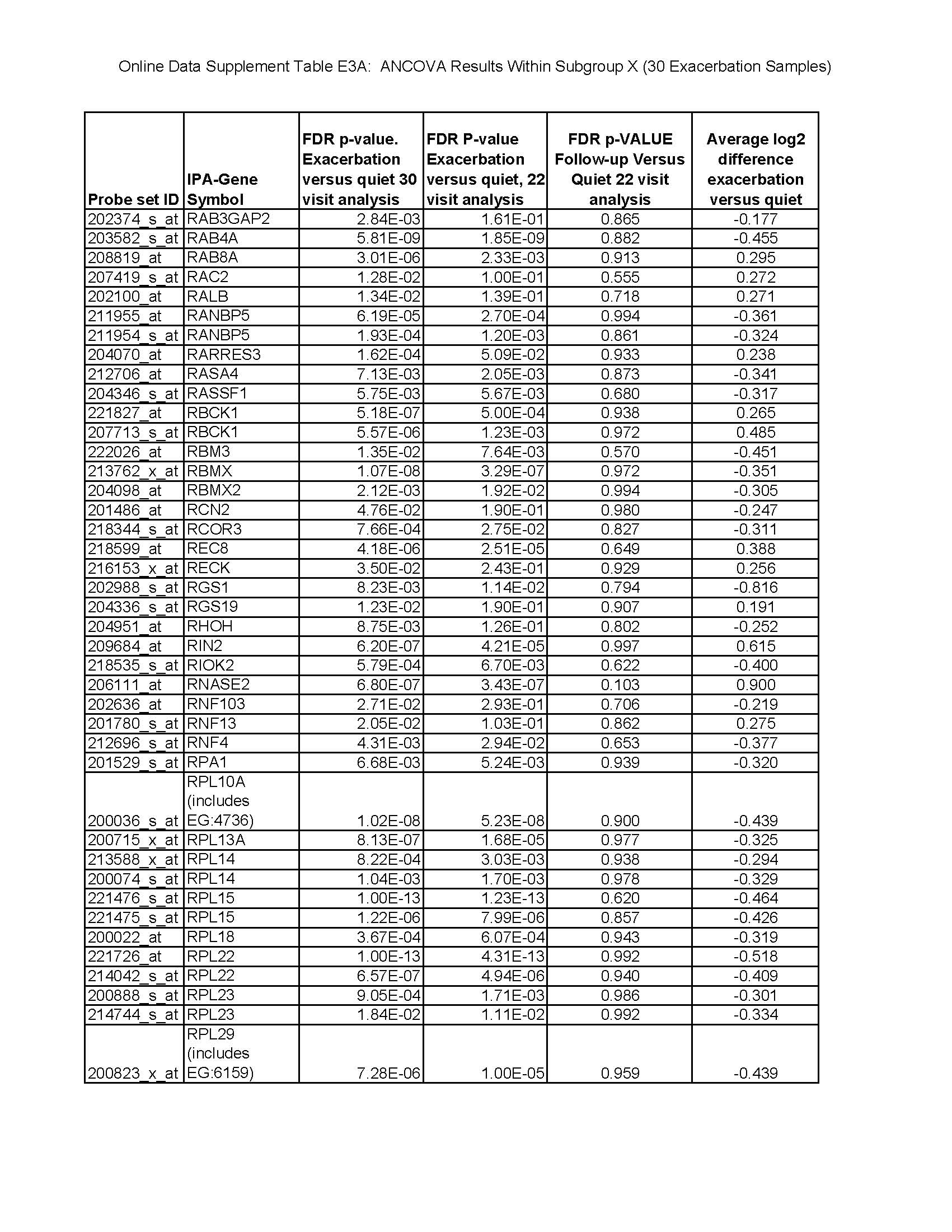


Table S18A: ANCOVA Results Subgroup X continued
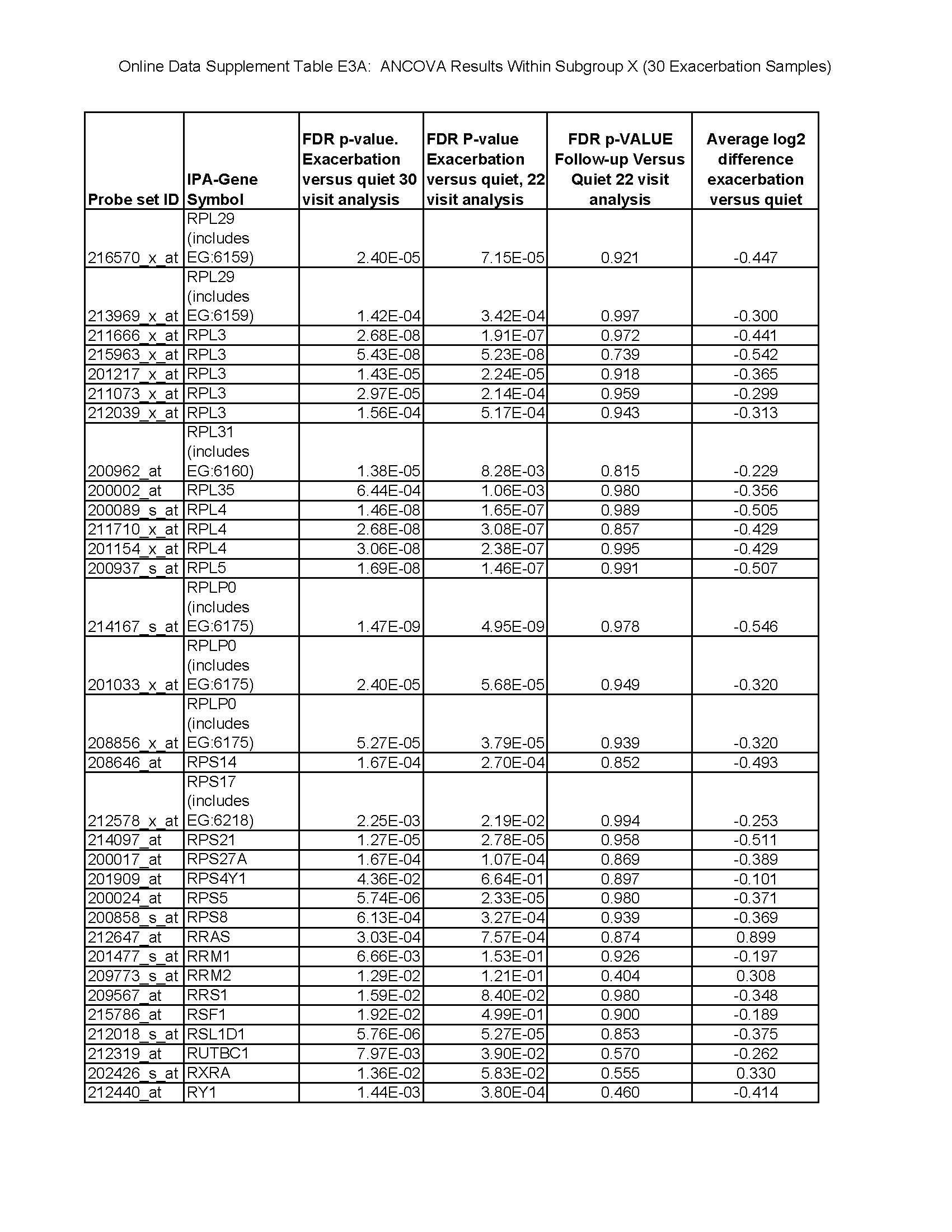


Table S18A: ANCOVA Results Subgroup X continued
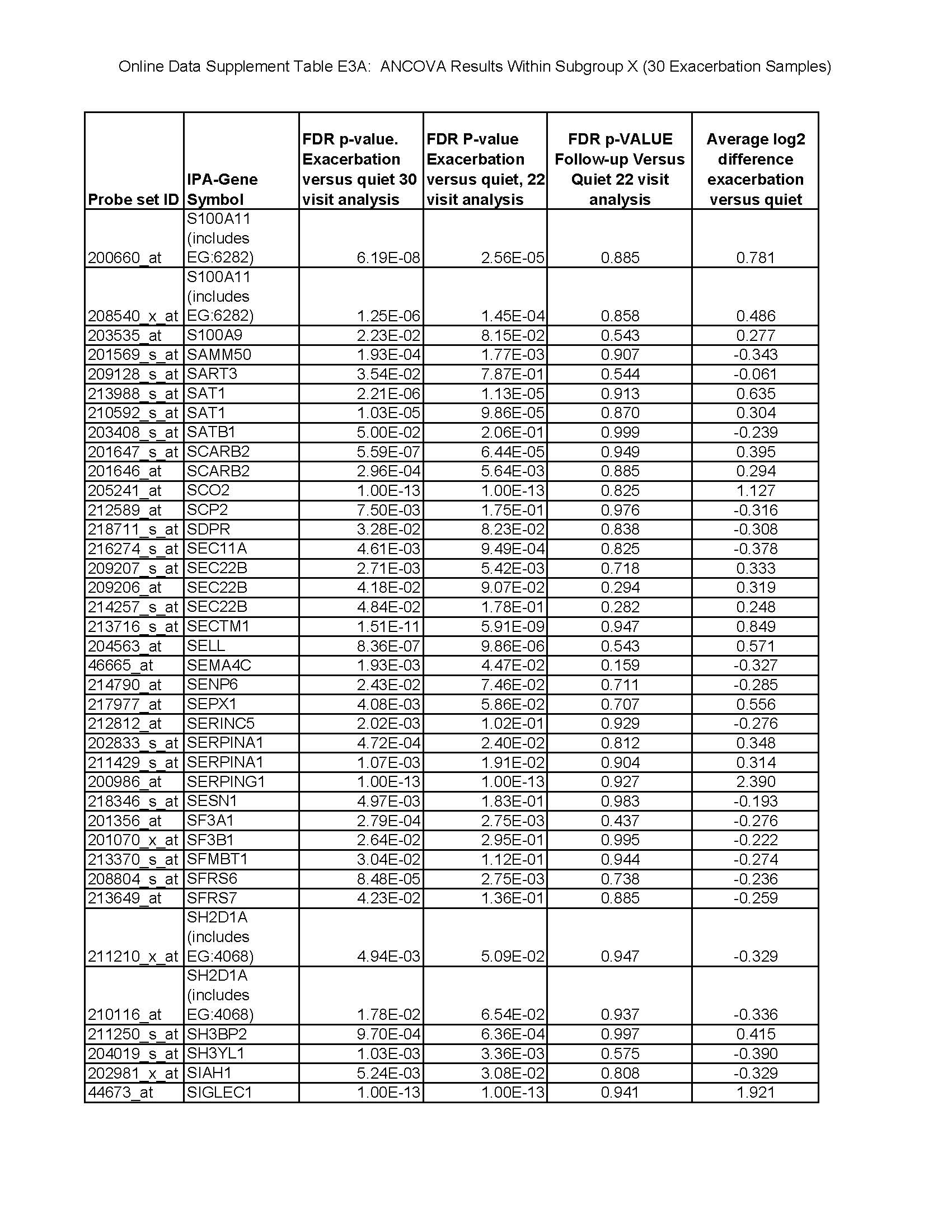


Table S18A: ANCOVA Results Subgroup X continued
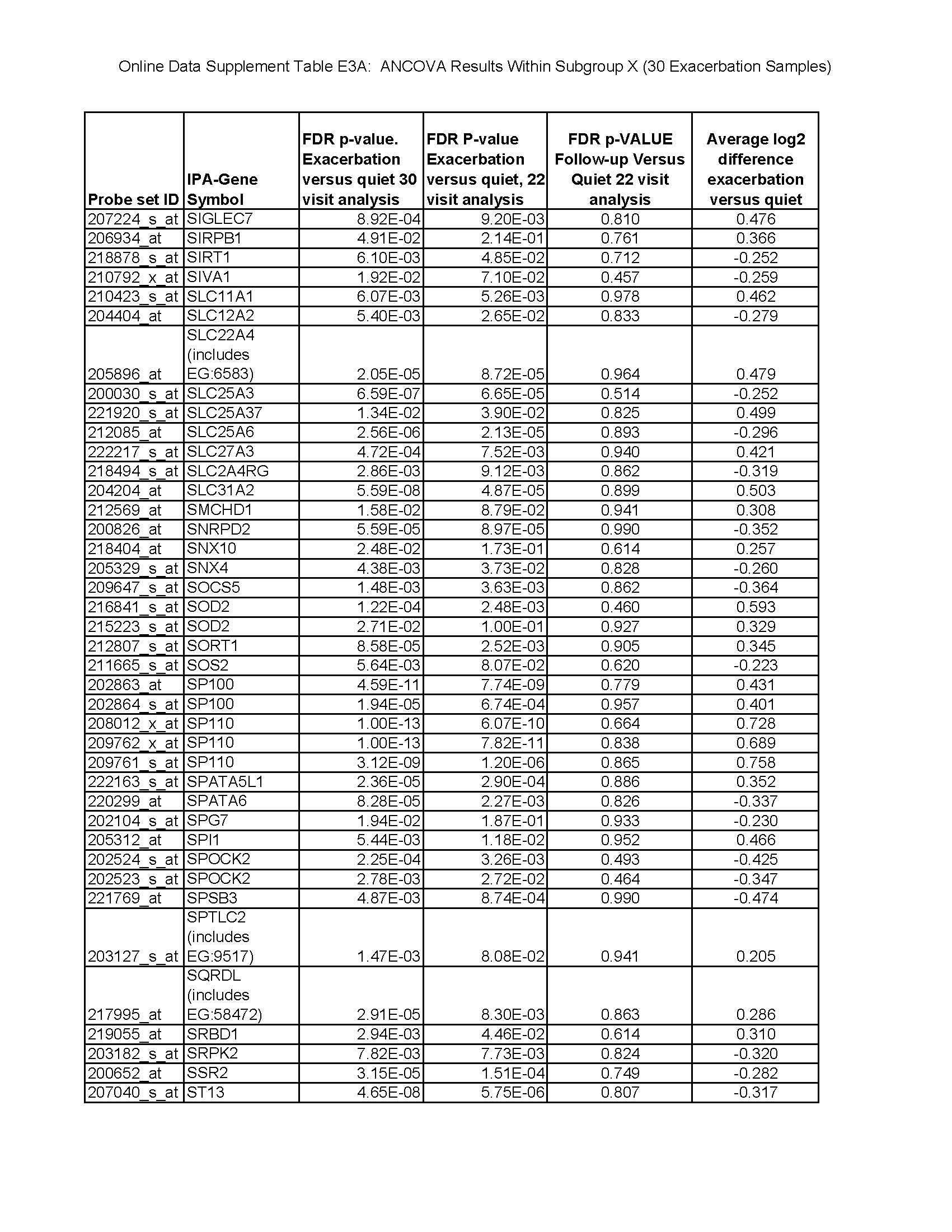


Table S18A: ANCOVA Results Subgroup X continued
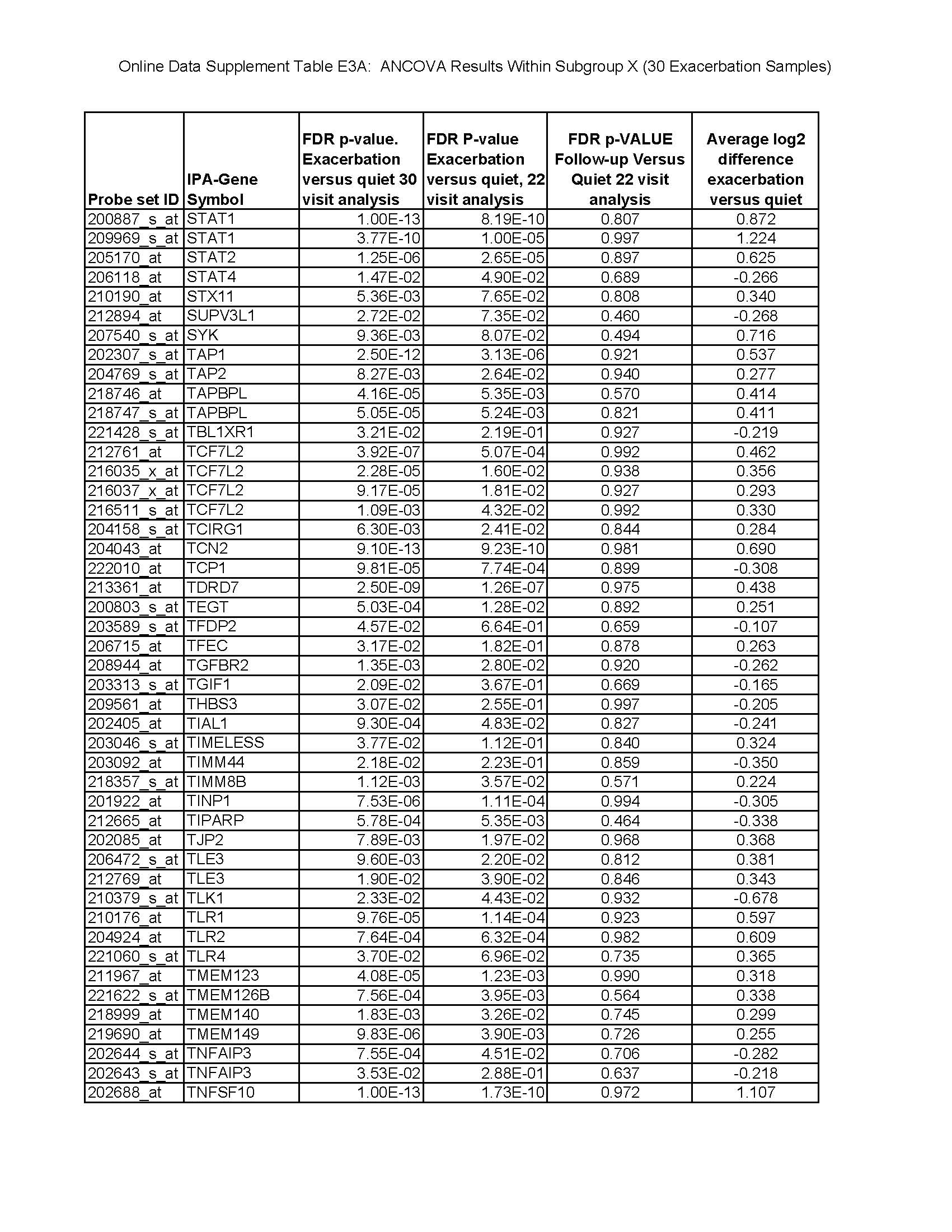


Table S18A: ANCOVA Results Subgroup X continued
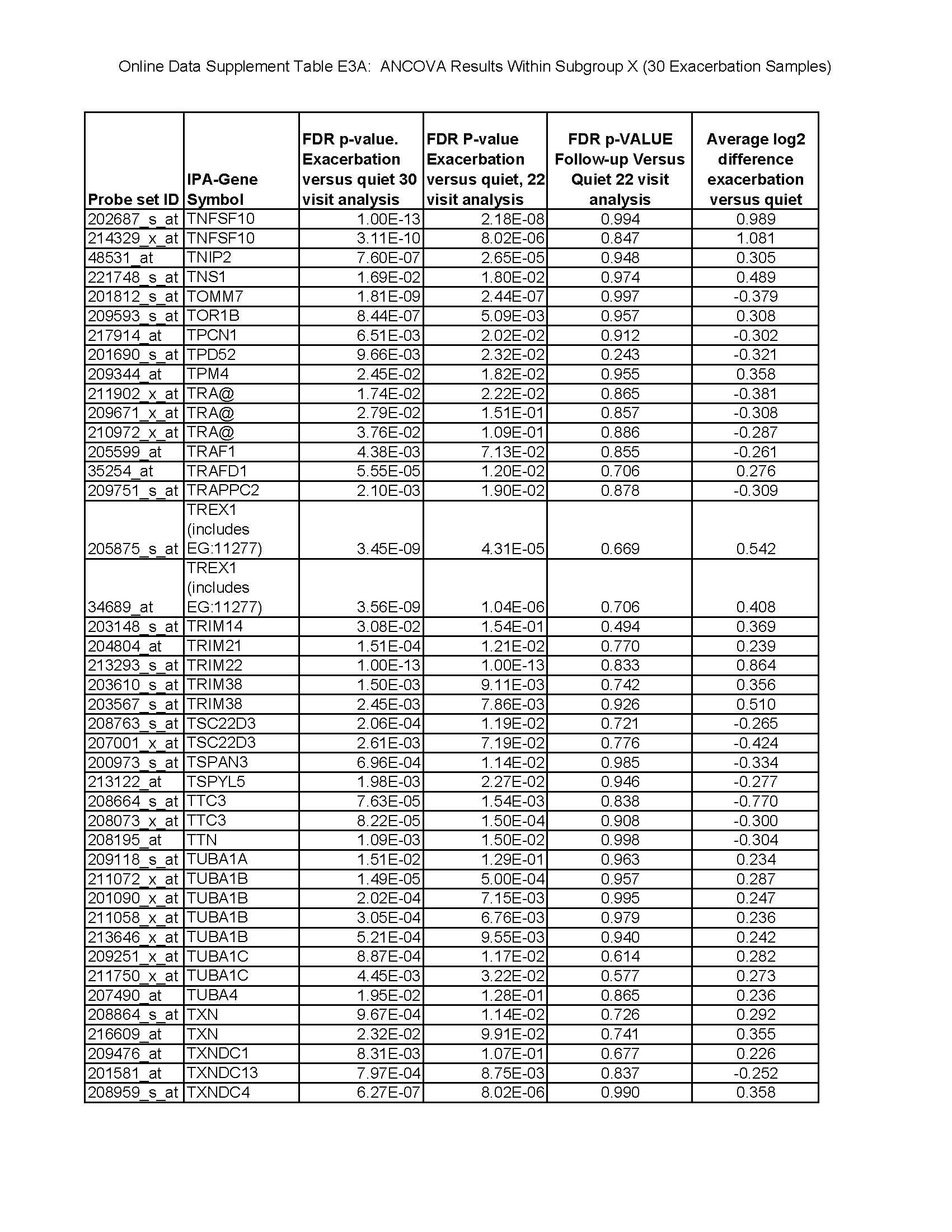


Table S18A: ANCOVA Results Subgroup X continued
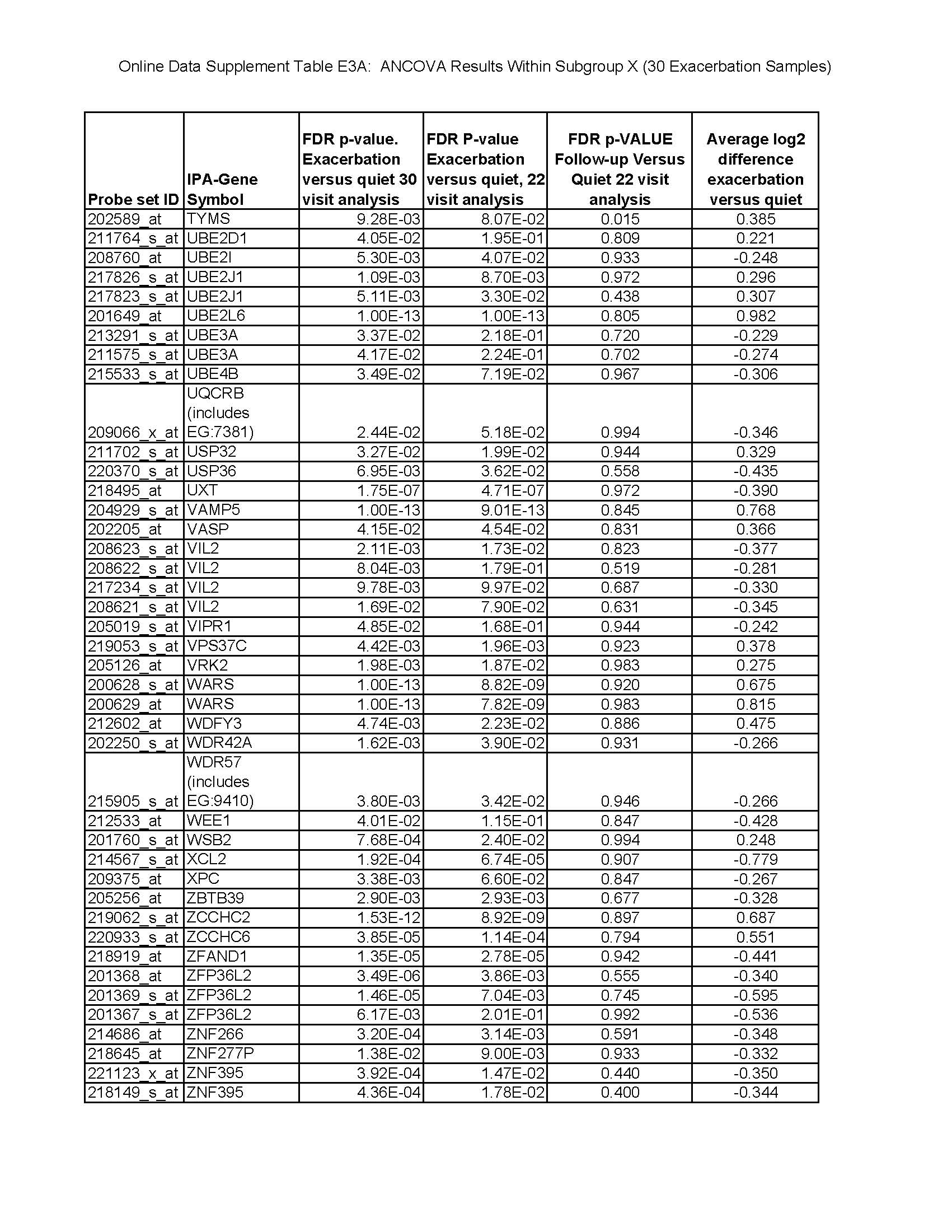

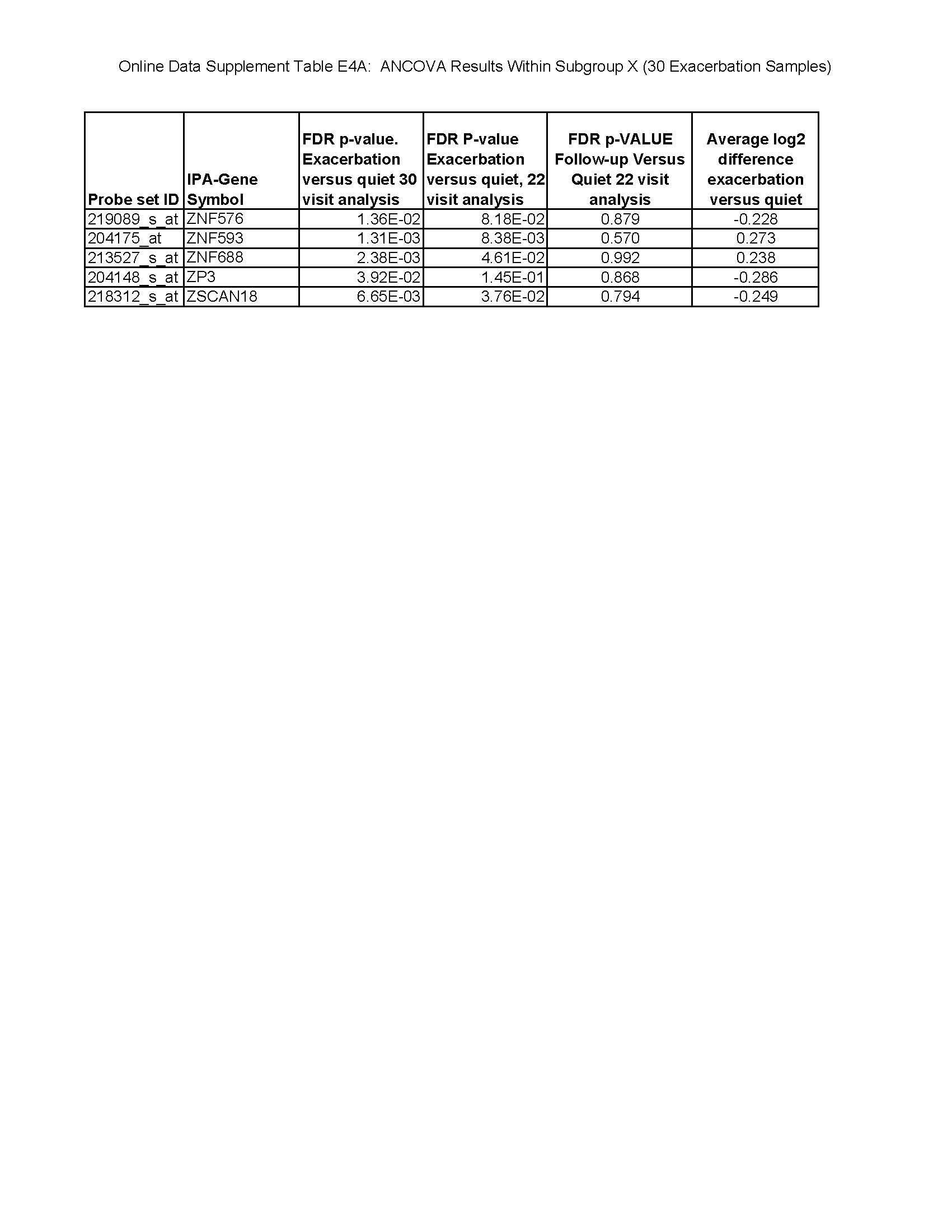


## Online Supporting Information Table S18B: ANCOVA Results Subgroup Y Samples


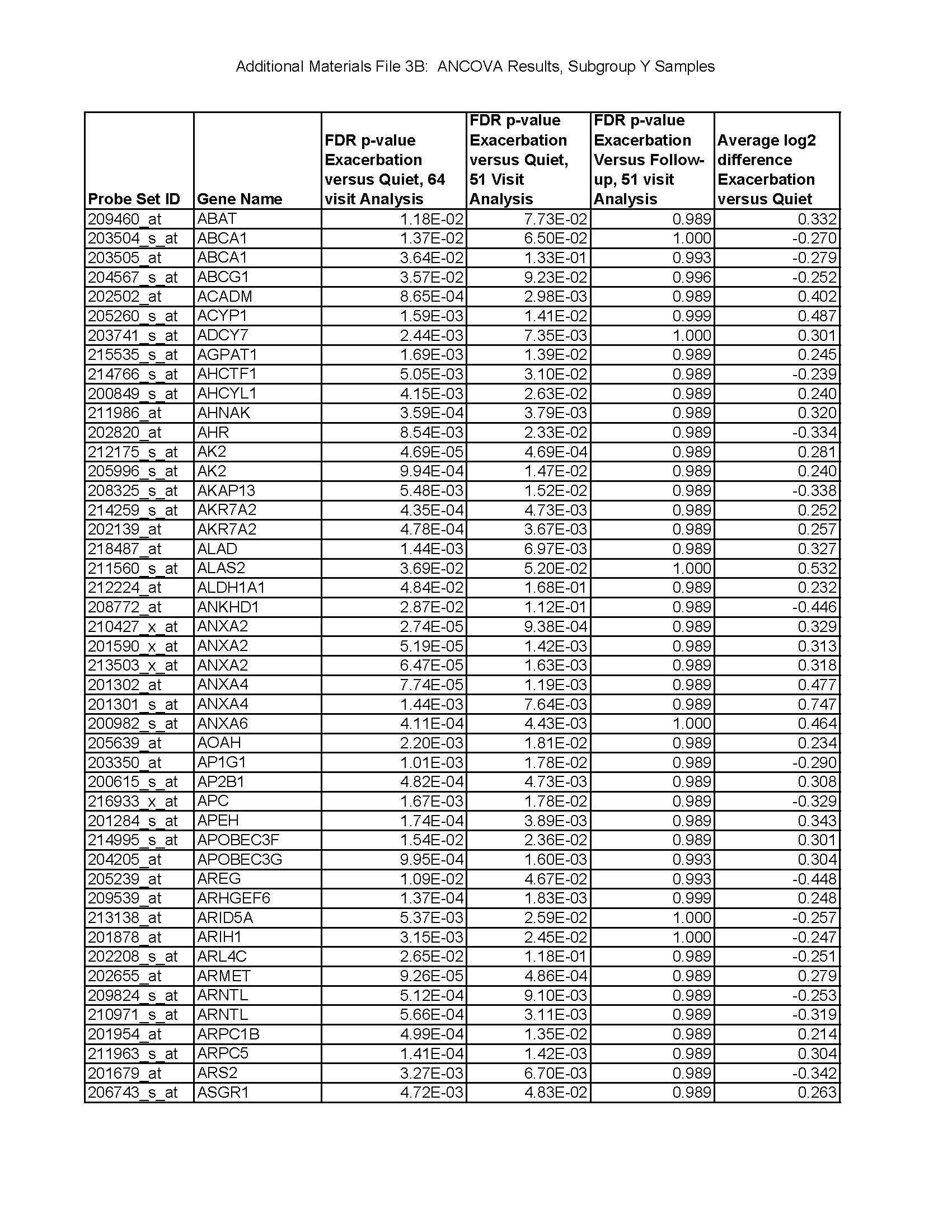


Table S18B: ANCOVA Results Subgroup Y Samples


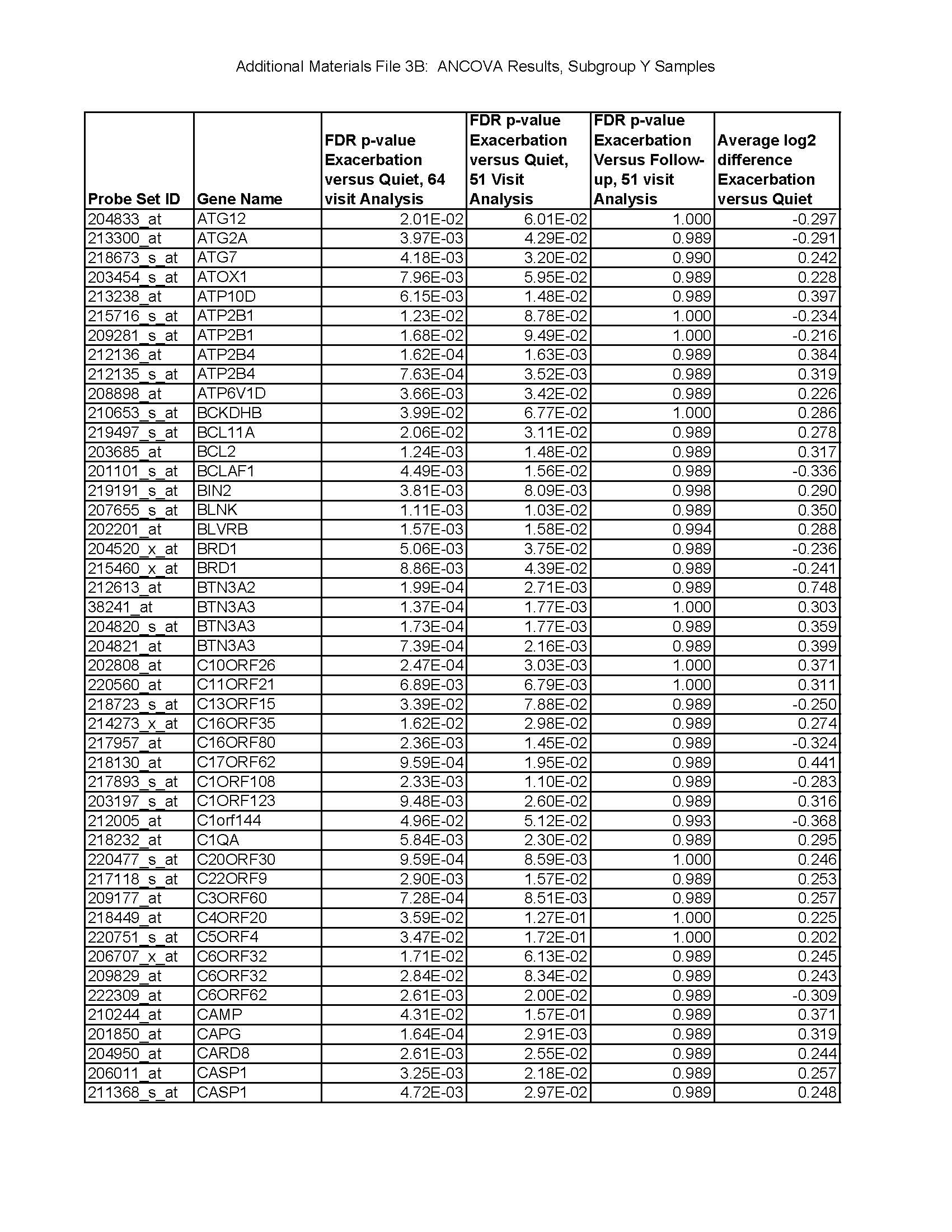


Table S18B: ANCOVA Results Subgroup Y continued
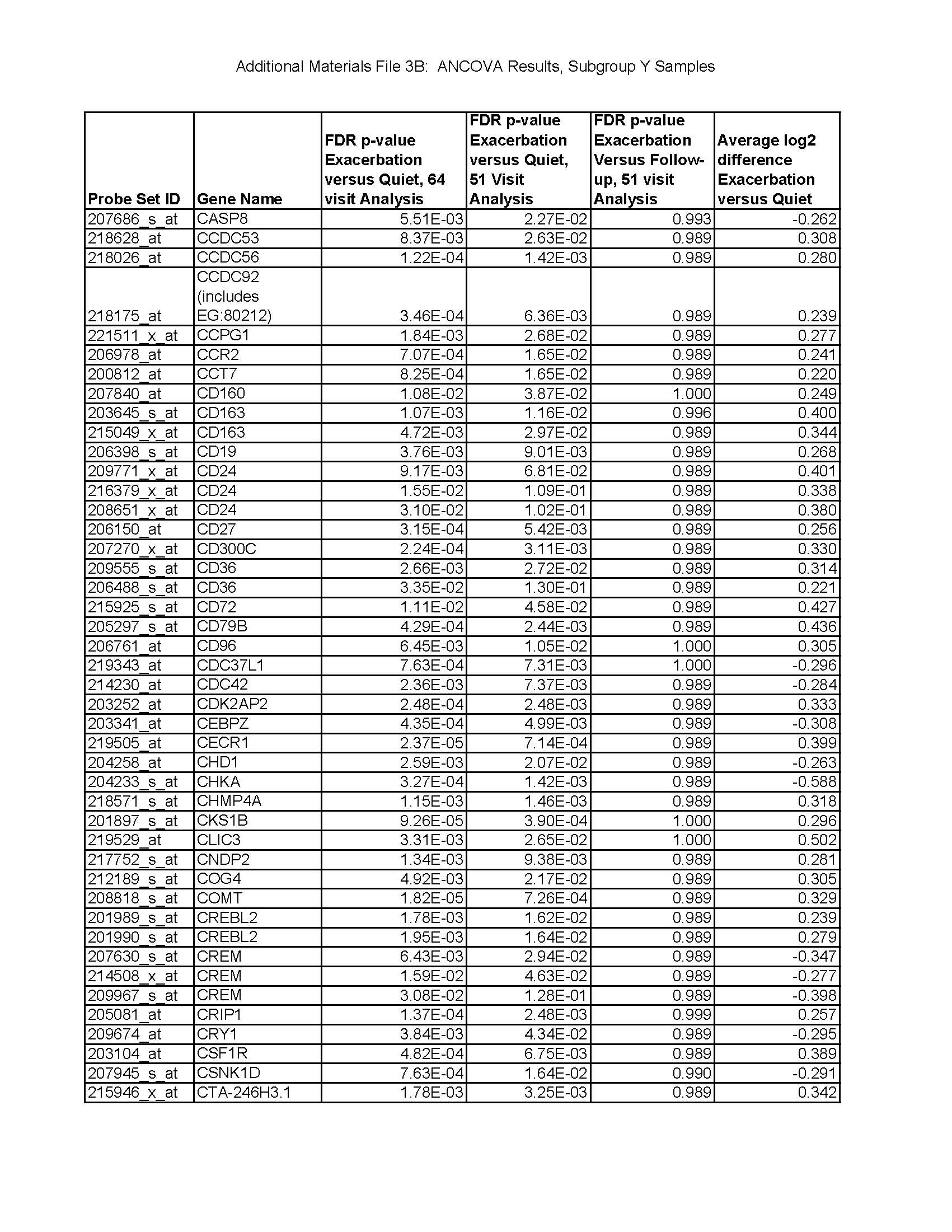


Table S18B: ANCOVA Results Subgroup Y continued
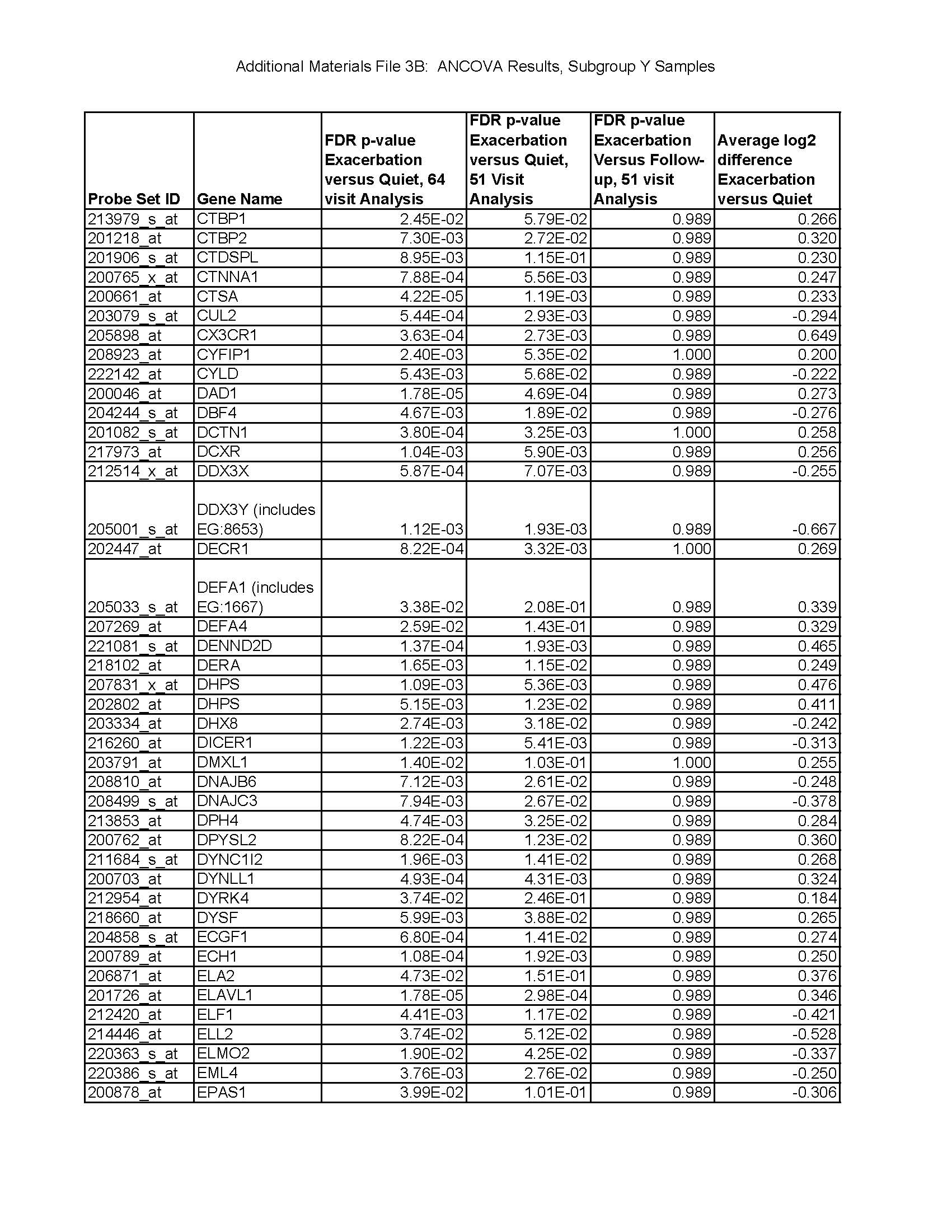


Table S18B: ANCOVA Results Subgroup Y continued
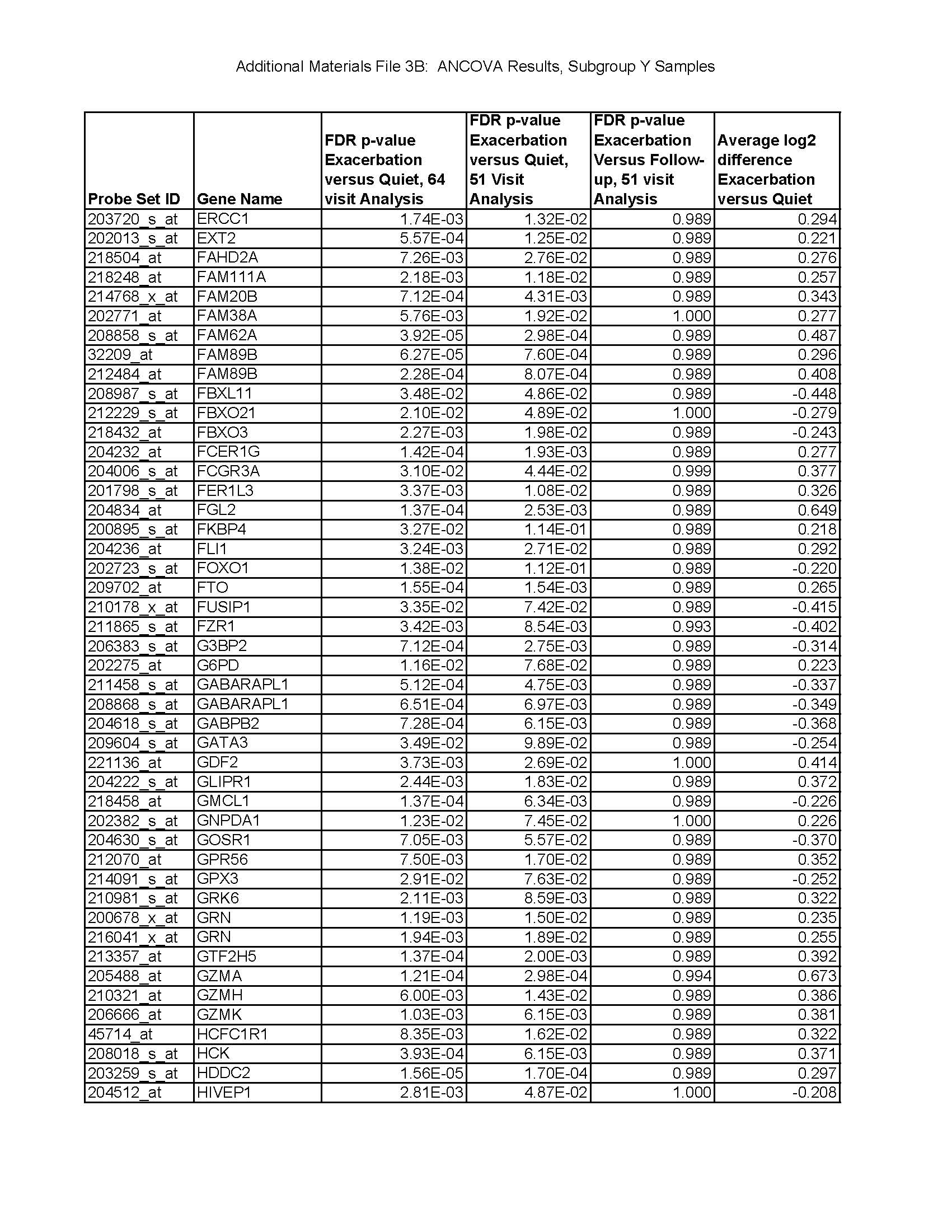


Table S18B: ANCOVA Results Subgroup Y continued
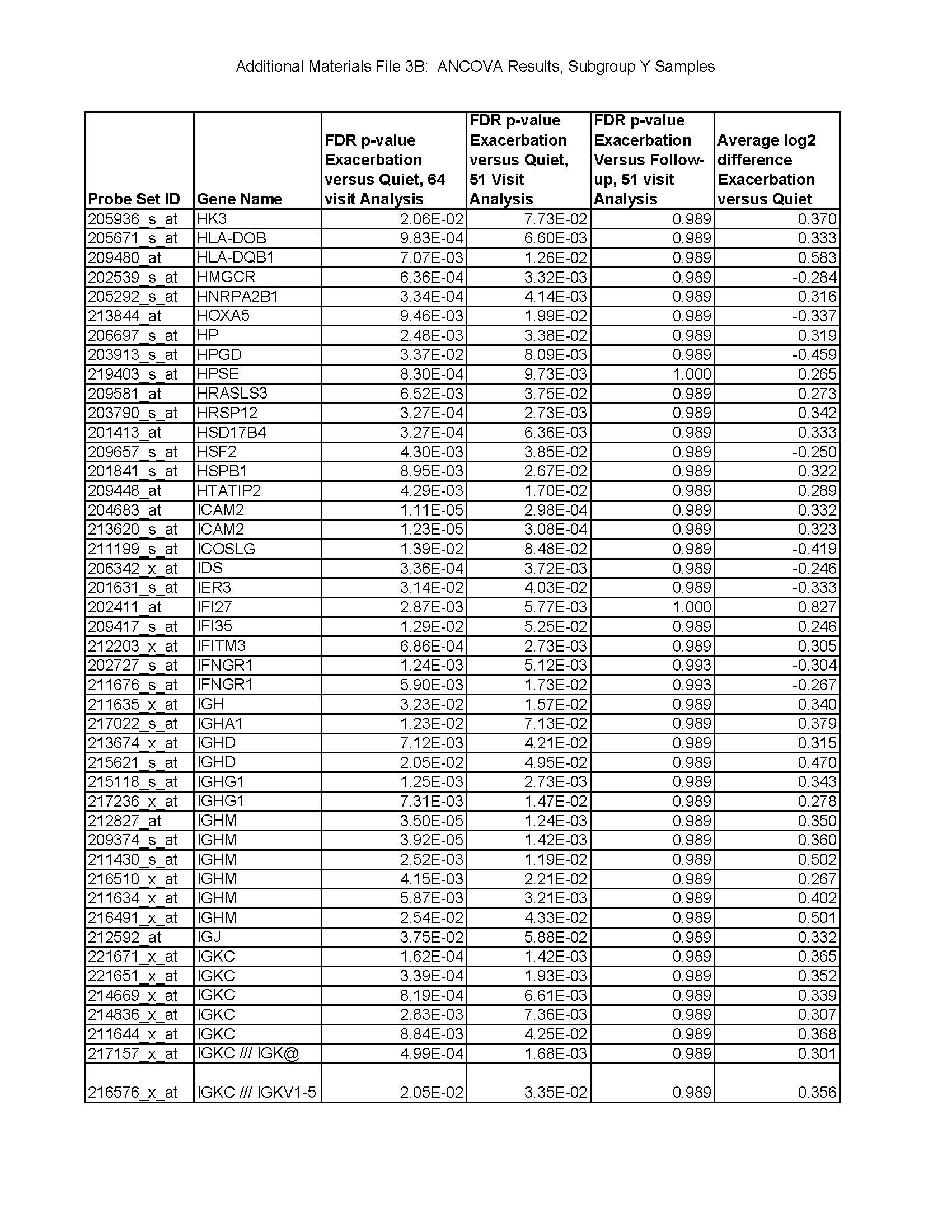


Table S18B: ANCOVA Results Subgroup Y continued
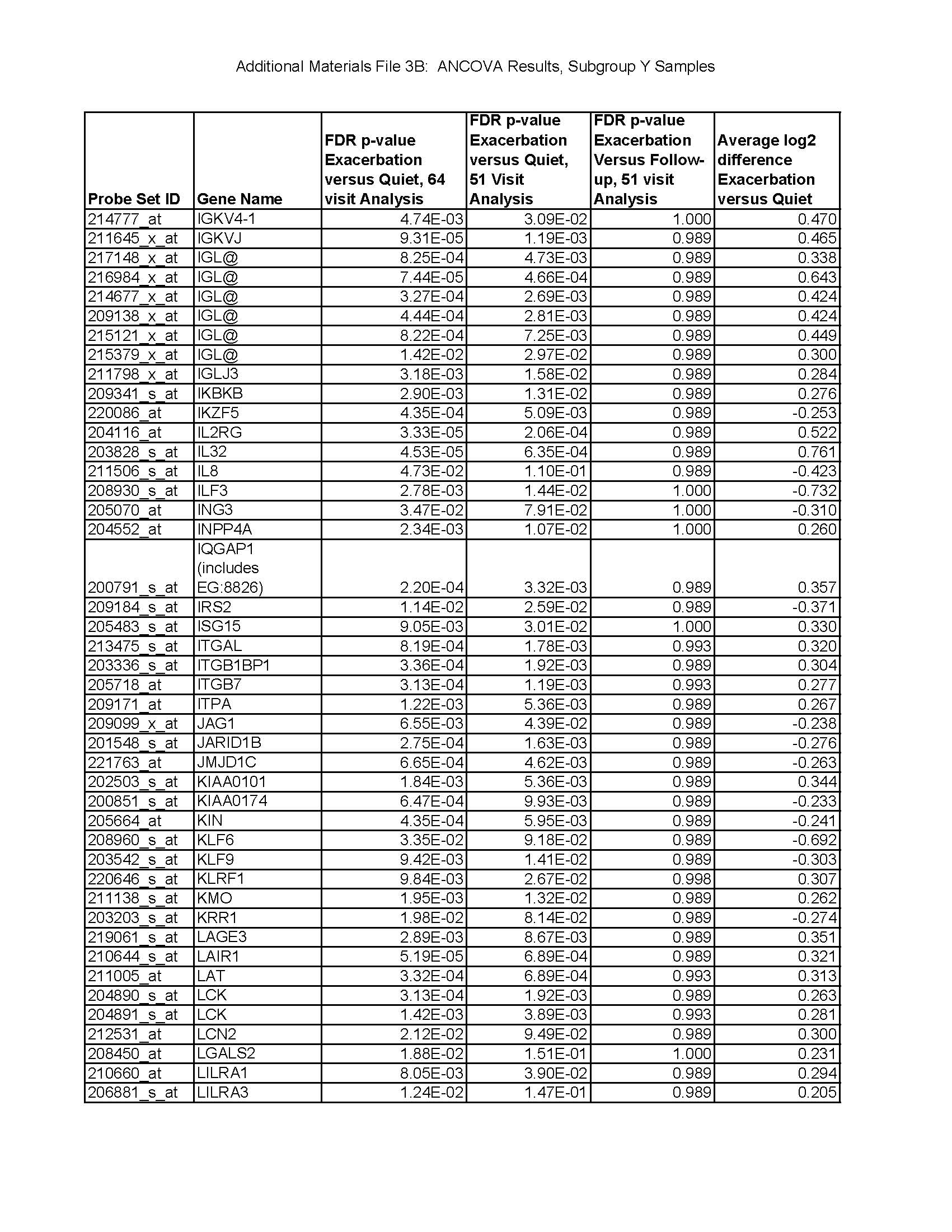


Table S18B: ANCOVA Results Subgroup Y continued
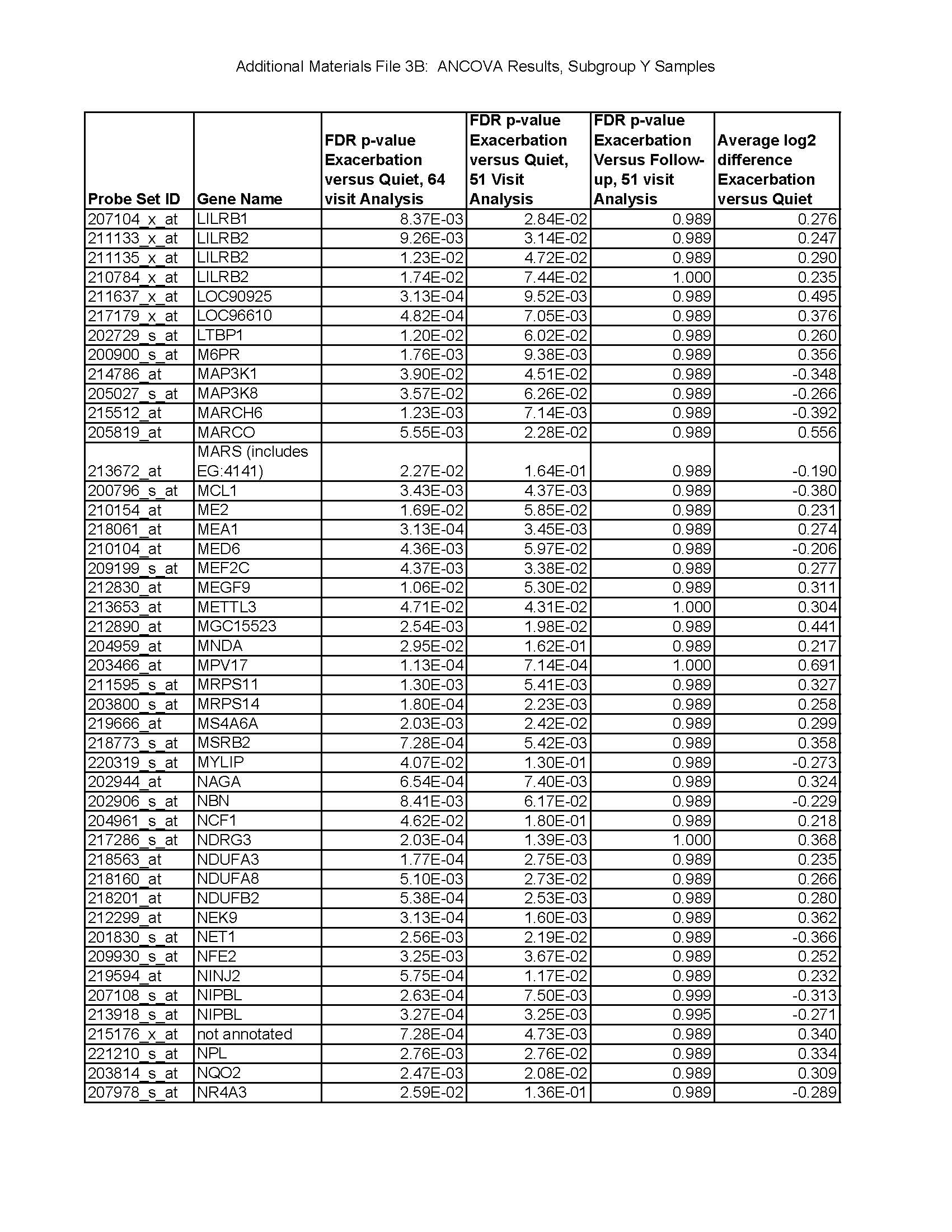


Table S18B: ANCOVA Results Subgroup Y continued
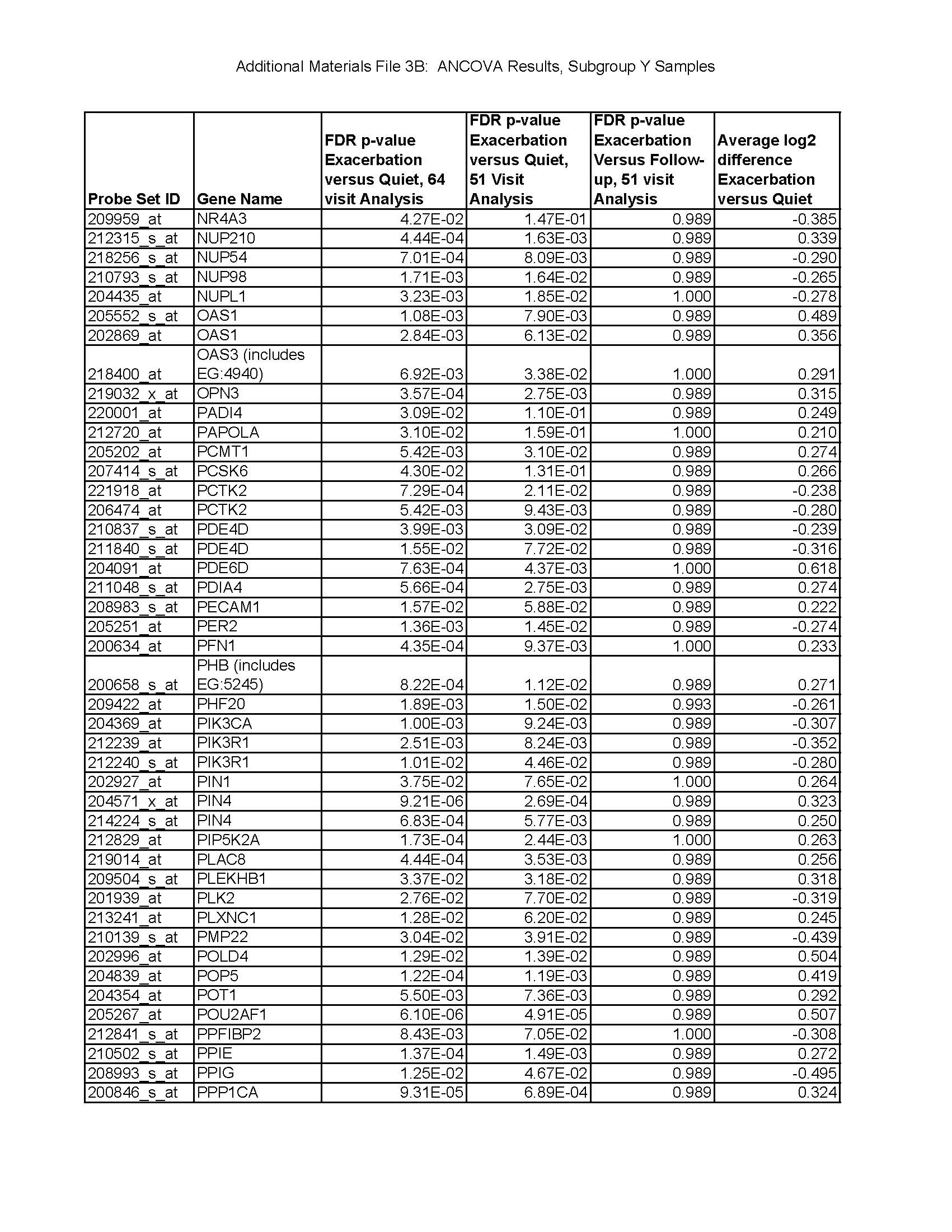


Table S18B: ANCOVA Results Subgroup Y continued
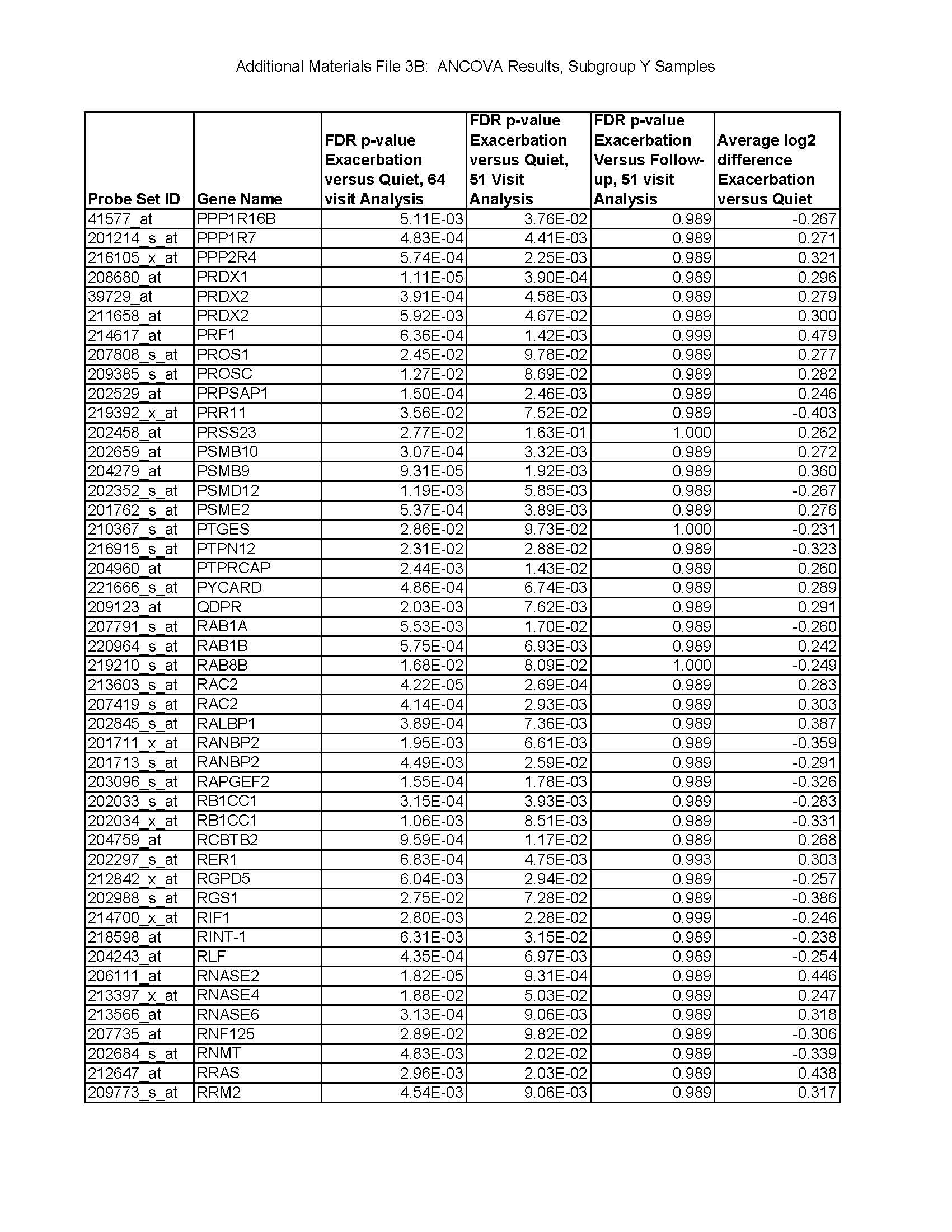


Table S18B: ANCOVA Results Subgroup Y continued
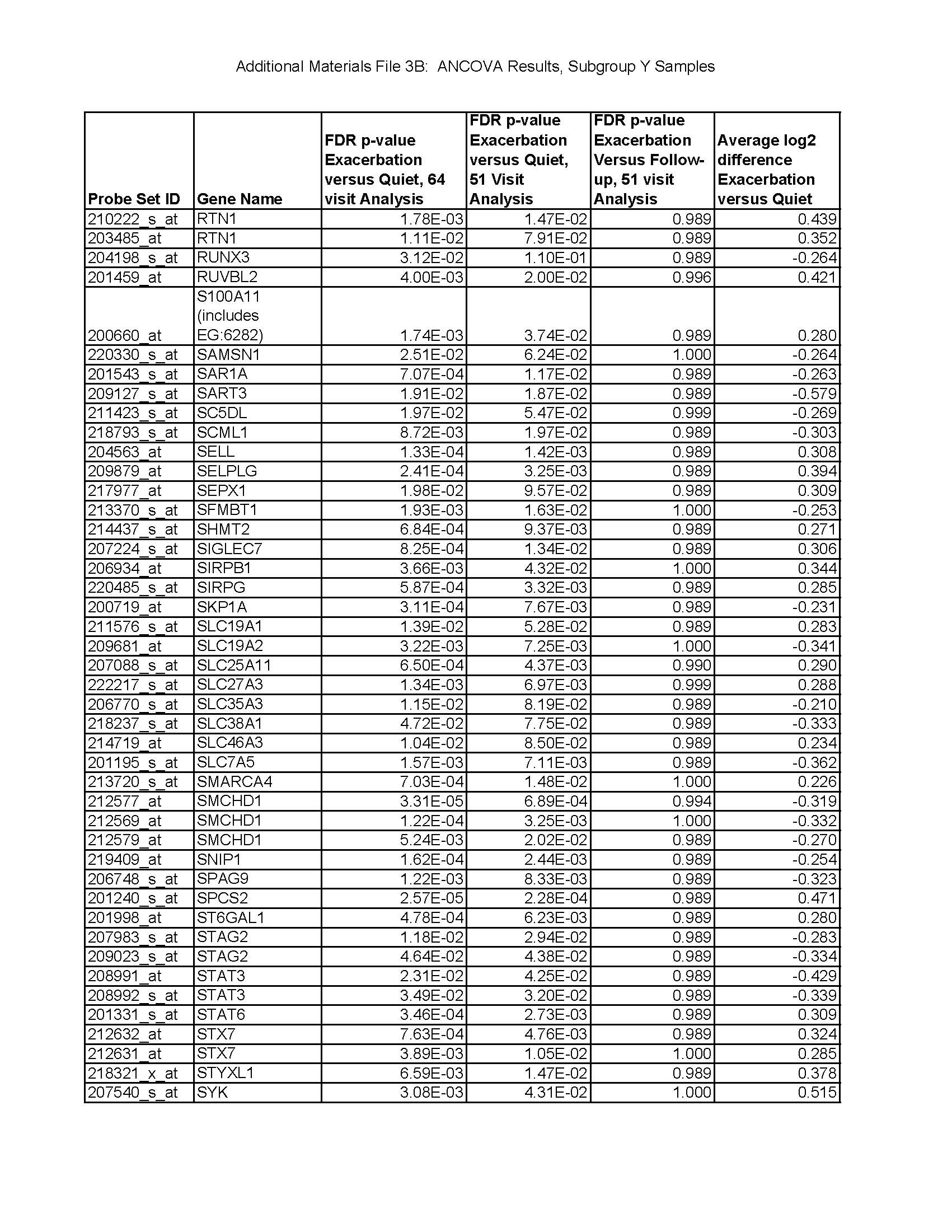
 Table S18B: ANCOVA Results Subgroup Y continued
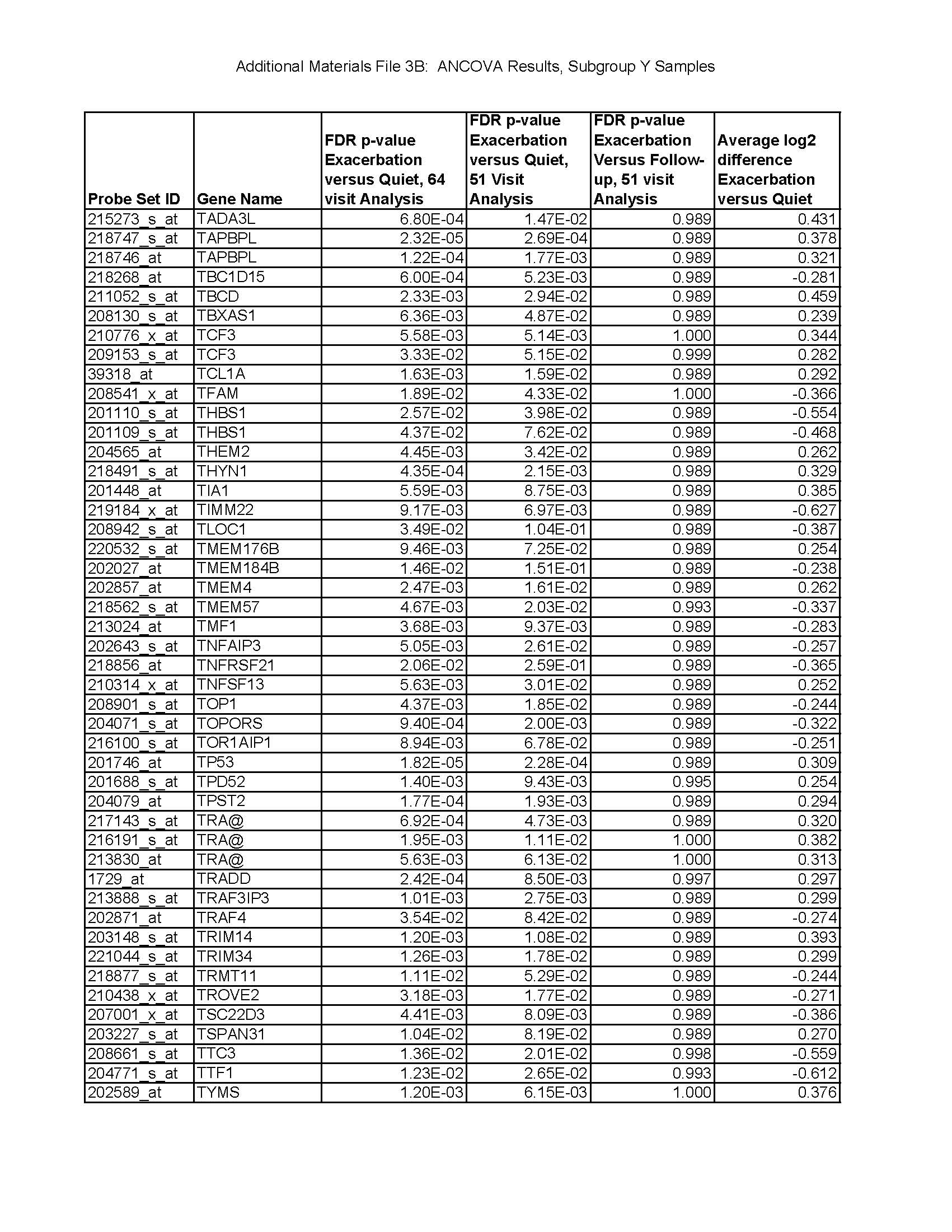


Table S18B: ANCOVA Results Subgroup Y continued


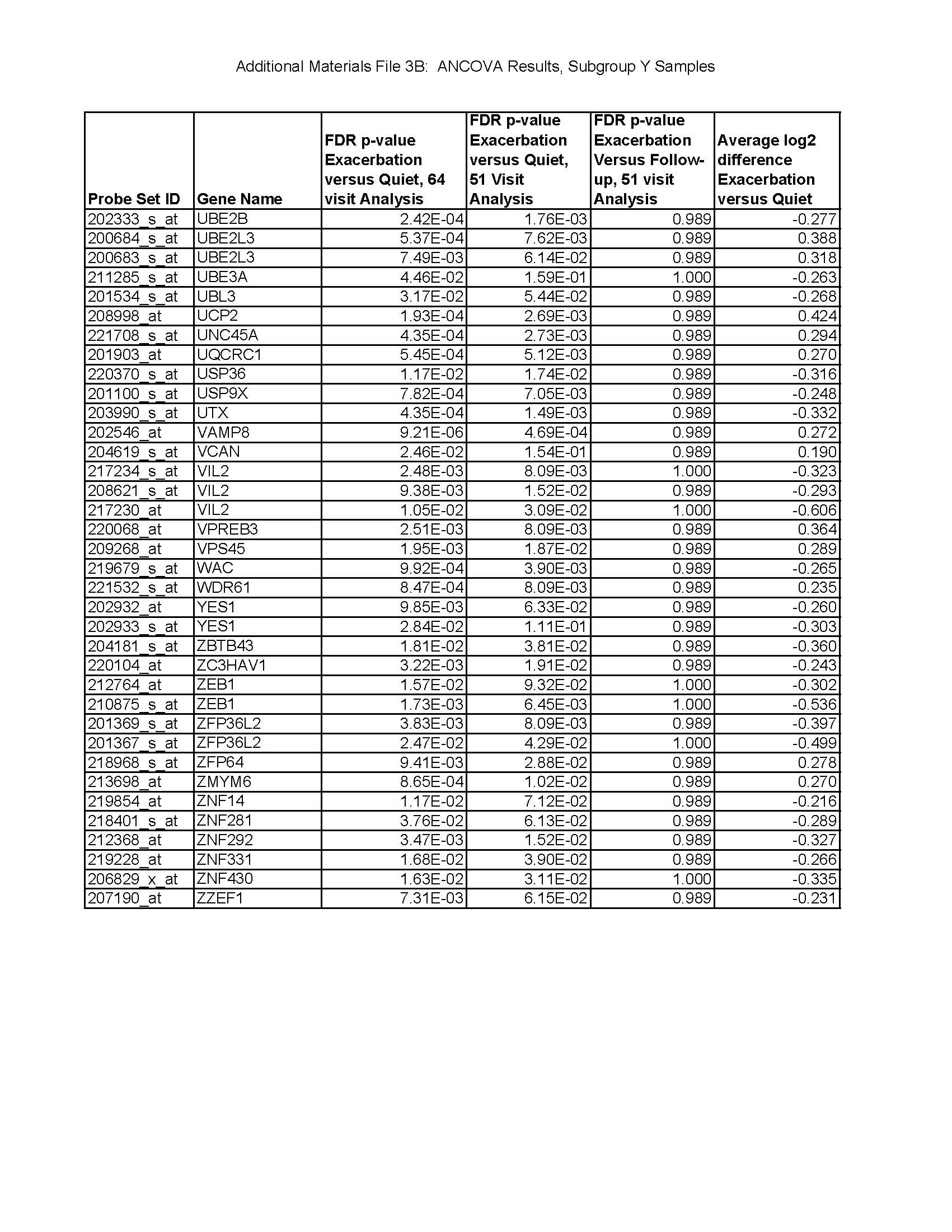


## Online Supporting Information Table S18C: ANCOVA Results Subgroup Z Samples


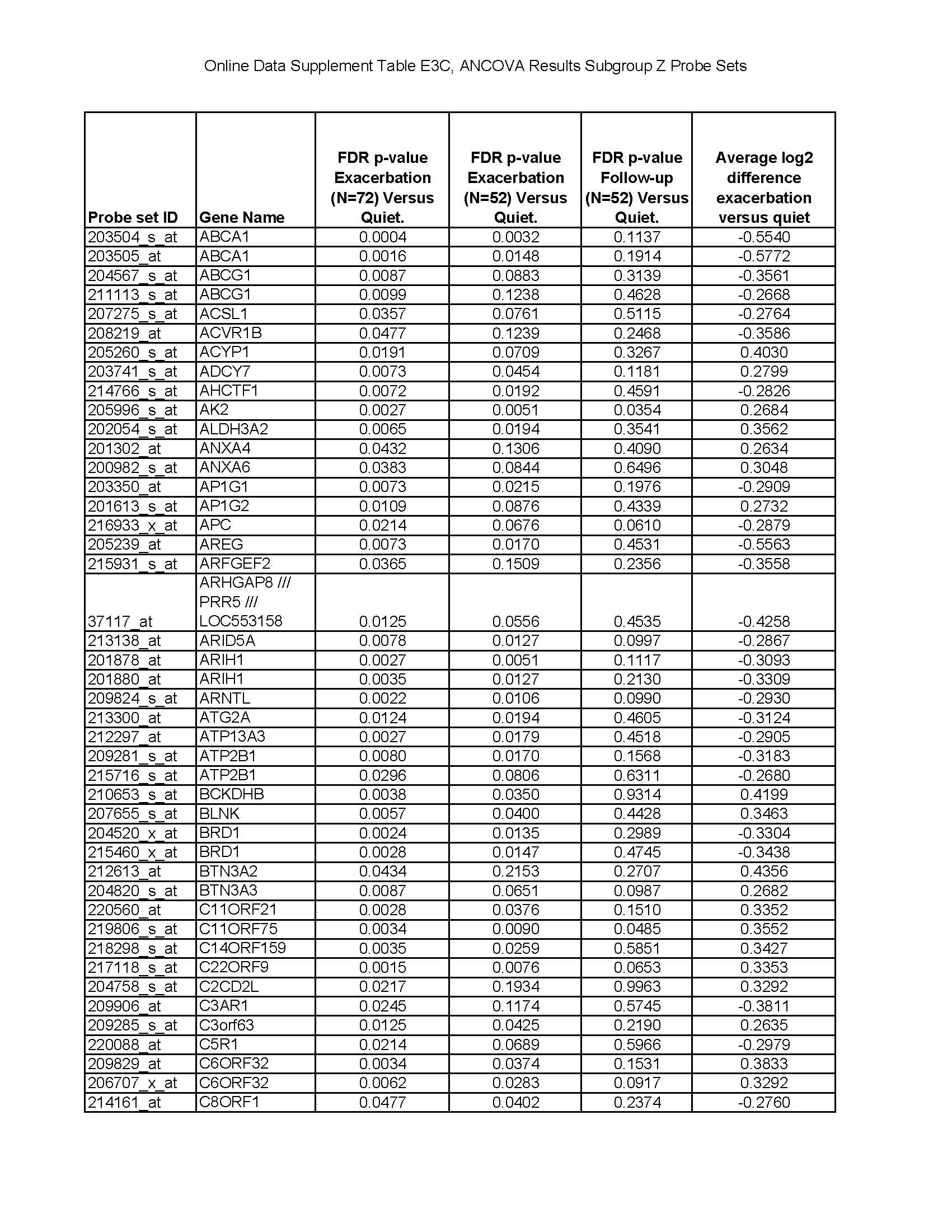


Table S18C: ANCOVA Results Subgroup Z continued
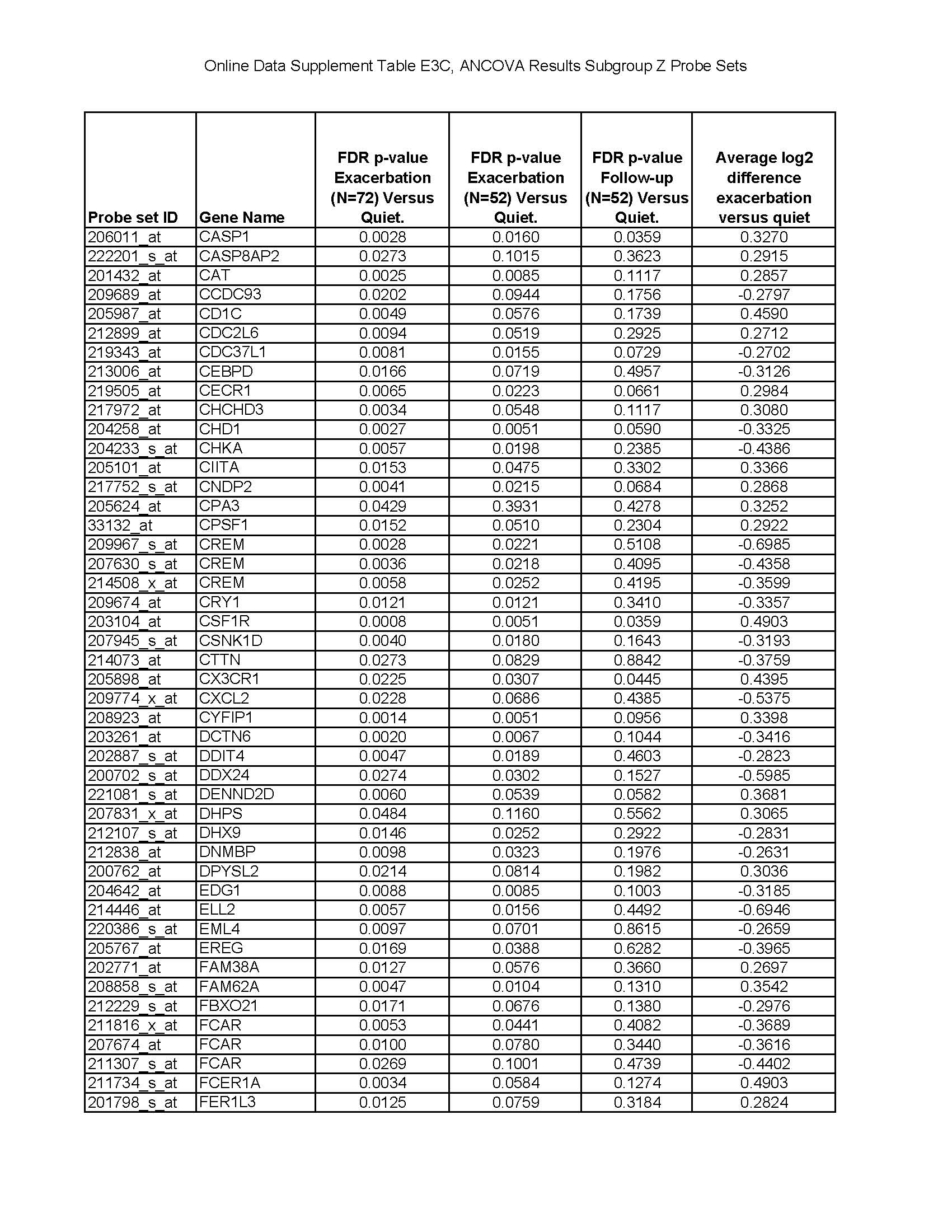


Table S18C: ANCOVA Results Subgroup Z continued
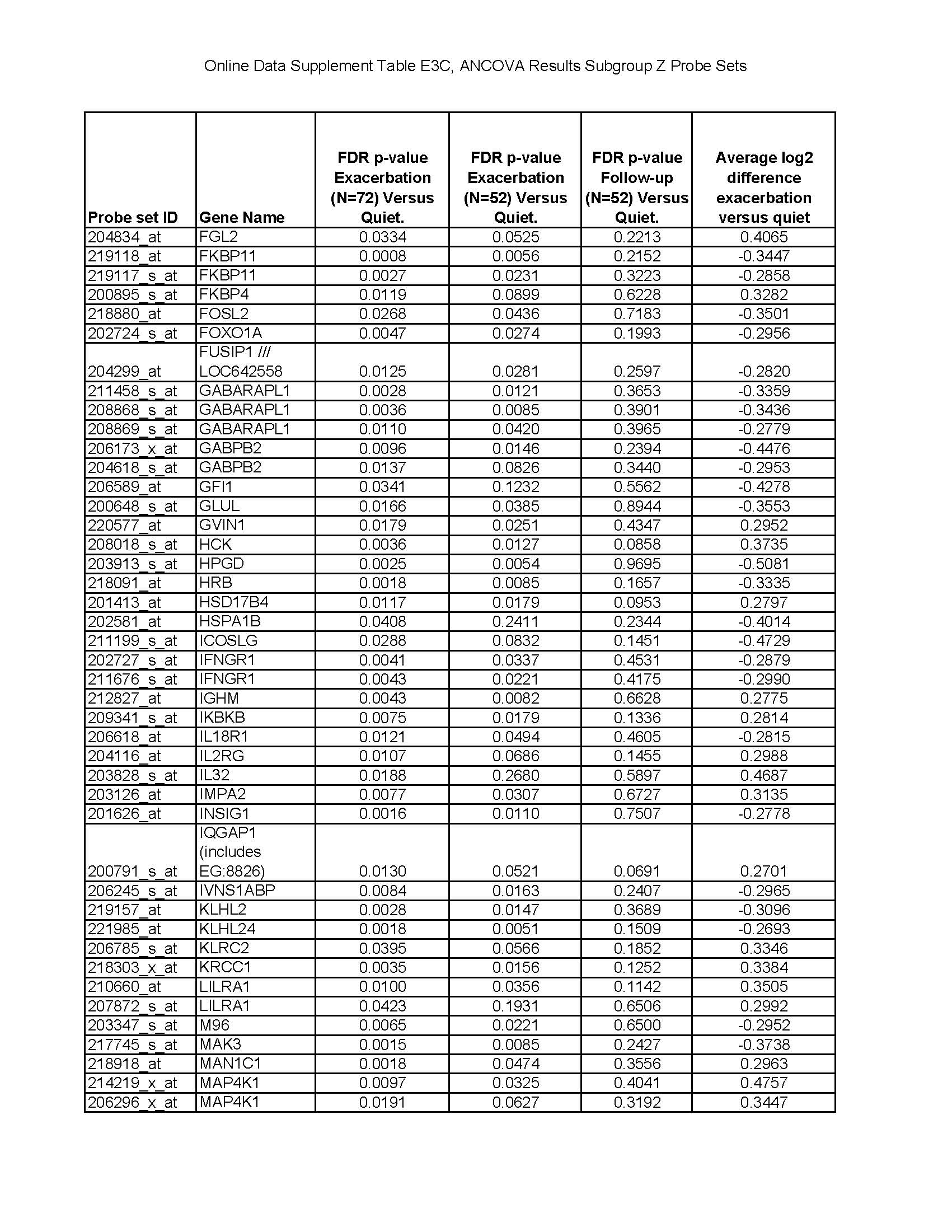


Table S18C: ANCOVA Results Subgroup Z continued
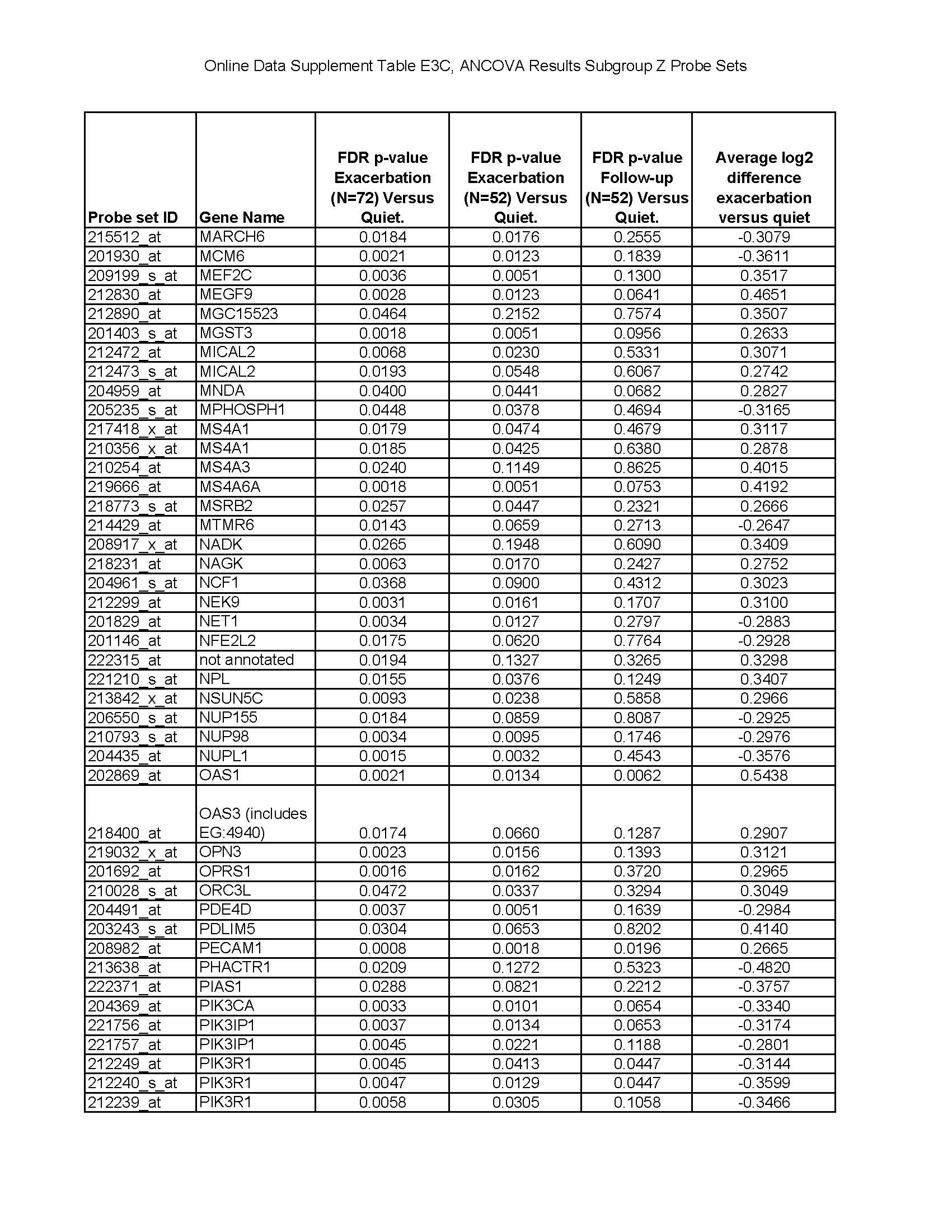


Table S18C: ANCOVA Results Subgroup Z continued
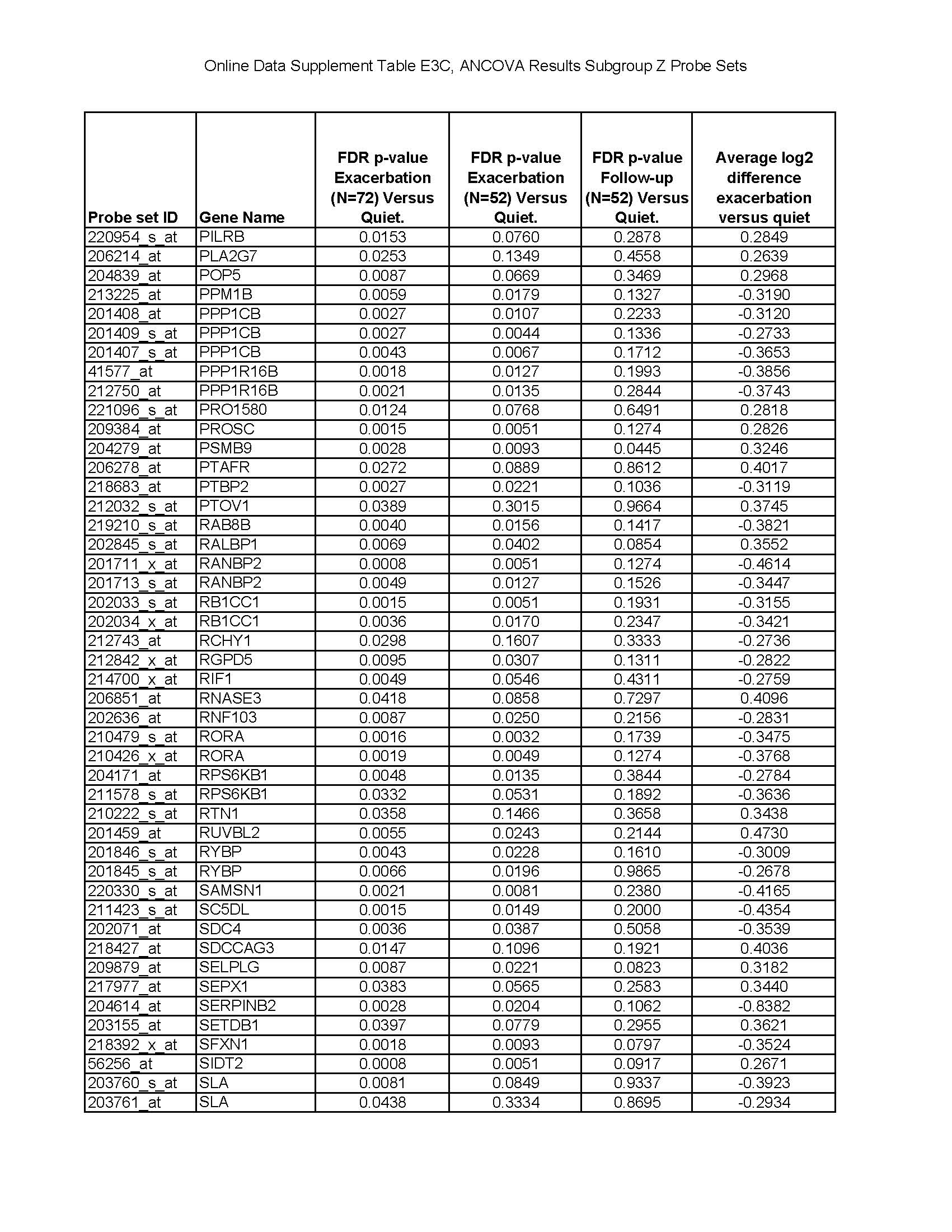


Table S18C: ANCOVA Results Subgroup Z continued
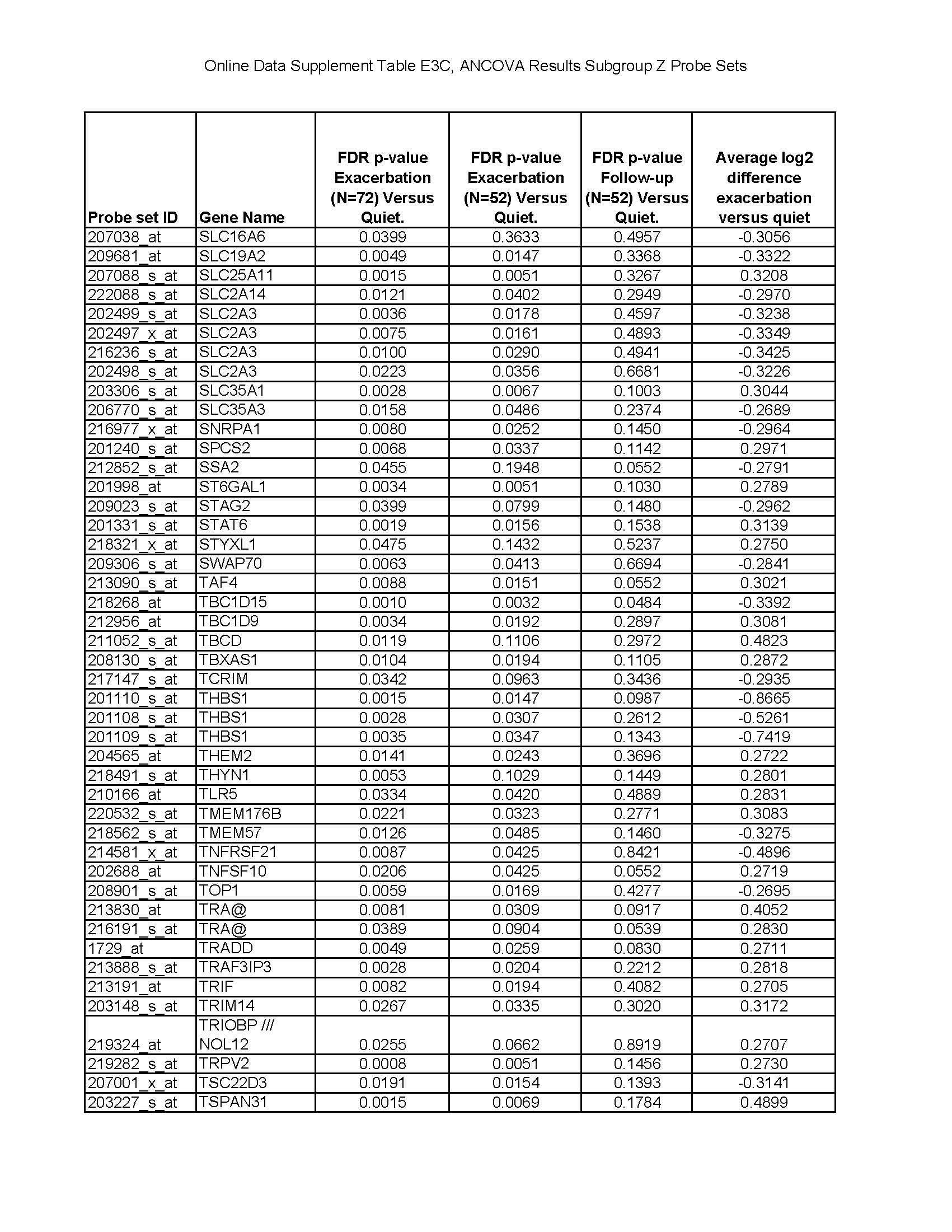


Table S18C: ANCOVA Results Subgroup Z continued


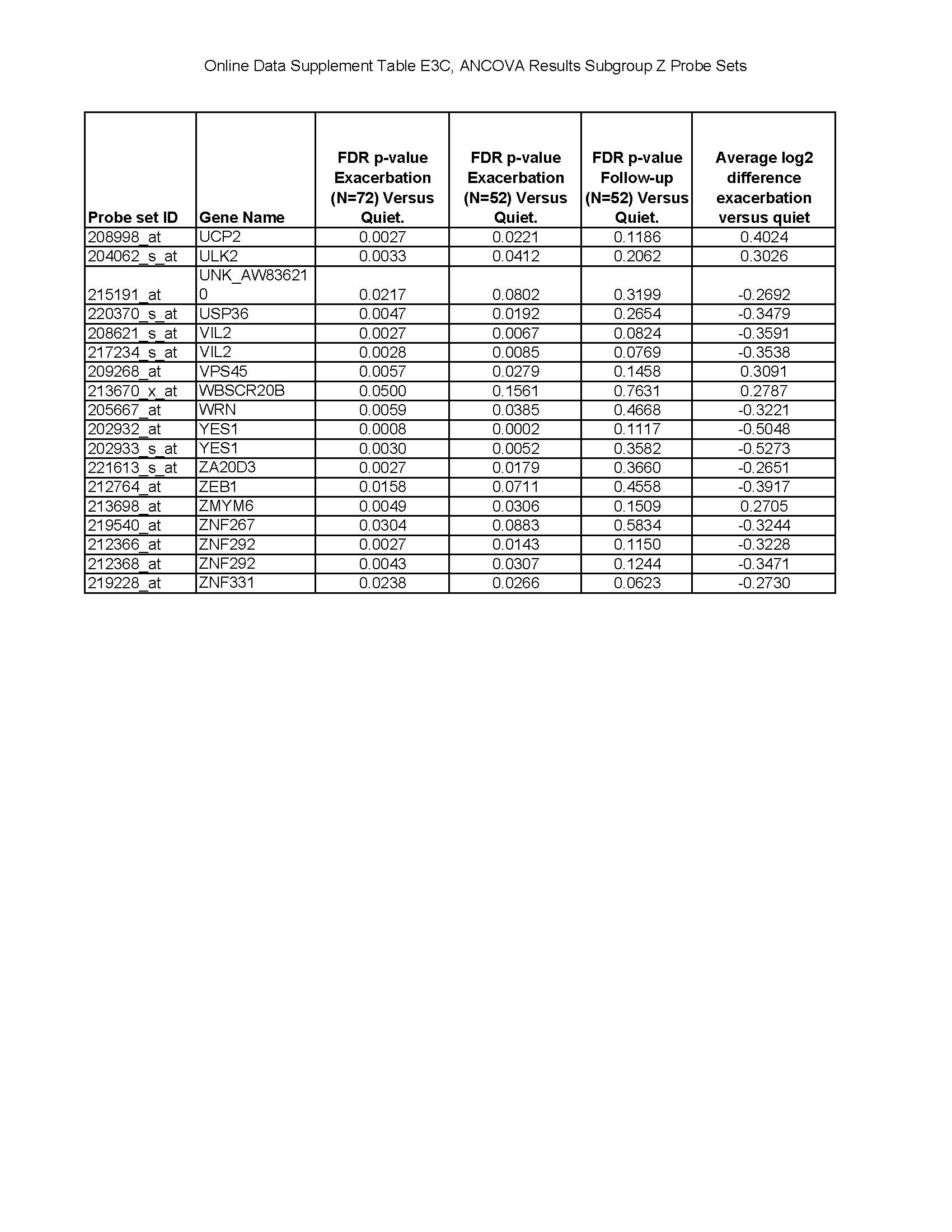

Supplement: Table S18 — ANCOVA results. A: subgroup X. B: subgroup Y C: subgroup Z. (DOC) [file pone.0021902.s025.doc]
