# Supplementary material for: Pathways Activated during Human Asthma Exacerbation as Revealed by Gene Expression Patterns in Blood
Source: PLoS One. 2011 Jul 14;6(7):e21902. doi: 10.1371/journal.pone.0021902 (PMC3136489; doi:10.1371/journal.pone.0021902)

Table S19: IL15 Pathway genes Associated with Exacerbation in Subgroup X continued


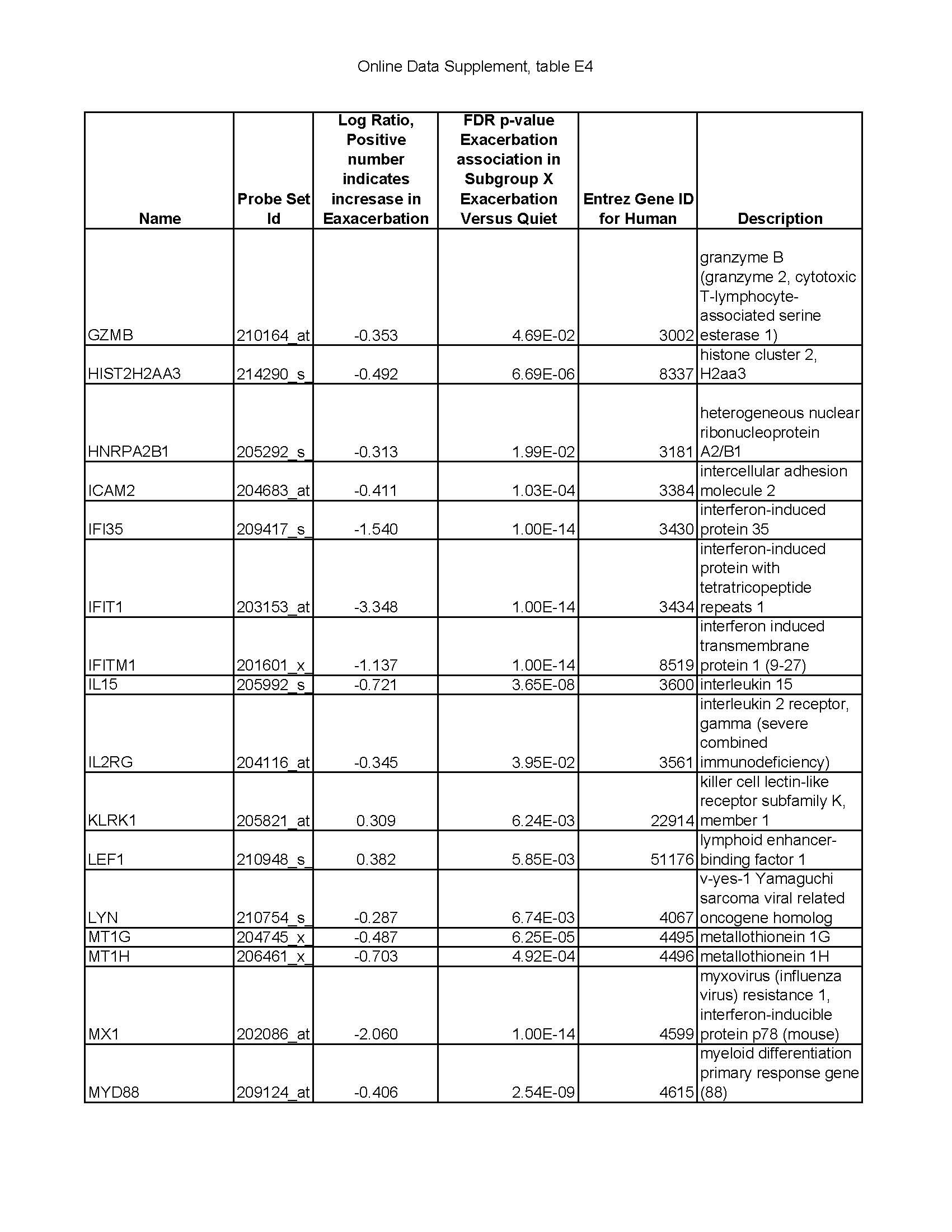


Table S19: IL15 Pathway genes Associated with Exacerbation in Subgroup X continued


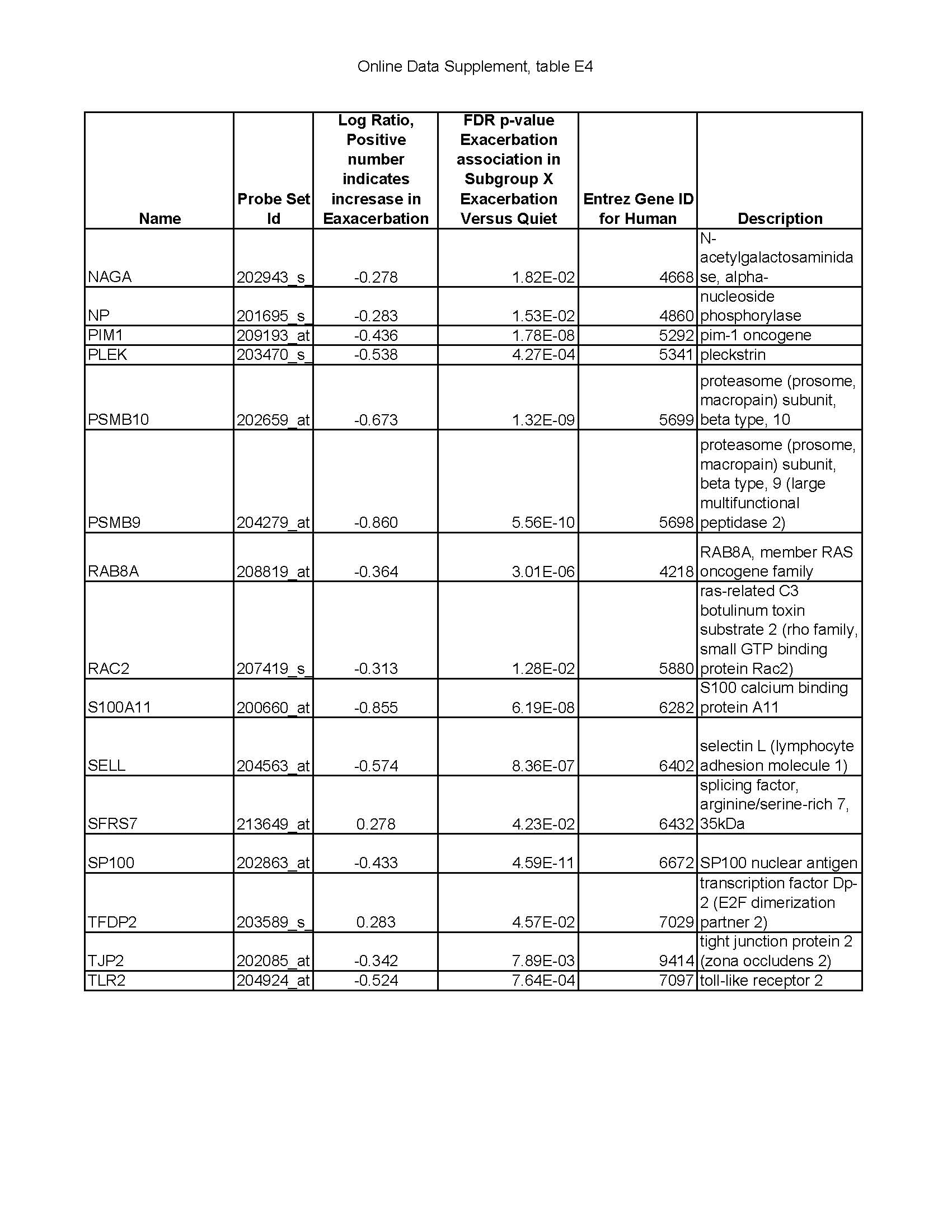

Supplement: Table S19 — IL15 pathway genes associated with exacerbation in subgroup X. (DOC) [file pone.0021902.s026.doc]
