# Supplementary material for: Pathways Activated during Human Asthma Exacerbation as Revealed by Gene Expression Patterns in Blood
Source: PLoS One. 2011 Jul 14;6(7):e21902. doi: 10.1371/journal.pone.0021902 (PMC3136489; doi:10.1371/journal.pone.0021902)
Supplement: Table S20 — Lack of subgroup association with FEV1 predicted. (DOC) [file pone.0021902.s027.doc]

### Online Supporting Information Table S20: Subgroup Association with FEV1 (predicted)

|  | **Subgroup based on K-means clustering (k=3) of 1079 probesets** | | |
| --- | --- | --- | --- |
| **Statistic** | **Subgroup X** | **SubgroupY** | **Subgroup Z** |
| N | 25 | 58 | 61 |
| Mean | 79.9 | 76.2 | 76.5 |
| Median | 82 | 82 | 77 |
| S.D. | 24.7 | 21.6 | 21.1 |
| CV | 30.9 | 28.3 | 27.6 |
| Missing values | 5 | 6 | 11 |

p-value from overall F-test = 0.67. Because the F-test p-value was not statistically significant at the 0.05 level, no pairwise comparisons between cluster means were performed.

Conclusion: No statistically significant differences among Subgroups in FEV1 (predicted) during exacerbation visits.
