# Supplementary material for: Pathways Activated during Human Asthma Exacerbation as Revealed by Gene Expression Patterns in Blood
Source: PLoS One. 2011 Jul 14;6(7):e21902. doi: 10.1371/journal.pone.0021902 (PMC3136489; doi:10.1371/journal.pone.0021902)
Supplement: Table S22 — Lack of subgroup association with FVC (predicted). (DOC) [file pone.0021902.s029.doc]

### Online Supporting Information Table S22: Subgroup Association with FVC (predicted)

|  | **Subgroup based on K-means clustering (k=3) of 1079 probesets** | | |
| --- | --- | --- | --- |
| **Statistic** | **Subgroup X** | **Subgroup Y** | **Subgroup Z** |
| N | 25 | 57 | 61 |
| Mean | 91.6 | 88.7 | 83.9 |
| Median | 94 | 92 | 80 |
| S.D. | 22.3 | 19.3 | 18.8 |
| CV | 24.4 | 21.8 | 22.4 |
| Missing values | 5 | 7 | 11 |

p-value from overall F-test = 0.53. Because the F-test p-value was not statistically significant at the 0.05 level, no pairwise comparisons between Subgroup means were performed.

Conclusion: No statistically significant differences among Subgroups in FVC (predicted) during exacerbation visits.
