# Supplementary material for: Pathways Activated during Human Asthma Exacerbation as Revealed by Gene Expression Patterns in Blood
Source: PLoS One. 2011 Jul 14;6(7):e21902. doi: 10.1371/journal.pone.0021902 (PMC3136489; doi:10.1371/journal.pone.0021902)
Supplement: Table S23 — Lack of subgroup association with FVC (predicted) change from baseline. (DOC) [file pone.0021902.s030.doc]

### Online Supporting Information Table S23: Subgroup Association with FVC (predicted) change from baseline

|  | **Subgroup based on K-means clustering (k=3) of 1079 probesets** | | |
| --- | --- | --- | --- |
| **Statistic** | **Subgroup X** | **Subgroup Y** | **Subgroup Z** |
| N | 25 | 56 | 60 |
| Mean | -8.7 | -4.7 | -4.2 |
| Median | -6.0 | -5.5 | -2.5 |
| S.D. | 11.4 | 17.3 | 13.7 |
| Missing values | 5 | 8 | 12 |

p-value from overall F-test = 0.28. Because the F-test p-value was not statistically significant at the 0.05 level, no pairwise comparisons between Subgroup means were performed.

Conclusion: No statistically significant differences among Subgroups in FVC (predicted) change from baseline during exacerbation visits.
