# Supplementary material for: Pathways Activated during Human Asthma Exacerbation as Revealed by Gene Expression Patterns in Blood
Source: PLoS One. 2011 Jul 14;6(7):e21902. doi: 10.1371/journal.pone.0021902 (PMC3136489; doi:10.1371/journal.pone.0021902)
Supplement: Table S24 — Lack of subgroup association with FEF 25–75% (predicted). (DOC) [file pone.0021902.s031.doc]

### Online Supporting Information Table S24: Subgroup Association with FEF 25-75% (predicted)

|  | **Subgroup based on K-means clustering (k=3) of 1079 probesets** | | |
| --- | --- | --- | --- |
| **Statistic** | **Subgroup X** | **Subgroup Y** | **Subgroup Z** |
| N | 24 | 57 | 61 |
| Mean | 55.6 | 53.3 | 60.0 |
| Median | 52 | 53 | 53 |
| S.D. | 25.1 | 28.7 | 30.4 |
| CV | 45.1 | 53.8 | 50.6 |
| Missing values | 6 | 7 | 11 |

p-value from overall F-test = 0.20. Because the F-test p-value was not statistically significant at the 0.05 level, no pairwise comparisons between Subgroup means were performed.

Conclusion: No statistically significant differences among Subgroups in FEF 25-75% (predicted) during exacerbation visits.
