# Supplementary material for: Pathways Activated during Human Asthma Exacerbation as Revealed by Gene Expression Patterns in Blood
Source: PLoS One. 2011 Jul 14;6(7):e21902. doi: 10.1371/journal.pone.0021902 (PMC3136489; doi:10.1371/journal.pone.0021902)
Supplement: Table S26 — Lack of subgroup association with PEF (predicted) change from baseline. (DOC) [file pone.0021902.s033.doc]

### Online Supporting Information Table S26: Subgroup Association with PEF (predicted)

|  | **Subgroup based on K-means clustering (k=3) of 1079 probesets** | | |
| --- | --- | --- | --- |
| **Statistic** | **Subgroup X** | **Subgroup Y** | **Subgroup Z** |
| N | 23 | 53 | 60 |
| Mean | 80.2 | 80.2 | 81.8 |
| Median | 95 | 93 | 86 |
| S.D. | 33.5 | 26.6 | 29.4 |
| CV | 41.8 | 33.1 | 36.0 |
| Missing values | 7 | 11 | 12 |

p-value from overall F-test = 0.66. Because the F-test p-value was not statistically significant at the 0.05 level, no pairwise comparisons between Subgroup means were performed.

Conclusion: No statistically significant differences among Subgroups in PEF (predicted) during exacerbation visits.
