# Supplementary material for: Pathways Activated during Human Asthma Exacerbation as Revealed by Gene Expression Patterns in Blood
Source: PLoS One. 2011 Jul 14;6(7):e21902. doi: 10.1371/journal.pone.0021902 (PMC3136489; doi:10.1371/journal.pone.0021902)
Supplement: Table S27 — Lack of subgroup association with PEF (predicted) change from baseline. (DOC) [file pone.0021902.s034.doc]

### Online Supporting Information Table S27: Subgroup Association with PEF (predicted) change from baseline

|  | **Subgroup based on K-means clustering (k=3) of 1079 probesets** | | |
| --- | --- | --- | --- |
| **Statistic** | **Subgroup X** | **Subgroup Y** | **Subgroup Z** |
| N | 23 | 50 | 57 |
| Mean | -7.2 | -4.6 | -4.2 |
| Median | -5.3 | -6.0 | -3.7 |
| S.D. | 19.4 | 15.1 | 15.8 |
| Missing values | 7 | 14 | 15 |

p-value from overall F-test = 0.62. Because the F-test p-value was not statistically significant at the 0.05 level, no pairwise comparisons between Subgroup means were performed.

Conclusion: No statistically significant differences among Subgroups in PEF (predicted) change from baseline during exacerbation visits.
