# Supplementary material for: Pathways Activated during Human Asthma Exacerbation as Revealed by Gene Expression Patterns in Blood
Source: PLoS One. 2011 Jul 14;6(7):e21902. doi: 10.1371/journal.pone.0021902 (PMC3136489; doi:10.1371/journal.pone.0021902)
Supplement: Table S28 — Lack of subgroup association with relevant respiratory infection. (DOC) [file pone.0021902.s035.doc]

## Online Supporting Information Table S28: Subgroup Association with Relevant Respiratory Infection

(visit-level variable, using respiratory infection as defined by Cristina Csimma)

|  | Subgroup based on K-means clustering (k=3) of 1079 probesets | | |  |
| --- | --- | --- | --- | --- |
| Any relevant infections | Subgroup X | Subgroup Y | Subgroup Z | Total |
| No | 10 (33.3%) | 28 (43.8%) | 39 (54.2%) | 77 |
| Yes | 20 (66.7%) | 36 (56.3%) | 33 (45.8%) | 89 |
| Total | 30 | 64 | 72 | 166 |

p-value = 0.14

Conclusion: No evidence of association between relevant infections and Subgroup assignments.
