# Supplementary material for: Pathways Activated during Human Asthma Exacerbation as Revealed by Gene Expression Patterns in Blood
Source: PLoS One. 2011 Jul 14;6(7):e21902. doi: 10.1371/journal.pone.0021902 (PMC3136489; doi:10.1371/journal.pone.0021902)
Supplement: Table S29 — Lack of subgroup association with disease severity. (DOC) [file pone.0021902.s036.doc]

## Online Supporting Information Table S29: Subgroup Association with Disease Severity

(donor-level variable)

|  | Subgroup based on K-means clustering (k=3) of 1079 probesets | | |  |
| --- | --- | --- | --- | --- |
| Severity | Subgroup X | Subgroup Y | Subgroup Z | Total |
| Mild | 2 (6.7%) | 4 (6.3%) | 1 (1.4%) | 7 |
| Moderate | 14 (46.7%) | 23 (35.9%) | 31 (43.1%) | 68 |
| Severe | 14 (46.7%) | 37 (57.8%) | 40 (55.6%) | 91 |
| Total | 30 | 64 | 72 | 166 |

p-value = 0.46 (note: would be better with exact test p-values)

Conclusion: No evidence of association between severity and Subgroup assignments.
