# Supplementary material for: Pathways Activated during Human Asthma Exacerbation as Revealed by Gene Expression Patterns in Blood
Source: PLoS One. 2011 Jul 14;6(7):e21902. doi: 10.1371/journal.pone.0021902 (PMC3136489; doi:10.1371/journal.pone.0021902)
Supplement: Table S31 — Lack of subgroup association with use of medication: inhaled corticosteroids. (DOC) [file pone.0021902.s038.doc]

### Online Supporting Information Table S31: Subgroup Association with Use of Medication: Inhaled Corticosteroids

(visit-level variable, using non-study medication classification of Charlotte McKee)

|  | Subgroup based on K-means clustering (k=3) of 1079 probesets | | |  |
| --- | --- | --- | --- | --- |
| Any inhaled steroid use | Subgroup X | Subgroup Y | Subgroup Z | Total |
| No | 1 (3.3) | 5 (7.8%) | 2 (2.8%) | 8 |
| Yes | 29 (96.7%) | 59 (92.2%) | 70 (97.2%) | 158 |
| Total | 30 | 64 | 72 | 166 |

p-value = 0.36 (would be better with exact test p-value)

Conclusion: No evidence of association between inhaled corticosteroid use and Subgroup assignments.
