# Supplementary material for: Pathways Activated during Human Asthma Exacerbation as Revealed by Gene Expression Patterns in Blood
Source: PLoS One. 2011 Jul 14;6(7):e21902. doi: 10.1371/journal.pone.0021902 (PMC3136489; doi:10.1371/journal.pone.0021902)
Supplement: Table S32 — Lack of subgroup association with use of medication: intranasal corticosteroids. (DOC) [file pone.0021902.s039.doc]

### Online Supporting Information Table S32: Subgroup Association with Use of Medication: Association with Use of Intranasal Corticosteroids

(visit-level variable, using non-study medication classification of Charlotte McKee, Medical Monitor)

|  | Subgroup based on K-means clustering (k=3) of 1079 probesets | | |  |
| --- | --- | --- | --- | --- |
| Any intranasal steroid use | Subgroup X | Subgroup Y | Subgroup Z | Total |
| No | 20 (66.7%) | 32 (50.0%) | 39 (54.2%) | 91 |
| Yes | 10 (33.3%) | 32 (50.0%) | 33 (45.8%) | 75 |
| Total | 30 | 64 | 72 | 166 |

p-value = 0.31

Conclusion: No evidence of association between intranasal corticosteroid use and node assignments.
