# Supplementary material for: Pathways Activated during Human Asthma Exacerbation as Revealed by Gene Expression Patterns in Blood
Source: PLoS One. 2011 Jul 14;6(7):e21902. doi: 10.1371/journal.pone.0021902 (PMC3136489; doi:10.1371/journal.pone.0021902)
Supplement: Table S33 — Lack of subgroup association with use of medication: leukotriene antagonists. (DOC) [file pone.0021902.s040.doc]

### Online Supporting Information Table S33: Subgroup Association with Use of Medication: Leukotriene Antagonists

(visit-level variable, using non-study medication classification of Charlotte McKee)

|  | Subgroup based on K-means clustering (k=3) of 1079 probesets | | |  |
| --- | --- | --- | --- | --- |
| Any leukotriene antagonist use | Subgroup X | Subgroup Y | Subgroup Z | Total |
| No | 19 (63.3%) | 48 (75.0%) | 48 (66.7%) | 115 |
| Yes | 11 (36.7%) | 16 (25.0%) | 24 (33.3%) | 51 |
| Total | 30 | 64 | 72 | 166 |

p-value = 0.43

Conclusion: No evidence of association between leukotriene antagonist use and the Subgroup assignments.
