# Supplementary material for: Pathways Activated during Human Asthma Exacerbation as Revealed by Gene Expression Patterns in Blood
Source: PLoS One. 2011 Jul 14;6(7):e21902. doi: 10.1371/journal.pone.0021902 (PMC3136489; doi:10.1371/journal.pone.0021902)
Supplement: Table S35 — Lack of subgroup association with use of medication: Any PPI non-study medication use. (DOC) [file pone.0021902.s042.doc]

### Online Supporting Information Table S35: Subgroup Association with Use of Medication: Any PPI Non-study Med Use

(visit-level analysis)

|  | Subgroup based on K-means clustering (k=3) of 1079 probesets | | |  |
| --- | --- | --- | --- | --- |
| Any PPI non-study med use? | Subgroup X | Subgroup Y | Subgroup Z | Total |
| No | 22 (73.3%) | 44 (68.8%) | 52 (72.2%) | 118 |
| Yes | 8 (26.7%) | 20 (31.3%) | 20 (27.8%) | 48 |
| Total | 30 | 64 | 72 | 166 |

p-value = 0.87

Conclusion: No evidence of association between PPI non-study medication use and Subgroup assignments.
