# Supplementary material for: Pathways Activated during Human Asthma Exacerbation as Revealed by Gene Expression Patterns in Blood
Source: PLoS One. 2011 Jul 14;6(7):e21902. doi: 10.1371/journal.pone.0021902 (PMC3136489; doi:10.1371/journal.pone.0021902)
Supplement: Table S36 — Lack of subgroup association with use of medication: any histamine H2 antagonist non-study medication use. (DOC) [file pone.0021902.s043.doc]

### Online Supporting Information Table S36: Subgroup Association with Use of Medication: Association with Any Histamine H2 Antagonist Non-study Med Use

(visit-level analysis)

|  | Subgroup based on K-means clustering (k=3) of 1079 probesets | | |  |
| --- | --- | --- | --- | --- |
| Any H2 antagonist non-study med use? | Subgroup X | Subgroup Y | Subgroup Z | Total |
| No | 30 (100%) | 63 (98.4%) | 72 (100%) | 165 |
| Yes | 0 (0%) | 1 (1.6%) | 0 (0%) | 1 |
| Total | 30 | 64 | 72 | 166 |

p-value = 0.45 (would be better with exact test p-values)

Conclusion: No evidence of association between H2 antagonist non-study medication use and Subgroup assignments.
