# Supplementary material for: Pathways Activated during Human Asthma Exacerbation as Revealed by Gene Expression Patterns in Blood
Source: PLoS One. 2011 Jul 14;6(7):e21902. doi: 10.1371/journal.pone.0021902 (PMC3136489; doi:10.1371/journal.pone.0021902)
Supplement: Table S37 — Lack of subgroup association with sex. (DOC) [file pone.0021902.s044.doc]

## Online Supporting Information Table S37: Subgroup Association with Sex

(donor-level variable)

|  | Subgroup based on K-means clustering (k=3) of 1079 probesets | | |  |
| --- | --- | --- | --- | --- |
| Sex | Subgroup X | Subgroup Y | Subgroup Z | Total |
| F | 17 (56.7%) | 46 (71.9%) | 54 (75.0%) | 117 |
| M | 13 (43.3%) | 18 (28.1%) | 18 (25.0%) | 49 |
| Total | 30 | 64 | 72 | 166 |

p-value = 0.17

Conclusion: No evidence for association between sex and Subgroup assignments.
