# Supplementary material for: Pathways Activated during Human Asthma Exacerbation as Revealed by Gene Expression Patterns in Blood
Source: PLoS One. 2011 Jul 14;6(7):e21902. doi: 10.1371/journal.pone.0021902 (PMC3136489; doi:10.1371/journal.pone.0021902)
Supplement: Table S38 — Subgroup assignment is not associated with race. (DOC) [file pone.0021902.s045.doc]

## Online Supporting Information Table S38: Subgroup Association with Race

(donor-level variable)

|  | Subgroup based on K-means clustering (k=3) of 1079 probesets | | |  |
| --- | --- | --- | --- | --- |
| Race | Subgroup X | Subgroup Y | Subgroup Z | Total |
| A | 1 (3.3%) | 1 (1.6%) | 3 (4.2%) | 5 |
| B | 5 (16.7%) | 3 (4.7%) | 8 (11.1%) | 16 |
| W | 24 (80.0%) | 60 (93.8%) | 61 (84.7%) | 145 |
| Total | 30 | 64 | 72 | 166 |

p-value = 0.33 (note: would be better with exact test p-values)

Conclusion: No evidence for association between race and Subgroup assignments.
