# Supplementary material for: Pathways Activated during Human Asthma Exacerbation as Revealed by Gene Expression Patterns in Blood
Source: PLoS One. 2011 Jul 14;6(7):e21902. doi: 10.1371/journal.pone.0021902 (PMC3136489; doi:10.1371/journal.pone.0021902)
Supplement: Table S39 — Subgroup assignment is not associated with laboratory in which the samples were processed. (DOC) [file pone.0021902.s046.doc]

## Online Supporting Information Table S39: Subgroup Association with Sample Processing Laboratory

(donor-level variable)

|  | Subgroup based on K-means clustering (k=3) of 1079 probesets | | |  |
| --- | --- | --- | --- | --- |
| CRO | Subgroup X | Subgroup Y | Subgroup Z | Total |
| AARI | 6 (20.0%) | 11 (17.2%) | 14 (19.4%) | 31 |
| DeCode | 7 (23.3%) | 18 (28.1%) | 18 (25.0%) | 43 |
| ICON-D | 6 (20.0%) | 14 (21.9%) | 20 (27.8%) | 40 |
| ICON-F | 11 (36.7%) | 21 (32.8%) | 20 (27.8%) | 52 |
| Total | 30 | 64 | 72 | 166 |

p-value = 0.95

Conclusion: No evidence for association between CRO and Subgroup assignments.
