# Supplementary material for: Pathways Activated during Human Asthma Exacerbation as Revealed by Gene Expression Patterns in Blood
Source: PLoS One. 2011 Jul 14;6(7):e21902. doi: 10.1371/journal.pone.0021902 (PMC3136489; doi:10.1371/journal.pone.0021902)
Supplement: Table S41 — Subgroup assignment is not associated with atopy status. (DOC) [file pone.0021902.s048.doc]

## Online Supporting Information Table S41: Subgroup Association with Atopy Status

(donor-level variable)

|  | Subgroup based on K-means clustering (k=3) of 1079 probesets | | |  |
| --- | --- | --- | --- | --- |
| Atopy Status | Subgroup X | Subgroup Y | Subgroup Z | Total |
| Atopic | 19 (63.3%) | 41 (64.1%) | 54 (75.0%) | 114 |
| Non-atopic | 7 (23.3%) | 18 (28.1%) | 11 (15.3%) | 36 |
| Unknown | 4 (13.3%) | 5 (7.8%) | 7 (9.7%) | 16 |
| Total | 30 | 64 | 70 | 166 |

p-value = 0.40

Conclusion: No evidence of association between atopy status and Subgroup assignments.
