# Supplementary material for: Pathways Activated during Human Asthma Exacerbation as Revealed by Gene Expression Patterns in Blood
Source: PLoS One. 2011 Jul 14;6(7):e21902. doi: 10.1371/journal.pone.0021902 (PMC3136489; doi:10.1371/journal.pone.0021902)
Supplement: Table S42 — Subgroup assignment is not associated with fasting status. (DOC) [file pone.0021902.s049.doc]

## Online Supporting Information Table S42: Subgroup Association with Fasting Status

(visit-level variable)

|  | Subgroup based on K-means clustering (k=3) of 1079 probesets | | |  |
| --- | --- | --- | --- | --- |
| Fasting status | Subgroup X | Subgroup Y | Subgroup Z | Total |
| Unknown | 0 (0.0%) | 9 (14.6%) | 2 (2.8%) | 11 |
| Fasting/Yes | 9 (30.0%) | 14 (21.9%) | 21 (29.2%) | 44 |
| Non-fasting/No | 21 (70.0%) | 41 (64.1%) | 49 (68.1%) | 111 |
| Total | 30 | 64 | 72 | 166 |

p-value = 0.042 (would be better with exact test p-values)

Conclusion: Evidence of association between fasting status and Subgroup assignments.
