# Supplementary material for: Pathways Activated during Human Asthma Exacerbation as Revealed by Gene Expression Patterns in Blood
Source: PLoS One. 2011 Jul 14;6(7):e21902. doi: 10.1371/journal.pone.0021902 (PMC3136489; doi:10.1371/journal.pone.0021902)
Supplement: Table S43 — Subgroup assignment is not associated with IgE titers. (DOC) [file pone.0021902.s050.doc]

## Online Supporting Information Table S43: Subgroup Association with IgE

|  | **Subgroup based on K-means clustering (k=3) of 1079 probesets** | | |
| --- | --- | --- | --- |
|  | **Exacerbation** | | |
| **Statistic** | **Subgroup X** | **Subgroup Y** | **Subgroup Z** |
| N | 10 | 21 | 24 |
| Mean | 317.1 | 348.1 | 113.7 |
| Median | 38.0 | 126.0 | 64.5 |
| S.D. | 541.7 | 572.5 | 174.4 |
| CV | 170.8 | 164.4 | 153.5 |
| 5^th^ percentile | 10 | 40 | 10 |
| 95^th^ percentile | 1500 | 992 | 326 |
| Missing values | 20 | 43 | 48 |

Conclusions:

1. Poorly behaved variable from a statistical point of view – very skewed, lots of variability.
2. Large amount of missing data makes if questionable how much we’ll get out of this.
