# Supplementary material for: Pathways Activated during Human Asthma Exacerbation as Revealed by Gene Expression Patterns in Blood
Source: PLoS One. 2011 Jul 14;6(7):e21902. doi: 10.1371/journal.pone.0021902 (PMC3136489; doi:10.1371/journal.pone.0021902)
Supplement: Table S44 — Subgroups assignment is not associated with medical history of acid reflux. (DOC) [file pone.0021902.s051.doc]

## Online Supporting Information Table S44: Subgroup Association with Medical History of Acid Reflux

(donor-level variable)

|  | Subgroup based on K-means clustering (k=3) of 1079 probesets | | |  |
| --- | --- | --- | --- | --- |
| Any history? | Subgroup X | Subgroup Y | Subgroup Z | Total |
| No | 20 (66.7%) | 39 (60.9%) | 45 (62.5%) | 104 |
| Yes | 10 (33.3%) | 25 (39.1%) | 27 (37.5%) | 62 |
| Total | 30 | 64 | 72 | 166 |

p-value = 0.87

Conclusion: No evidence for association between medical history of acid reflux and Subgroup assignments.
