# Supplementary material for: Pathways Activated during Human Asthma Exacerbation as Revealed by Gene Expression Patterns in Blood
Source: PLoS One. 2011 Jul 14;6(7):e21902. doi: 10.1371/journal.pone.0021902 (PMC3136489; doi:10.1371/journal.pone.0021902)
Supplement: Table S45 — Association between BMI (at screening) and subgroup assignment. (DOC) [file pone.0021902.s052.doc]

## Online Supporting Information Table S45: Association with BMI (based on screening height and weight)

(donor-level variable)

For the days after quiet visits and BMI analyses, the p-value is from a one-way analysis of variance (ANOVA).

|  | **Cluster based on 1079 probeset clustering** | | |
| --- | --- | --- | --- |
| **Statistic** | **Cluster X** | **Cluster Y** | **Cluster Z** |
| N | 30 | 64 | 53 |
| Mean | 28.4 | 32.4 | 30.2 |
| Median | 28.0 | 32.5 | 29.3 |
| S.D. | 6.2 | 6.6 | 6.2 |
| CV | 22.0 | 20.5 | 20.6 |

p-value from test for differences of means among clusters = 0.015

Conclusion: Some evidence of differences among clusters in mean BMI. Mean BMI is statistically significantly lower (p=0.006) in cluster 1 than cluster 2, and is statistically suggestively lower (p=0.0501) in cluster 3 than cluster 2.
