# Supplementary material for: Pathways Activated during Human Asthma Exacerbation as Revealed by Gene Expression Patterns in Blood
Source: PLoS One. 2011 Jul 14;6(7):e21902. doi: 10.1371/journal.pone.0021902 (PMC3136489; doi:10.1371/journal.pone.0021902)
Supplement: Table S46 — Association between subgroup assignment and days since quiet visit. (DOC) [file pone.0021902.s053.doc]

## Online Supporting Information Table S46: Subgroup Association with Days Since Quiet Visit

|  | **Subgroup based on 1079 probeset clustering** | | |
| --- | --- | --- | --- |
| **Statistic** | **Subgroup X** | **Subgroup Y** | **Subgroup Z** |
| N | 30 | 64 | 71 |
| Mean | 48.4 | 62.7 | 79.6 |
| Median | 40.5 | 40 | 69 |
| S.D. | 45.3 | 55.3 | 64.0 |
| CV | 93.6 | 88.3 | 80.5 |
| 5^th^ percentile | 9 | 7 | 11 |
| 95^th^ percentile | 91 | 181 | 211 |
| Missing values | 0 | 0 | 1 |

p-value from test for differences of means among Subgroups = 0.03

Conclusions:

1. Large variability, some skewing, not many missing observations. May be a variable with some statistical utility.
2. Interesting differences between nodes in mean number of days since quiet visit. Exacerbation visits occur sooner after a quiet in some nodes than in others. Statistically significant difference between Subgroups 1 and 3 (p=0.014), difference between Subgroups 2 and 3 is statistically suggestive (p=0.091).
