# Supplementary material for: Pathways Activated during Human Asthma Exacerbation as Revealed by Gene Expression Patterns in Blood
Source: PLoS One. 2011 Jul 14;6(7):e21902. doi: 10.1371/journal.pone.0021902 (PMC3136489; doi:10.1371/journal.pone.0021902)
Supplement: Text S1 — Extensive details of the study are provided in this 101 page document. (DOC) [file pone.0021902.s054.doc]

**Supporting Information**

**Table of Contents**

[Biological Samples 4](#__RefHeading___Toc296618726)

[Sample Preparation 4](#__RefHeading___Toc296618727)

[Table S1: Post-CPT Purification Monocyte and Lymphocyte Percent in Quiet and Exacerbation Visits. 4](#__RefHeading___Toc296618728)

[Table S2: Quality Control Criteria for Inclusion of GeneChip in Analysis 5](#__RefHeading___Toc296618729)

[Determination of Gene Expression Levels Using Affymetrix GeneChip Platform: 5](#__RefHeading___Toc296618730)

[Statistical Methods 6](#__RefHeading___Toc296618731)

[Initial Filtering and Analysis Dataset Construction 6](#__RefHeading___Toc296618732)

[ANCOVA Methods 6](#__RefHeading___Toc296618733)

[Quiet Samples Per subject 6](#__RefHeading___Toc296618734)

[Figure S1: Distribution of 384 Quiet Samples from 118 Subjects 7](#__RefHeading___Toc296618735)

[Covariates 8](#__RefHeading___Toc296618736)

[ANCOVA 8](#__RefHeading___Toc296618737)

[Analysis of Concordance of GeneChip and Taqman Platforms 9](#__RefHeading___Toc296618738)

[Figure S2: Concordance of Results Using GeneChip and Taqman Platforms 11](#__RefHeading___Toc296618739)

[Identification of *Exacerbation* Subgroups X, Y and Z. 11](#__RefHeading___Toc296618740)

[Subgrouping of *Exacerbation* Samples by K-means Clustering 11](#__RefHeading___Toc296618741)

[Assessment of Subgroup Robustness by Simulation 12](#__RefHeading___Toc296618742)

[Figure S3. Silhouette Statistic for K = 2 through K = 8 Clusters 12](#__RefHeading___Toc296618743)

[Figure S4. Robustness statistics for K = 2 through K = 8 14](#__RefHeading___Toc296618744)

[Ordering of Samples and Probe Sets by Spectral Biclustering 14](#__RefHeading___Toc296618745)

[Figure S5: Visual Representation of Differences Between Gene Expression Levels in Each of 166 Exacerbation Samples and the Average of Quiet Samples from the Same Patient. 15](#__RefHeading___Toc296618746)

[Real Time (TaqMan) PCR Methods 15](#__RefHeading___Toc296618747)

[Purification of RNA and Conversion to cDNA 15](#__RefHeading___Toc296618748)

[Taqman Gene Expression Assays 15](#__RefHeading___Toc296618749)

[Table S3: Genes Analyzed by Taqman. 16](#__RefHeading___Toc296618750)

[Generation of Standard Curve RNA/cDNA 17](#__RefHeading___Toc296618751)

[Identification of Appropriate Endogenous Control 17](#__RefHeading___Toc296618752)

[Information On Enrolled Subjects 18](#__RefHeading___Toc296618753)

[Demographics and Asthma Status at Baseline 18](#__RefHeading___Toc296618754)

[Study Population 18](#__RefHeading___Toc296618755)

[Table S4: Demographic and Baseline Characteristics by Asthma Severity 19](#__RefHeading___Toc296618756)

[Patient and physician agreement of asthma control 20](#__RefHeading___Toc296618757)

[Table S5: Global Assessment of Asthma Control by the Subject and by the Investigator at Screening 21](#__RefHeading___Toc296618758)

[Asthma Healthcare Resource Use 21](#__RefHeading___Toc296618759)

[Table S6: Reported Asthma Healthcare Resource Use Before Enrollment 22](#__RefHeading___Toc296618760)

[Atopic status 23](#__RefHeading___Toc296618761)

[Table S7: Atopy Status at Screening 23](#__RefHeading___Toc296618762)

[Tobacco use 24](#__RefHeading___Toc296618763)

[Table S8: Body Mass Index and Gastrointestinal Reflux Disease: 25](#__RefHeading___Toc296618764)

[Table S9: History of Reflux Disease 26](#__RefHeading___Toc296618765)

[Changes in asthma severity over 12 months of study 26](#__RefHeading___Toc296618766)

[Information Table S10: Subjects with a Change in Asthma Severity by Visit 27](#__RefHeading___Toc296618767)

[Concomitant Anti-asthmatic Medications 27](#__RefHeading___Toc296618768)

[Table S11: Number (%) of Subjects Who Used Concomitant Anti-asthmatic Medications by Asthma Severity 27](#__RefHeading___Toc296618769)

[Geographical differences in the use of asthma medications 28](#__RefHeading___Toc296618770)

[Table S12: Number (%) of Subjects Who Used Concomitant Anti-asthmatic Medications by Country 29](#__RefHeading___Toc296618771)

[Asthma Healthcare Resource Use During Study 30](#__RefHeading___Toc296618772)

[Table S13: Reported Asthma Healthcare Resource Use During the Study (number of events reported) 30](#__RefHeading___Toc296618773)

[Asthma Precipitating or Aggravating Factors During the Study 31](#__RefHeading___Toc296618774)

[Table S14: Asthma Precipitating or Aggravating Factors by Visit 31](#__RefHeading___Toc296618775)

[Adverse Events During the Study 32](#__RefHeading___Toc296618776)

[Table S15: Number (%) of Subjects Experiencing Adverse Events 33](#__RefHeading___Toc296618777)

[Table S16: Most Common (≥10% of Subjects in Any Severity Group) Respiratory Adverse Events, Number (%) of Subjects 34](#__RefHeading___Toc296618778)

[Pulmonary Function Tests 35](#__RefHeading___Toc296618779)

[Table S17: Mean FEV1 (% Predicted) at Scheduled Non-Exacerbation Visits 35](#__RefHeading___Toc296618780)

[Figure S6A: Relative FDR p-value Obtained From ANCOVA On Subgroup X Samples Using Only Exacerbation Samples with Corresponding Follow-up Sample 36](#__RefHeading___Toc296618781)

[Figure S6B: Relative FDR p-value Obtained From ANCOVA On Subgroup Y Samples Using Only Exacerbation Samples with Corresponding Follow-up Sample 37](#__RefHeading___Toc296618782)

[Figure S6C: Relative FDR p-value Obtained From ANCOVA On Subgroup Z Samples Using Only Exacerbation Samples with Corresponding Follow-up Sample. 38](#__RefHeading___Toc296618783)

[List by Subgroup of Probesets (Genes) Associated with Exacerbation 39](#__RefHeading___Toc296618784)

[Table S18A: ANCOVA Results Subgroup X 39](#__RefHeading___Toc296618785)

[Table S18B: ANCOVA Results Subgroup Y Samples 64](#__RefHeading___Toc296618786)

[Table S18C: ANCOVA Results Subgroup Z Samples 77](#__RefHeading___Toc296618787)

[Table S19: IL15 Pathway Genes Associated with Exacerbation in Subgroup X 84](#__RefHeading___Toc296618788)

[Associations Between Covariates and Subgroup Assignments 87](#__RefHeading___Toc296618789)

[Association between Exacerbation Onset and Days to Exacerbation Sample Collection 87](#__RefHeading___Toc296618790)

[Figure S7. Subgroup Assignment and Days Between Exacerbation Onset and Exacerbation Sample Collection 87](#__RefHeading___Toc296618791)

[Association with Respiratory Function Measurements 88](#__RefHeading___Toc296618792)

[Table S20: Subgroup Association with FEV1 (predicted) 89](#__RefHeading___Toc296618793)

[Table S21: Subgroup Association with FEV1 (predicted) change from baseline 89](#__RefHeading___Toc296618794)

[Table S22: Subgroup Association with FVC (predicted) 90](#__RefHeading___Toc296618795)

[Table S23: Subgroup Association with FVC (predicted) change from baseline 90](#__RefHeading___Toc296618796)

[Table S24: Subgroup Association with FEF 25-75% (predicted) 91](#__RefHeading___Toc296618797)

[Table S25: Subgroup Association with FEF 25-75% (predicted) change from baseline 91](#__RefHeading___Toc296618798)

[Table S26: Subgroup Association with PEF (predicted) 92](#__RefHeading___Toc296618799)

[Table S27: Subgroup Association with PEF (predicted) change from baseline 92](#__RefHeading___Toc296618800)

[Table S28: Subgroup Association with Relevant Respiratory Infection 92](#__RefHeading___Toc296618801)

[Table S29: Subgroup Association with Disease Severity 93](#__RefHeading___Toc296618802)

[Association with Use of Medication 93](#__RefHeading___Toc296618803)

[Table S30: Subgroup Association with Use of Medication: Systemic Corticosteroids 94](#__RefHeading___Toc296618804)

[Table S31: Subgroup Association with Use of Medication: Inhaled Corticosteroids 94](#__RefHeading___Toc296618805)

[Table S32: Subgroup Association with Use of Medication: Association with Use of Intranasal Corticosteroids 94](#__RefHeading___Toc296618806)

[Table S33: Subgroup Association with Use of Medication: Leukotriene Antagonists 95](#__RefHeading___Toc296618807)

[Table S34: Subgroup Association with Use of Medication: Any GI Non-Study Med Use 95](#__RefHeading___Toc296618808)

[Table S35: Subgroup Association with Use of Medication: Any PPI Non-study Med Use 95](#__RefHeading___Toc296618809)

[Table S36: Subgroup Association with Use of Medication: Association with Any Histamine H2 Antagonist Non-study Med Use 96](#__RefHeading___Toc296618810)

[Table S37: Subgroup Association with Sex 96](#__RefHeading___Toc296618811)

[Table S38: Subgroup Association with Race 96](#__RefHeading___Toc296618812)

[Table S39: Subgroup Association with Sample Processing Laboratory 97](#__RefHeading___Toc296618813)

[Table S40: Subgroup Association with Country 98](#__RefHeading___Toc296618814)

[Table S41: Subgroup Association with Atopy Status 98](#__RefHeading___Toc296618815)

[Table S42: Subgroup Association with Fasting Status 98](#__RefHeading___Toc296618816)

[Table S43: Subgroup Association with IgE 99](#__RefHeading___Toc296618817)

[Table S44: Subgroup Association with Medical History of Acid Reflux 99](#__RefHeading___Toc296618818)

[Table S45: Association with BMI (based on screening height and weight) 100](#__RefHeading___Toc296618819)

[Table S46: Subgroup Association with Days Since Quiet Visit 100](#__RefHeading___Toc296618820)

[References 101](#__RefHeading___Toc296618821)

# Biological Samples

## Sample Preparation

Blood samples for mRNA expression studies were obtained at each visit. The frequency of these sample collections was intended to provide a sufficient number of longitudinal samples to evaluate intra-subject variability, while also attempting to minimize any interference with the subjects’ routine visit schedule.

PBMCs from asthma subjects were isolated from whole blood samples (8 ml x 6 tubes) collected into cell purification tubes (Becton Dickinson, Franklin Lakes, NJ) according to the manufacturer’s recommendations. All asthma samples were shipped at room temperature in a temperature controlled box overnight from the clinical site, cell differential counts taken, peripheral blood mononuclear cells (PBMCs) purified according to CPT manufacturer instructions and cell pellets stored at –80^o^C pending RNA purification.

## Table S1: Post-CPT Purification Monocyte and Lymphocyte Percent in Quiet and Exacerbation Visits.

|  | Quiet | | | Exacerbation | | |
| --- | --- | --- | --- | --- | --- | --- |
|  | mean | s.d. | n | mean | s.d. | n |
| Monocyte percent | 20.97 | 12.49 | 309 | 26.14 | 13.13 | 134 |
|  |  |  |  |  |  |  |
| Lymphocyte percent | 64.79 | 18.52 | 309 | 60.02 | 17.53 | 134 |

RLT lysis buffer (with 0.1% β-mercaptoethanol) was added to frozen pellets, RNA isolated using RNeasy Mini Kit (Catalog #74104, Qiagen, Valencia, CA) and DNase treated (Qiagen RNase-free DNase Kit Catalog #79254). Eluted RNA was quantified using a Spectramax96 well plate UV reader (Molecular Devices, Sunnyvale, CA, USA) monitoring A260/280 OD values. The quality of each RNA sample was assessed by the integrity of the 28S and 18S peaks by capillary electrophoresis alongside an RNA molecular weight ladder on the Agilent 2100 bioanalyzer (Agilent Technologies, Palo Alto, CA, USA). RNA was quantified using Spectramax96 (Molecular Devices, Sunnyvale, CA).

## Table S2: Quality Control Criteria for Inclusion of GeneChip in Analysis

| 1 | Defect on visual inspection |  |
| --- | --- | --- |
| 2 | Bactin Gapdh Freq Avg Exp | > 0.6 |
| 3 | Genechip Raw Q Exp | < 7 |
| 4 | Qc P Prob Freq Exp | < 20 |
| 5 | Qc P Prob Avg Diff Exp | < 205 |
| 6 | Qc Sensitivity Exp | < 6.1 |
| 7 | Scale Factor Exp | < 4 and > 0.25 |
|  |  |  |
| 1 | Defect on visual inspection: Patterns in chip fluorescence visible after the chip has been run that reveal scratches, uneven staining or other defects. |  |
| 2 | Ratio of signal portion of the gene. A measure of the integrity of the RNA sample. |  |
| 3 | Raw Q: measure of the noise level of the array, it is the degree of pixel-to-pixel variation among the probe cells used to calculate the background. |  |
| 4 | QCP probability average difference: signal value for which there is a 70% probability of a Present call. |  |
| 5 | QCP probability frequency: QCP probability average difference expressed in ppm units. |  |
| 6 | Chip sensitivity: concentration level, in ppm, at which there is a 70% probability of obtaining a Present call. |  |
| 7 | Scale factor: the value required to obtain a trimmed mean intensity indicated by the target value. For all data in this study, the target value was set to a value of 100 and the scale factor was determined by dividing the trimmed mean of all probe sets by the target value. |  |

# Determination of Gene Expression Levels Using Affymetrix GeneChip Platform:

Labeled target for oligonucleotide arrays were prepared using 2 μg of total RNA according to the protocol provided by Affymetrix (Santa Clara CA.). Biotinylated cRNA was hybridized to the HG-U133A Affymetrix GeneChip Array®. Raw intensity values were processed using Affymetrix MAS 5.0 software, which calculated signal expression levels and present/absent calls for each probe set. Quality control acceptance criteria are shown in Table S1. Samples that did not pass these quality control criteria were re-run, and samples that failed twice were excluded from analyses. A sample was considered evaluable if GeneChip quality control acceptance criteria were met and paired *exacerbation* and *quiet* visit samples were available from the same subject. Of the 22283 probe sets on the U133A array, only the 9696 probe sets that met the following two criteria were included in statistical analyses described below: a) detection as present in at least 10% of samples, and b) signal of at least 50 in at least 10% of samples.

# Statistical Methods

## Initial Filtering and Analysis Dataset Construction

QC criteria were applied to all chips (Supplemental Text 1), and chips that failed QC were not included in the analyses.

Prior to analysis, a filter was applied to remove from further consideration Affymetrix U133a probe sets with minimal evidence of expression. Probe sets were retained for analysis if they were called “Present” by the Affymetix MAS5 software in at least 10% of samples and if at least 10% of the samples had a MAS5 signal greater than 50. A total of 9696 probe sets passed this filter.

Relevant clinical data were extracted from a clinical database, sample-level data were extracted from a LIMS system, and microarray expression data were extracted from an expression database. The data were merged and re-formatted as necessary using SAS version 9.1.

# ANCOVA Methods

## Quiet Samples Per subject

The percent of subjects with 1, 2, 3, 4 or 5 quiet samples is shown in Figure S1. There was more than 1 *quiet* sample analyzed for 96% of the 118 subjects. Repeated-measures analysis of covariance (ANCOVA) methods were used to compare mean expression levels among groups of primary interest. Separate ANCOVAs were performed for each probe set that passed the nominal filter described above. Log_2_-transformed MAS5 signal was the dependent variable in all ANCOVAs.

## Figure S1: Distribution of 384 Quiet Samples from 118 Subjects

Three or more *quiet* samples were analyzed from the majority (84%) of the 118 subjects with *exacerbation* samples, with 3 samples from 38% of subjects, 4 samples from 40% of subjects, and 5 samples analyzed from 6% of subjects. Two quiet samples were analyzed from 12% of the subjects, and only 1 quiet sample was available for the remaining 3%.

## Covariates

Covariates thought to potentially influence expression levels were included in the ANCOVA models to reduce error variance and minimize confounding of their effects with the effects of primary interest. Sex, race (Asian, black, white), age category (18-39, 40-59, 60-83), baseline severity (mild, moderate, severe persistent by NIH guidelines) and initial processing lab (a four-level variable, completely confounded with geographic location) were subject-level covariates included in all ANCOVA models. Visit type (e.g. *quiet*, *exacerbation*, *follow-up*), degree of corticosteroid use (a 5-level variable), leukotriene receptor antagonist use (y/n), β−actin to GAPDH 5’3’ ratios (a measure of RNA quality), and monocyte/leucocyte ratio were visit- or sample-level variables included in all models. For analyses that compared expression levels in *quiet* visits versus each of the three *exacerbation* subgroups, visit type was replaced with a four-level variable (*quiet*, subgroup X, subgroup Y, subgroup Z).

## ANCOVA

Multiple visits per subject were included in the analyses; therefore all models included a random subject-level compound symmetry term to account for potential intra-subject correlation across visits.

In some analyses, particular pair-wise comparisons (e.g., *quiet* versus subgroup X) were of interest in addition to the overall ANCOVA F-tests. Pair-wise comparisons were made using two-sample t-tests, with error terms for the t-statistics based on the appropriate error term from the ANCOVA.

Adjustment of raw p-values to account for the substantial multiple testing problem was done using the Benjamini and Hochberg (1995) “false discovery rate” (FDR) approach as implemented in SAS PROC MULTTEST. For each hypothesis tested, the adjustment was done based on the set of 9696 raw p-values generated for the tested probe sets. In the ANCOVAs that compared expression levels in *quiet* samples to those in the three *exacerbation* subgroups, the raw p-values and adjusted p-values are understood to be biased (too liberal), since the subgroups were defined based on unsupervised clustering that acted to maximize differences among the subgroups. The degree of bias is unknown; hence the p-values (raw or adjusted) should be interpreted as being useful primarily for ranking the probe sets in terms of strength of evidence for differentiation between *quiet* and *exacerbation* expression levels.

All analyses were performed using SAS version 9.1.

# Analysis of Concordance of GeneChip and Taqman Platforms

We assessed concordance between results from the U133A GeneChip and results from Taqman PCR by designing at two Taqman Low Density Arrays (TLDAs) that interrogated 198 genes and comparing the differences in gene expression observed between paired samples as assessed using each of these platforms [1]. This analysis was done using quiet asthma samples that were included in the study reported here, but the comparator samples were from normal healthy volunteers. The reason for the choice of healthy volunteer (rather than exacerbation) comparator samples was, in advance of an independent clinical study using an investigative drug, to confirm by Taqman selected biomarkers of asthma in the blood that had been identified through a GeneChip-based comparison of quiet asthma and healthy volunteer.

The TLDAs that were used to assay quiet asthma samples from this study were designed to include two classical reference genes were included (18S and GAPDH) and in addition 2 other genes, GUSB and PGK1, commonly used as reference genes that had performed well as normalizer in another PBMC study we had performed previously. In addition, we selected as a normalizer ZNF592, a gene identified through searches of expression profile databases as having invariant expression levels in multiple studies involving different types of cells and tissues. We have used ZNF592 in many of our studies involving skin, colon and PBMC and confirmed its utility as a normalizer gene due to very low variability in expression level. Previous analyses characterized the behavior of the TLDA housekeeper genes and combinations of housekeeper genes in replicate samples of a universal RNA control. These analyses indicated that delta Cts based on the mean of the four housekeeper genes (18S excluded) provided an average reduction in measurement variability compared to raw Cts. To assess the performance of the housekeeper genes with respect to adjusting for technical measurement variability in the test samples, delta Cts were calculated for each of the duplicates run for the samples and compared and the delta Ct method of normalization proved highly effective.

To compare results from TLDA and GeneChip, a subset of the samples (previously analyzed by GeneChip) were re-assayed by TLDA. To comprehensively compare the performance of the two platforms and ensure the diversity in our sample set, we selected for analysis the top pairs of asthma and healthy samples, with the constraint that no single sample could be included in more than one pair. Each of these samples was then assayed in duplicate on TLDAs, and differential expression (absolute log2-fold change) for 168 genes interrogated by TLDA, in each sample pair was calculated from the TLDA data and compared to the absolute log2-fold change observed by GeneChip.

The overall Pearson correlation coefficient (for the two platform measurements of expression differences) was 0.859. The results of this comparative analysis are shown below in Figure S1, and we have observed similar platform concordance in a second comparative analysis using unrelated samples [1]. Because of the remarkably tight concordance between TLDA and GeneChip platforms observed in both studies, we did not perform the analysis with exacerbation samples. Our results indicating close concordance between results obtained using oligonucleotide array and PCR based platforms is consistent with other reports that have compared results across platforms [2-4]

## Figure S2: Concordance of Results Using GeneChip and Taqman Platforms


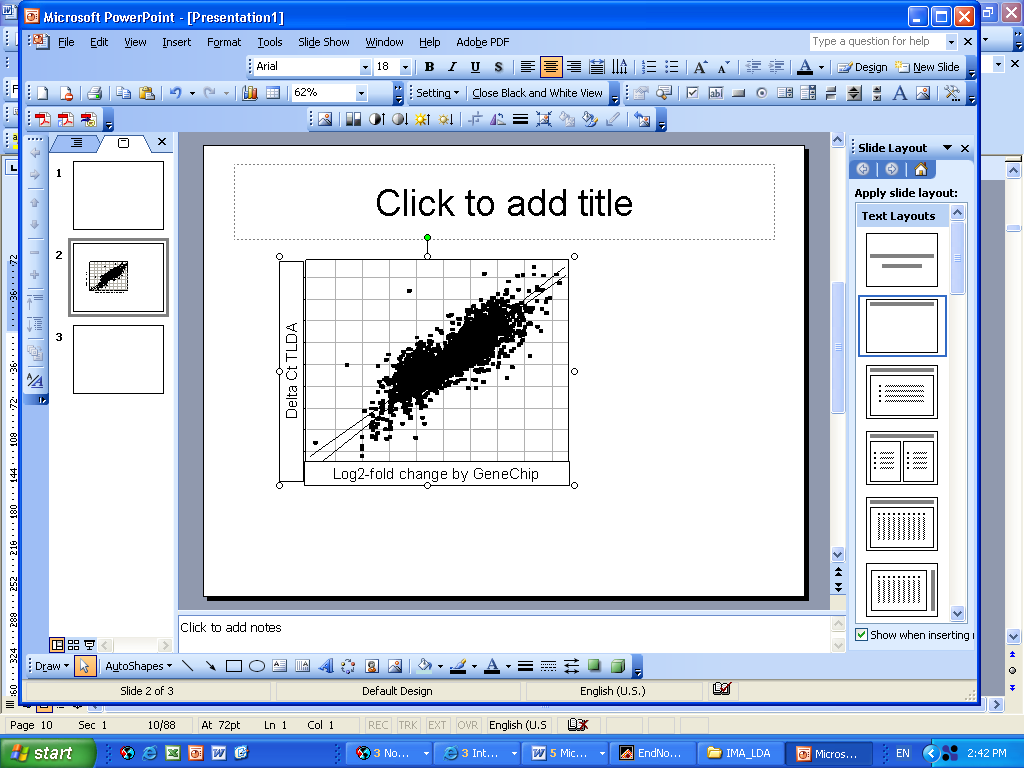


A strong correlation was observed between expression levels as measured by Affymetrix U133A GeneChip and as measured by TaqMan®  Low Density Array. Differences in expression between paired samples as observed in the two platforms are shown. Signal sample pair differences (log 2 from GeneChip) are shown on the X axis, and delta CT sample pair differences (from TaqMan®) on the Y axis.

# Identification of *Exacerbation* Subgroups X, Y and Z.

## Subgrouping of *Exacerbation* Samples by K-means Clustering

Evidence of heterogeneity among *exacerbation*-associated gene signatures was evaluated using K-means clustering (which groups samples according to degree of similarity in gene expression profile). The *exacerbation*-to-average *quiet* log2-ratio profiles of donors were clustered by K-means clustering [5] into K=3 groups. K-means clustering was executed in the R software package (version 2.1.1; [www.r-project.org](http://www.r-project.org/)). Initial cluster centers were selected randomly from the profiles in the dataset.

## Assessment of Subgroup Robustness by Simulation

Figure S3 below shows silhouette statistics for K=2, 3, 4, and 8 clusters. As expected, silhouette decreases with increasing K. A reasonable method for assessing the silhouette statistic is to find the number of clusters where there is an “elbow” in the slope of the SW curve, indicating that further increases in K have diminishing benefits in distinguishing distinct groups. This elbow occurred at K=3.

## Figure S3. Silhouette Statistic for K = 2 through K = 8 Clusters


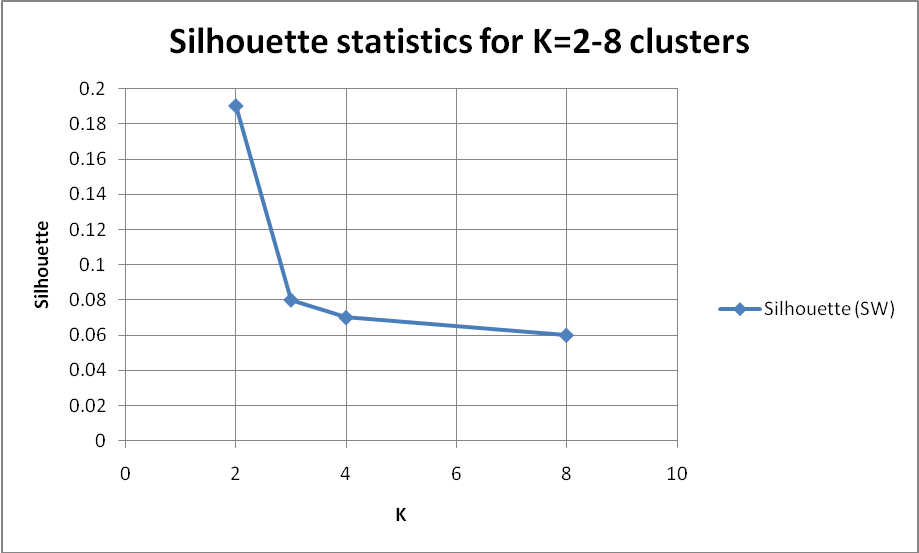


The silhouette statistic for K = 2, K = 3, K = 4 and K = 8 is shown.

We also assessed the robustness of K-means clusters of Samples using both the **silhouette statistic (SW) [7] and a simulation-based robustness index ( R),** similar to the approach of McShane et al.[8]. **Iterative K-means cluster analyses were run for K = 2, 3, 4, and 8 using the log-ratios metric described above.** For the robustness index calculation, Gaussian random noise with zero mean and realistic amplitude (a standard deviation of 0.3) was computationally added to the observed log-ratios to simulate biological replication. For each of 100 realizations of the noisy data, K-means clustering was executed as described above, and the co-clustering of all donor pairs was recorded. The resulting co-clustering matrix was then divided by the number of realizations (100) to yield a symmetrical matrix of cluster co-occurrence fractions for every sample-pair in the dataset. The average co-occurrence fraction of each cluster $\bar{f}_{i}$was computed as the average co-occurrence fraction of all the distinct sample-pairs in the cluster:

$$\bar{f}_{i}=\frac{\sum_{j=1}^{m_{i}} f_{j}}{m_{i}}$$

Where *j* indexes over all *m_i_* distinct pairs of Samples in cluster *i*. The cluster robustness index R was then calculated as the weighted average value of $\bar{f}_{i}$for the K-means clusters:

This index R reflects the cohesiveness of the K-means clusters in the presence of simulated experimental noise. A value R=1 indicated that all pairs of Samples that clustered together in the observed data, also clustered together in all simulations. A value of R=0 indicated that none of the Sample-pairs that clustered together in the observed data could be re-confirmed in the presence of simulated experimental noise.

**In interim analyses there was clear and robust separation into K=2 clusters (SW=0.19, R=0.998) or K=3 clusters (SW=0.08, R=0.88).  Beyond K=3 clusters, the SW and R measures declined, indicating little support for more than three subgroups.**

Subjects were therefore assigned to one of three sub-groups, designated sub-groups X, Y and Z, by K-means clustering. K-means clustering assigned 30 samples (18%) to subgroup X, 64 (38%) to subgroup Y, and 72 (43%) to subgroup Z.

ANCOVAs were run for all 9696 probe sets to identify cluster-specific differences in *quiet* and *exacerbation* sample expression levels that might have been masked by subject heterogeneity when all 166 Samples were analyzed together. Within a sub-group, probe sets with differences between *quiet* and *exacerbation* levels that had FDR≤0.05 and average absolute fold difference >1.2 were classified as being associated with *exacerbation* in that sub-group.

## Figure S4. Robustness statistics for K = 2 through K = 8


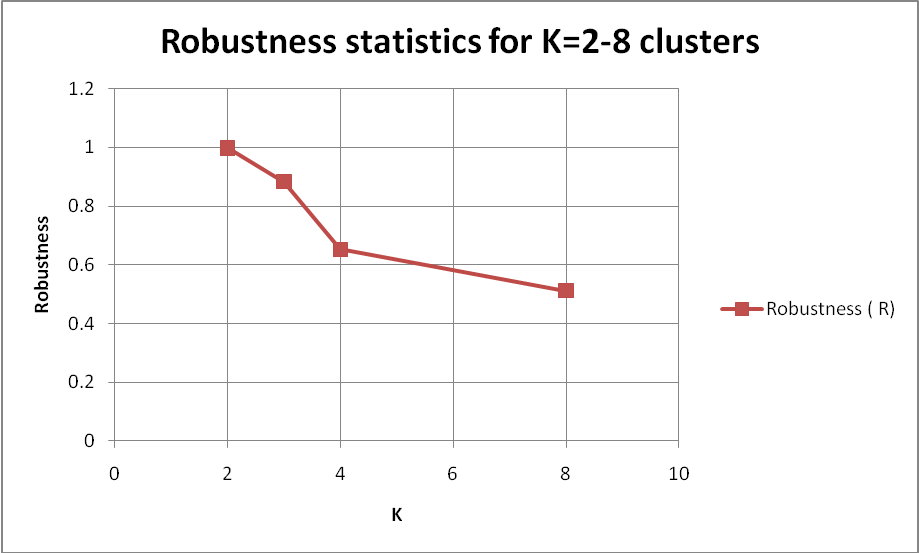


The larger drop in robustness statistic ( R) from K=3 to K=4, compared to either the K=1-2 or K=4-8 drops, is shown indicating that increasing from 3 to 4 clusters markedly reduced the robustness of the cluster assignments to simulated experimental noise.

Combining these observations with the imperative to use the simplest model that is consistent with the data, we selected K=3.

## Ordering of Samples and Probe Sets by Spectral Biclustering

For display purposes, we used a spectral biclustering method [6] to order Samples and probe sets within K-means clusters by the singular-value decomposition of the interactions matrix **Figure S2**. Spectral biclustering was executed in the MATLAB software package (version 7.1.0.246; [www.mathworks.com](http://www.mathworks.com/) ).

## Figure S5: Visual Representation of Differences Between Gene Expression Levels in Each of 166 Exacerbation Samples and the Average of Quiet Samples from the Same Patient.


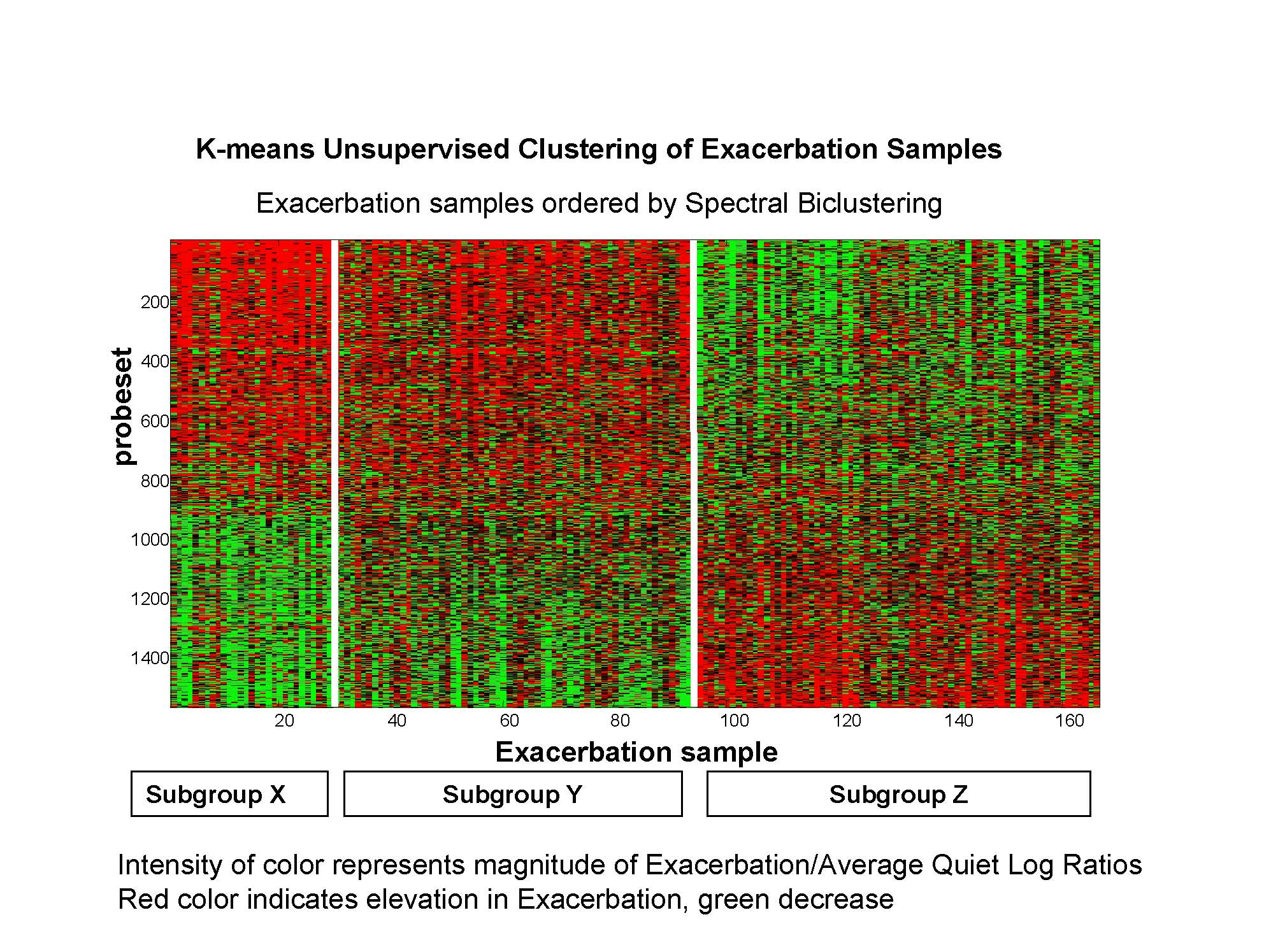


Color representation of differences between gene expression levels in each of 166 *exacerbation* samples and the average of *quiet* samples from the same patient. Intensity of color indicates magnitude of *exacerbation*/average *quiet* log ratios. Red color indicates elevation in expression in *exacerbation*, and green represents a decrease

# Real Time (TaqMan) PCR Methods

## Purification of RNA and Conversion to cDNA

Conversion of 2 μg of total RNA from the above preparations to cDNA was accomplished using the Applied Biosystems High Capacity cDNA Archive Kit (Catalog #4322171) by following the manufacturer’s instructions.

## Taqman Gene Expression Assays

Applied Biosystems (ABI) Assays-on-Demand (AOD) gene specific primer-probe pairs are pre-validated, QC tested and optimized for use on any ABI PRISM sequence detection system. Real-time quantitative gene expression assay kits were ordered through the Applied Biosystems online AOD product catalog for targets listed in Table S2 and the endogenous control gene ZNF592 (ABI catalog # Hs00206029_m1). Using the AOD product insert volume recommendations, a master mix was prepared using Taqman Universal PCR Master Mix (Catalog #4304437) and aliquoted into a 96 well plate (ABI Catalog #N801-0560 and caps #N801-0935) for a final volume of 50 μl/well. Duplicate wells for serially diluted standards and cDNA samples (50 ng/well) were assayed on an ABI PRISM 7700 Sequence detector (Sequence Detector Software v1.7) using universal thermal cycling conditions of 50^0^C for 2 minutes, 95^0^C for 10 minutes and 40 cycles of 95^0^C for 15 seconds, 60^0^C for 1 minute.

Relative quantification of RNA transcript levels was performed following the guidelines described in ABI PRISM 7700 Sequence Detection System User Bulletin #2 using the relative standard curve method. Specifically, standard curves are calculated for target standards and endogenous control, input values determined for target and endogenous control using standard curves’ slope and y-intercept, target input values are normalized to endogenous control. Fold change is calculated using the 50 ng standard as a calibrator and relative concentration of sample is obtained by multiplying fold change by calibrator, then averaged.

## Table S3: Genes Analyzed by Taqman.

| **Gene**  **Symbol** | **Applied Biosystems, Catalog #** | **Interrogated**  **Sequence** | **Exon Boundary** | **Probe**  **Binding Location** | **Tissue source for standard curve** |
| --- | --- | --- | --- | --- | --- |
| IFNα1 | Hs00256882_s1 | NM_024013 | 1-1 | 649 | Cervix tumor,  Ambion |
| IFNβ1 | Hs00277188_s1 | NM_002176 | 1-1 | 637 | Human monocyte, activated at Wyeth |
| IFNγ | Hs00174143_m1 | NM_000619 | 1-2 | 242 | Human PBMC activated at Wyeth |
| IL-13 | Hs00174379_m1 | NM_002188 | 1-2 | 192 | Thymus Ambion |

## Generation of Standard Curve RNA/cDNA

To utilize the standard curve method, a tissue is empirically determined to express the target gene using Applied Biosystems Taqman AOD. RNA preparations from sources listed in Table 2 above were converted to cDNA, as described above, and successfully assayed for expression of target gene transcripts by Taqman. Cycle threshold (Ct) values of >35 were considered below the limits of detection. For standard curve development, the goal was to achieve a Ct value between 18 and 25 for 100 ng of cDNA. This allowed for appropriate standard curve dynamic range. Standard curves consisted of two-fold serial dilutions of total cDNA from 100 ng/well to 1.5 ng/well. Standard curves were performed on each plate for every assay and were used for sample quantification and assay performance monitoring.

## Identification of Appropriate Endogenous Control

Genes that are expressed at similar levels in all samples (i.e. treated and untreated, lesional and non-lesional, etc.) can serve as endogenous controls in the relative standard curve method. An endogenous control is used to normalize sample quantification by accounting for variation in sample loading. Applied Biosystems suggests a number of genes for use as endogenous controls. We selected as a normalizer ZNF592.  This gene was identified by a survey of oligonucleotide array expression data for 44,928 transcripts across a compendium of 9,270 hybridizations, including multiple studies involving different types of cells and tissues.  In this broad survey, ZNF592 had substantially less variability than all commonly utilized endogneous controls, including the controls recommended by Applied Biosystems. Specifically, ZNF592 had a coefficient of variation represented the 0.01-th percentile of variation among all surveyed transcripts.  We have used ZNF592 in many of our studies involving skin, colon and PBMC and confirmed its utility as a normalizer gene due to very low variability in expression level within studies and across tissue and cell types.

Visual Representations of

# Information On Enrolled Subjects

To accurately capture the natural course of asthma in these clinical settings, no changes in how asthma care was delivered were allowed during the study. Further to accurately represent “typical” asthma subjects worldwide, a wide age range of subjects was chosen along with different racial groups. Also multinational clinics were chosen to ensure that different practice routines according to geographic diversity were obtained. This was all done to capture the closest possible model of global asthma in the real life setting of clinical practice to examine the natural history of this disease.

Asthma clinics chosen for the study were facilities where patients are seen on a regular basis and followed up - “research” clinics were excluded. This study employed longitudinal collection of clinical data to characterize patients receiving standard drug treatments. It was planned that data from this study be used to evaluate the natural history of asthma and its severity with clinical evaluations and lung function measures.

The summary tables below tabulate results for the 357 subjects enrolled in the study, including 20 smokers who were excluded from the analyses of *exacerbation*-related gene expression changes.

## Demographics and Asthma Status at Baseline

### Study Population

Between February 2003 and September 2004, 357 subjects 18 –83 years were enrolled in the study at 16 clinical centers. Nine sites were in the US, 3 in Australia and 4 in Europe (1 Iceland, 1 Ireland, and 2 UK). The subjects were stratified for severity at enrollment into mild persistent (36 subjects), moderate (149 subjects) or severe asthma (172 subjects).

The mean age of the subjects differed among the 3 asthma severity groups (*P*=0.014). Subjects in the mild asthma group were younger (41 years) than those in the severe asthma group (47 years). This difference in age was not clinically meaningful. Overall, more women (230 [64.4%]) than men (127 [35.6%]) were enrolled in the study, with the proportion of women to men about the same in each asthma severity category. Most subjects (311 [87.1%]) were white. With the exception of age, the 3 groups (mild, moderate, and severe asthma) generally were similar with respect to demographic characteristics.

Of the subjects with knowledge of blood relatives history (cutoff of first cousin), a greater proportion reported a genetic history of asthma (70% yes, 26% no) and allergic rhinitis (50% yes, 42% no). However, there was no significant difference in reported family history between the severity groups.

| Table S4: Demographic and Baseline Characteristics by Asthma Severity | | | | | |
| --- | --- | --- | --- | --- | --- |
|  |  | Asthma Severity | | |  |
| Characteristic | *P*-value | Mild (n=36) | Moderate (n=149) | Severe (n=172) | Total (N=357) |
|  |  |  |  |  |  |
| n |  | 36 | 149 | 172 | 357 |
| Age (yr) Mean | 0.014[a](#aoneway) | 41.14 | 43.40 | 47.37 | 45.08 |
| Standard deviation |  | 12.85 | 15.18 | 14.71 | 14.88 |
| Sex n (%) | 0.449[b](#bfishers) |  |  |  |  |
| Female |  | 26 (72.2) | 98 (65.8) | 106 (61.6) | 230 (64.4) |
| Male |  | 10 (27.8) | 51 (34.2) | 66 (38.4) | 127 (35.6) |
| Race n (%) | 0.125[b](#bfishers) |  |  |  |  |
| Asian |  | 0 | 4 (2.7) | 6 (3.5) | 10 (2.8) |
| Black |  | 1 (2.8) | 11 (7.4) | 24 (14.0) | 36 (10.1) |
| White |  | 35 (97.2) | 134 (89.9) | 142 (82.6) | 311 (87.1) |
| Ethnicity n (%) | 0.120[b](#bfishers) |  |  |  |  |
| Hispanic |  | 0 | 3 (2.0) | 10 (5.8) | 13 (3.6) |
| Non-Hispanic |  | 36 (100 ) | 146 (98.0) | 162 (94.2) | 344 (96.4) |
| Weight (kg) |  |  |  |  |  |
| n |  | 36 | 149 | 170 | 355 |
| Mean | 0.518[a](#aoneway) | 78.03 | 82.01 | 80.87 | 81.06 |
| Standard deviation |  | 13.29 | 18.83 | 19.88 | 18.86 |
| Height (cm) |  |  |  |  |  |
| n |  | 36 | 149 | 172 | 357 |
| Mean | 0.058[a](#aoneway) | 167.37 | 169.54 | 166.95 | 168.07 |
| Standard deviation |  | 10.39 | 10.08 | 9.47 | 9.88 |
| a One‑way analysis of variance with severity category as factor.  b Fisher's exact test *P*-value (2-tail) for comparison across asthma severity groups. | | | | | |

Subjects were followed for 12 months according to site standard of care.

Twenty-seven subjects (7.6%) did not complete the study. The most common reason overall for early withdrawal from the study was failure to return.

The most common reason for exclusion was FEV_1_ reversibility. The reasons for patient exclusion tended to be site-specific rather than country-specific.

### Patient and physician agreement of asthma control

The subjects’ level of asthma control was assessed at screening by both subjects and investigators. This assessment was done independently by subjects and investigators and was requested of the subject without using leading or coercive language. The subject’s assessment was not necessarily the same as the investigator’s assessment. The global assessment of asthma control at screening was rated as “good” in approximately 50% of subjects overall by both subjects and investigators. Subject and investigator assessments were in agreement for 68% of subjects.

| Table S5: Global Assessment of Asthma Control by the Subject and by the Investigator at Screening | | | | |
| --- | --- | --- | --- | --- |
|  | Asthma Severity | | |  |
| Characteristic | Mild (n=36) | Moderate (n=149) | Severe (n=172) | Total (N=357) |
| Subject’s Assessment^a^ n (%) |  |  |  |  |
| Excellent | 13 (36.1) | 32 (21.5) | 24 (14.0) | 69 (19.3) |
| Good | 18 (50.0) | 77 (51.7) | 87 (50.6) | 182 (51.0) |
| Fair | 5 (13.9) | 33 (22.1) | 48 (27.9) | 86 (24.1) |
| Poor | 0 | 6 (4.0) | 13 (7.6) | 19 (5.3) |
| Investigator’s Assessment n (%) |  |  |  |  |
| Excellent | 16 (44.4) | 31 (20.8) | 19 (11.0) | 66 (18.5) |
| Good | 19 (52.8) | 85 (57.0) | 86 (50.0) | 190 (53.2) |
| Fair | 1 (2.8) | 30 (20.1) | 52 (30.2) | 83 (23.2) |
| Poor | 0 | 3 (2.0) | 15 (8.7) | 18 (5.0) |
| a The assessment was not done for 1 subject. | | | | |

## Asthma Healthcare Resource Use

Subjects in each of the 3 asthma severity strata reported using some type of healthcare resource before enrolling in the study. In general, the proportion of subjects who reported using any resource and the frequency of use tended to increase with increasing disease severity. For each type of healthcare resource analyzed, there was a significant difference (*P*≤0.023) among asthma severity groups in terms of the proportion of subjects who used the resource. Most subjects (>70%) in this study, reported not visiting the emergency department for asthma, not requiring intubation for asthma, or not having experienced a near‑fatal episode due to asthma. Approximately 50% of subjects reported requiring an oral steroid taper or hospitalization for asthma on 1 or more occasion. Only about one-third of subjects reported never experiencing an exacerbation of their asthma.

| Table S6: Reported Asthma Healthcare Resource Use Before Enrollment | | | | | | |
| --- | --- | --- | --- | --- | --- | --- |
|  |  | Asthma Severity | | | |  |
| Characteristic | *P*-value^a^ | Mild (n=36) | Moderate (n=149) | Severe (n=172) | Total (N=357) | |
| **Number of ER Visits n (%)** | 0.01 |  |  |  |  | |
| None |  | 32 (88.9) | 129 (86.6) | 117 (68.0) | 278 (77.9) | |
| 1-3 Times |  | 4 (11.1) | 18 (12.1) | 40 (23.3) | 62 (17.4) | |
| 4-6 Times |  | 0 | 1 (0.7) | 10 (5.8) | 11 (3.1) | |
| >6 Times |  | 0 | 1 (0.7) | 5 (2.9) | 6 (1.7) | |
| **Number of Exacerbations n (%)** | 0.009 |  |  |  |  | |
| None |  | 15 (41.7) | 55 (36.9) | 41 (23.8) | 111 (31.1) | |
| 1-3 Times |  | 15 (41.7) | 62 (41.6) | 67 (39.0) | 144 (40.3) | |
| 4-6 Times |  | 3 (8.3) | 19 (12.8) | 27 (15.7) | 49 (13.7) | |
| >6 Times |  | 3 (8.3) | 13 (8.7) | 37 (21.5) | 53 (14.8) | |
| **Oral Steroid Taper n (%)** | <0.001 |  |  |  |  | |
| None |  | 26 (72.2) | 78 (52.3) | 61 (35.5) | 165 (46.2) | |
| 1-3 Times |  | 10 (27.8) | 53 (35.6) | 66 (38.4) | 129 (36.1) | |
| 4-6 Times |  | 0 | 14 (9.4) | 21 (12.2) | 35 (9.8) | |
| >6 Times |  | 0 | 4 (2.7) | 24 (14.0) | 28 (7.8) | |
| **Near-Fatal Episode due to Asthma n (%)** | 0.023 |  |  |  |  | |
| None |  | 33 (91.7) | 116 (77.9) | 112 (65.1) | 261 (73.1) | |
| 1-3 Times |  | 3 (8.3) | 27 (18.1) | 51 (29.7) | 81 (22.7) | |
| 4-6 Times |  | 0 | 4 (2.7) | 4 (2.3) | 8 (2.2) | |
| >6 Times |  | 0 | 2 (1.3) | 5 (2.9) | 7 (2.0) | |
| **Intubation n (%)** | 0.010 |  |  |  |  | |
| None |  | 36 (100) | 143 (96.0) | 150 (87.2) | 329 (92.2) | |
| 1-3 Times |  | 0 | 6 (4.0) | 19 (11.0) | 25 (7.0) | |
| 4-6 Times |  | 0 | 0 | 3 (1.7) | 3 (0.8) | |
| **Hospital Admissions for Asthma n (%)** | <0.001 |  |  |  |  | |
| None |  | 26 (72.2) | 88 (59.1) | 68 (39.5) | 182 (51.0) | |
| 1-3 Times |  | 6 (16.7) | 32 (21.5) | 49 (28.5) | 87 (24.4) | |
| 4-6 Times |  | 3 (8.3) | 15 (10.1) | 20 (11.6) | 38 (10.6) | |
| >6 Times |  | 1 (2.8) | 14 (9.4) | 35 (20.3) | 50 (14.0) | |
| a Fisher's exact test *P*-value (2-tail).  Abbreviations: ER = emergency room | | | | | | |

## Atopic status

Clinics were asked to determine the atopic status of subjects as routinely performed in their practice. When determined using objective criteria (prick skin testing) the proportion of subjects who were atopic was 40 % than when reported by nonobjective (history, total IgE) means (67%). There was a significant difference (*P*=0.017) among severity groups in terms of atopic status only when determined by skin testing.

Environmental history and exposure to animals or pets was similar between the severity strata and the proportion of subjects with various exposures remained constant over the 12 month course of the study.

| Table S7: Atopy Status at Screening | | | | | |
| --- | --- | --- | --- | --- | --- |
|  |  | Asthma Severity | | |  |
| Characteristic | *P*-value | Mild (n=36) | Moderate (n=149) | Severe (n=172) | Total (N=357) |
| Atopy Status (history, total IgE) n (%) | 0.488^a^ |  |  |  |  |
| Atopic |  | 23 (63.9) | 100 (67.1) | 116 (67.4) | 239 (66.9) |
| Nonatopic |  | 5 (13.9) | 23 (15.4) | 34 (19.8) | 62 (17.4) |
| Atopy Status (Skin Test/RAST) n (%) | 0.017^a^ |  |  |  |  |
| Atopic |  | 14 (38.9) | 71 (47.7) | 59 (34.3) | 144 (40.3) |
| Nonatopic |  | 14 (38.9) | 52 (34.9) | 91 (52.9) | 157 (44.0) |
|  |  |  |  |  |  |
| Unknown |  | 8 (22.2) | 26 (17.4) | 22 (12.8) | 56 (15.7) |
| a Fisher's exact test *P*-value (2-tail) for comparison across asthma severity groups. | | | | | |

## Tobacco use

There was no significant difference among the groups in terms of the proportion of subjects who used tobacco. The proportion of subjects who reported using tobacco at some time in the past was highest in Iceland (46%) and Australia (44%). Reported tobacco use at some time in the past was approximately the same (~30%) in UK, Ireland, and the USA. The only significant difference among severity strata in terms of the proportion of subjects who reported using tobacco was observed for the USA (*P*=0.009). Reported tobacco use increased with worsening asthma severity.

There was a statistically significant difference (p=0.007) among the asthma severity groups by exposure to second-hand smoke, the proportion of subjects exposed increased with worsening severity. Subjects who were still smokers at study initiation were excluded from the analysis reported here.

## Table S8: Body Mass Index and Gastrointestinal Reflux Disease:

When demographic characteristics were analyzed by sex , body mass index in men varied significantly (*P*=0.037) by asthma severity.

| Body mass index |
| --- |

| **BMI (men)** |  |  |  |  |  |
| --- | --- | --- | --- | --- | --- |
| n | p | 10 | 51 | 65 | 126 |
| Mean | 0.037[a](#aoneway) | 26.01 | 27.73 | 29.58 | 28.55 |
| Standard deviation |  | 2.97 | 4.98 | 5.19 | 5.07 |
| **BMI (Women)** |  |  |  |  |  |
| n |  | 26 | 98 | 105 | 229 |
| Mean | 0.907[a](#aoneway) | 28.82 | 28.87 | 28.49 | 28.69 |
| Standard deviation |  | 5.95 | 6.18 | 6.72 | 6.39 |
| a One‑way analysis of variance with severity category as factor.  b Fisher's exact test *P*-value (2-tail) for comparison across asthma severity groups.  Abbreviations: BMI = body mass index | | | | | |

A total of 142 (40%) of asthmatics in all severity groups used antacids of some sort. A statistically significant difference (p< 0.05) in the use of these medications was seen between the severity groups with 8 (22%) subjects in the mild, 53 (36%) in the moderately severe and 81 (47%) in the severe asthma group (Table VI).

| Table S9: History of Reflux Disease | | | | | |
| --- | --- | --- | --- | --- | --- |
| Category | Overall *P*‑Value | Asthma Severity | | | Total (N=357) |
|  |  | Mild (n=36) | Moderate (n=149) | Severe (n=172) |  |
| Subjects with History of Reflux n (%) | 0.0232 | 4 (11.1) | 39 (26.2) | 56 (32.6) | 99 (27.7) |
| Men with Medical History Data |  | 10 | 51 | 66 | 127 |
| Men with History of Reflux n (%) | 0.6402 | 1 (10.0) | 14 (27.5) | 18 (27.3) | 33 (26.0) |
| Women with Medical History Data |  | 26 | 98 | 106 | 230 |
| Women with History of Reflux n (%) | 0.0302 | 3 (11.5) | 25 (25.5) | 38 (35.8) | 66 (28.7) |
| a Overall *P*-value: Fisher’s exact test P-value (2-tail) for comparison across severity groups. | | | | | |

## Changes in asthma severity over 12 months of study

The number and proportion of subjects who exhibited a change in asthma severity class during the study are summarizedbelow. Whereas most subjects with mild asthma remained the same at each visit (10-20%), most subjects with moderate or severe asthma changed severity strata as determined by severity scores (60-70% and 70-80% respectively).

| Information Table S10: Subjects with a Change in Asthma Severity by Visit | | | | | |
| --- | --- | --- | --- | --- | --- |
| Asthma Severity at Screening | Status | Visit 3 n (%) | Visit 4 n (%) | Visit 5 n (%) | Visit 6 n (%) |
| Mild (n=36) | Same | 27 (79.4) | 30 (88.2) | 26 (76.5) | 29 (90.6) |
|  | Changed | 7 (20.6) | 4 (11.8) | 8 (23.5) | 3 (9.4) |
| Moderate (n=149) | Same | 48 (33.6) | 52 (36.1) | 41 (29.1) | 42 (29.4) |
|  | Changed | 95 (66.4) | 92 (63.9) | 100 (70.9) | 101 (70.6) |
| Severe (n=172) | Same | 44 (26.8) | 37 (22.7) | 33 (20.6) | 31 (19.3) |
|  | Changed | 120 (73.2) | 126 (77.3) | 127 (79.4) | 130 (80.7) |
|  | | | | | |

## Concomitant Anti-asthmatic Medications

The number and proportion of subjects who used concomitant anti-asthmatic medications at any time during the study are summarized by severity are summarized below. There was a statistically significant difference (P<0.0001) among severity groups with regard to the use of systemic and inhaled corticosteroids and leukotriene antagonists. It is noted that the proportion of subjects with moderate or severe asthma who used inhaled corticosteroids was similar. A larger proportion of subjects, overall and by severity strata, used systemic corticosteroids when all visits were taken into consideration as compared with scheduled non-*exacerbation* visits. This may be related to the use of short‑term tapering corticosteroid regimens during exacerbation attack visits, which are included in all visits. The use of intranasal corticosteroids was similar across all 3 severity strata throughout the study as well as during non-exacerbation phases.

| Table S11: Number (%) of Subjects Who Used Concomitant Anti-asthmatic Medications by Asthma Severity | | | | | |
| --- | --- | --- | --- | --- | --- |
|  |  | Asthma Severity | | |  |
| Characteristic | *P*-value^a^ | Mild (n=36) | Moderate (n=149) | Severe (n=172) | Total (N=357) |
| All Visits |  |  |  |  |  |
| Systemic corticosteroids | <0.0001 | 5 (13.9) | 63 (42.3) | 91 (52.9) | 159 (44.5) |
| Inhaled corticosteroids | <0.0001 | 30 (83.3) | 147 (98.7) | 168 (97.7) | 345 (96.6) |
| Intranasal corticosteroids | 0.3876 | 18 (50.0) | 79 (53.0) | 78 (45.4) | 175 (49.0) |
| Leukotriene antagonists | <0.0001 | 3 (8.3) | 36 (24.2) | 71 (41.2) | 110 (30.8) |
| Scheduled Non-Exacerbation Visits | | | | | |
| Systemic corticosteroids | <0.0001 | 0 | 33 (22.2) | 70 (40.7) | 103 (28.9) |
| Inhaled corticosteroids | <0.0001 | 29 (80.6) | 147 (98.7) | 168 (97.7) | 344 (96.4) |
| Intranasal corticosteroids | 0.2819 | 18 (50.0) | 79 (53.0) | 76 (44.2) | 173 (48.5) |
| Leukotriene antagonists | <0.0001 | 3 (8.3) | 35 (23.5) | 69 (40.1) | 107 (30.0) |
| a Chi-square test for comparison across asthma severity groups. | | | | | |

There was a significant difference (P=0.005) among severity groups with regard to the use of systemic antibacterial agents. A higher proportion of subjects with moderate (53.0%) or severe (59.9%) asthma used systemic antibacterial agents as compared with subjects who had mild asthma (30.6%).

### Geographical differences in the use of asthma medications

Concomitant anti-asthmatic medication use by country is summarized below for all visits and for scheduled non‑exacerbation visits (Visits 2 through 6). Of interest are the relatively larger proportion of subjects in Iceland who used intranasal steroids and the relatively smaller proportion of subjects in Australia and Great Britain who used leukotriene antagonists. These differences among countries were statistically significant (*P*<0.0001) throughout the study as well as at scheduled non-exacerbation visits and may reflect differences in regional prescribing practices or the availability of drugs approved for marketing in various areas.

| Table S12: Number (%) of Subjects Who Used Concomitant Anti-asthmatic Medications by Country | | | | | | |
| --- | --- | --- | --- | --- | --- | --- |
|  |  | Country | | | | |
| Characteristic | *P*-value | Australia (n=73) | G Britain (n=51) | Ireland (n=35) | Iceland (n=61) | USA (n=137) |
| **All Visits** |  |  |  |  |  |  |
| Systemic corticosteroids | 0.0635^a^ | 28 (38.4) | 20 (39.2) | 13 (37.1) | 37 (60.7) | 61 (44.5) |
| Inhaled corticosteroids | 0.1030^b^ | 72 (98.6) | 48 (94.1) | 35 (100.0) | 61 (100.0) | 129 (94.2) |
| Intranasal corticosteroids | <0.0001^a^ | 19 (26.0) | 15 (29.4) | 18 (51.4) | 53 (86.9) | 70 (51.1) |
| Leukotriene antagonists | <0.0001^a^ | 6 (8.2) | 5 (9.8) | 14 (40.0) | 22 (36.1) | 63 (46.0) |
| **Scheduled Non-Exacerbation Visits** | | | | | | |
| Systemic corticosteroids | 0.8955 ^a^ | 18 (24.7) | 15 (29.4) | 10 (28.6) | 17 (27.9) | 43 (31.4) |
| Inhaled corticosteroids | 0.1770^b^ | 71 (97.3) | 48 (94.1) | 35 (100.0) | 61 (100.0) | 129 (94.2) |
| Intranasal corticosteroids | <0.0001^a^ | 18 (24.7) | 15 (29.4) | 17 (48.6) | 53 (86.9) | 70 (51.1) |
| Leukotriene antagonists | <0.0001^a^ | 5 (6.9) | 5 (9.8) | 14 (40.0) | 22 (36.1) | 61 (44.5) |
| a Chi-square test for comparison across asthma severity groups  b Fisher’s exact test for comparison across asthma severity groups | | | | | | |

### Asthma Healthcare Resource Use During Study

A summary of reported asthma healthcare resource use at any time from visit 3 onward is presented below. Subjects in each asthma severity stratum reported using at least 1 type of healthcare resource during the study. A greater proportion of subjects with moderate or severe asthma than with mild asthma reported that they experienced an exacerbation (*P*=0.0043) or required an oral steroid tapering regimen (*P*=0.0028). There were, however, subjects in the mild asthma category who reported exacerbations during the study (35%) or reported requiring an oral steroid taper (15%). None of the subjects in the mild asthma group reported visiting the emergency department or a hospital admission, and none of the subjects in the mild or moderate asthma groups reported experiencing a near-fatal episode or requiring intubation. When compared with healthcare resource use reported at screening, the proportion of subjects who reported using healthcare resources during the study was lower, particularly for visits to the emergency department, near‑fatal episodes, intubation, and hospital admissions.

| Table S13: Reported Asthma Healthcare Resource Use During the Study (number of events reported) | | | | | |
| --- | --- | --- | --- | --- | --- |
|  |  | Asthma Severity | | |  |
| Characteristic | *P*-value^a^ | Mild (n=34) | Moderate (n=147) | Severe (n=169) | Total (N=350) |
| **Number of ER Visits n (%)** | 0.0690 | 0 | 7 (4.8) | 16 (9.5) | 23 (6.6) |
| 1-3 Times |  | 0 | 7 | 26 | 33 |
| **Number of Exacerbations n (%)** | 0.0043 | 12 (35.3) | 63 (42.9) | 99 (58.6) | 174 (49.7) |
| 1-3 Times |  | 22 | 128 | 243 | 393 |
| 4-6 Times |  | 0 | 2 | 2 | 4 |
| >6 Times |  | 0 | 2 | 0 | 2 |
| **Oral Steroid Taper n (%)** | 0.0028 | 5 (14.7) | 56 (38.1) | 76 (45.0) | 137 (39.1) |
| 1-3 Times |  | 8 | 85 | 180 | 273 |
| 4-6 Times |  | 0 | 0 | 2 | 2 |
| >6 Times |  | 0 | 1 | 5 | 6 |
| **Near-Fatal Episode n (%)** | 0.5932 | 0 | 0 | 2 (1.2) | 2 (0.6) |
| 1-3 Times |  | 0 | 0 | 2 | 2 |
| Intubation n (%) | 1.0000 | 0 | 0 | 1 (0.6) | 1 (0.3) |
| 1-3 Times |  | 0 | 0 | 1 | 1 |
| **Hospital Admissions n (%)** | 0.2243 | 0 | 6 (4.1) | 12 (7.1) | 18 (5.1) |
| 1-3 Times |  | 0 | 6 | 17 | 23 |
| a Fisher's exact test *P*-value (2-tail) for comparison across asthma severity groups.  room  Abbreviations: ER = emergency Source: | | | | | |

## Asthma Precipitating or Aggravating Factors During the Study

Viral respiratory infections were the most common risk factor, and was similar across all visits and severity strata (Table XI). This was followed by changes in weather/exposure to cold air, exercise, environmental allergens (indoor and outdoor), tobacco smoke and emotions. Aspirin or NSAIDs remained less important factors throughout the study. Although these factors such as exposure to irritants were less often triggers for exacerbations in the mild vs moderate and severe groups, these numbers were not significant. There was no difference among severity strata in terms of the importance of these factors at screening or changes from the previous visit.

| Table S14: Asthma Precipitating or Aggravating Factors by Visit | | | | | |
| --- | --- | --- | --- | --- | --- |
| Characteristic | Visit 1 | Visit 3 | Visit 4 | Visit 5 | Visit 6 |
|  | Screening (N=357) | Wk 1-13 (N=341) | Wk 14-26 (N=341) | Wk 27-39 (N=335) | Wk 40-52 (N=335) |
| **Viral Respiratory Infections n (%)** |  |  |  |  |  |
| Yes | 325 (91.0) |  |  |  |  |
| No | 30 (8.4) |  |  |  |  |
| No Change Since Last Visit |  | 337 (98.8) | 340 (99.7) | 334 (99.7) | 335 (100) |
| **Changes in Weather, Exposure to Cold Air n (%)** |  |  |  |  |  |
| Yes | 307 (86.0) |  |  |  |  |
| No | 46 (12.9) |  |  |  |  |
| No Change Since Last Visit |  | 338 (99.1) | 341 (100) | 334 (99.7) | 334 (99.7) |
| **Exercise n (%)** |  |  |  |  |  |
| Yes | 290 (81.2) |  |  |  |  |
| No | 64 (17.9) |  |  |  |  |
| No Change Since Last Visit |  | 338 (99.1) | 341 (100) | 334 (99.7) | 334 (99.7) |
| **Environmental Allergens n (%)** |  |  |  |  |  |
| Yes | 283 (79.3) |  |  |  |  |
| No | 72 (20.2) |  |  |  |  |
| No Change Since Last Visit |  | 341 (100) | 341 (100) | 334 (99.7) | 335 (100) |
| **Smoke n (%)** |  |  |  |  |  |
| Yes | 278 (77.9) |  |  |  |  |
| No | 74 (20.7) |  |  |  |  |
| No Change Since Last Visit |  | 340 (99.7) | 339 (99.4) | 333 (99.4) | 335 (100) |
| **NSAIDs or Aspirin n (%)** |  |  |  |  |  |
| Yes | 46 (12.9) |  |  |  |  |
| No | 289 (81.0) |  |  |  |  |
| No Change Since Last Visit |  | 340 (99.7) | 341 (100) | 334 (99.7) | 335 (100) |
| Note: Subjects without a “yes” or “no” response were not exposed or exposure status was unknown (unknown was categorized as not exposed).  Abbreviations: NSAIDs = nonsteroidal antiinflammatory drugs; Wk = Weeks | | | | | |

## Adverse Events During the Study

Adverse events were reported for a total of 310 (86.8%) subjects overall, including 27 (75.0%) subjects in the mild asthma group, 126 (84.6%) subjects in the moderate asthma group, and 157 (91.3%) subjects in the severe asthma group. A summary of adverse events (AE) by asthma severity is provided below

| Table S15: Number (%) of Subjects Experiencing Adverse Events | | | | | |
| --- | --- | --- | --- | --- | --- |
| Adverse Event | Overall *P*‑Value^a^ | Asthma Severity | | | Total (N=357) |
|  |  | Mild (n=36) | Moderate (n=149) | Severe (n=172) |  |
| Any AE | 0.020* | 27 (75.0) | 126 (84.6) | 157 (91.3) | 310 (86.8) |
| Any study‑related AE | 0.365 | 0 | 2 (1.3) | 0 | 2 (0.6) |
| Any grade 3 or 4 AE^b^ | 0.035* | 1 (2.8) | 20 (13.4) | 32 (18.6) | 53 (14.8) |
| Any SAE | 0.169 | 2 (5.6) | 23 (15.4) | 31 (18.0) | 56 (15.7) |
| a Overall *P*-value: Fisher's exact test *P*-value (2-tail) for comparison across asthma severity groups. Statistical significance at the 0.05, 0.01, 0.001 levels is denoted by *, **, ***, respectively.  b Severe or life-threatening  Abbreviations: AE = adverse event; SAE = serious adverse event | | | | | |

The incidence of AEs differed among the severity groups (*P*<0.05), with higher incidences reported for the moderate and severe asthma groups as compared with the mild asthma group.

The most common AEs overall were reported for the respiratory system (72.5% of all subjects) and body as a whole (60.8% of all subjects). Among the most common AEs in the respiratory system, the incidences of dyspnea, wheezing, and increased sputum differed among the 3 severity groups (*P*<0.05). The incidence of each of these AEs was highest in the severe asthma group (43.6%, 37.2%, and 12.2%, respectively) and lowest in the mild asthma group (25.0%, 25.0%, and 2.8%, respectively) (Table XIII)

The incidence of severe respiratory system AEs was significantly different (*P*=0.008) among the 3 asthma severity groups: 0 for mild; 2.7% for moderate; and 9.9% for severe. The incidence of wheezing was also different (*P*=0.018) among the 3 groups (0, 0.7%, and 5.8%, respectively).

| Table S16: Most Common (≥10% of Subjects in Any Severity Group) Respiratory Adverse Events, Number (%) of Subjects | | | | | |
| --- | --- | --- | --- | --- | --- |
|  | Overall P‑Value^a^ | Asthma Severity | | | Total (N=357) |
|  |  | Mild (n=36) | Moderate (n=149) | Severe (n=172) |  |
| Respiratory system | 0.033* | 22 (61.1) | 102 (68.5) | 135 (78.5) | 259 (72.5) |
| Cough increased | 0.868 | 13 (36.1) | 59 (39.6) | 71 (41.3) | 143 (40.1) |
| Dyspnea | 0.048* | 9 (25.0) | 50 (33.6) | 75 (43.6) | 134 (37.5) |
| Wheezing | 0.032* | 9 (25.0) | 36 (24.2) | 64 (37.2) | 109 (30.5) |
| Rhinitis | 0.716 | 11 (30.6) | 41 (27.5) | 43 (25.0) | 95 (26.6) |
| Pharyngitis | 0.234 | 9 (25.0) | 21 (14.1) | 24 (14.0) | 54 (15.1) |
| Upper respiratory infection | 0.625 | 3 (8.3) | 20 (13.4) | 26 (15.1) | 49 (13.7) |
| Sinusitis | 0.532 | 4 (11.1) | 22 (14.8) | 18 (10.5) | 44 (12.3) |
| Pulmonary physical finding | 0.359 | 1 (2.8) | 13 (8.7) | 18 (10.5) | 32 (9.0) |
| Sputum increased | 0.014* | 1 (2.8) | 6 (4.0) | 21 (12.2) | 28 (7.8) |
| a Overall *P*-value: Fisher's exact test *P*-value (2-tail) for comparison across asthma severity groups. Statistical significance at the 0.05, 0.01, 0.001 levels is denoted by *, **, *** respectively. The incidence of AEs reported for all other body systems was less than 20. | | | | | |

## Pulmonary Function Tests

Mean FEV_1_ values during scheduled non-exacerbation visits are summarized by asthma severity in Table XVIII. There was a difference in FEV_1_ among asthma severity groups, and this difference was highly statistically significant (*P*=0.0000). Mean FEV_1_ values decreased with worsening asthma severity. Furthermore, the difference was observed both in subjects who had experienced at least 1 *exacerbation* as well as those who had never experienced an *exacerbation*.

| Table S17: Mean FEV1 (% Predicted) at Scheduled Non-Exacerbation Visits | | | | |
| --- | --- | --- | --- | --- |
| Exacerbation Status | Statistic | Asthma Severity | | |
|  |  | Mild | Moderate | Severe |
| Never | n | 81 | 295 | 300 |
|  | **Mean** | **95.01** | **85.51** | **74.85** |
|  | *P*-value^a^ | 0.0000 |  |  |
| At Least 1 | n | 33 | 181 | 244 |
|  | **Mean** | **90.27** | **86.33** | **74.15** |
|  | *P*-value^a^ | 0.0000 |  |  |
|  | *P*-value^b^ | 0.0479 | 0.5675 | 0.6851 |
| All Subjects | n | 114 | 476 | 544 |
|  | **Mean** | **93.64** | **85.83** | **74.54** |
|  | *P*-value^a^ | 0.0000 |  |  |
| a *P*-value indicates test for differences among asthma severity groups  b *P*-value indicates test for difference between exacerbation status groups (never had an exacerbation versus had at least 1 exacerbation) within an asthma severity category  Abbreviations: FEV_1_ = forced expiratory volume in 1 second | | | | |

# Figure S6A: Relative FDR p-value Obtained From ANCOVA On Subgroup X Samples Using Only Exacerbation Samples with Corresponding Follow-up Sample


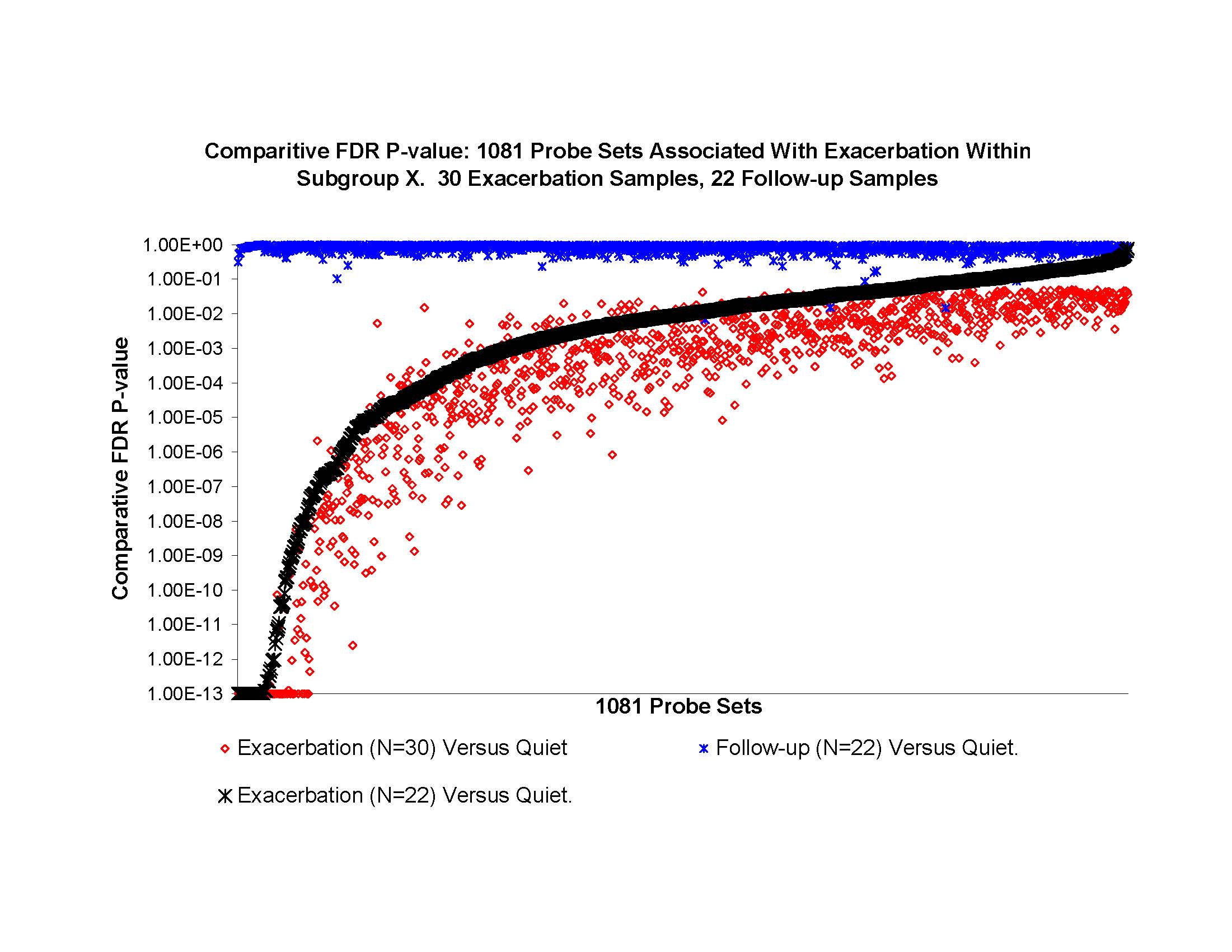


Comparison of relative FDR p-values for association with exacerbation obtained using N= 30 *exacerbation* samples and N = 22 *exacerbation* samples for which a follow-up sample was available. As expected, there is in general a small reduction in significance with the smaller sample number, but relative FDR p-values are very similar.

# Figure S6B: Relative FDR p-value Obtained From ANCOVA On Subgroup Y Samples Using Only Exacerbation Samples with Corresponding Follow-up Sample


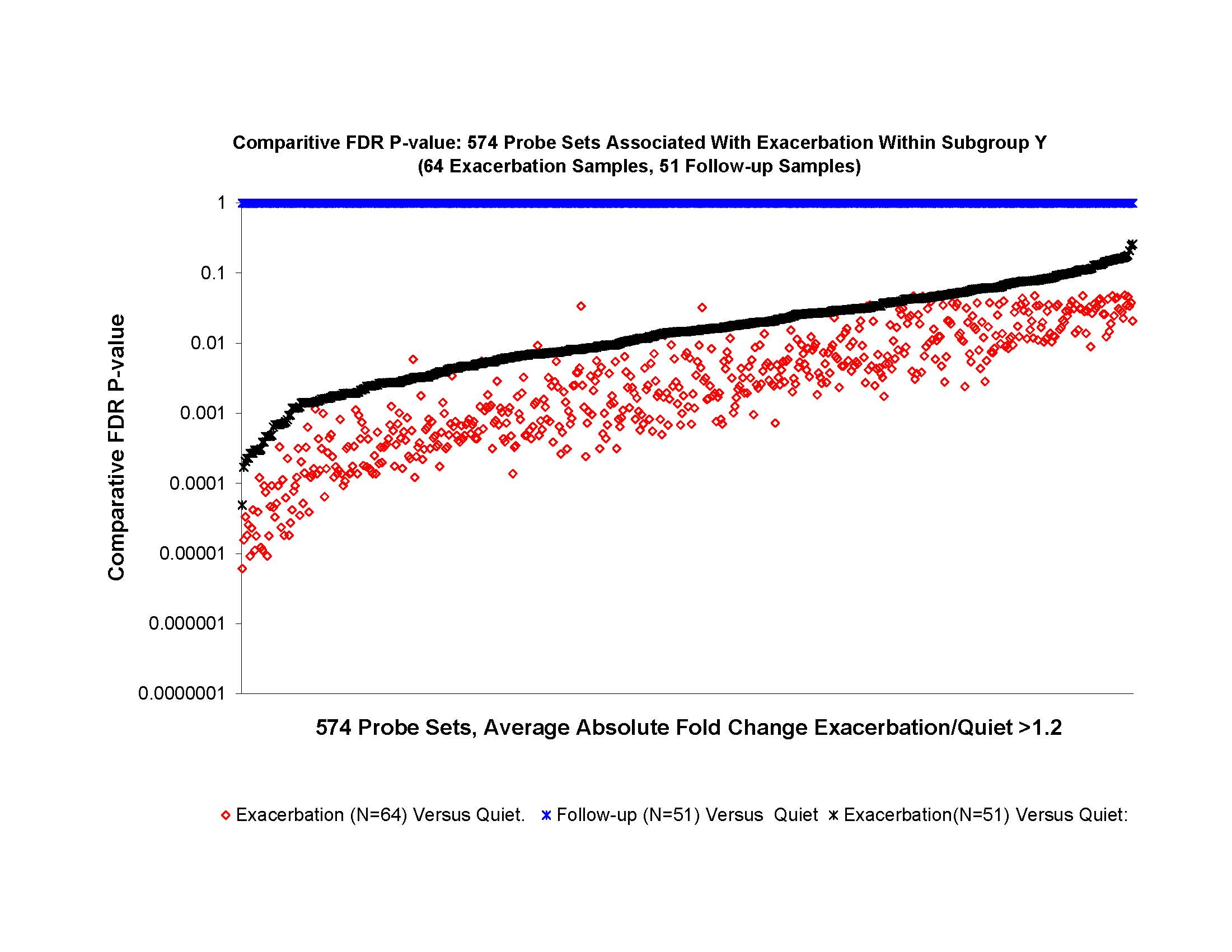


Comparison of relative FDR p-values for association with exacerbation obtained using N= 64 *exacerbation* samples and N = 51 *exacerbation* samples for which a follow-up sample was available. As expected, there is in general a small reduction in significance with the smaller sample number, but relative FDR p-values are very similar.

# Figure S6C: Relative FDR p-value Obtained From ANCOVA On Subgroup Z Samples Using Only Exacerbation Samples with Corresponding Follow-up Sample.


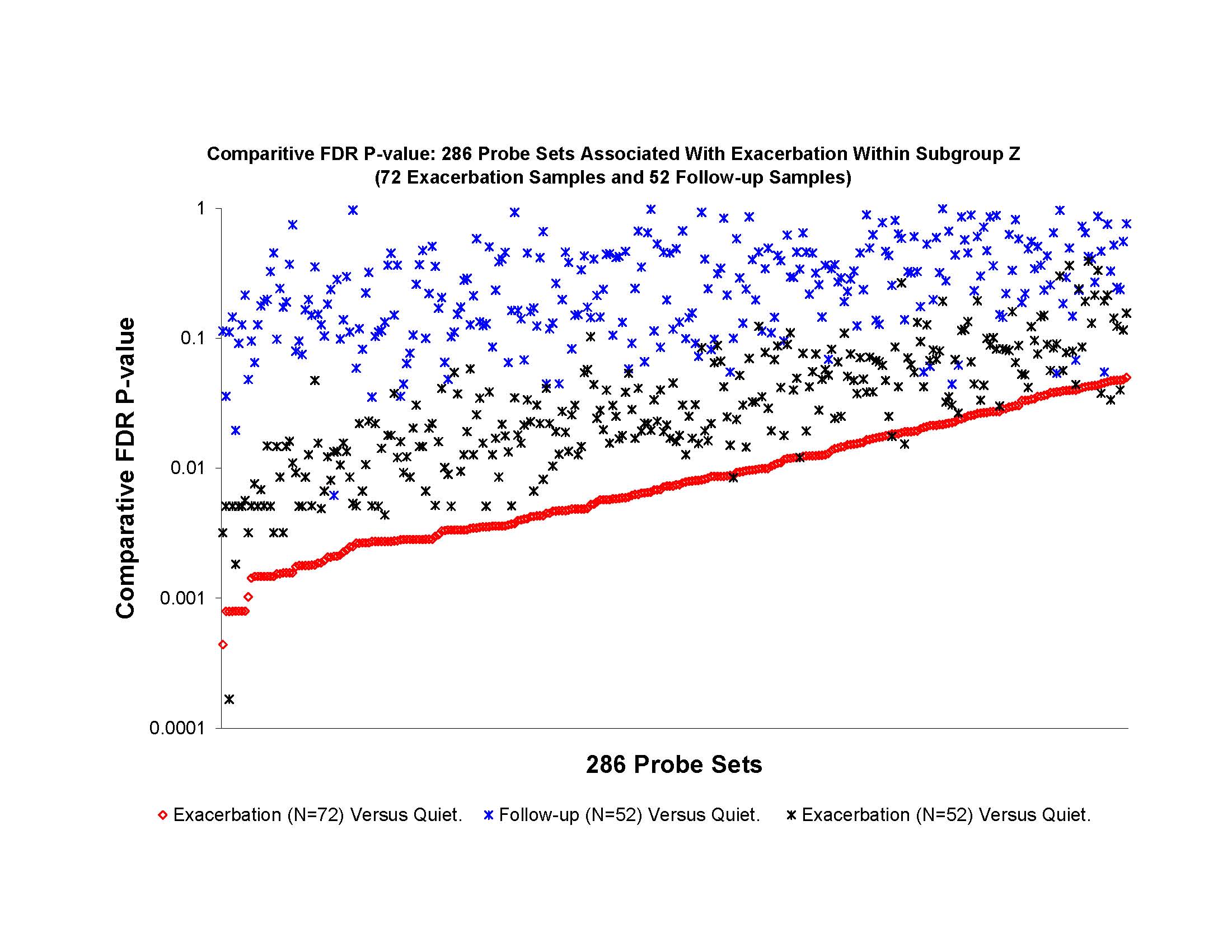


Results of ANCOVA indicating the lack of a robust gene expression pattern (in comparison to Subgroups X and Y) associated with Subgroup Z exacerbations. In the analysis using the 52 exacerbation samples for which a corresponding follow-up sample was available, the FDRs in the *Quiet* versus *Exacerbation* analysis is, as expected, less significant than the FDRs obtained with the larger sample set (N = 72).

# List by Subgroup of Probesets (Genes) Associated with Exacerbation

## Table S18A: ANCOVA Results Subgroup X

##
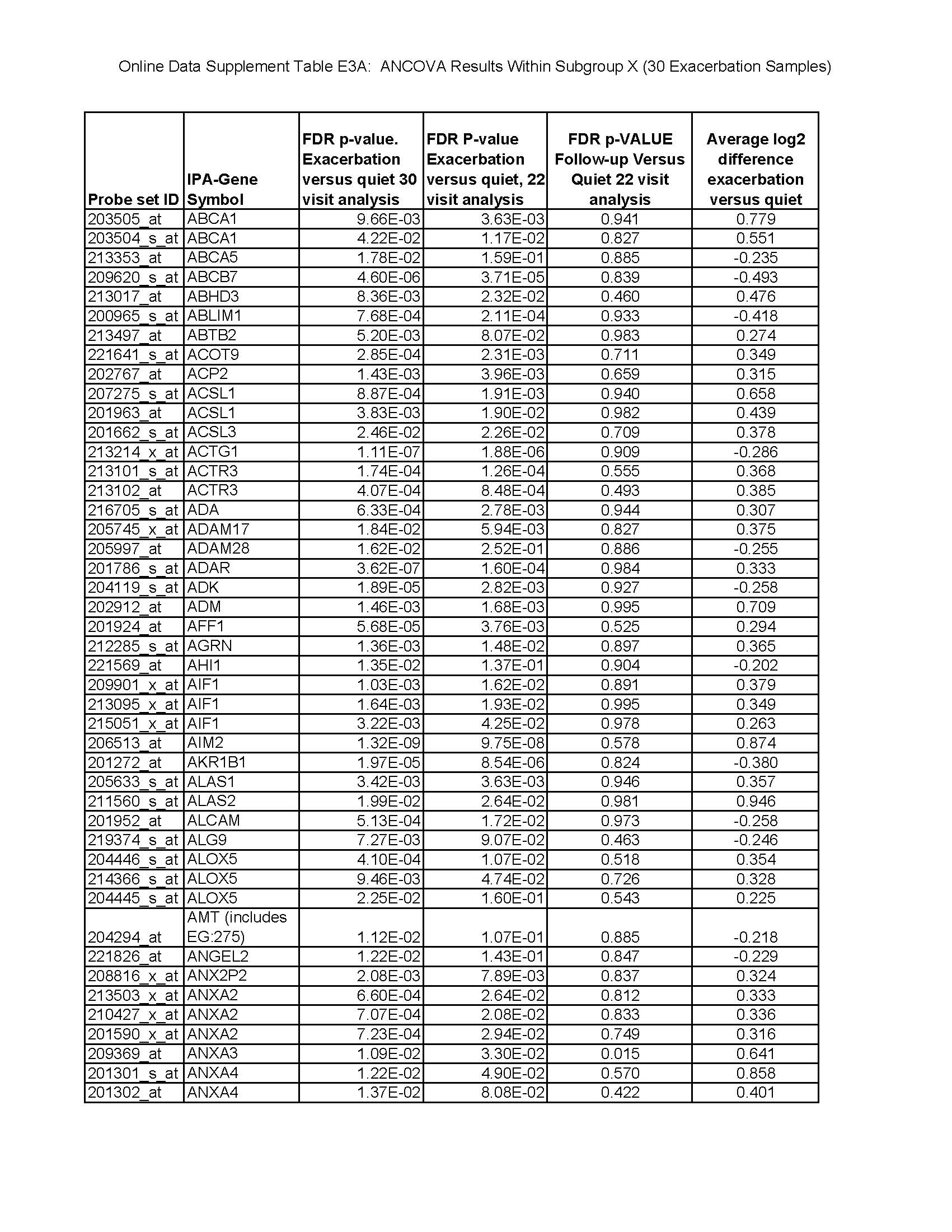


Table S18A: ANCOVA Results Subgroup X, continued
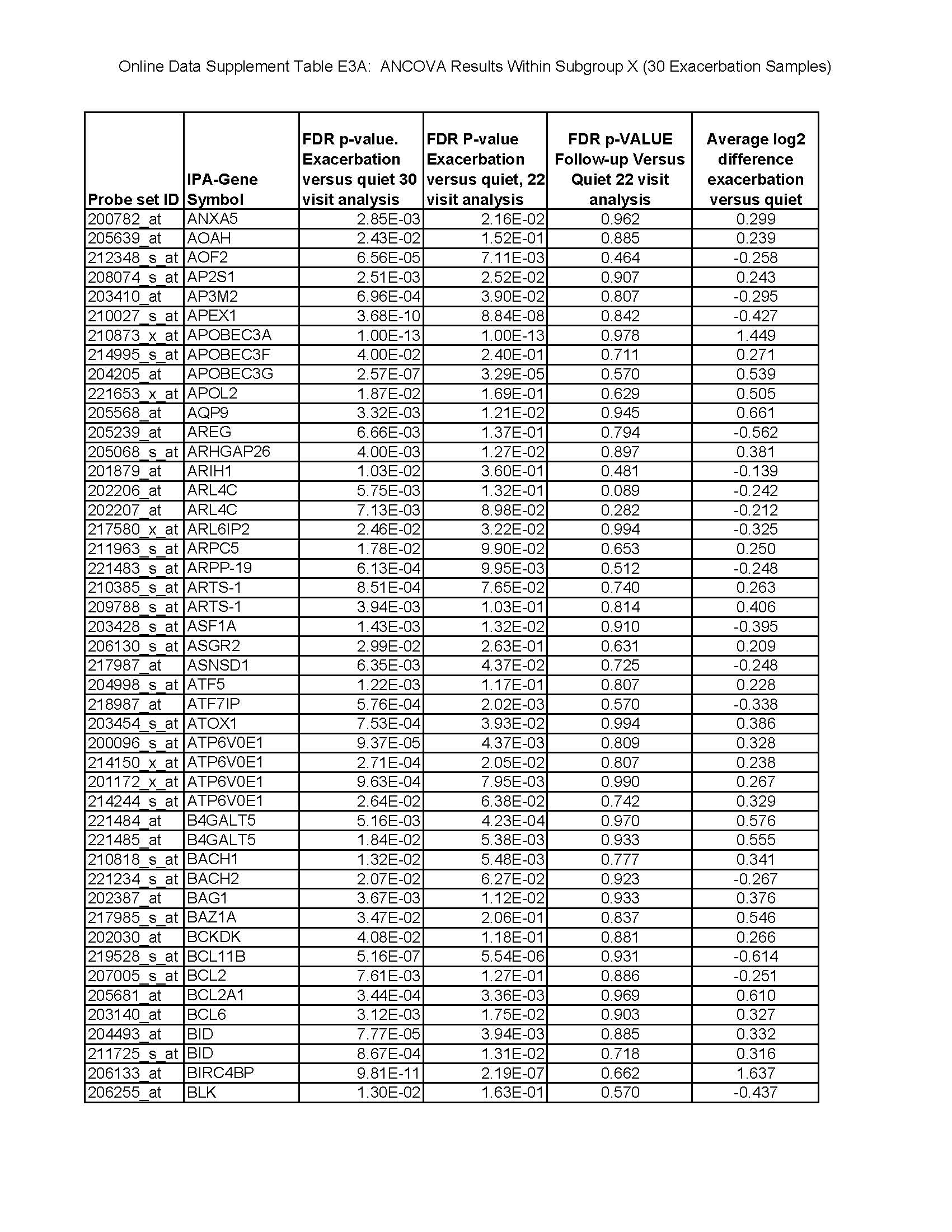


Table S18A: ANCOVA Results Subgroup X, continued
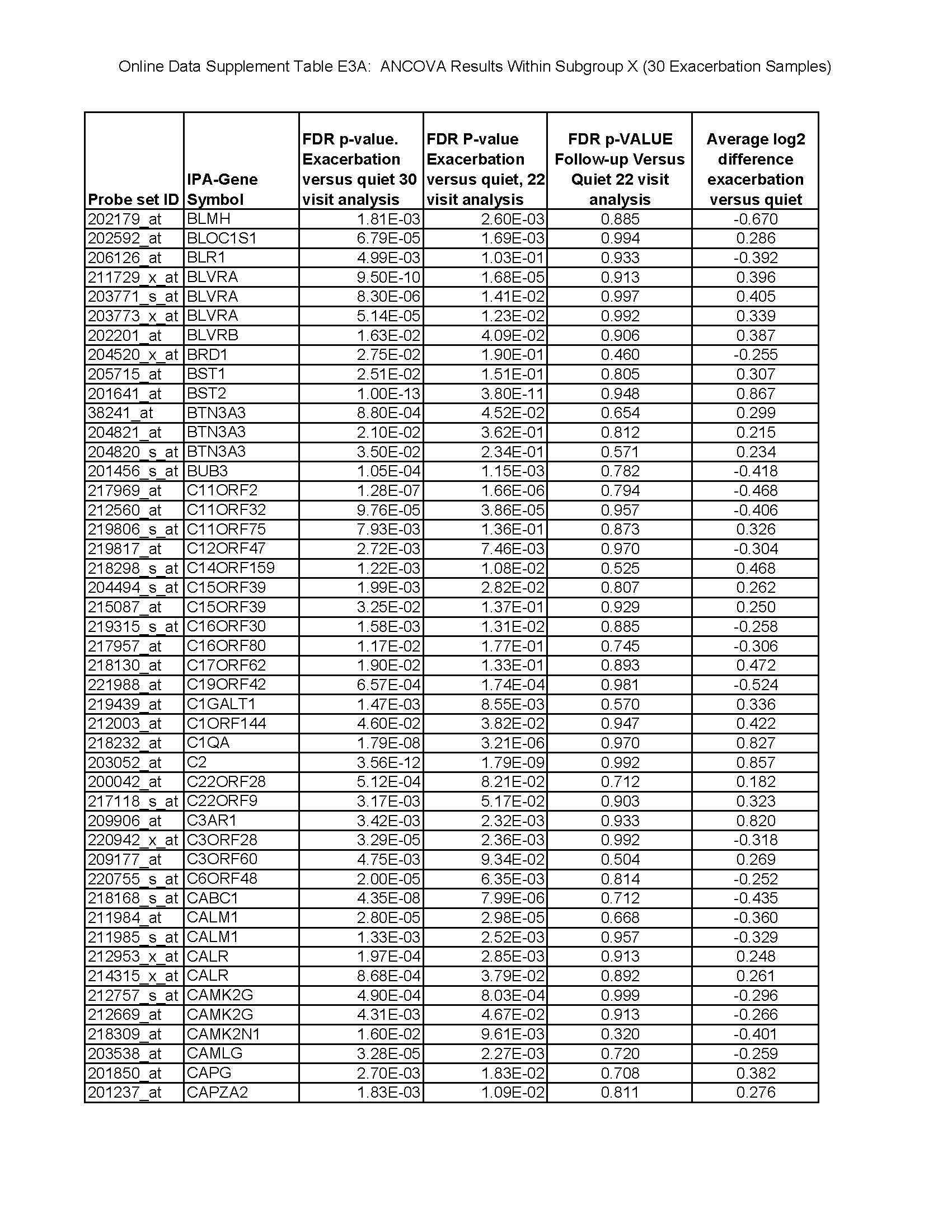


Table S18A: ANCOVA Results Subgroup X continued \
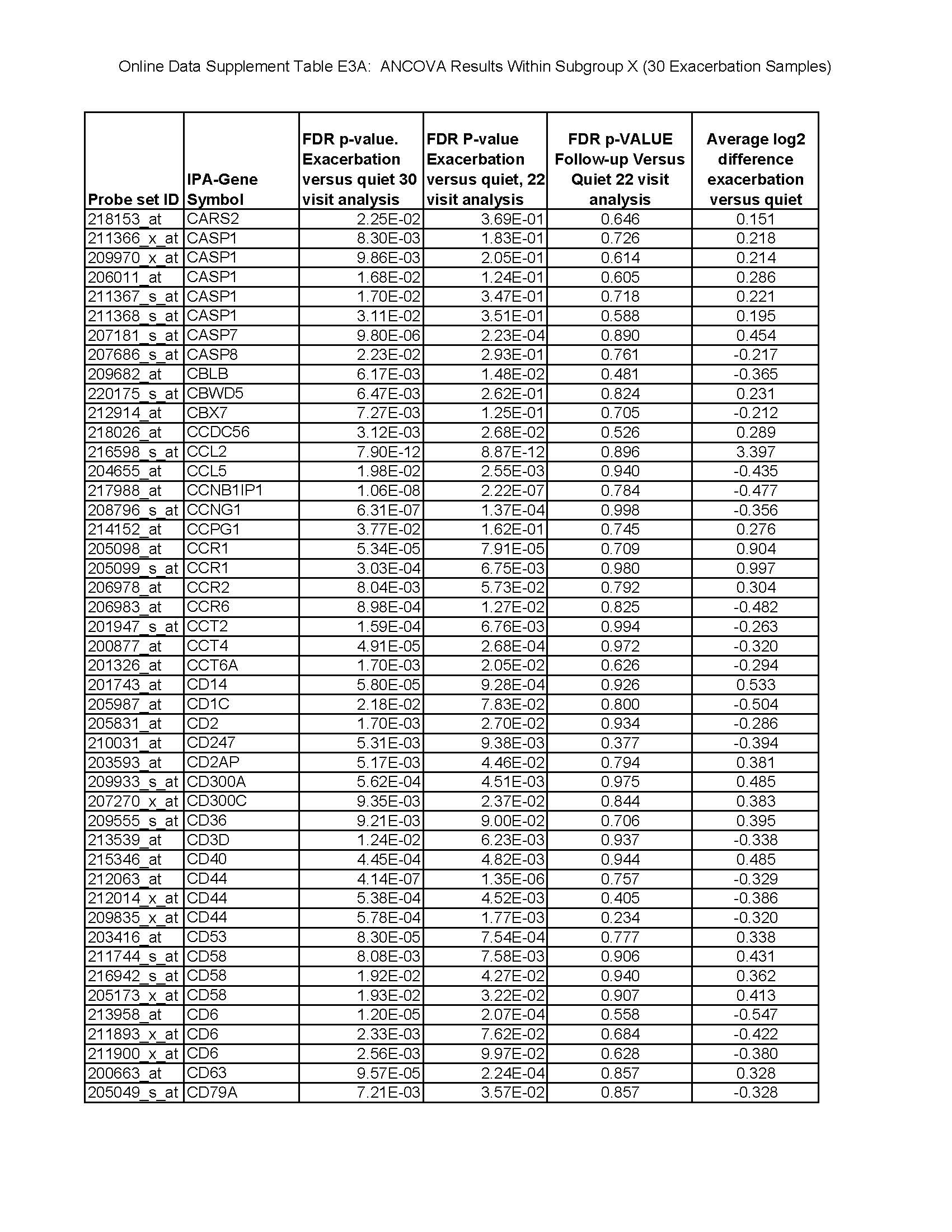


Table S18A: ANCOVA Results Subgroup X continued
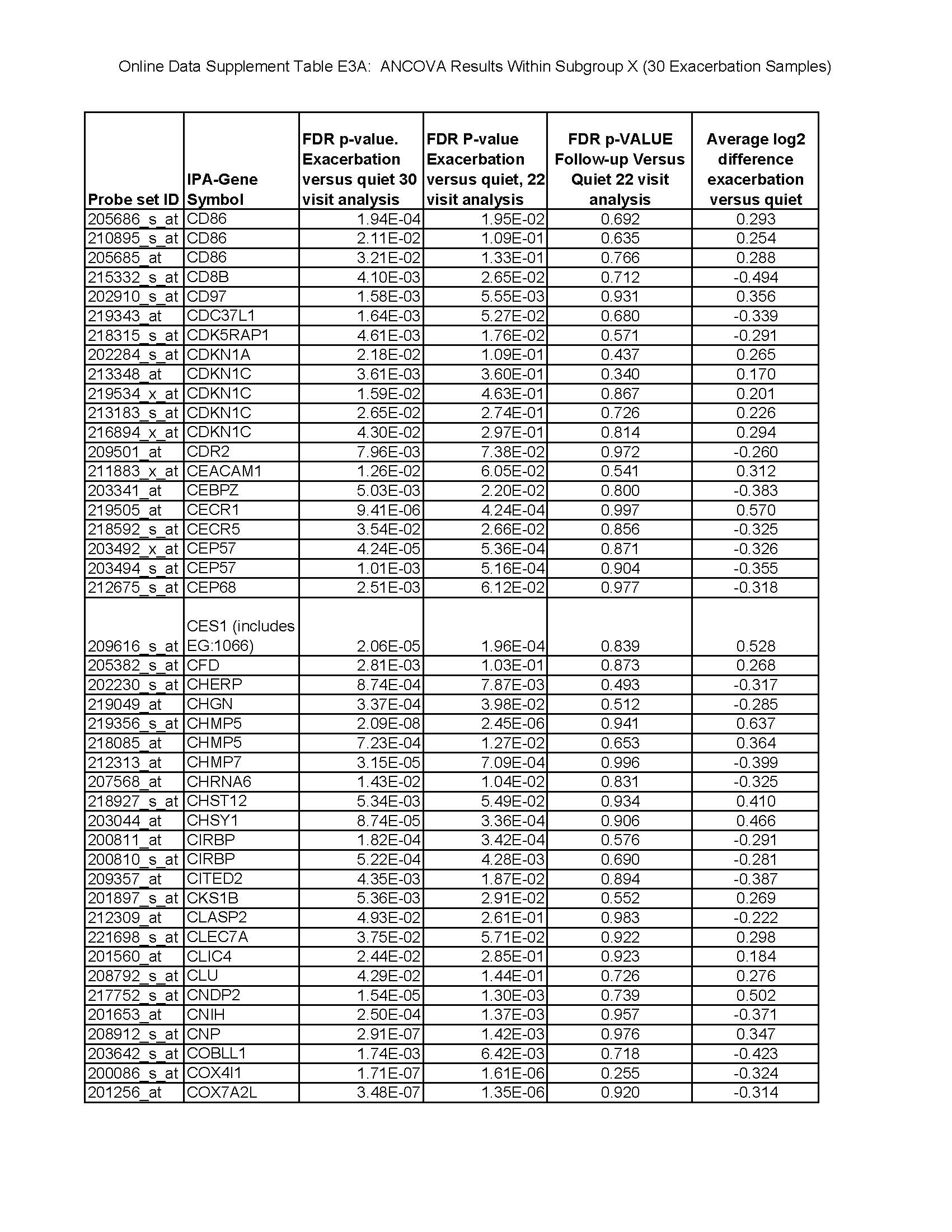


Table S18A: ANCOVA Results Subgroup X continued
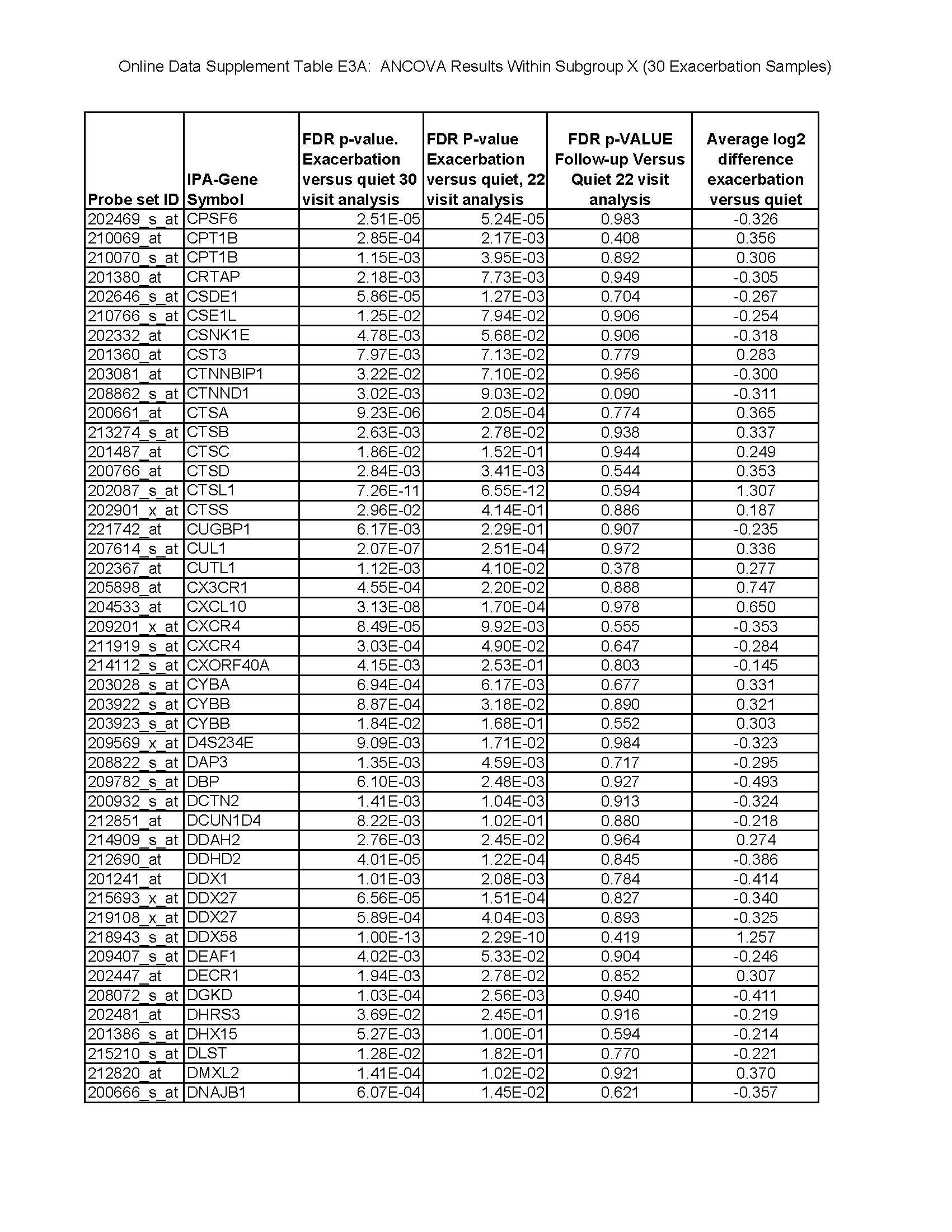


Table S18A: ANCOVA Results Subgroup X continued
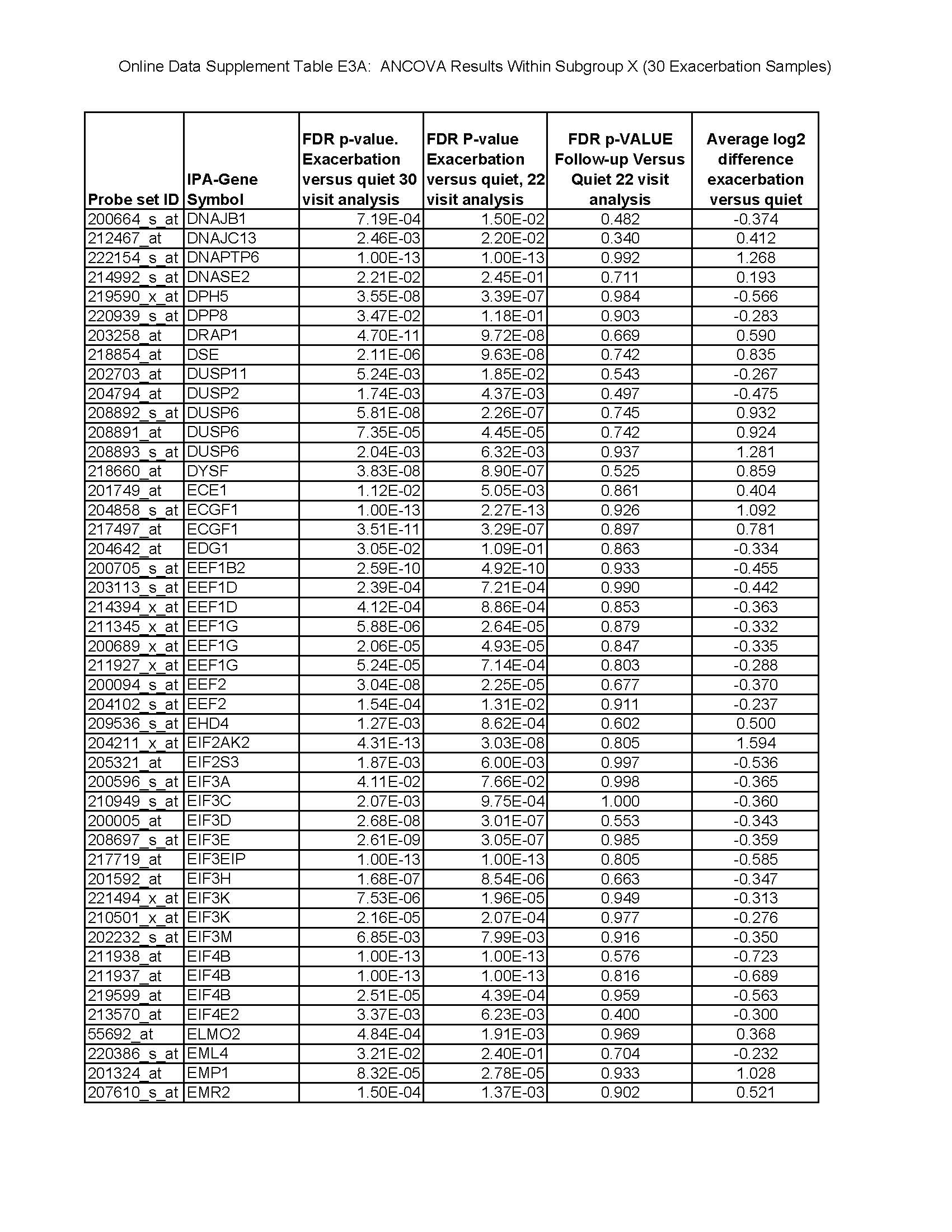


Table S18A: ANCOVA Results Subgroup X continued
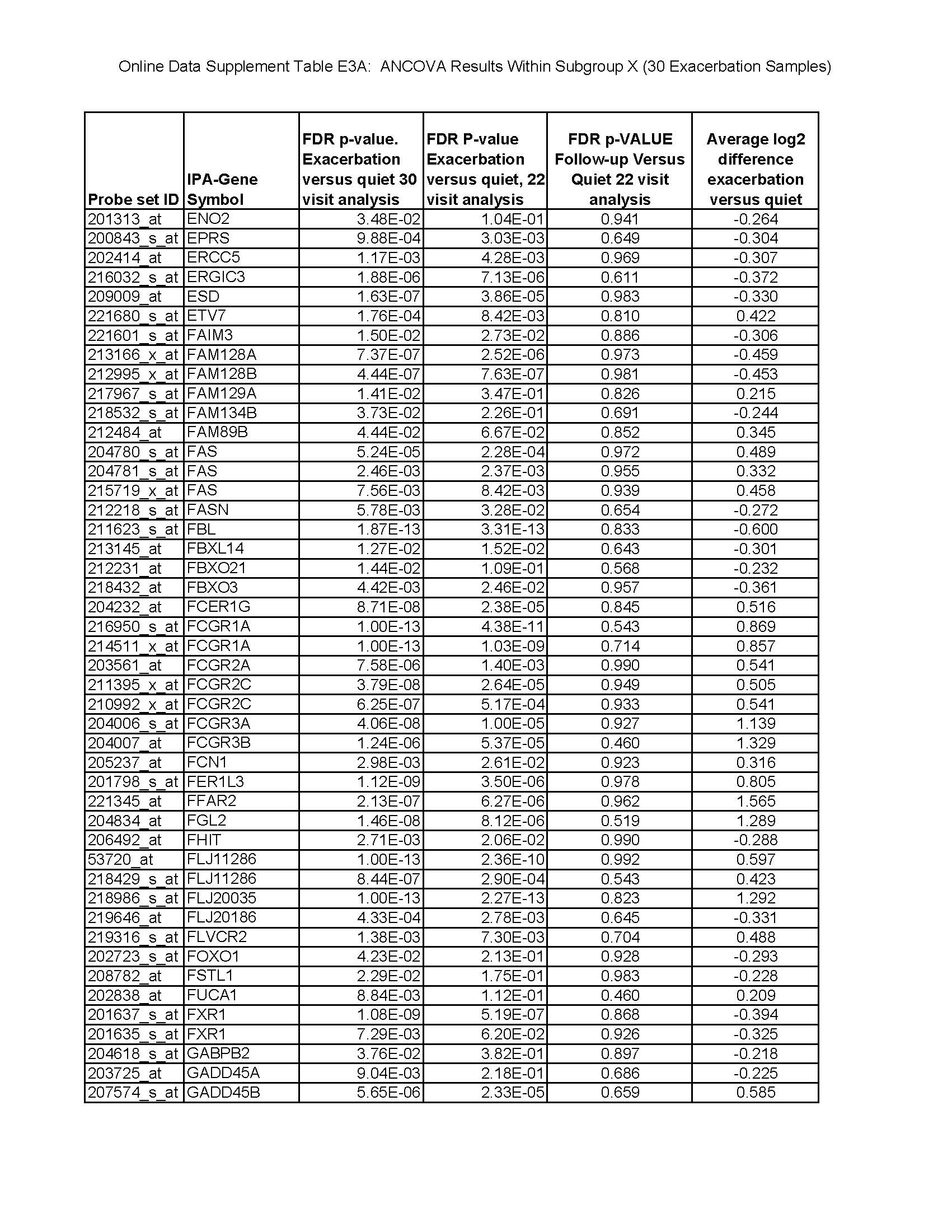


Table S18A: ANCOVA Results Subgroup X continued
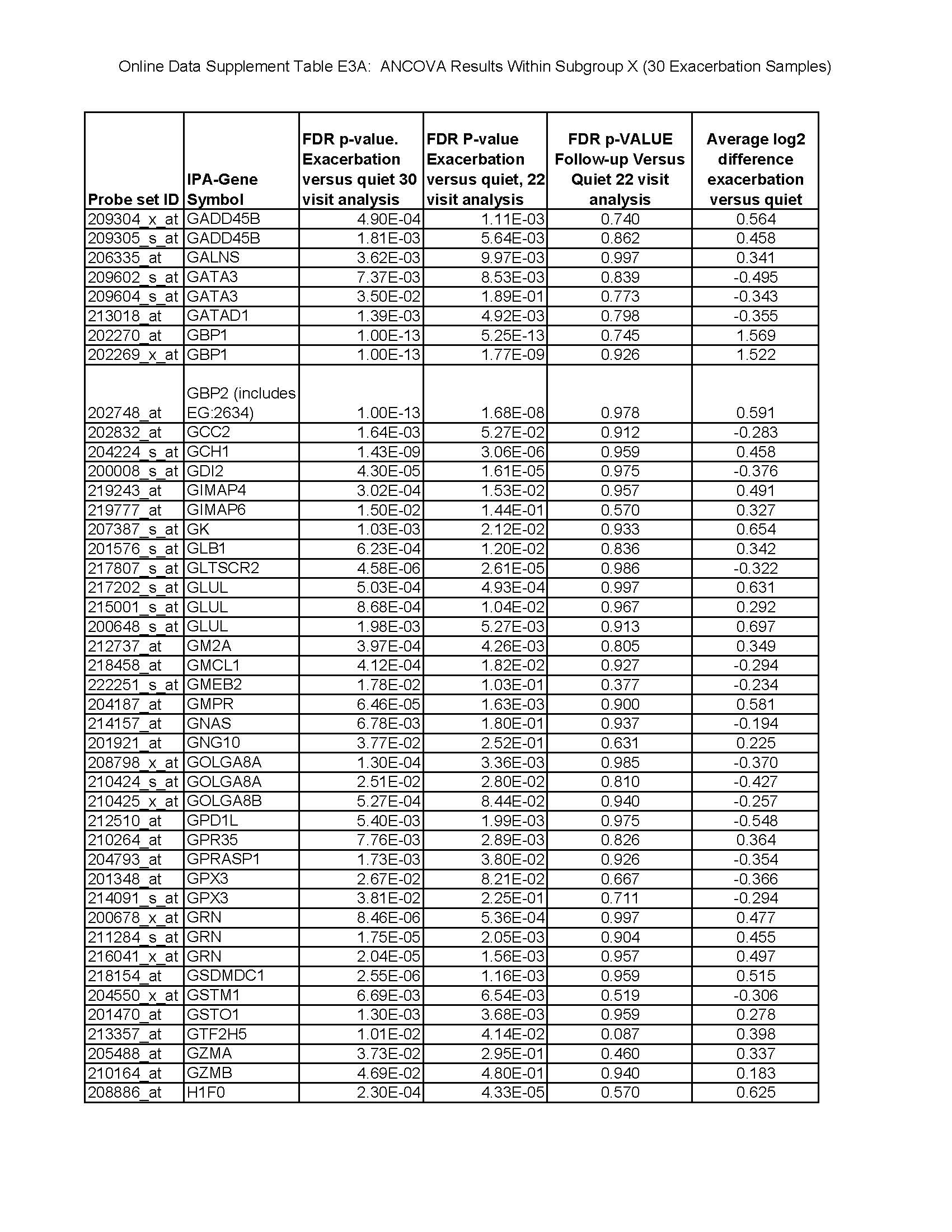


Table S18A: ANCOVA Results Subgroup X continued
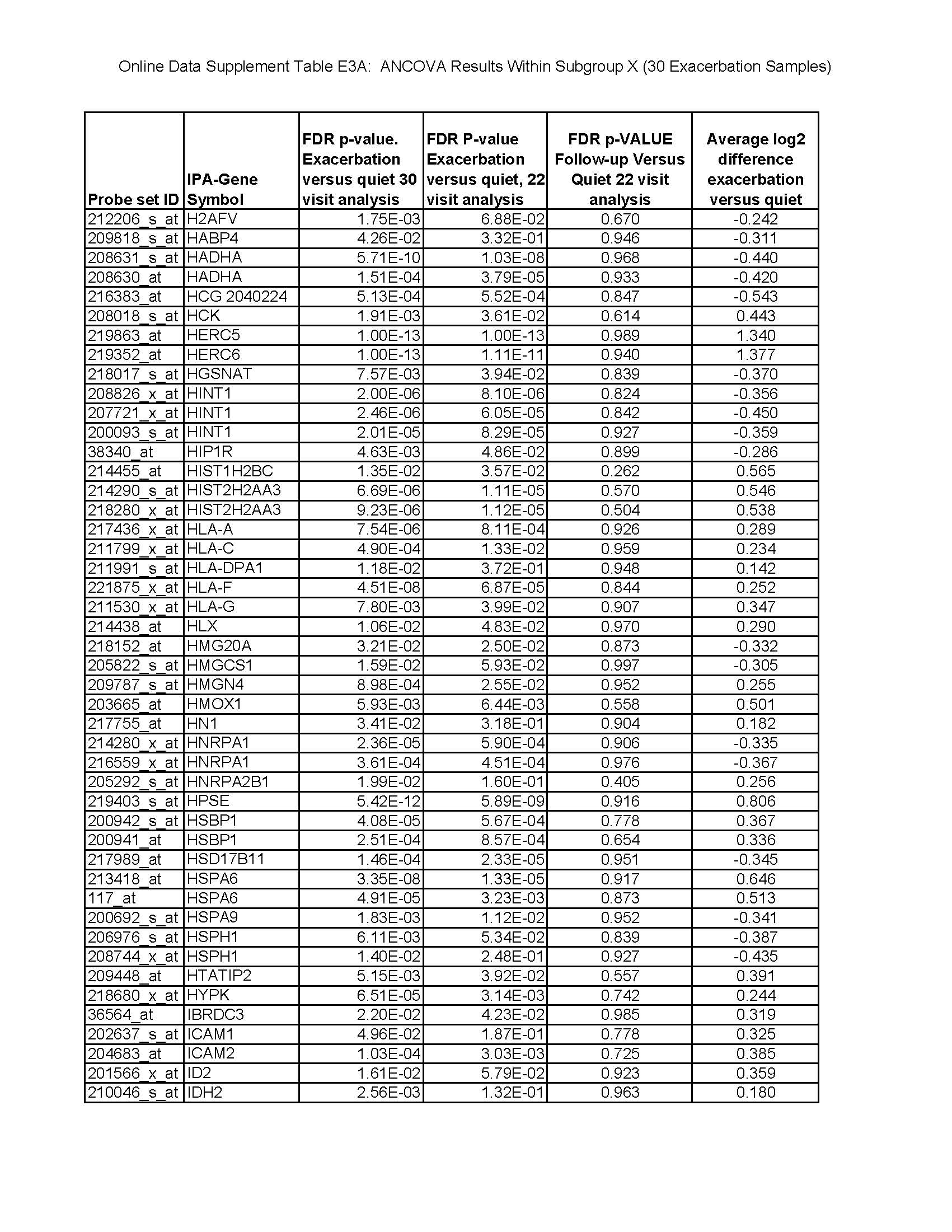


Table S18A: ANCOVA Results Subgroup X continued
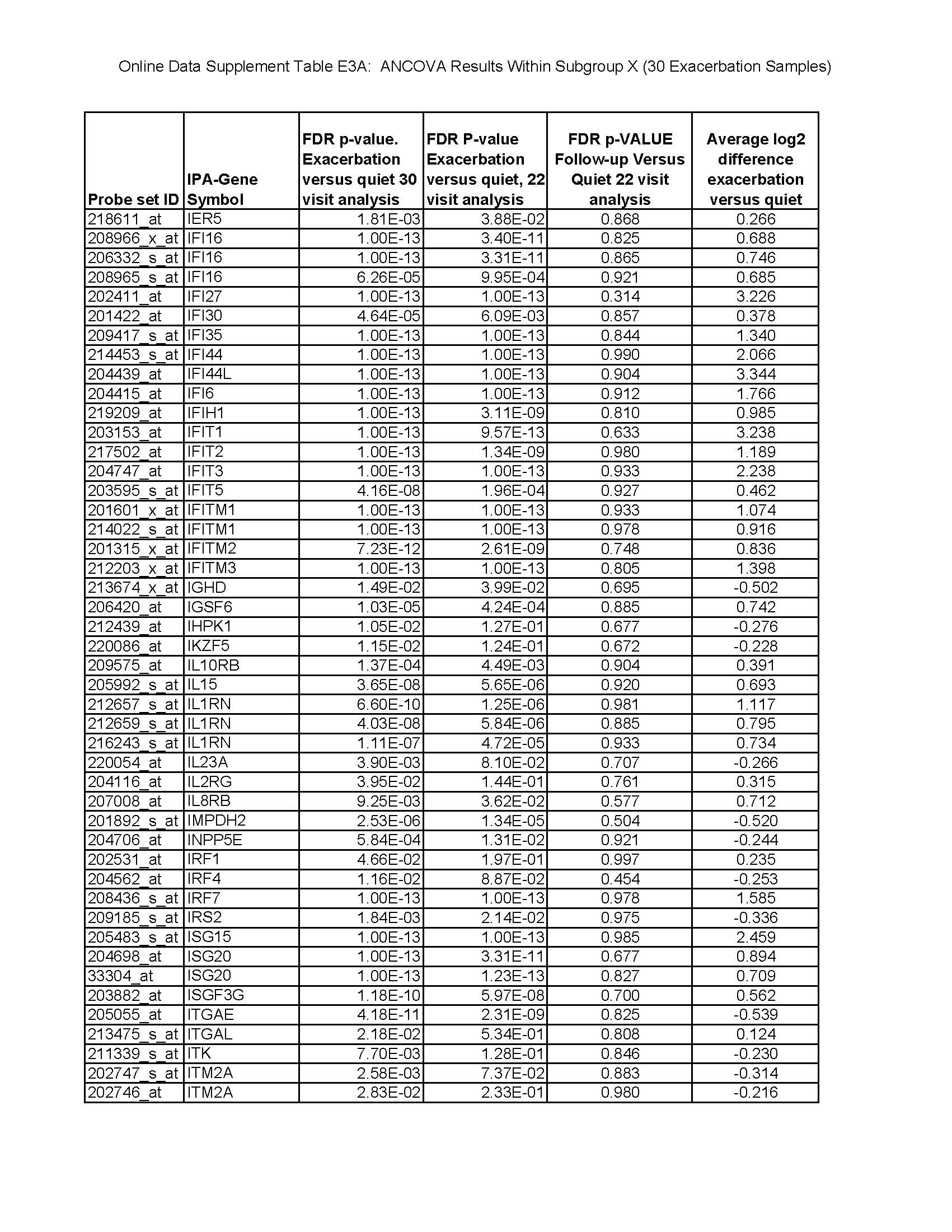


Table S18A: ANCOVA Results Subgroup X continued
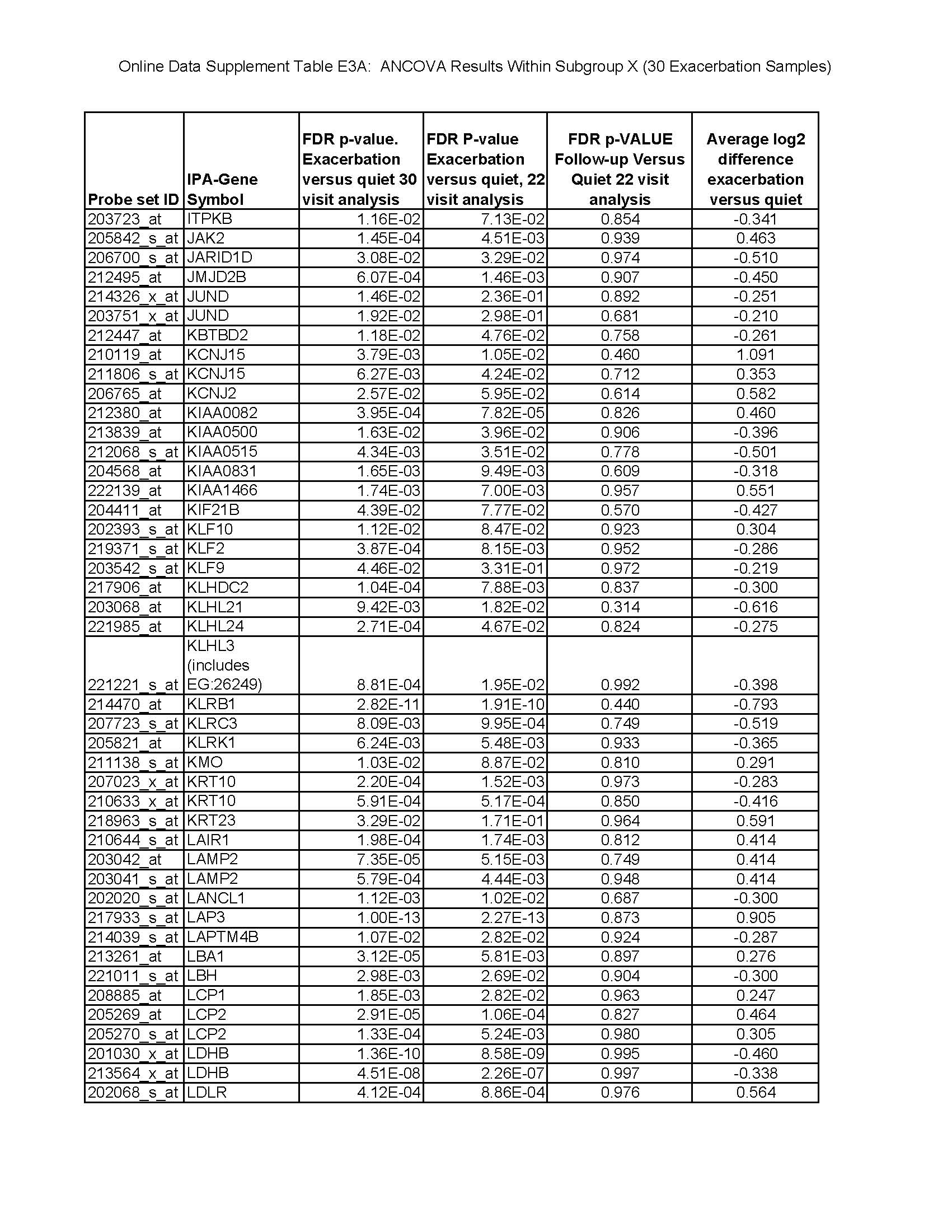


Table S18A: ANCOVA Results Subgroup X continued
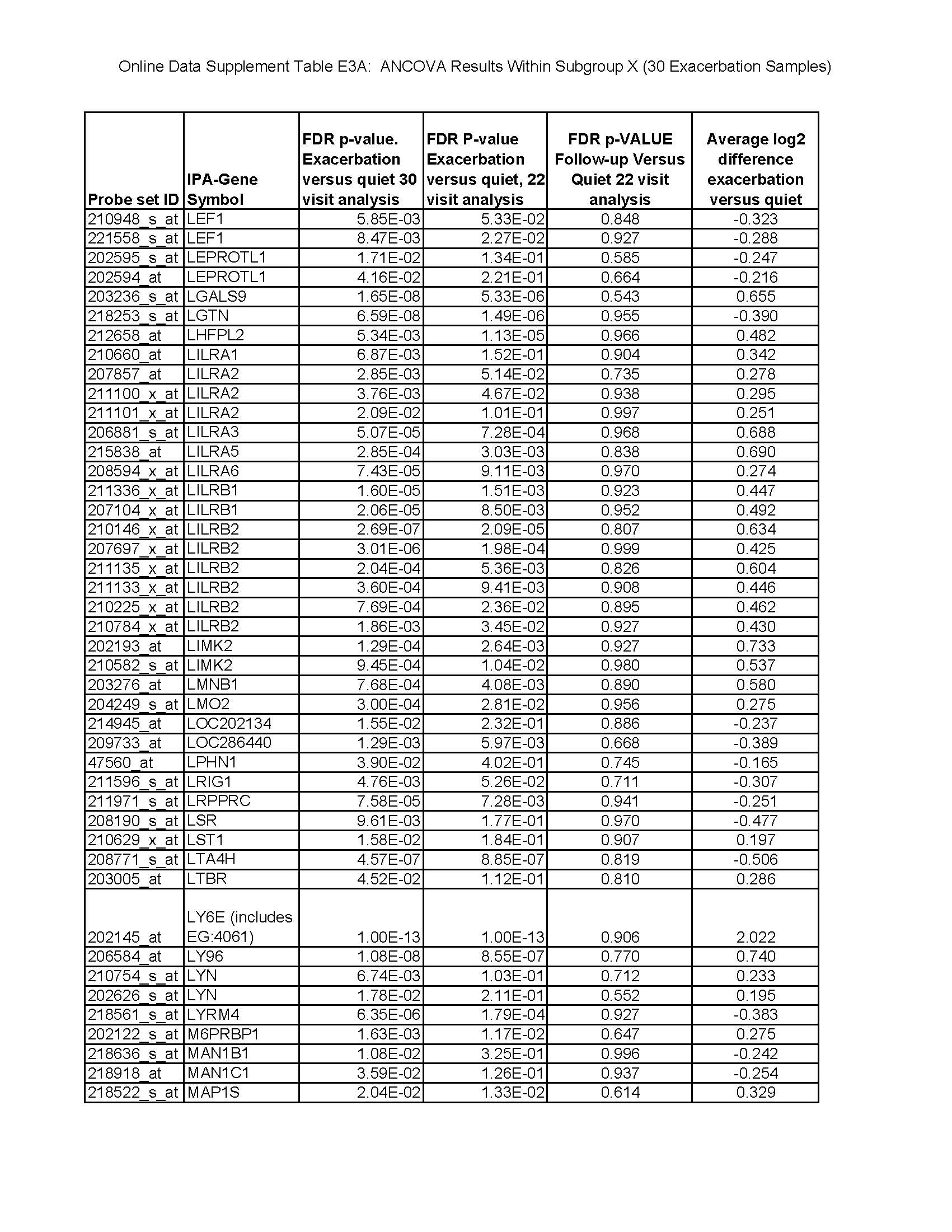


Table S18A: ANCOVA Results Subgroup X continued
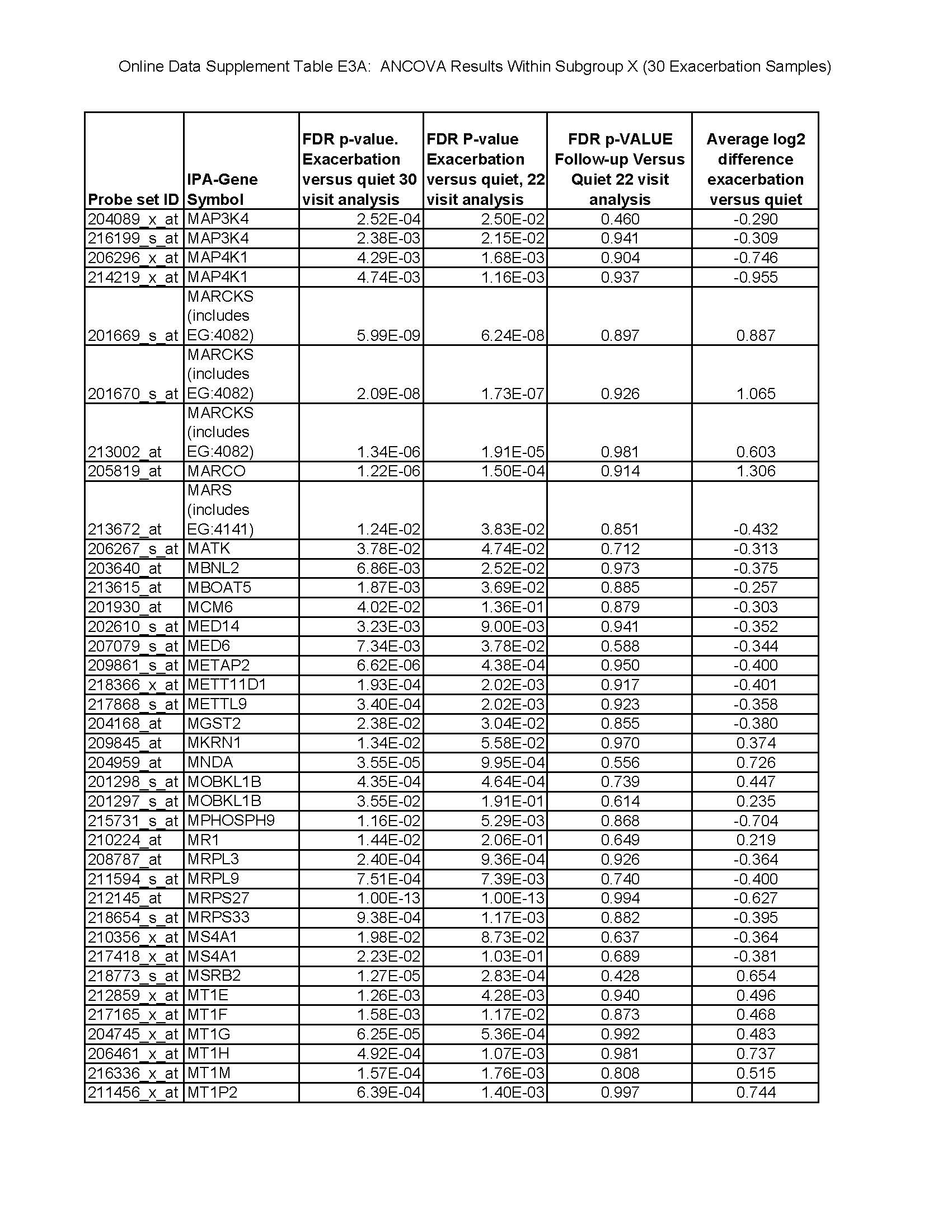
 Table S18A: ANCOVA Results Subgroup X continued
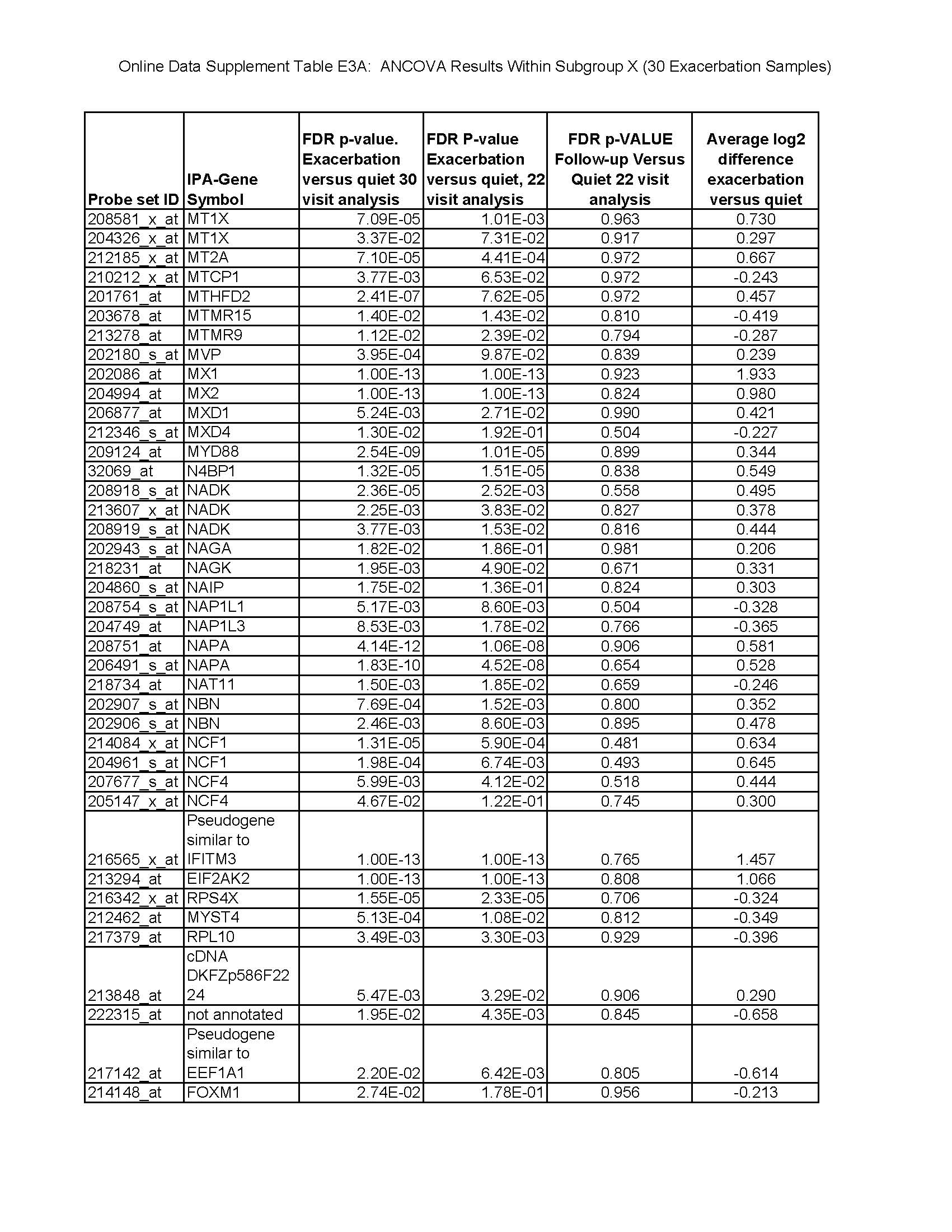


Table S18A: ANCOVA Results Subgroup X continued
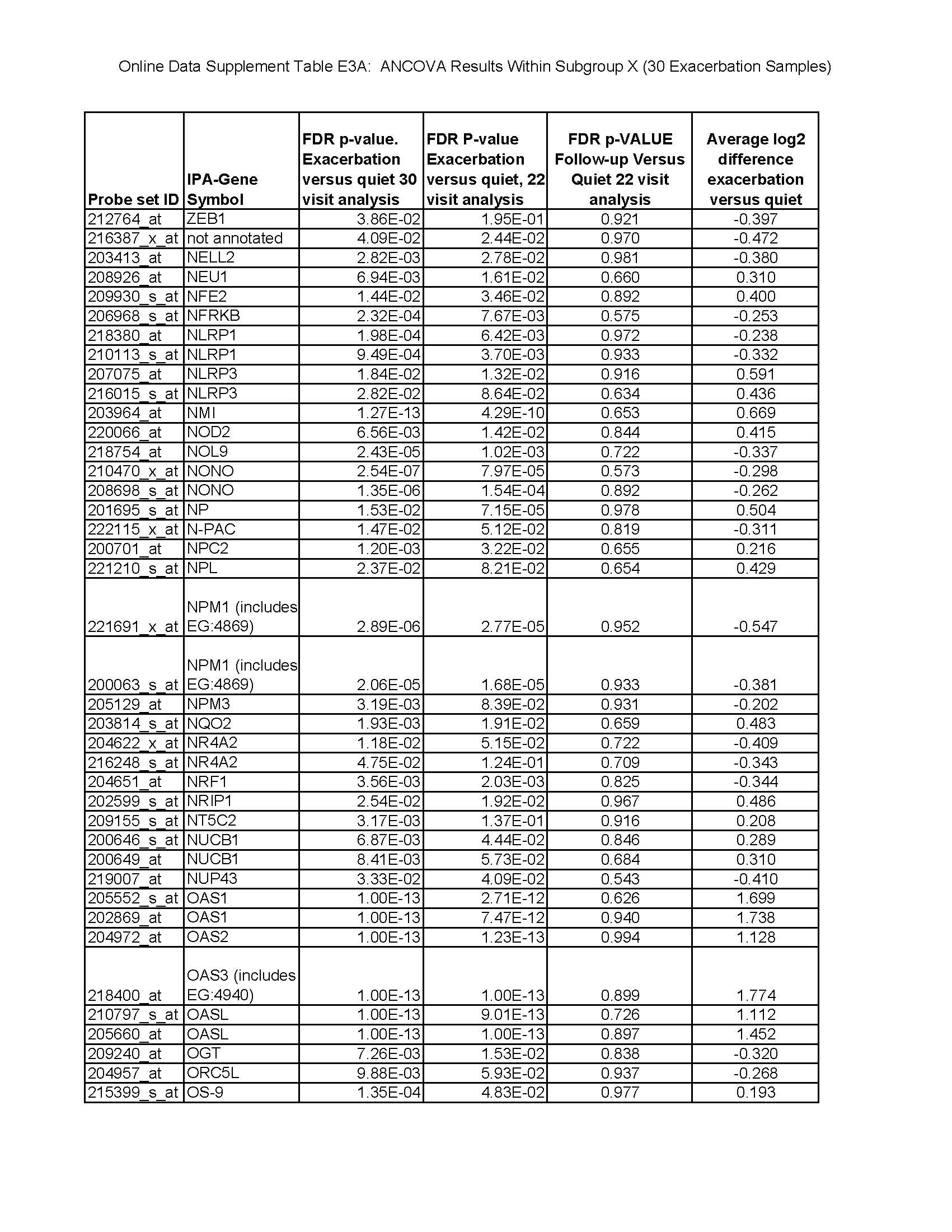


Table S18A: ANCOVA Results Subgroup X continued
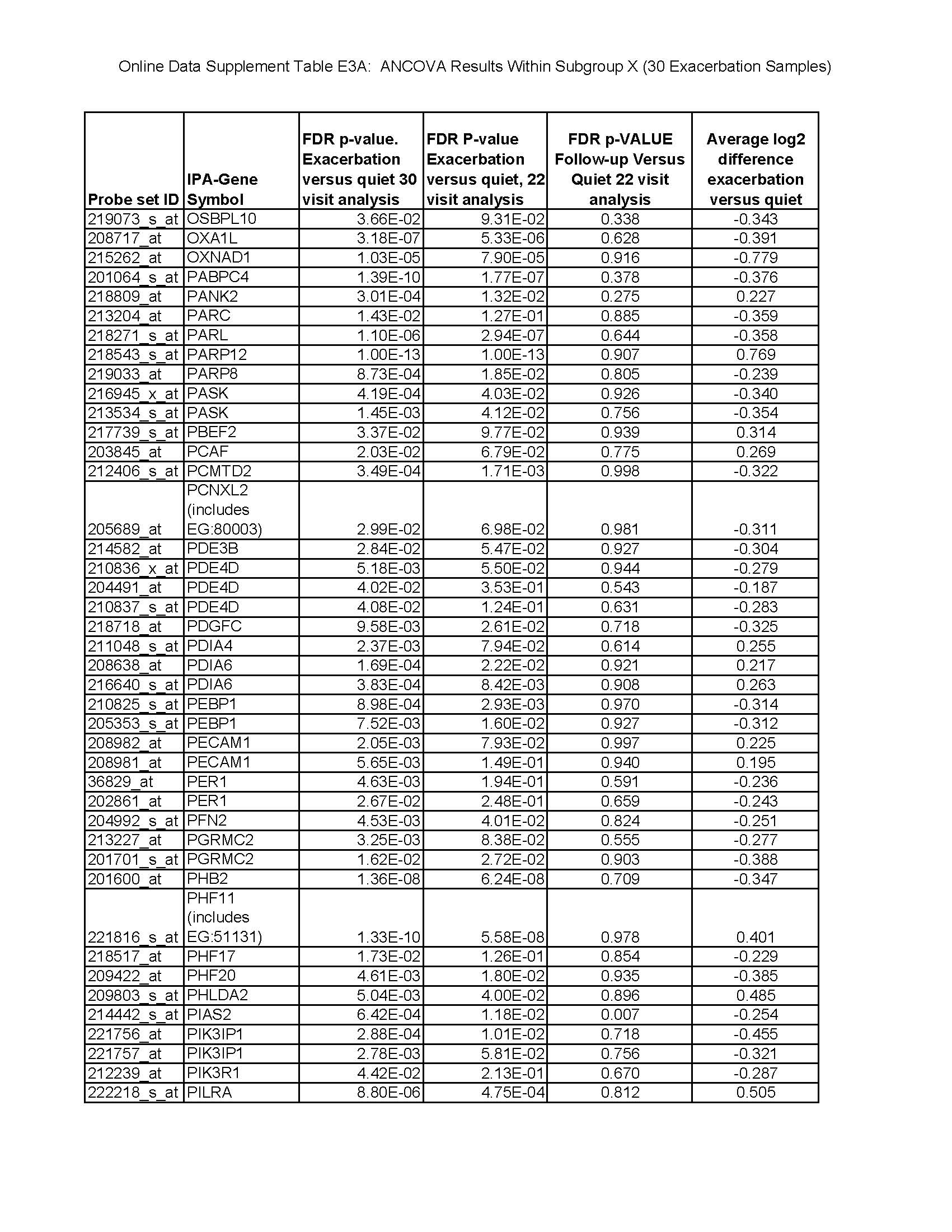


Table S18A: ANCOVA Results Subgroup X continued
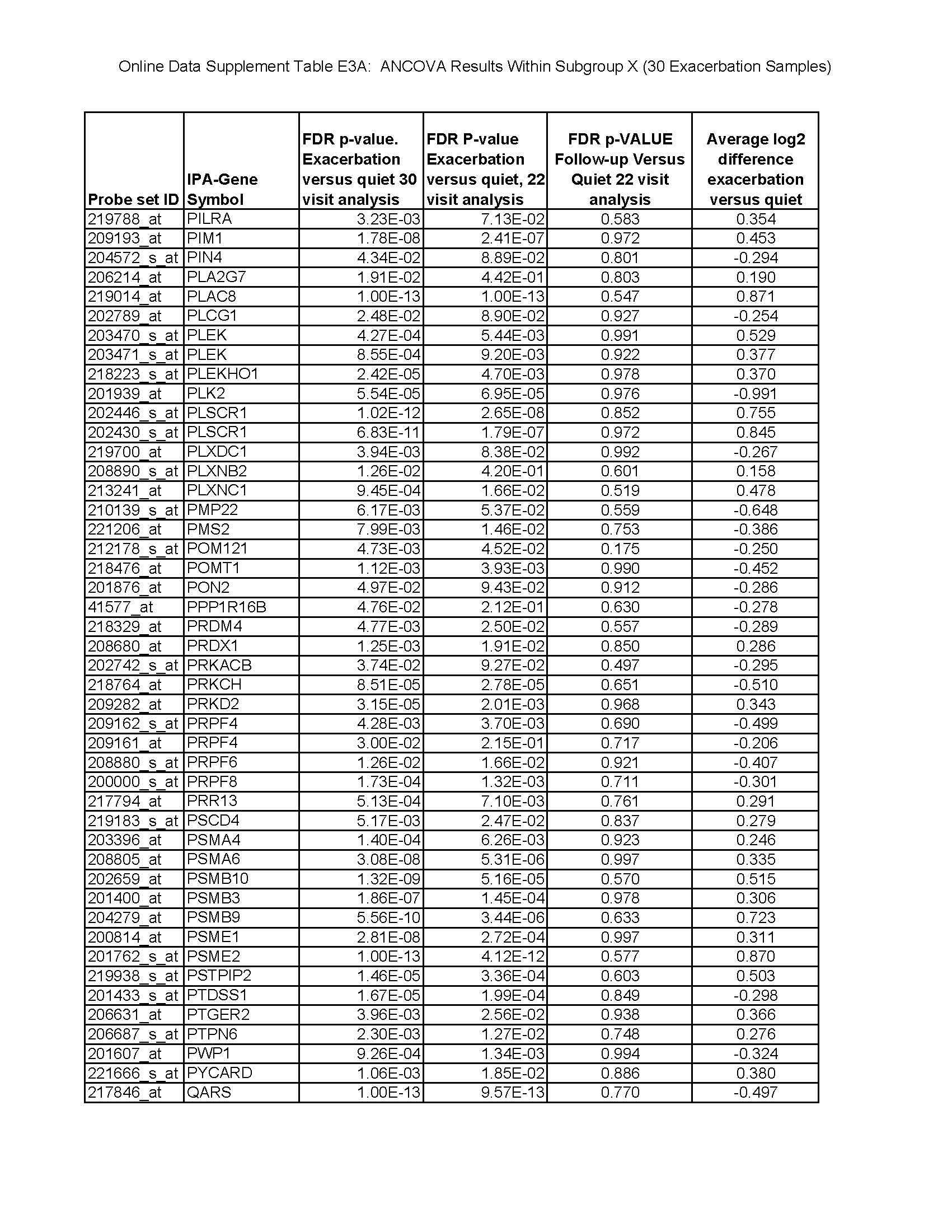


Table S18A: ANCOVA Results Subgroup X continued
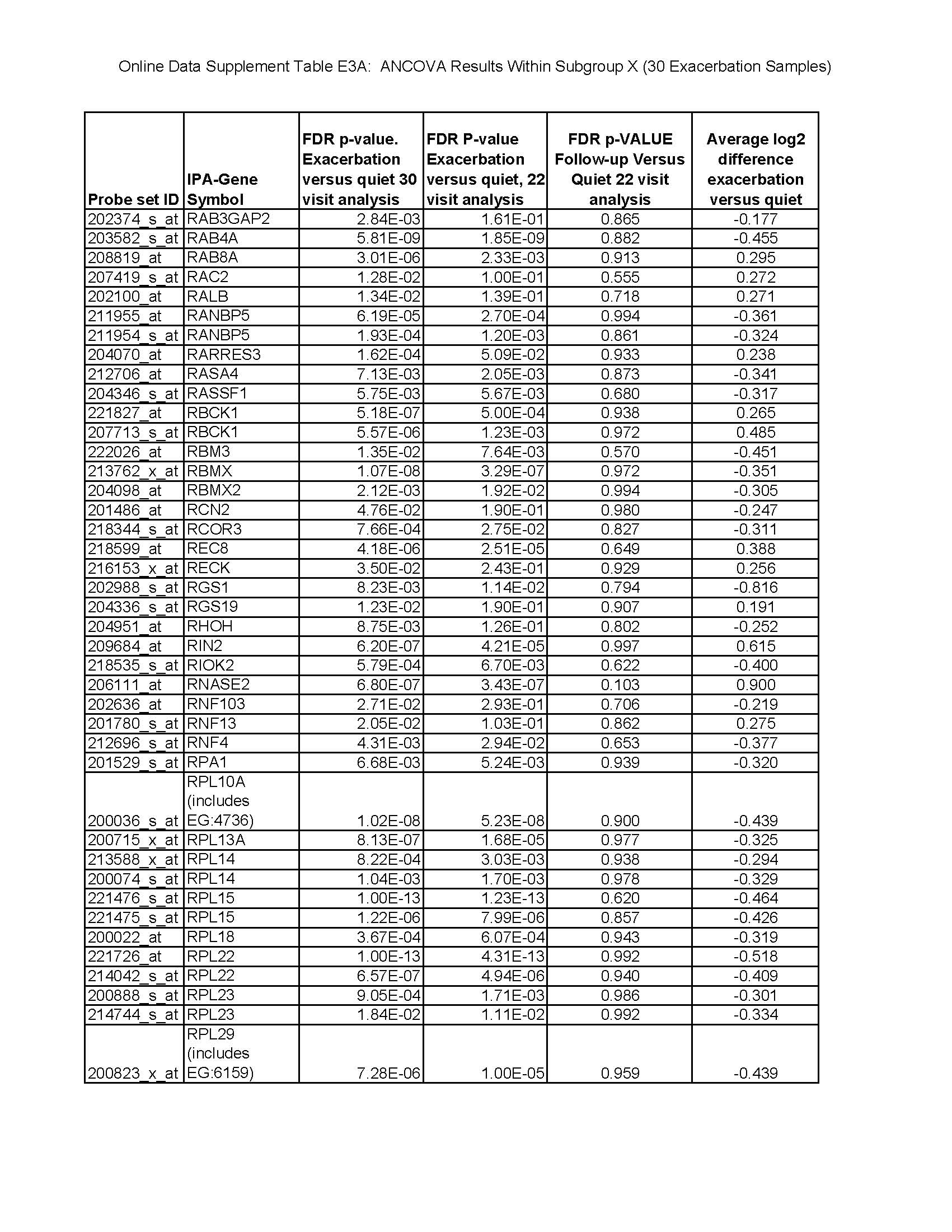


Table S18A: ANCOVA Results Subgroup X continued
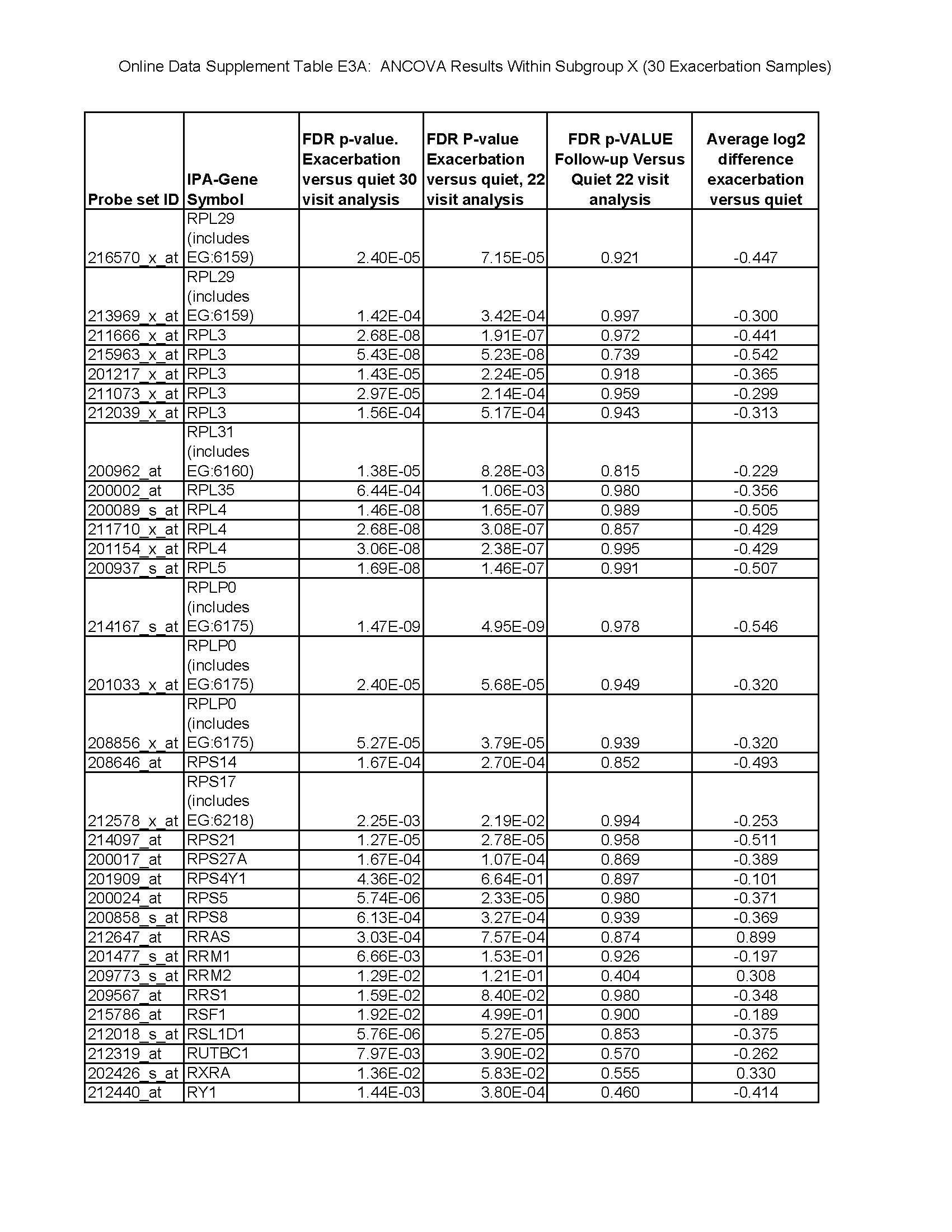


Table S18A: ANCOVA Results Subgroup X continued
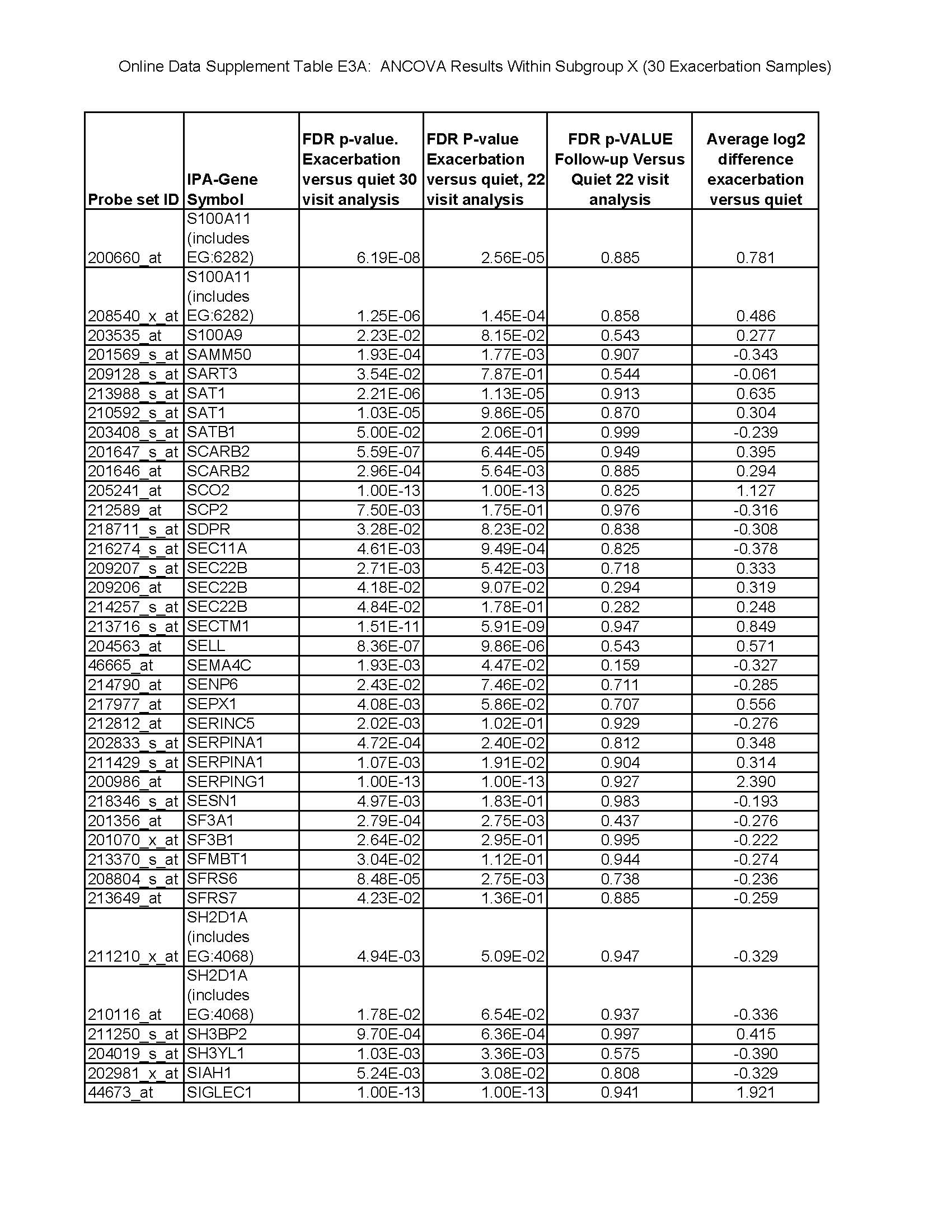


Table S18A: ANCOVA Results Subgroup X continued
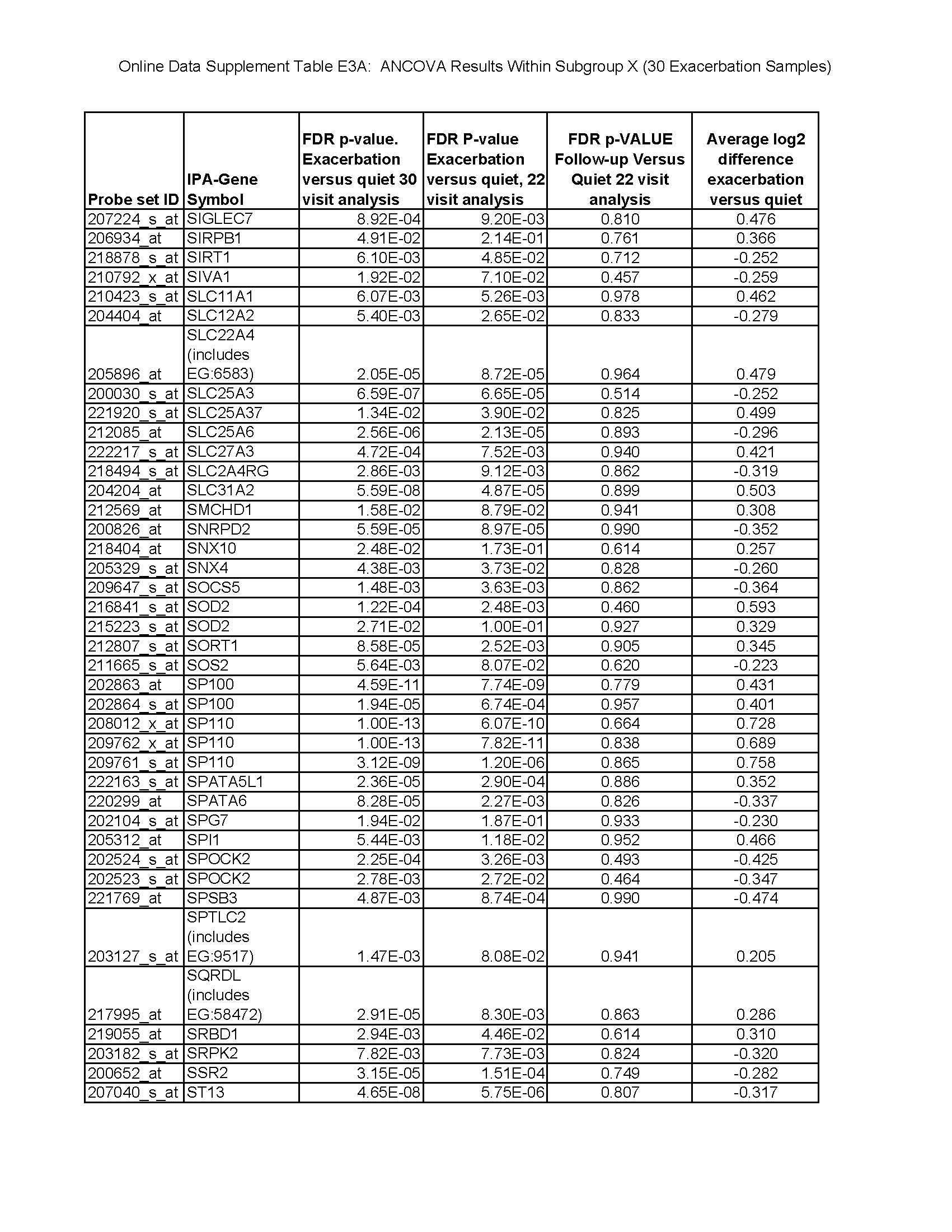


Table S18A: ANCOVA Results Subgroup X continued
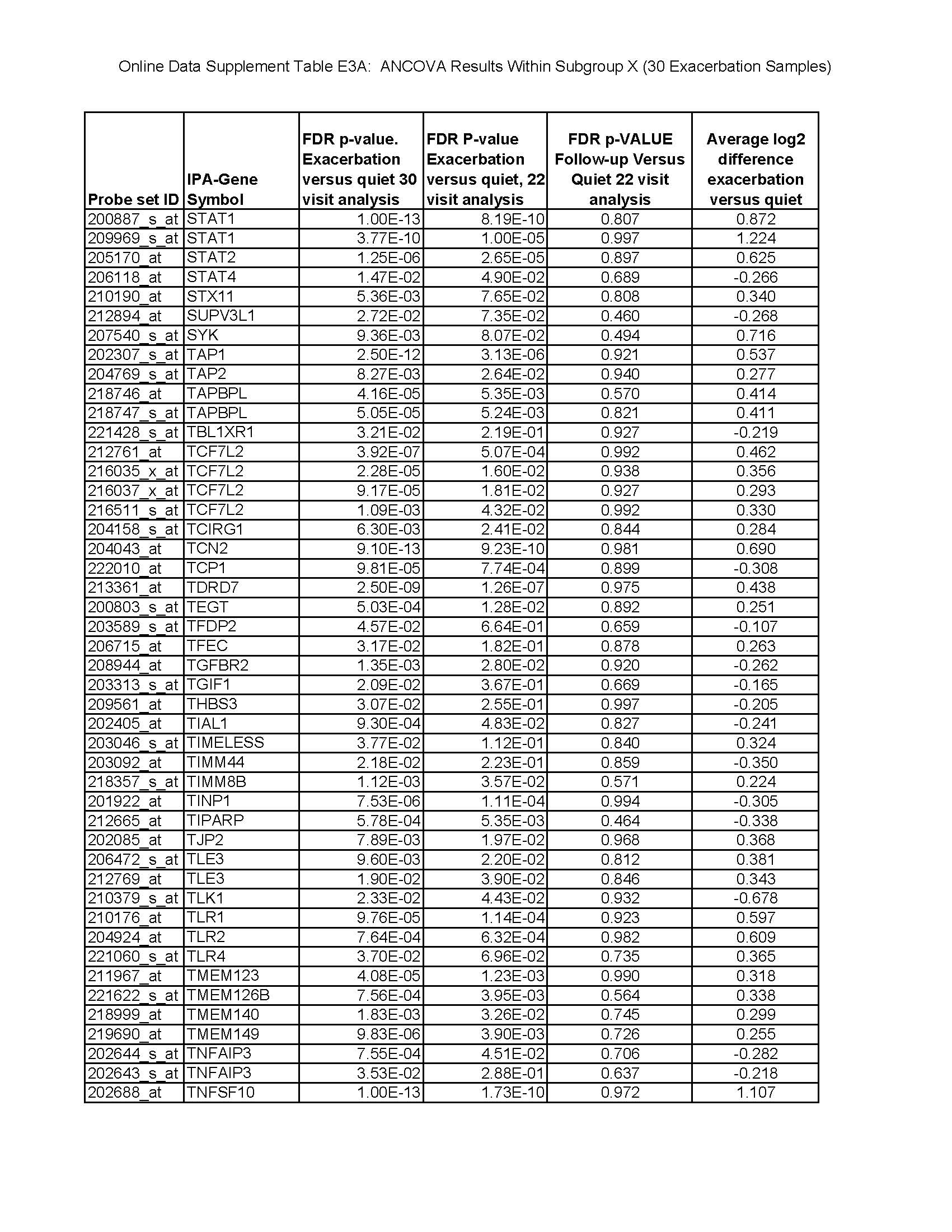


Table S18A: ANCOVA Results Subgroup X continued
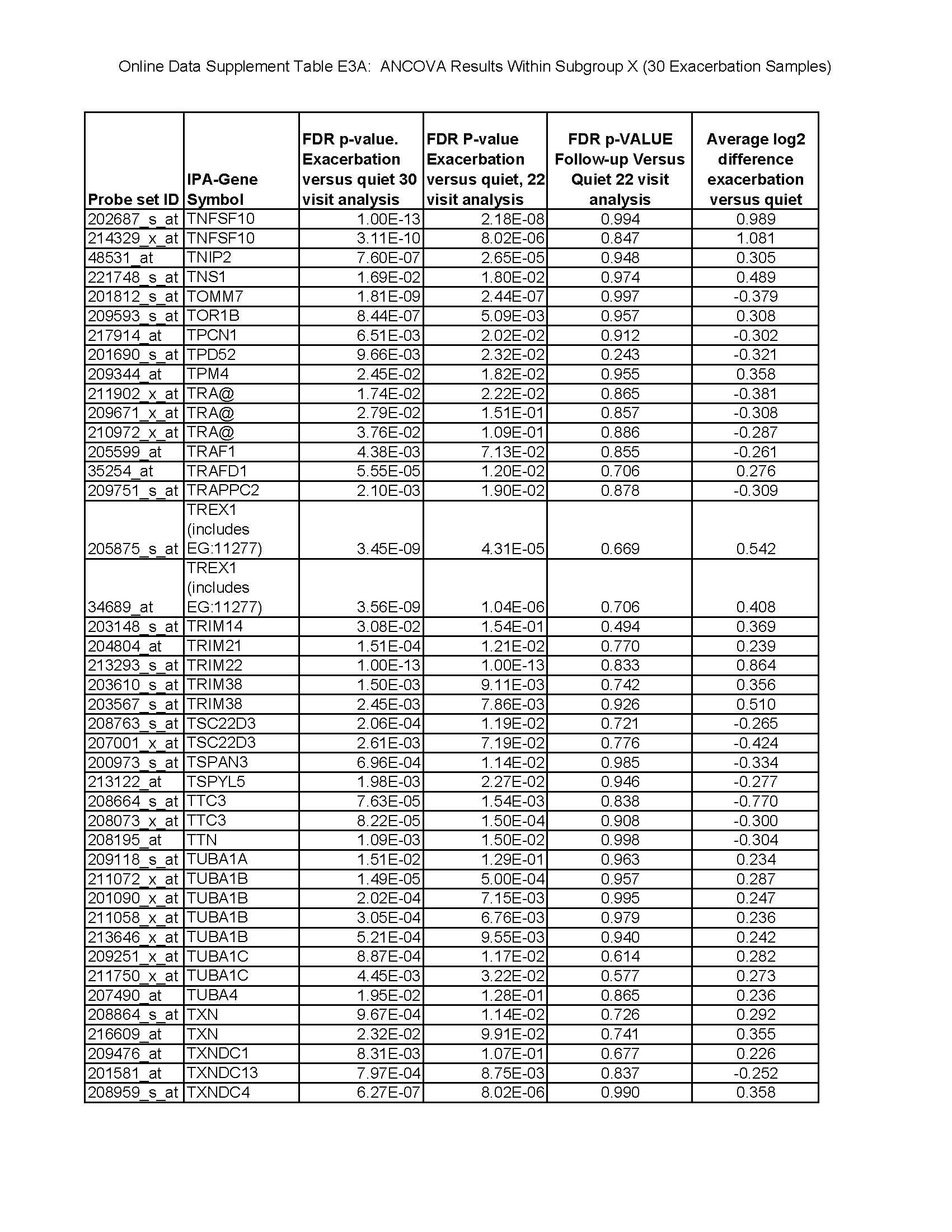


Table S18A: ANCOVA Results Subgroup X continued
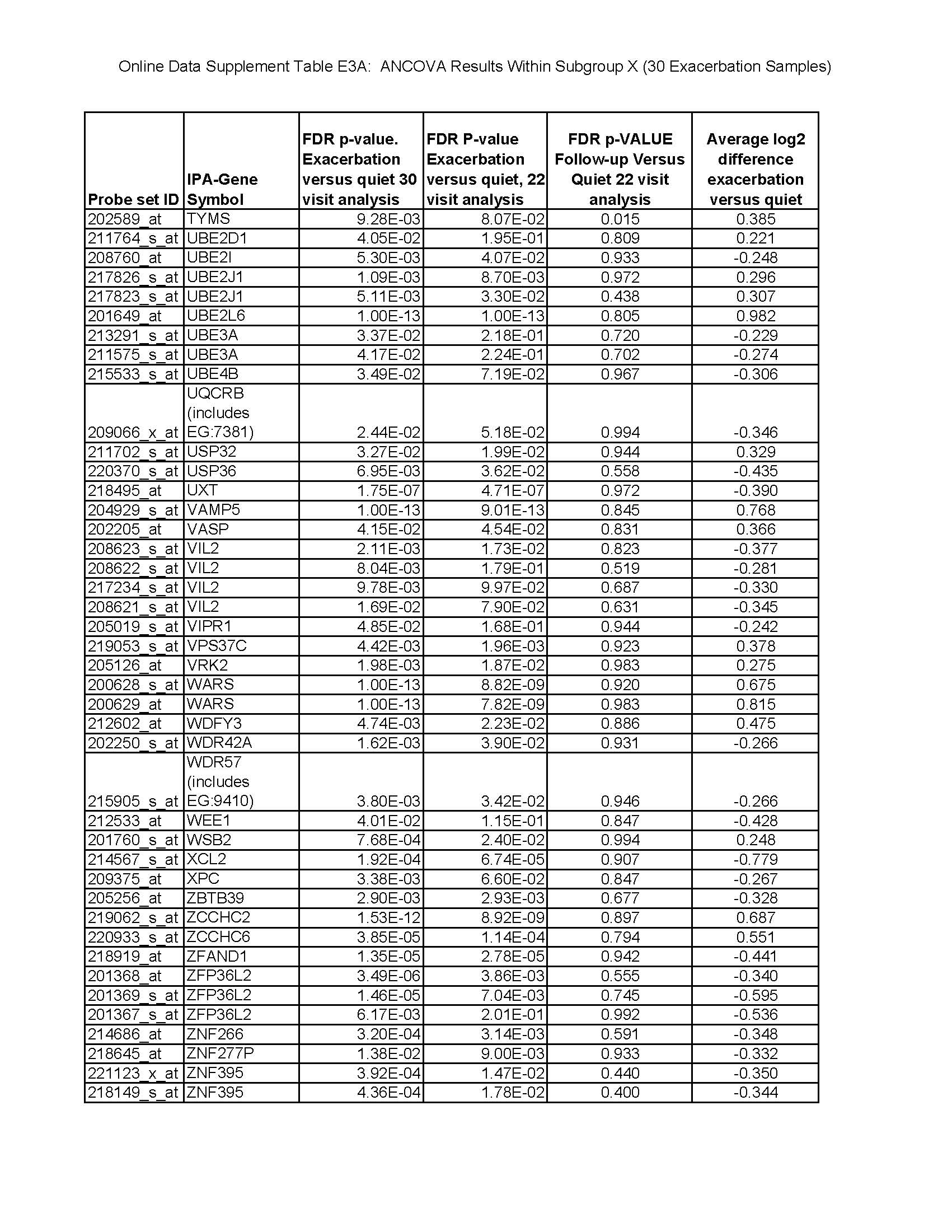

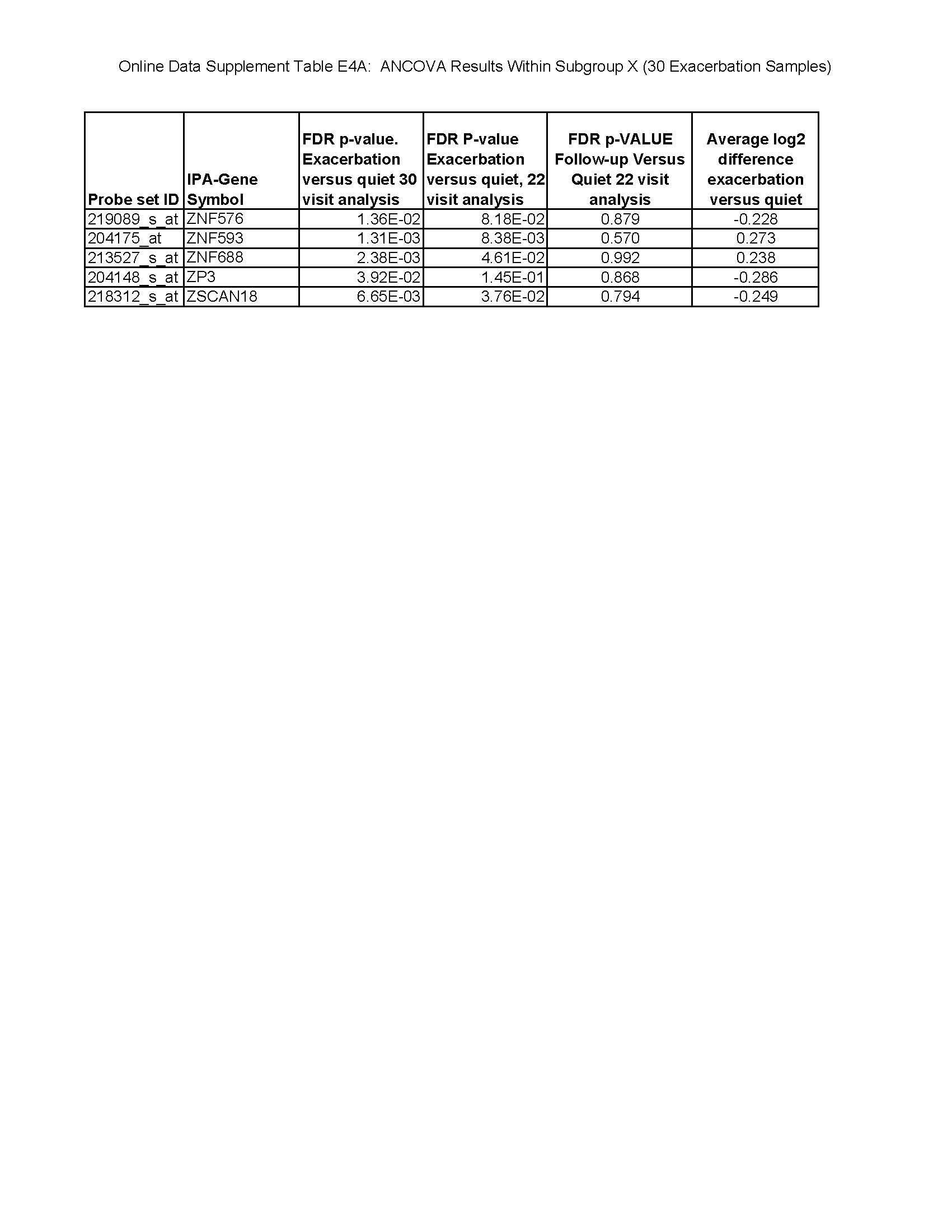


## Table S18B: ANCOVA Results Subgroup Y Samples


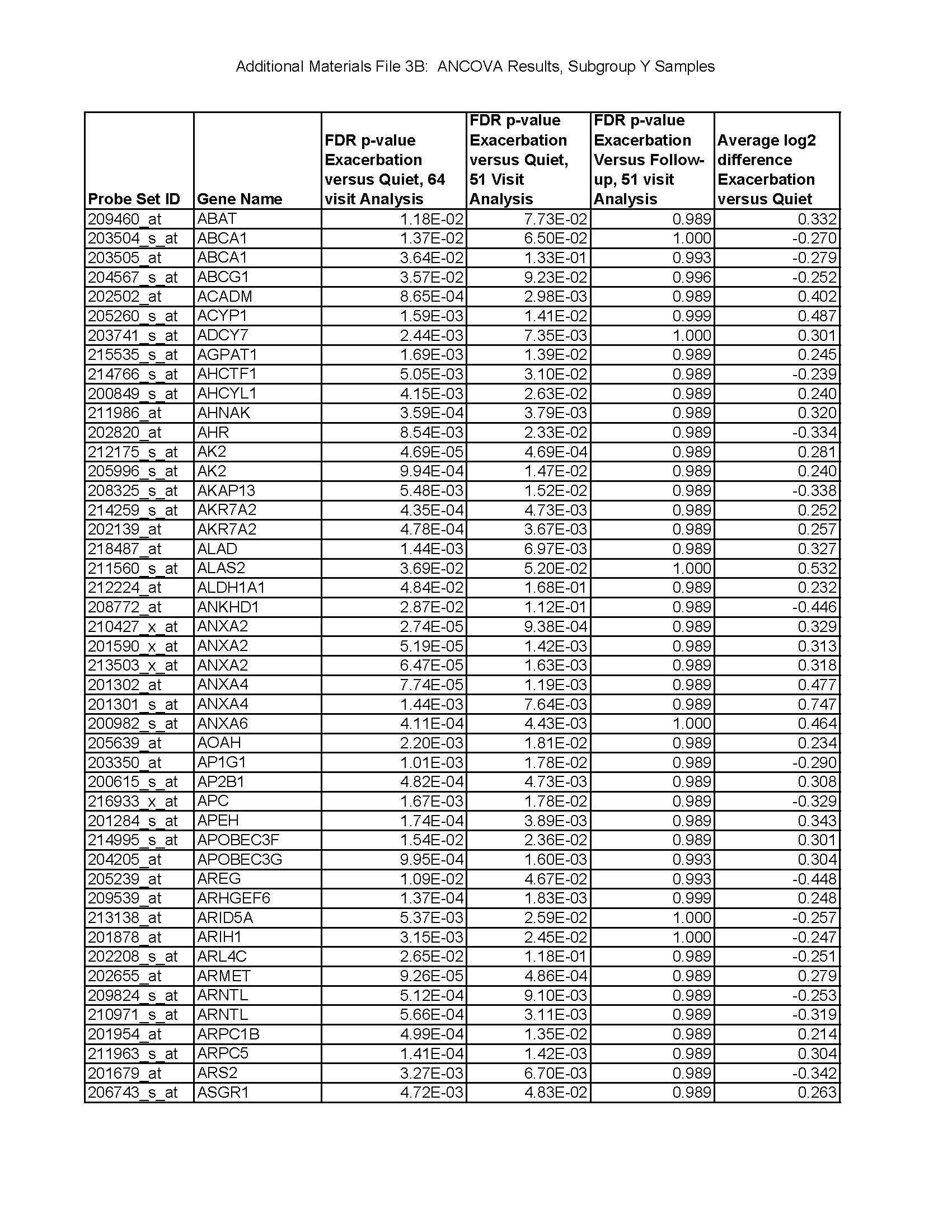


Table S18B: ANCOVA Results Subgroup Y Samples


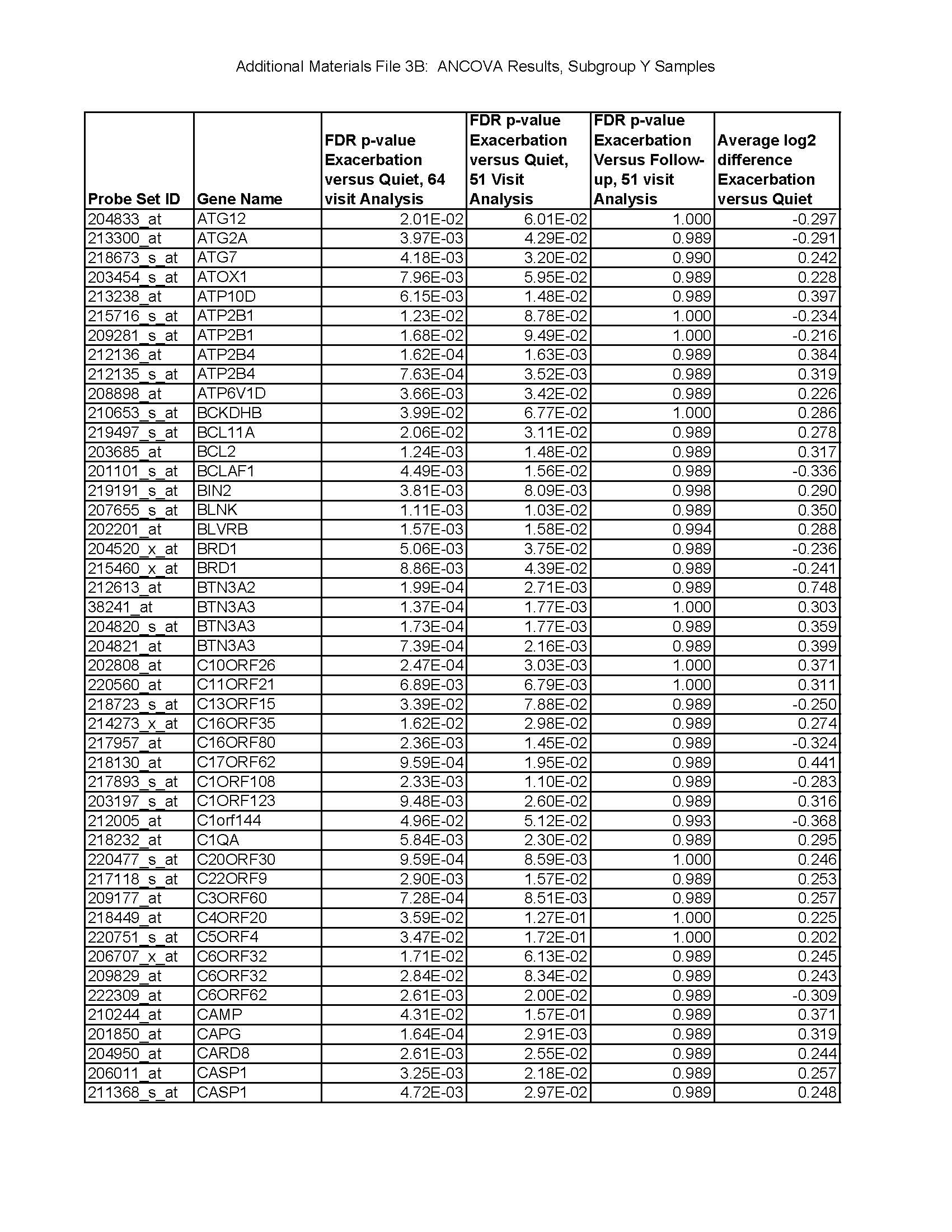


Table S18B: ANCOVA Results Subgroup Y continued
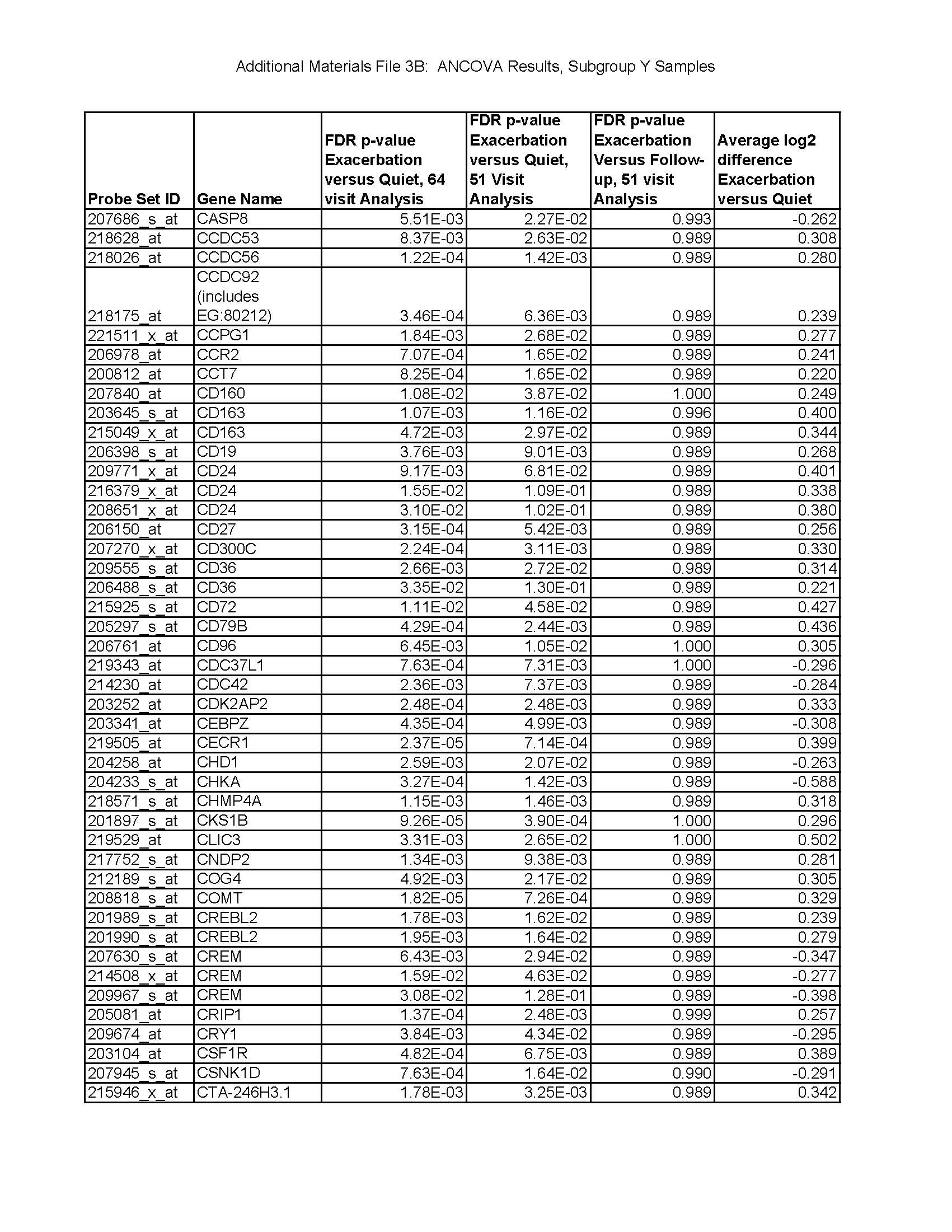


Table S18B: ANCOVA Results Subgroup Y continued
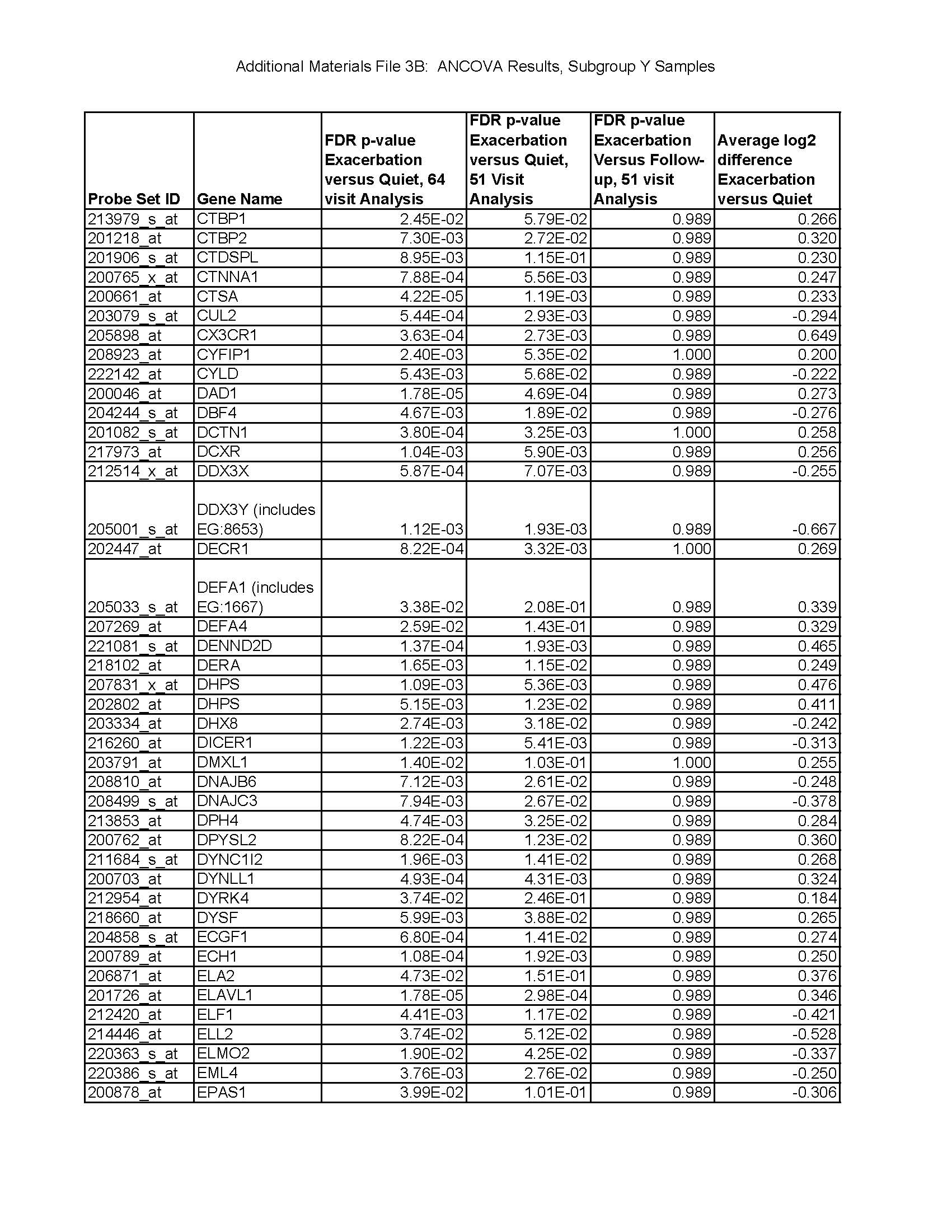


Table S18B: ANCOVA Results Subgroup Y continued
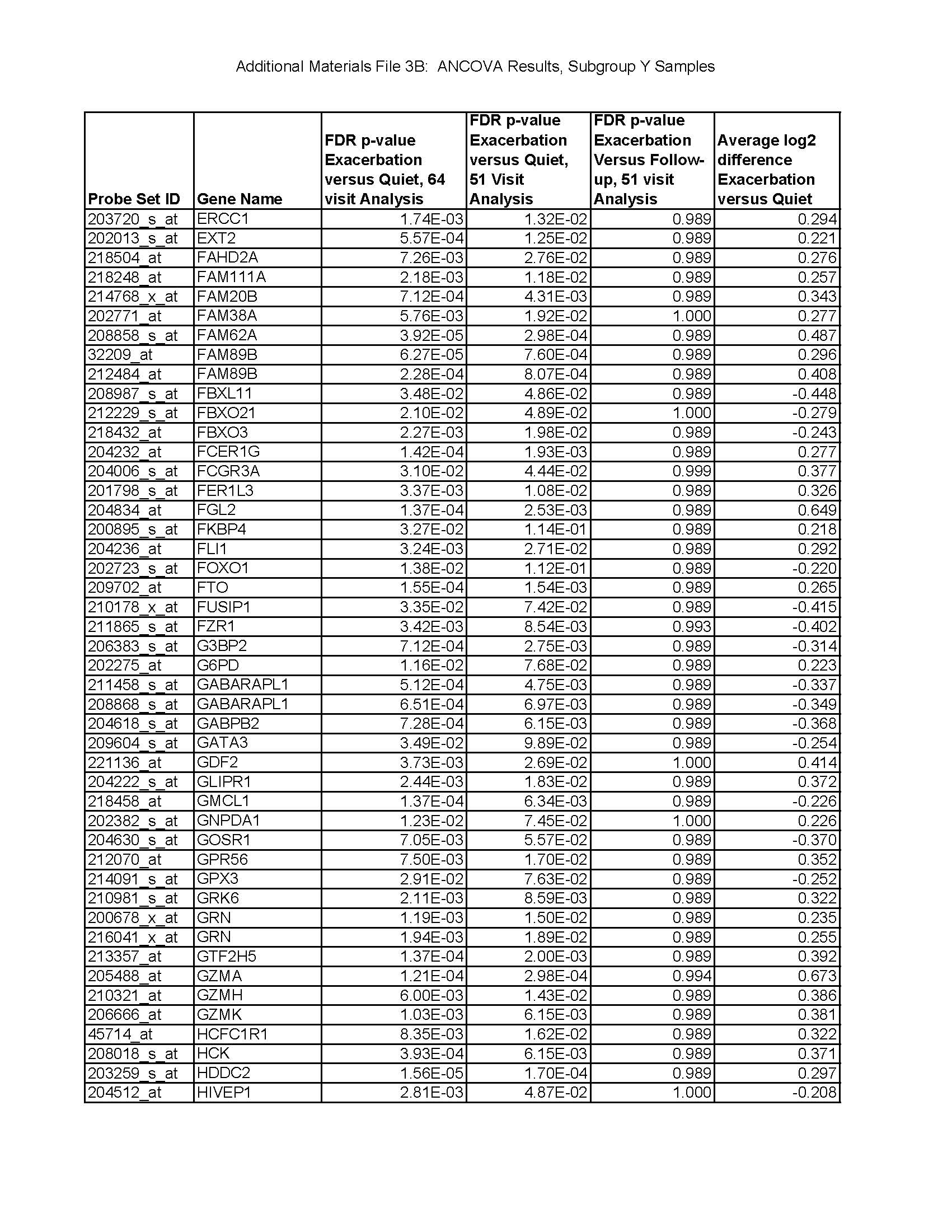


Table S18B: ANCOVA Results Subgroup Y continued
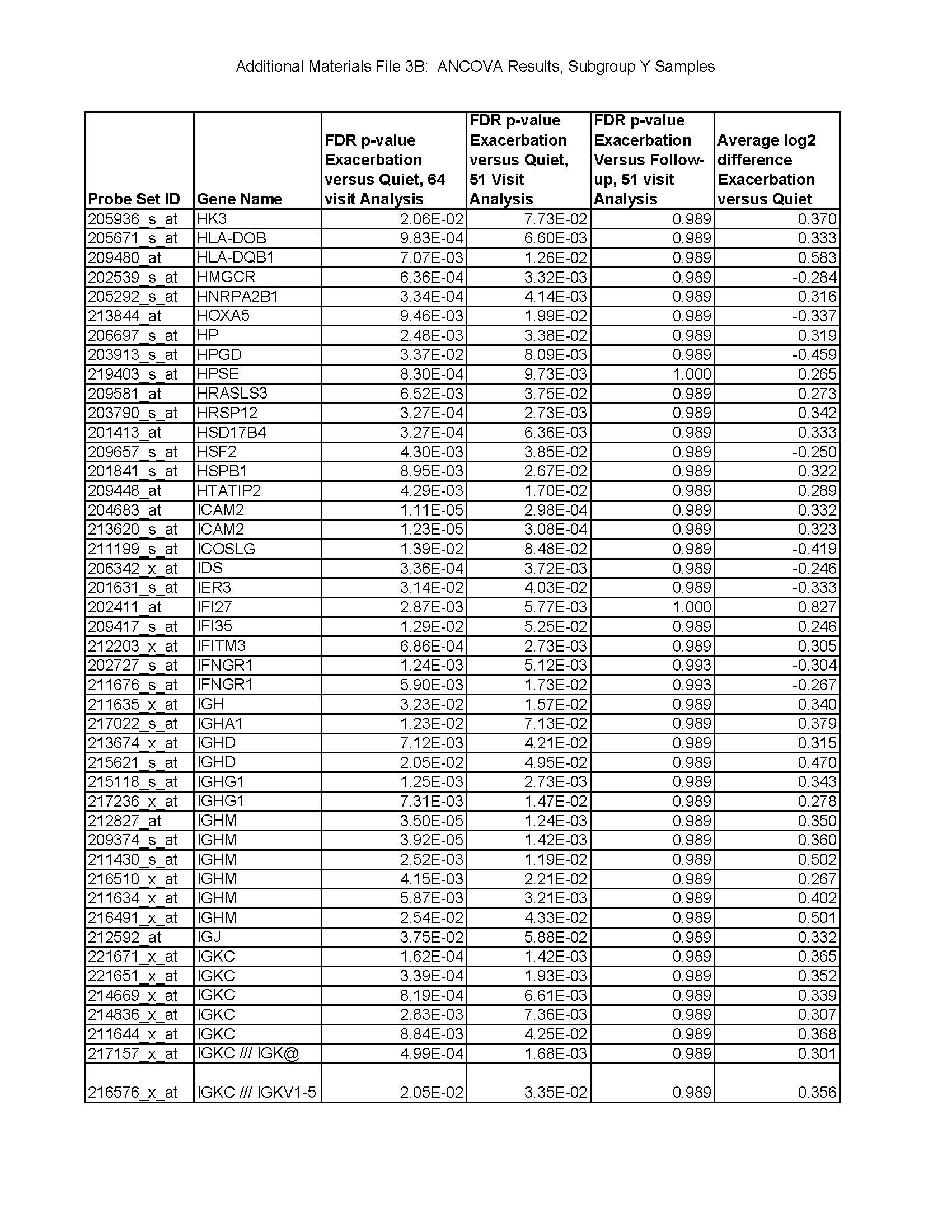


Table S18B: ANCOVA Results Subgroup Y continued
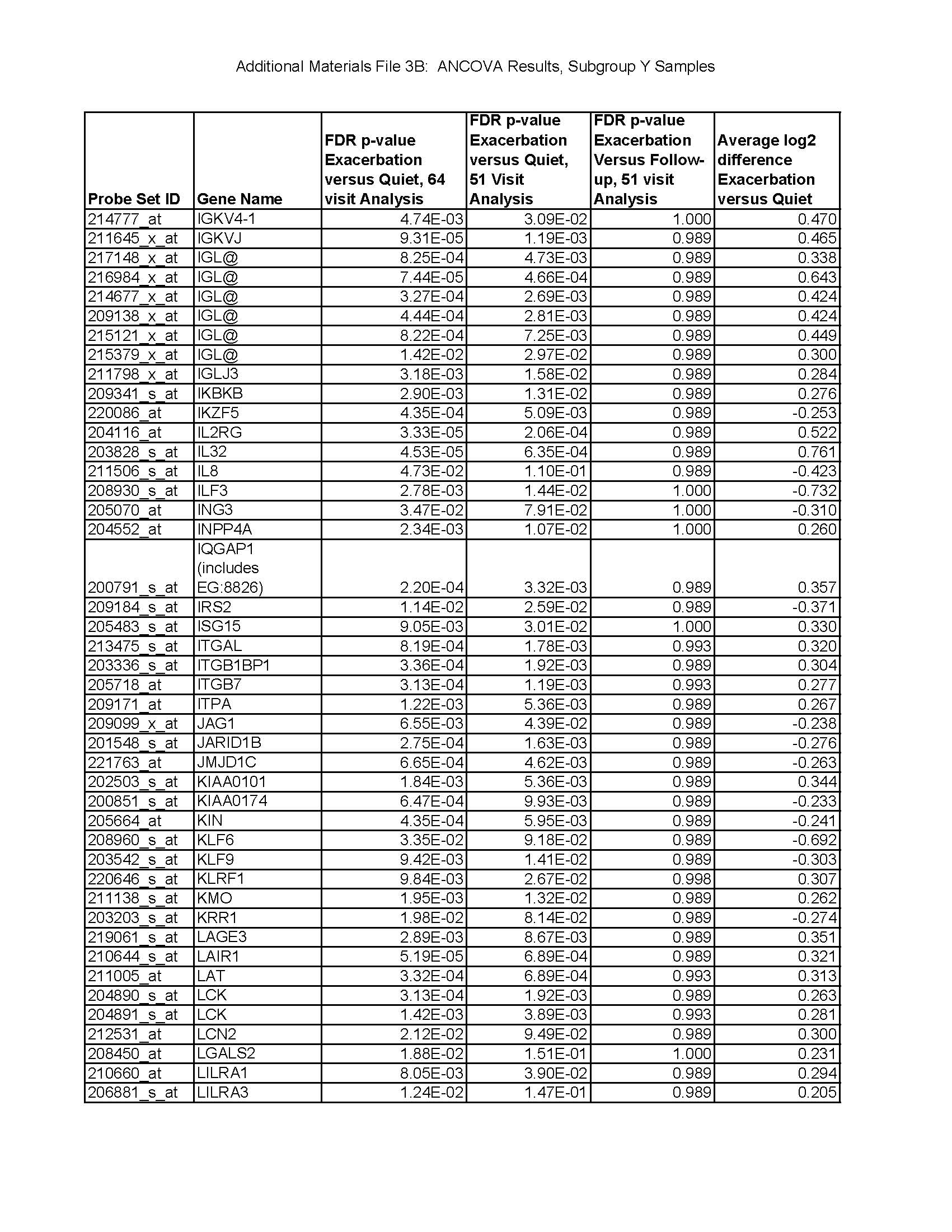


Table S18B: ANCOVA Results Subgroup Y continued
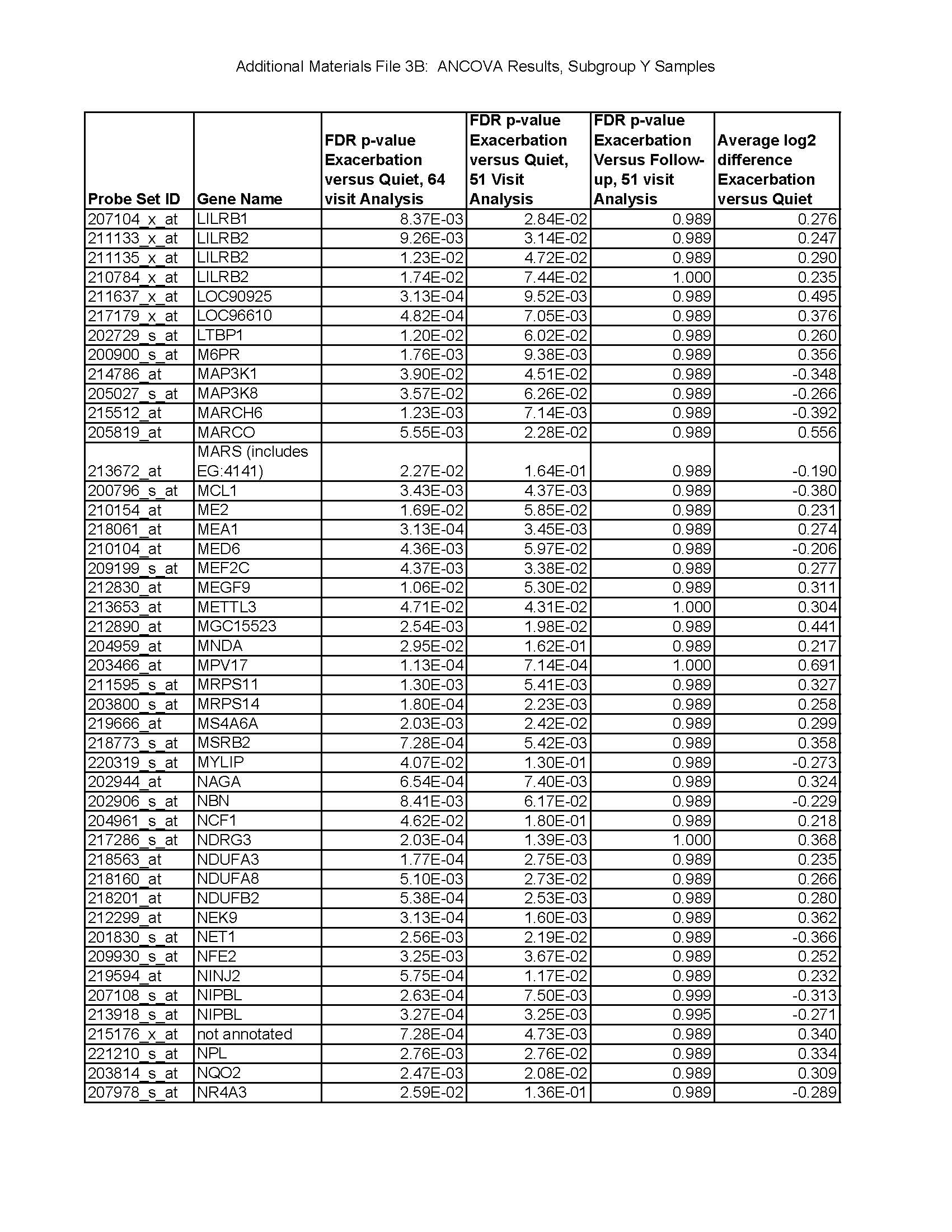


Table S18B: ANCOVA Results Subgroup Y continued
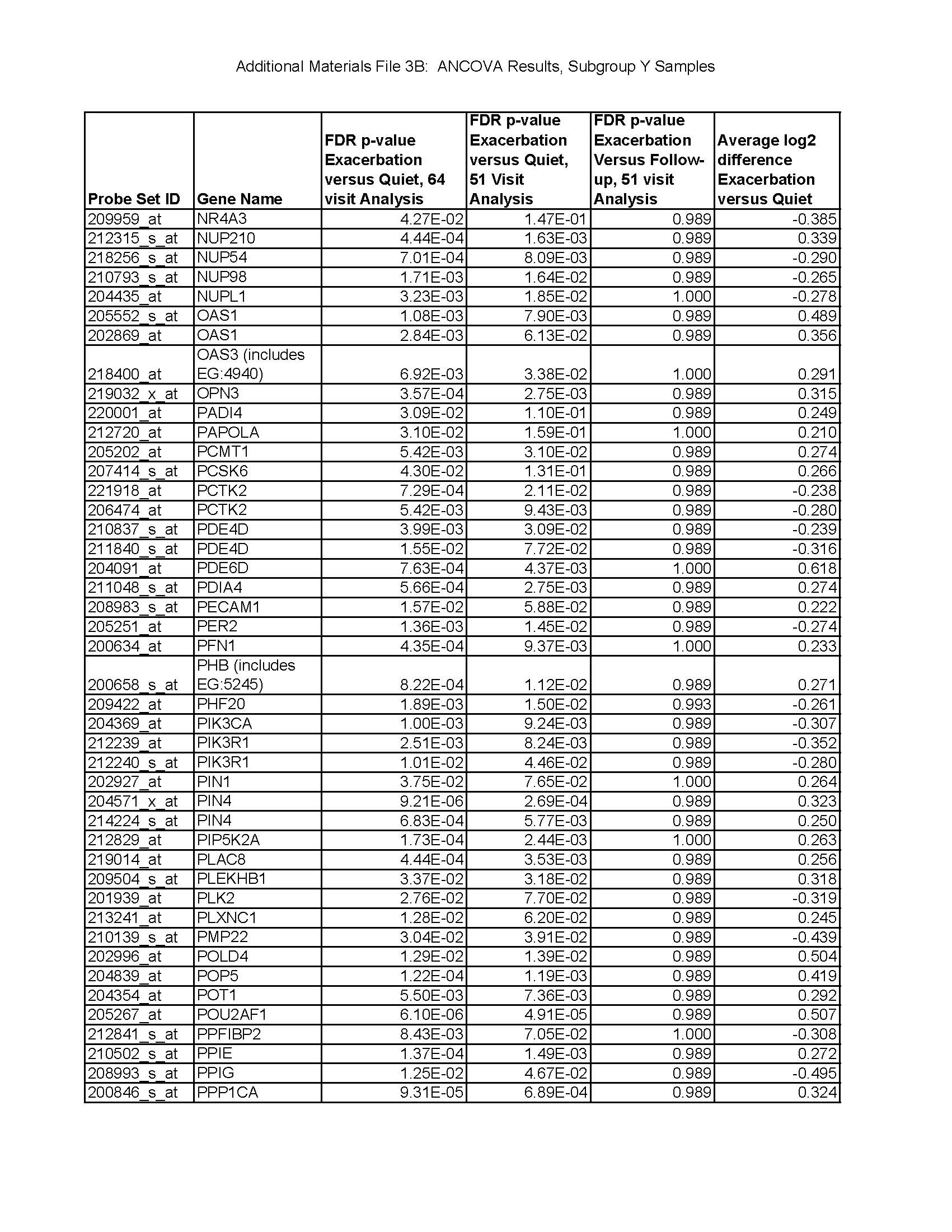


Table S18B: ANCOVA Results Subgroup Y continued
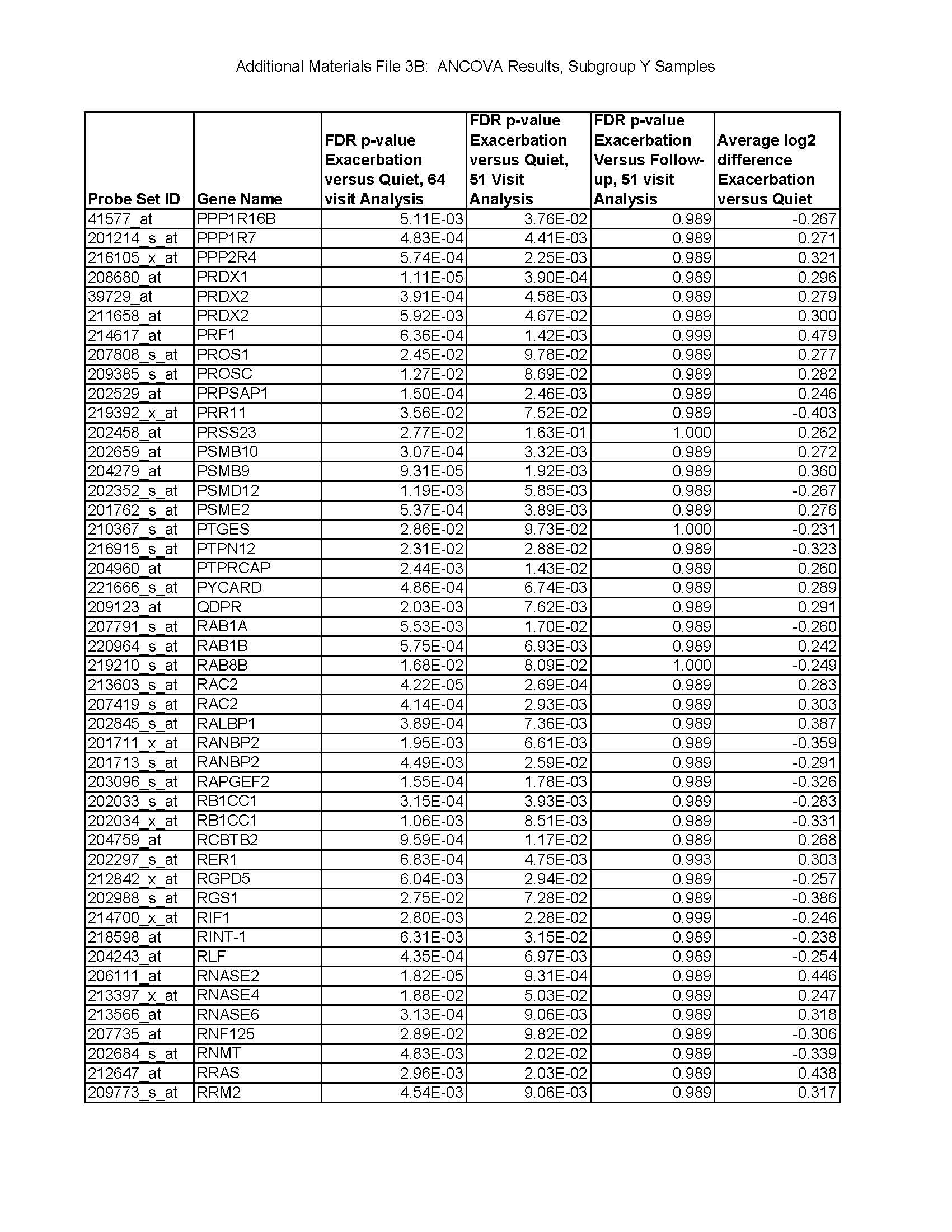


Table S18B: ANCOVA Results Subgroup Y continued
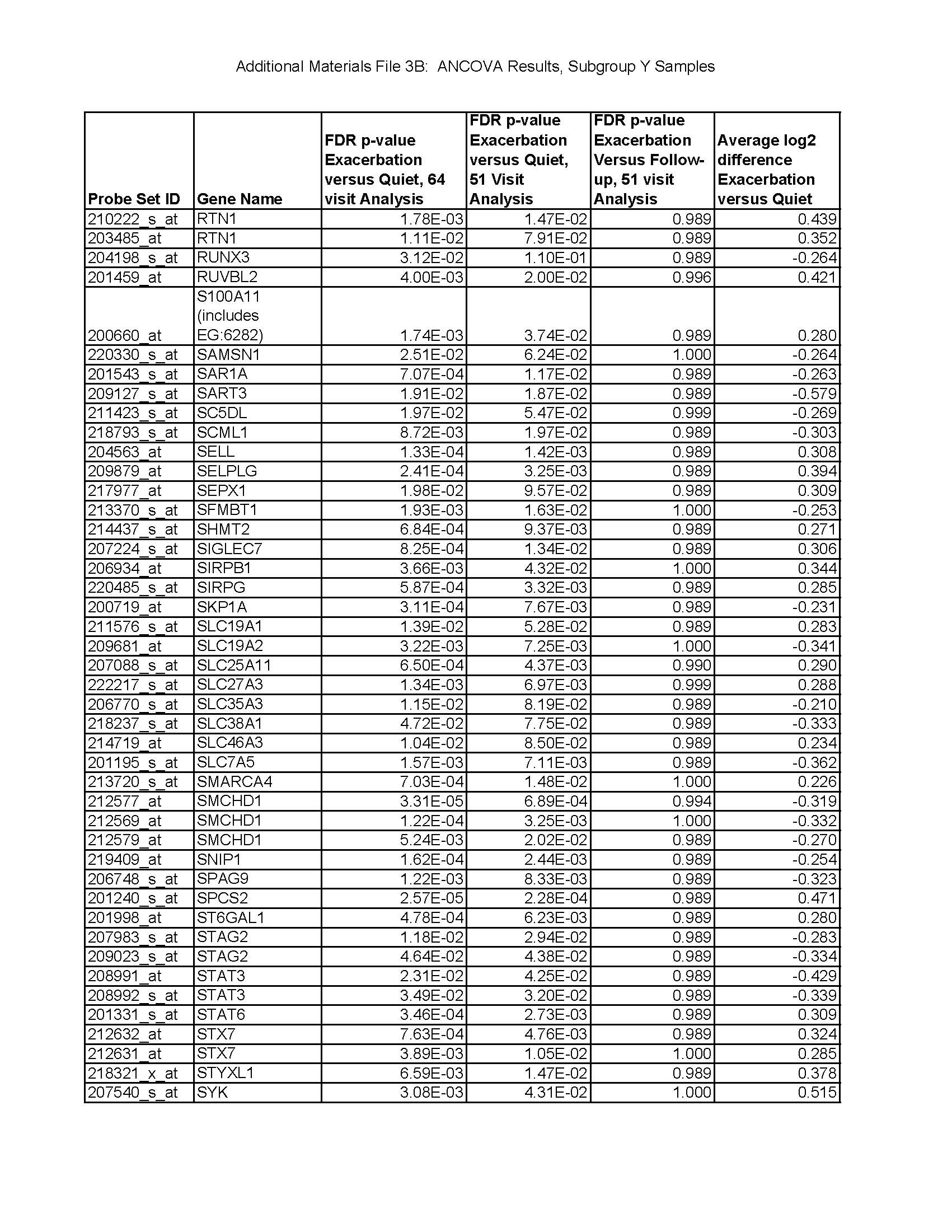
 Table S18B: ANCOVA Results Subgroup Y continued
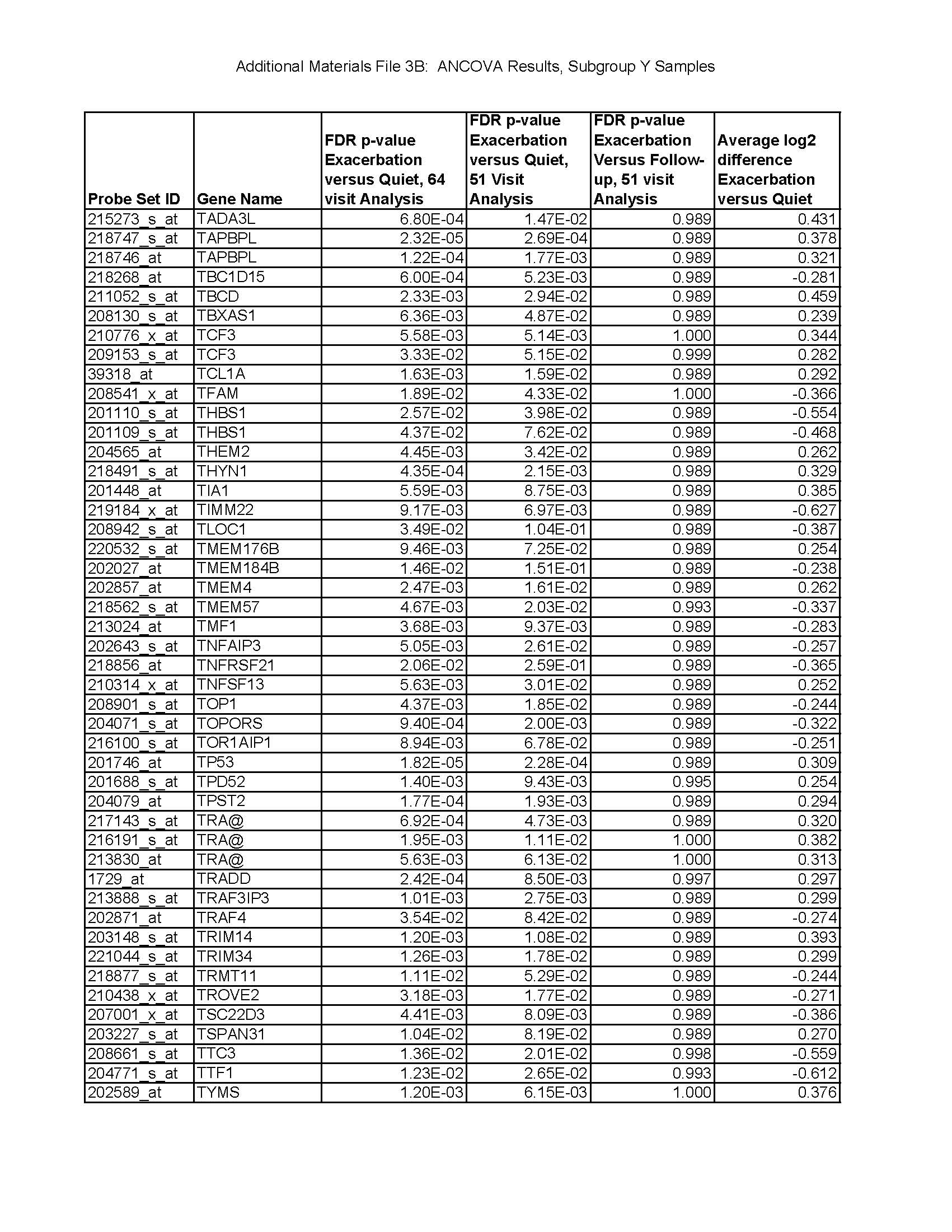


Table S18B: ANCOVA Results Subgroup Y continued


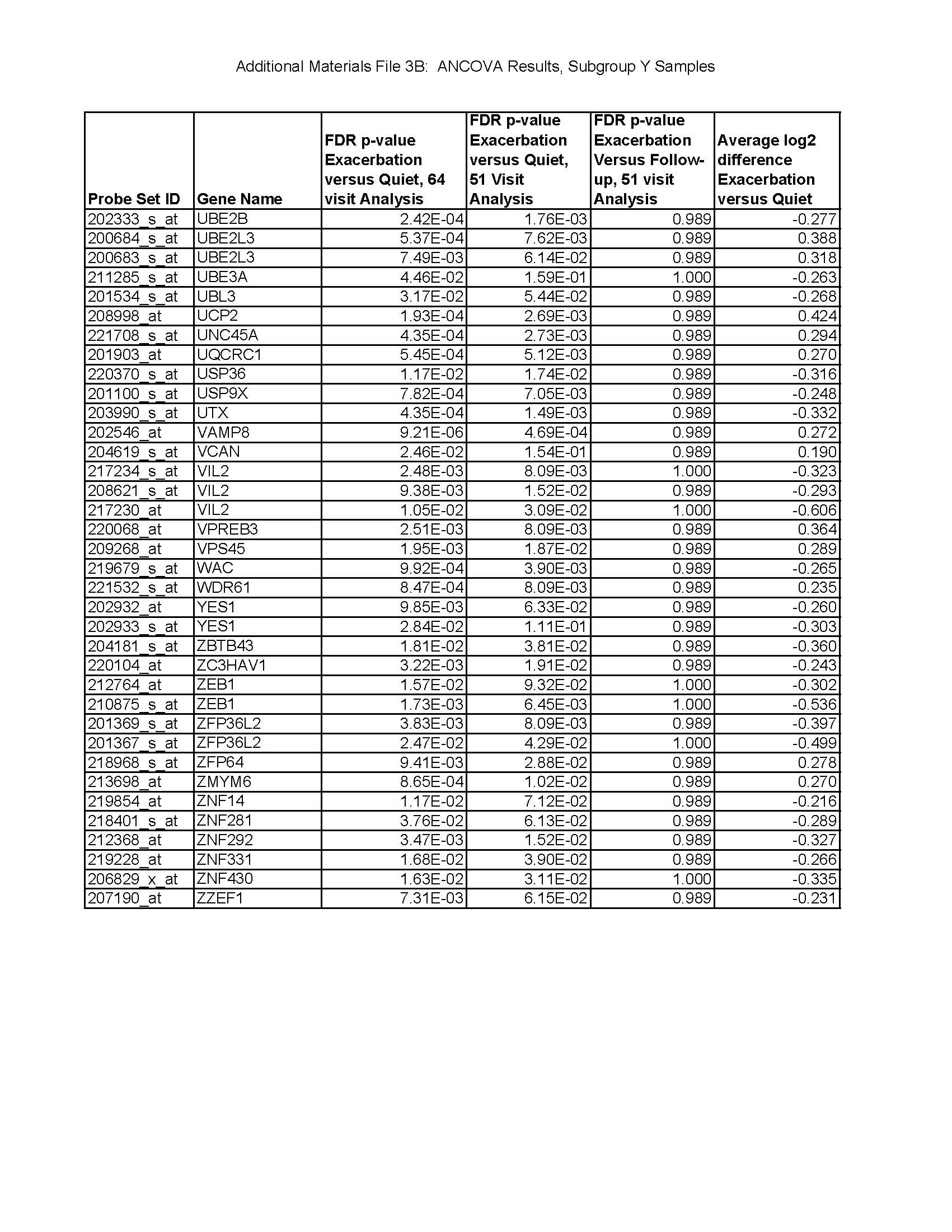


## Table S18C: ANCOVA Results Subgroup Z Samples


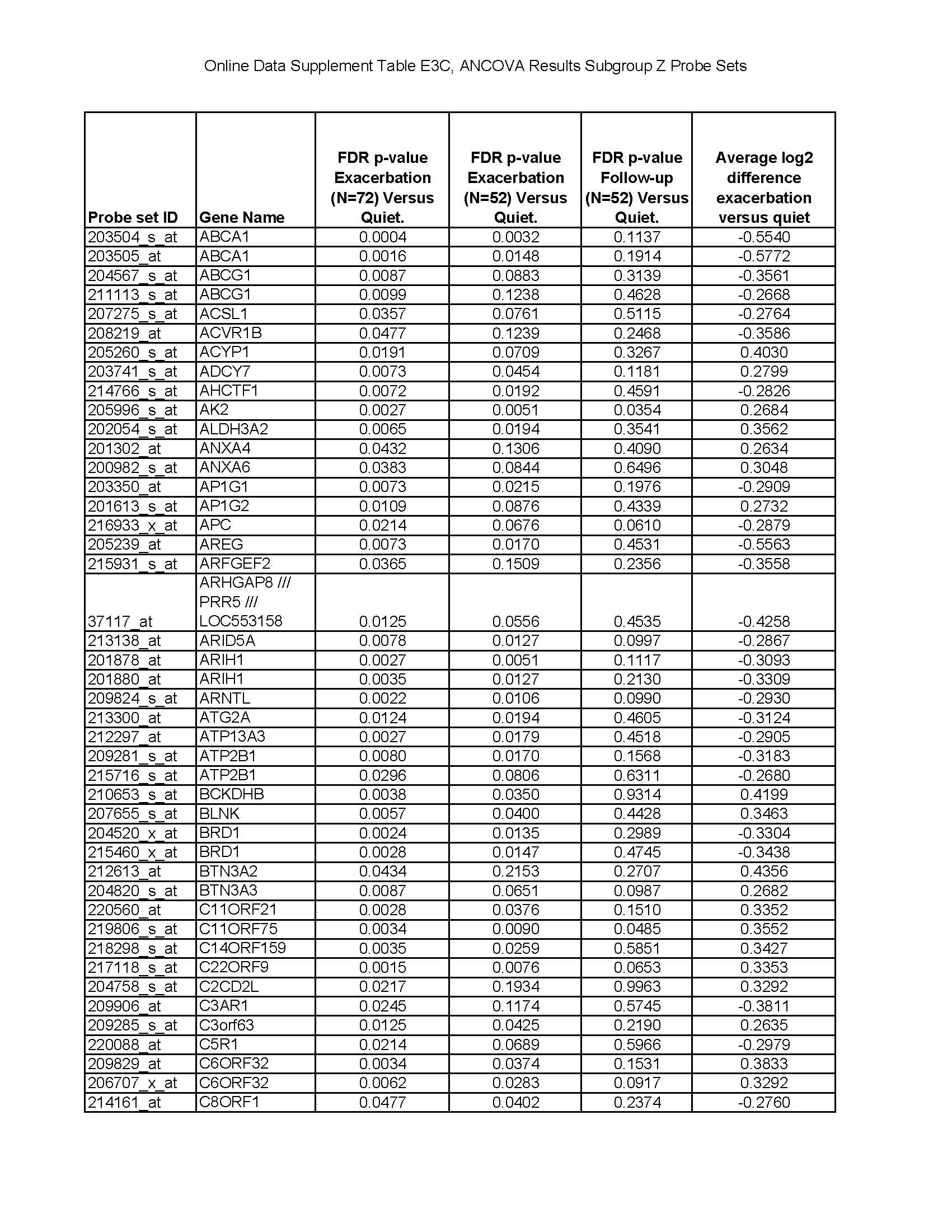


Table S18C: ANCOVA Results Subgroup Z continued
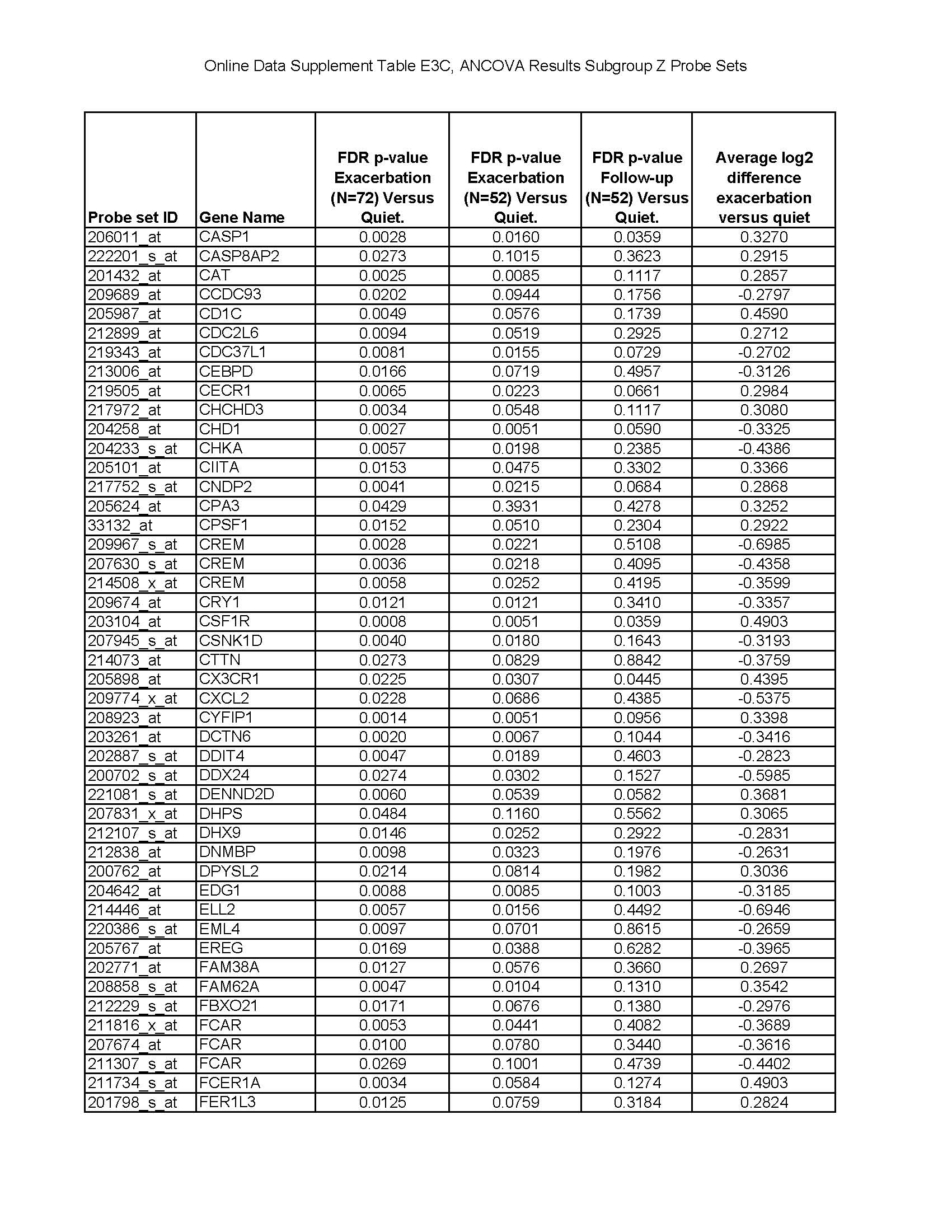


Table S18C: ANCOVA Results Subgroup Z continued
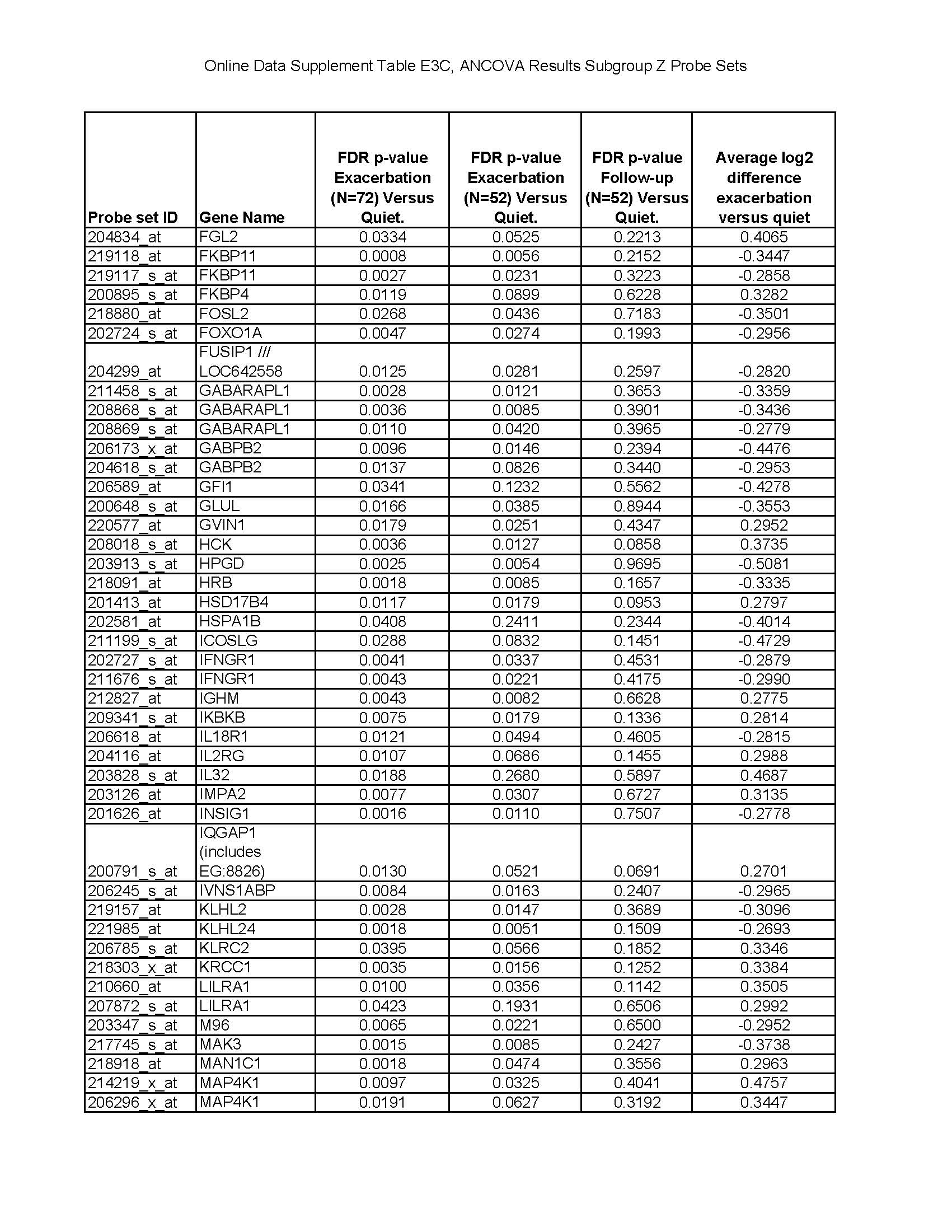


Table S18C: ANCOVA Results Subgroup Z continued
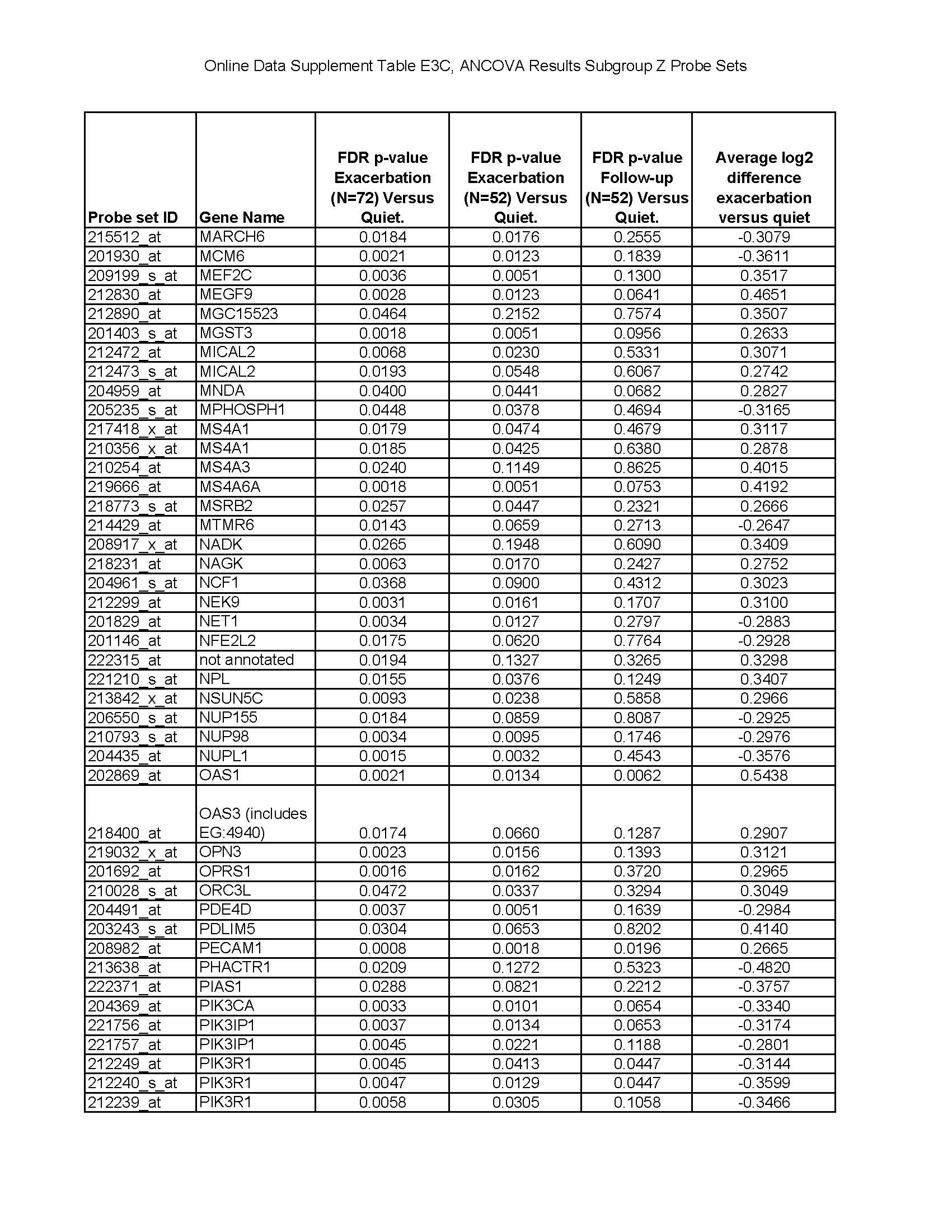


Table S18C: ANCOVA Results Subgroup Z continued
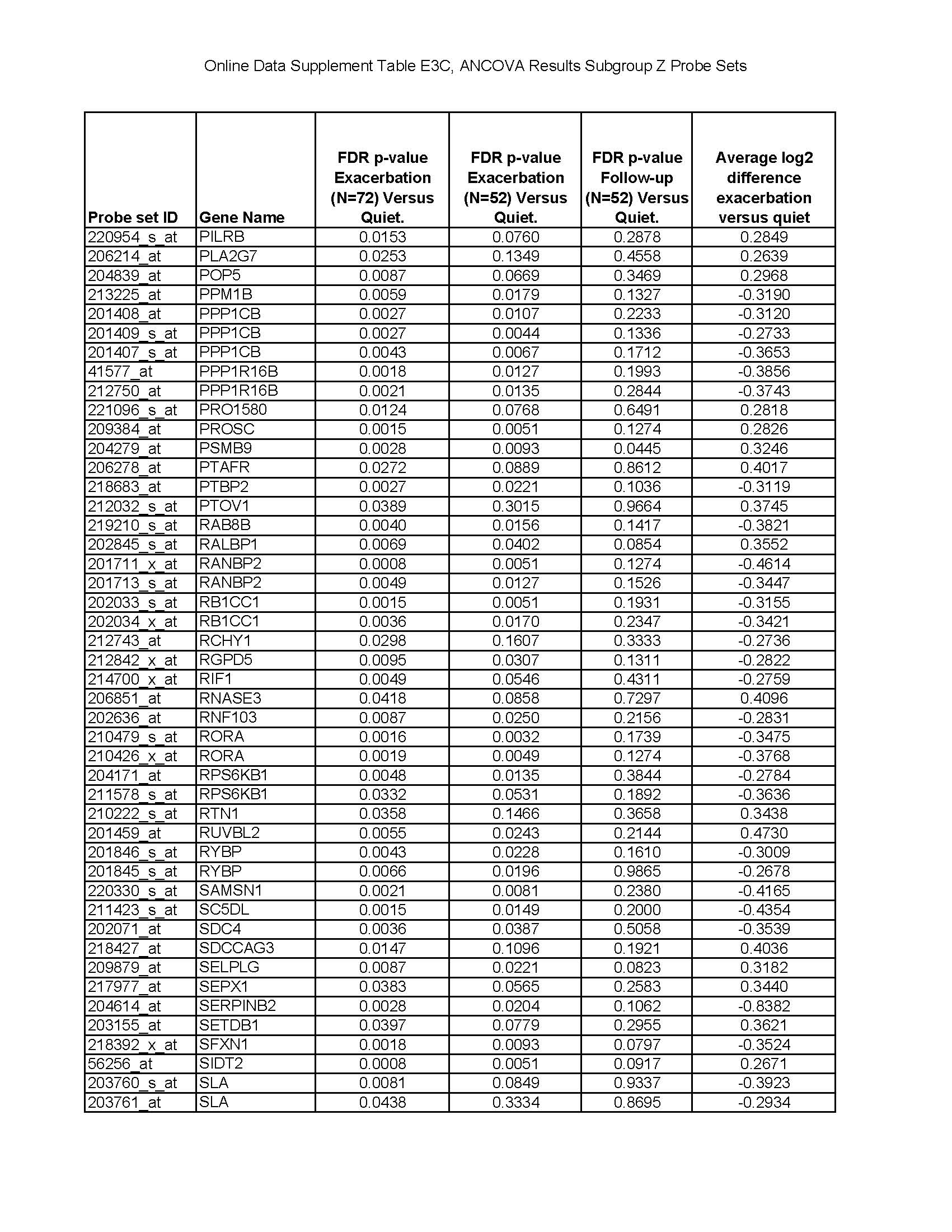


Table S18C: ANCOVA Results Subgroup Z continued
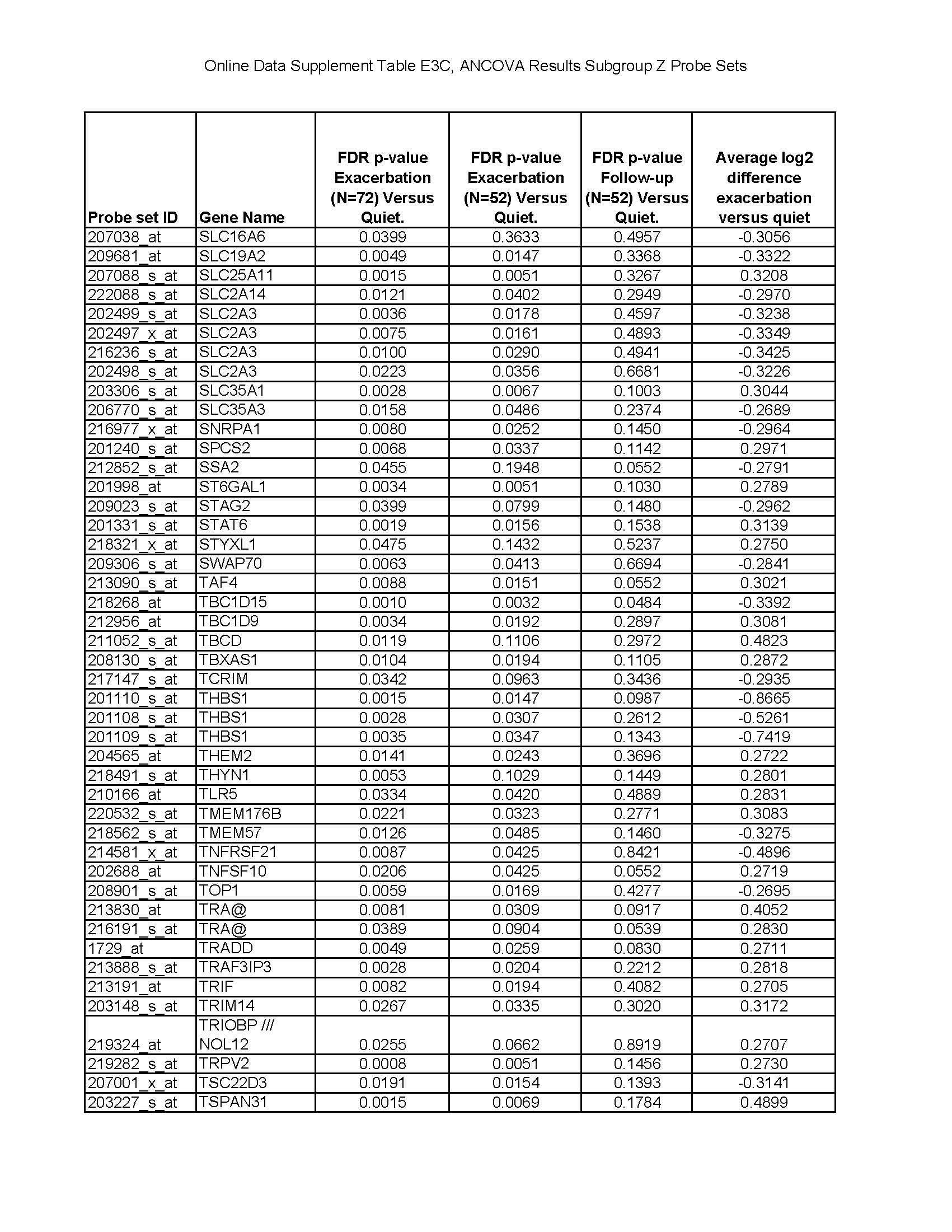


Table S18C: ANCOVA Results Subgroup Z continued


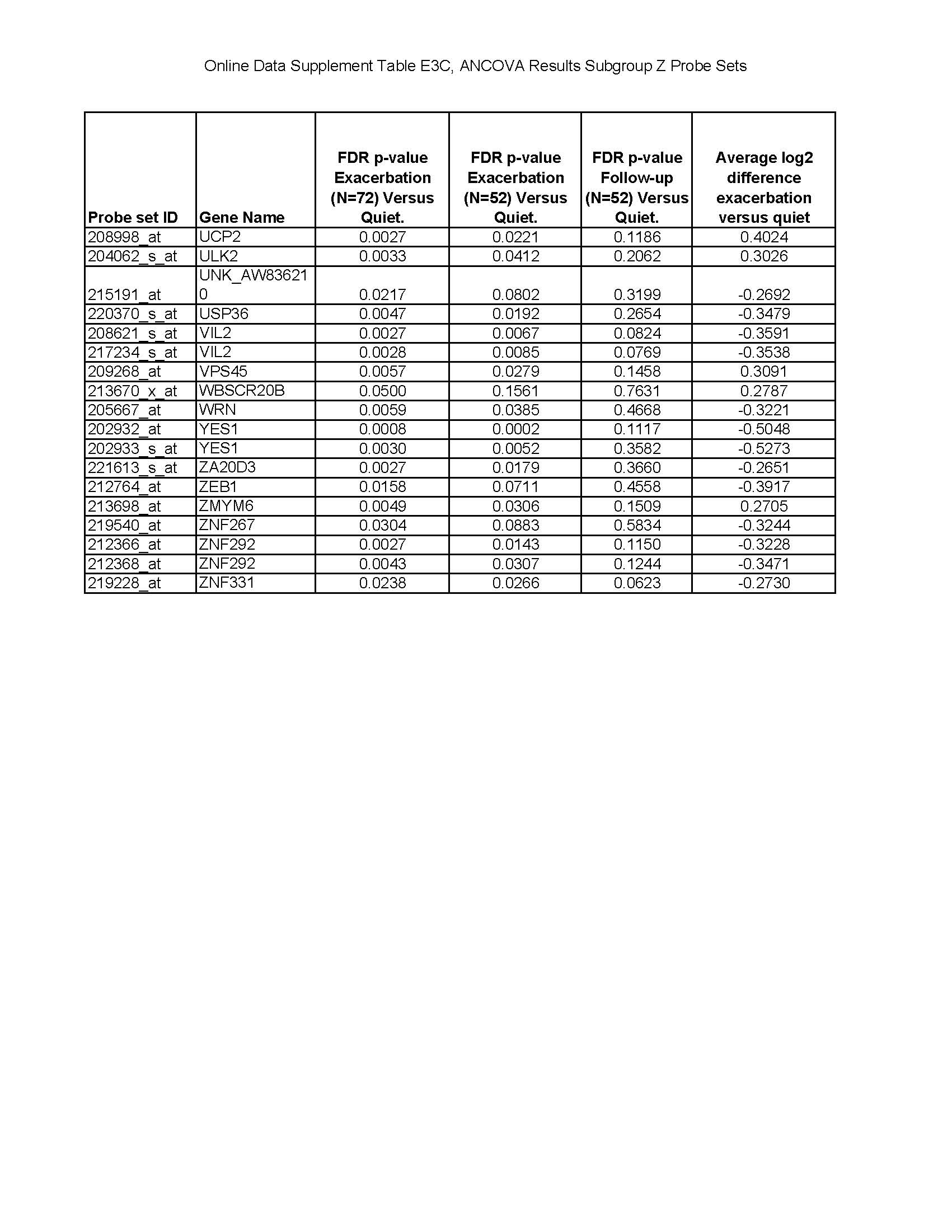


# Table S19: IL15 Pathway Genes Associated with Exacerbation in Subgroup X


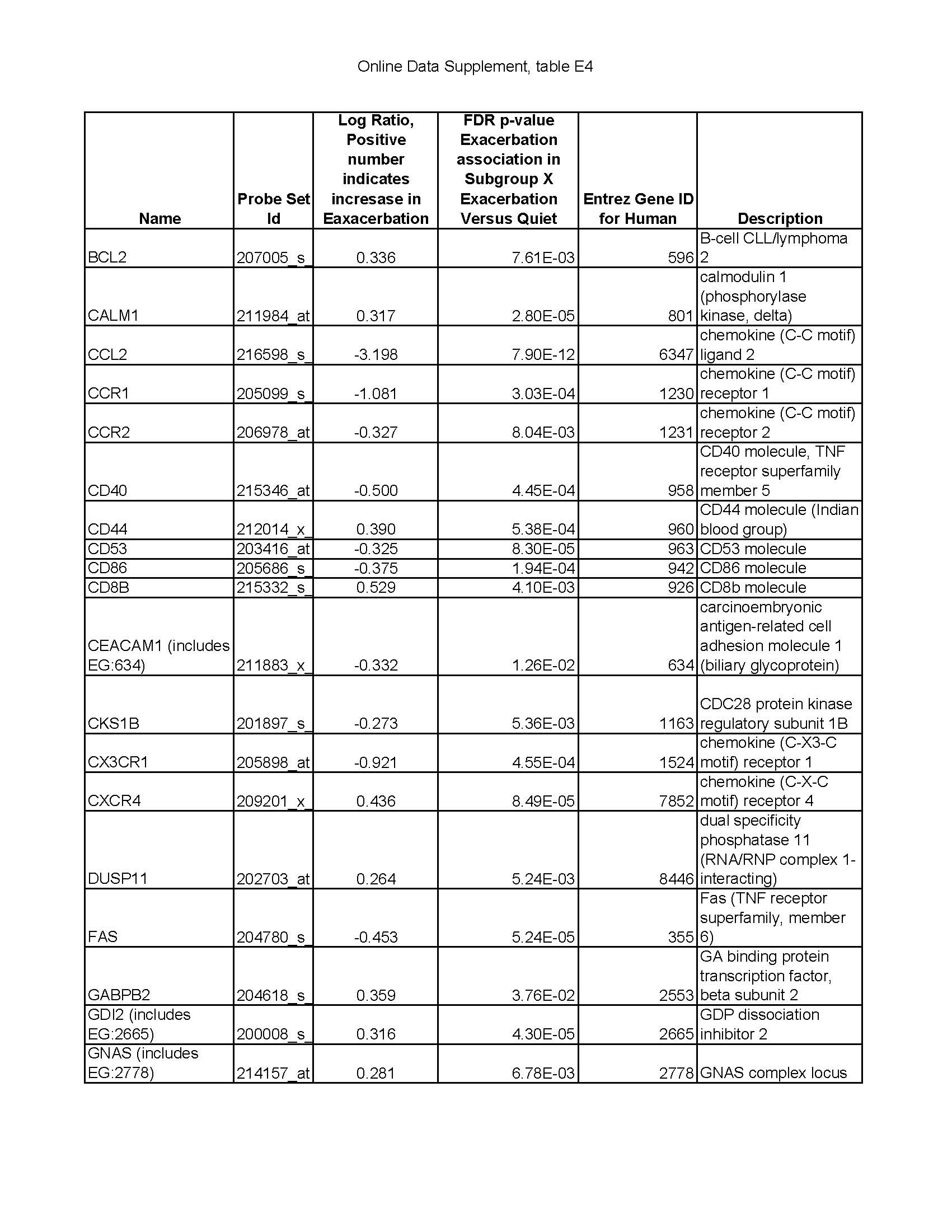


Table S19: IL15 Pathway genes Associated with Exacerbation in Subgroup X continued


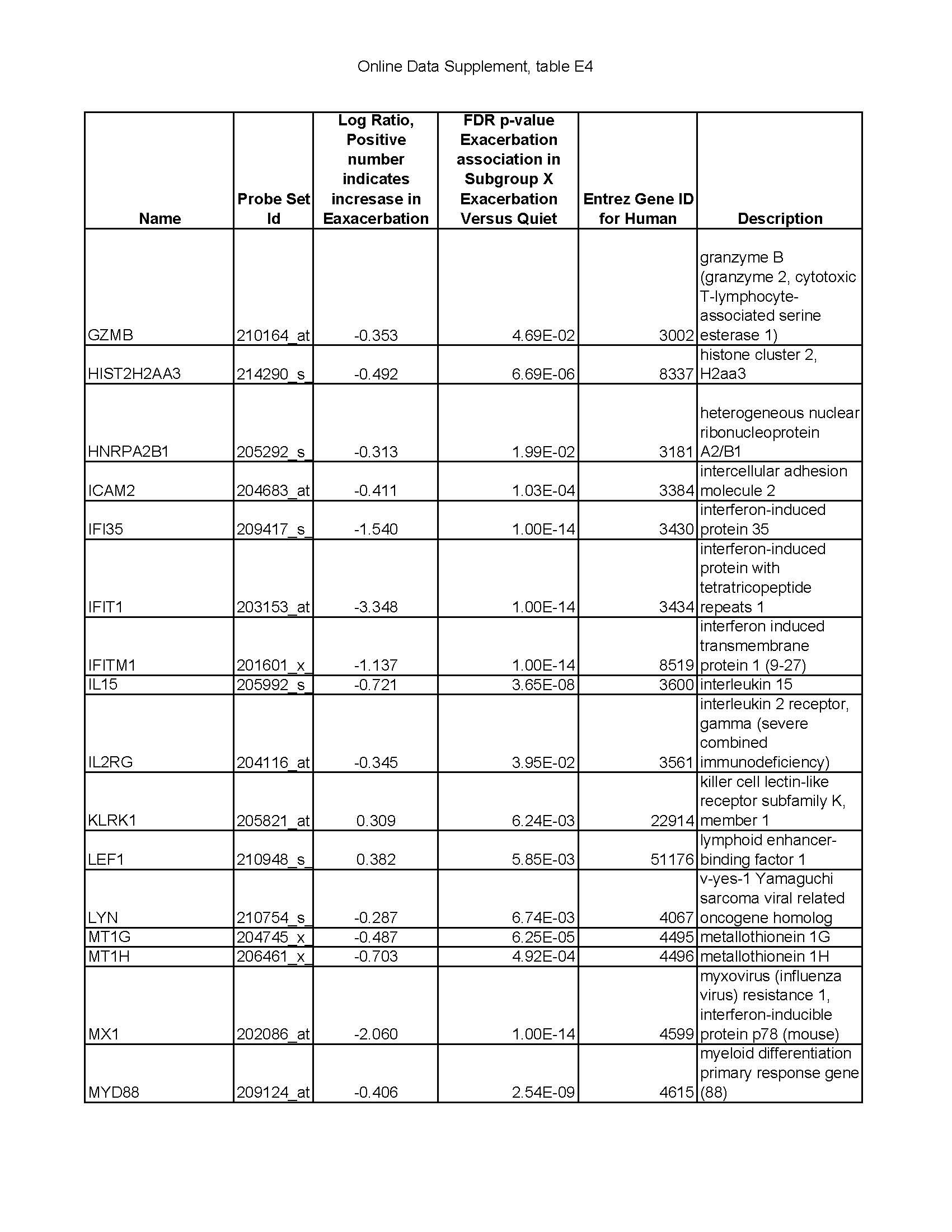


Table S19: IL15 Pathway genes Associated with Exacerbation in Subgroup X continued


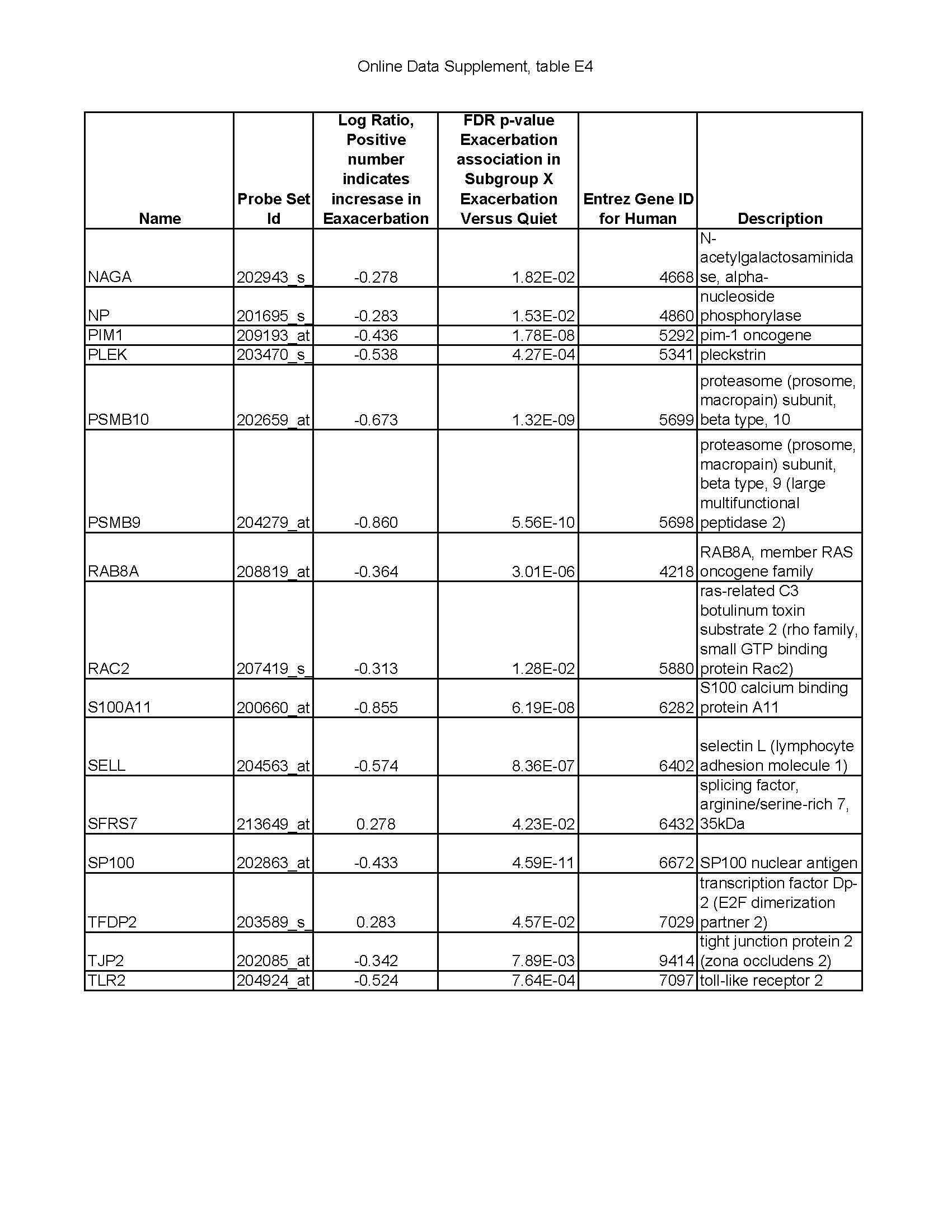


# Associations Between Covariates and Subgroup Assignments

Following definition of *exacerbation* subgroups based on k-means clustering of expression data, analyses were performed to evaluate the degree of association between subgroup assignments and selected demographic and clinical parameters.

## Association between Exacerbation Onset and Days to Exacerbation Sample Collection

The time between exacerbation onset and collection of the *exacerbation* sample varied from 0 to 14 days, but the subgroups did not differ with respect to this parameter. This is shown Figure S7.

## Figure S7. Subgroup Assignment and Days Between Exacerbation Onset and Exacerbation Sample Collection


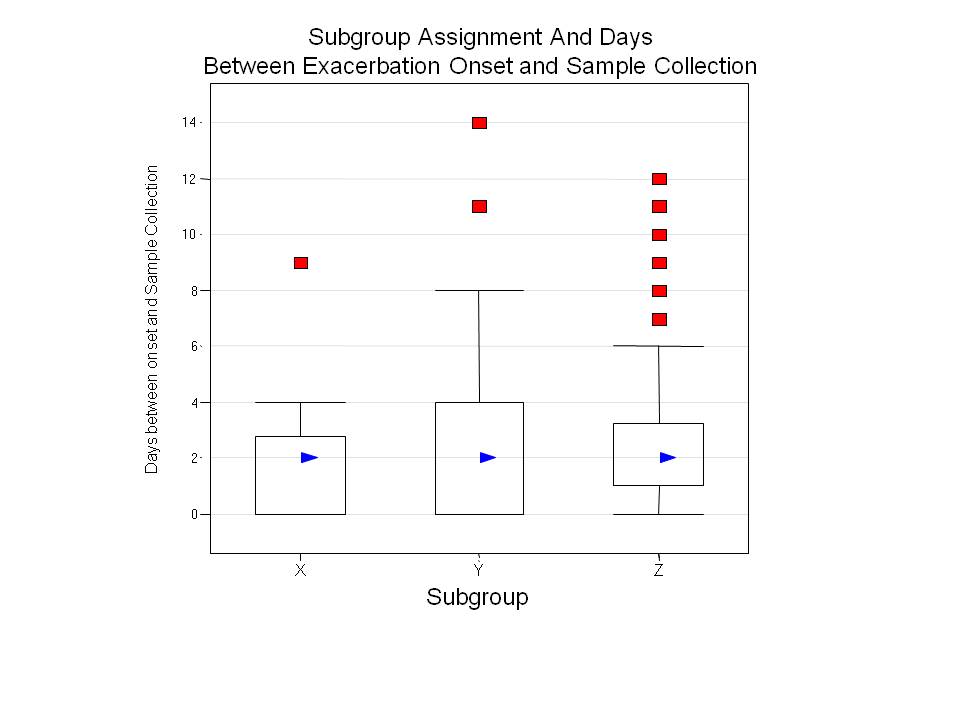


Results of analysis showing lack of association between days between exacerbation onset and collection of *exacerbation* sample.

For categorical demographic and clinical variables, chi-square tests for association were run. Fisher’s exact tests were used if expected counts were small (less than 5) for several cells in a cross-tabulation table. For continuous variables, one-way ANOVAs and associated pair-wise comparisons were done to compare means among the three subgroups. No adjustments for multiple testing were done.

## Association with Respiratory Function Measurements

The following tables summarize relationships between clinical measures of respiratory function during exacerbation visits and the cluster assignments defined based on k-means clustering, with k = 3, of donor-level ratios of expression during exacerbation and quiet visits. Separate analyses were done for each of four measures of respiratory function, and for each of the four corresponding measures of change from baseline in the respiratory function.

The clusters were based on clustering of ratios for 1079 probe sets for 166 exacerbation visits, resulting in 3 clusters designated as 1, 2, and 3, respectively. The 166 exacerbation visits were from 118 donors that had at least one quiet visit and at least one exacerbation visit, and one or more chips that passed QC filters for each type of visit. Note that due to missing values for some covariates, not all 166 visits were included in the analyses of covariance done in conjunction with the clustering effort.

For each measure of respiratory function or change from baseline in respiratory function, a table summarizing the findings is presented below. The table presents select summary statistics for each cluster, and p-values associated with comparisons among the clusters. The p-values are based on a repeated-measures analysis of variance with cluster as a fixed effect. The baseline values of the respiratory function measure were included as a covariate in the analyses of the respiratory function measures (but not in the analyses of change from baseline). Correlations between values within donors were addressed by including a compound symmetry variance structure in the model.

### Table S20: Subgroup Association with FEV1 (predicted)

|  | **Subgroup based on K-means clustering (k=3) of 1079 probesets** | | |
| --- | --- | --- | --- |
| **Statistic** | **Subgroup X** | **SubgroupY** | **Subgroup Z** |
| N | 25 | 58 | 61 |
| Mean | 79.9 | 76.2 | 76.5 |
| Median | 82 | 82 | 77 |
| S.D. | 24.7 | 21.6 | 21.1 |
| CV | 30.9 | 28.3 | 27.6 |
| Missing values | 5 | 6 | 11 |

p-value from overall F-test = 0.67. Because the F-test p-value was not statistically significant at the 0.05 level, no pairwise comparisons between cluster means were performed.

Conclusion: No statistically significant differences among Subgroups in FEV1 (predicted) during exacerbation visits.

### Table S21: Subgroup Association with FEV1 (predicted) change from baseline

|  | **Subgroup based on K-means clustering (k=3) of 1079 probesets** | | |
| --- | --- | --- | --- |
| **Statistic** | **Subgroup X** | **Subgroup Y** | **Subgroup Z** |
| N | 25 | 58 | 61 |
| Mean | -1.66 | 1.96 | 3.47 |
| Median | -0.1 | 1.2 | 2.0 |
| S.D. | 19.1 | 20.9 | 17.2 |
| Missing values | 5 | 6 | 11 |

p-value from overall F-test = 0.33. Because the F-test p-value was not statistically significant at the 0.05 level, no pairwise comparisons between Subgroup means were performed.

Conclusion: No statistically significant differences among subgroups in FEV1 (predicted) change from baseline during exacerbation visits.

### Table S22: Subgroup Association with FVC (predicted)

|  | **Subgroup based on K-means clustering (k=3) of 1079 probesets** | | |
| --- | --- | --- | --- |
| **Statistic** | **Subgroup X** | **Subgroup Y** | **Subgroup Z** |
| N | 25 | 57 | 61 |
| Mean | 91.6 | 88.7 | 83.9 |
| Median | 94 | 92 | 80 |
| S.D. | 22.3 | 19.3 | 18.8 |
| CV | 24.4 | 21.8 | 22.4 |
| Missing values | 5 | 7 | 11 |

p-value from overall F-test = 0.53. Because the F-test p-value was not statistically significant at the 0.05 level, no pairwise comparisons between Subgroup means were performed.

Conclusion: No statistically significant differences among Subgroups in FVC (predicted) during exacerbation visits.

### Table S23: Subgroup Association with FVC (predicted) change from baseline

|  | **Subgroup based on K-means clustering (k=3) of 1079 probesets** | | |
| --- | --- | --- | --- |
| **Statistic** | **Subgroup X** | **Subgroup Y** | **Subgroup Z** |
| N | 25 | 56 | 60 |
| Mean | -8.7 | -4.7 | -4.2 |
| Median | -6.0 | -5.5 | -2.5 |
| S.D. | 11.4 | 17.3 | 13.7 |
| Missing values | 5 | 8 | 12 |

p-value from overall F-test = 0.28. Because the F-test p-value was not statistically significant at the 0.05 level, no pairwise comparisons between Subgroup means were performed.

Conclusion: No statistically significant differences among Subgroups in FVC (predicted) change from baseline during exacerbation visits.

### Table S24: Subgroup Association with FEF 25-75% (predicted)

|  | **Subgroup based on K-means clustering (k=3) of 1079 probesets** | | |
| --- | --- | --- | --- |
| **Statistic** | **Subgroup X** | **Subgroup Y** | **Subgroup Z** |
| N | 24 | 57 | 61 |
| Mean | 55.6 | 53.3 | 60.0 |
| Median | 52 | 53 | 53 |
| S.D. | 25.1 | 28.7 | 30.4 |
| CV | 45.1 | 53.8 | 50.6 |
| Missing values | 6 | 7 | 11 |

p-value from overall F-test = 0.20. Because the F-test p-value was not statistically significant at the 0.05 level, no pairwise comparisons between Subgroup means were performed.

Conclusion: No statistically significant differences among Subgroups in FEF 25-75% (predicted) during exacerbation visits.

### Table S25: Subgroup Association with FEF 25-75% (predicted) change from baseline

|  | **Subgroup based on K-means clustering (k=3) of 1079 probesets** | | |
| --- | --- | --- | --- |
| **Statistic** | **Subgroup X** | **Subgroup Y** | **Subgroup Z** |
| N | 23 | 55 | 57 |
| Mean | -9.8 | -2.9 | -0.9 |
| Median | -5.0 | -2.0 | -3.8 |
| S.D. | 25.8 | 14.4 | 19.0 |
| Missing values | 7 | 9 | 15 |

p-value from overall F-test = 0.12. Because the F-test p-value was not statistically significant at the 0.05 level, no pairwise comparisons between Subgroup means were performed.

Conclusion: No statistically significant differences among Subgroups in FEF 25-75% (predicted) change from baseline during exacerbation visits.

### Table S26: Subgroup Association with PEF (predicted)

|  | **Subgroup based on K-means clustering (k=3) of 1079 probesets** | | |
| --- | --- | --- | --- |
| **Statistic** | **Subgroup X** | **Subgroup Y** | **Subgroup Z** |
| N | 23 | 53 | 60 |
| Mean | 80.2 | 80.2 | 81.8 |
| Median | 95 | 93 | 86 |
| S.D. | 33.5 | 26.6 | 29.4 |
| CV | 41.8 | 33.1 | 36.0 |
| Missing values | 7 | 11 | 12 |

p-value from overall F-test = 0.66. Because the F-test p-value was not statistically significant at the 0.05 level, no pairwise comparisons between Subgroup means were performed.

Conclusion: No statistically significant differences among Subgroups in PEF (predicted) during exacerbation visits.

### Table S27: Subgroup Association with PEF (predicted) change from baseline

|  | **Subgroup based on K-means clustering (k=3) of 1079 probesets** | | |
| --- | --- | --- | --- |
| **Statistic** | **Subgroup X** | **Subgroup Y** | **Subgroup Z** |
| N | 23 | 50 | 57 |
| Mean | -7.2 | -4.6 | -4.2 |
| Median | -5.3 | -6.0 | -3.7 |
| S.D. | 19.4 | 15.1 | 15.8 |
| Missing values | 7 | 14 | 15 |

p-value from overall F-test = 0.62. Because the F-test p-value was not statistically significant at the 0.05 level, no pairwise comparisons between Subgroup means were performed.

Conclusion: No statistically significant differences among Subgroups in PEF (predicted) change from baseline during exacerbation visits.

## Table S28: Subgroup Association with Relevant Respiratory Infection

(visit-level variable, using respiratory infection as defined by Cristina Csimma)

|  | Subgroup based on K-means clustering (k=3) of 1079 probesets | | |  |
| --- | --- | --- | --- | --- |
| Any relevant infections | Subgroup X | Subgroup Y | Subgroup Z | Total |
| No | 10 (33.3%) | 28 (43.8%) | 39 (54.2%) | 77 |
| Yes | 20 (66.7%) | 36 (56.3%) | 33 (45.8%) | 89 |
| Total | 30 | 64 | 72 | 166 |

p-value = 0.14

Conclusion: No evidence of association between relevant infections and Subgroup assignments.

## Table S29: Subgroup Association with Disease Severity

(donor-level variable)

|  | Subgroup based on K-means clustering (k=3) of 1079 probesets | | |  |
| --- | --- | --- | --- | --- |
| Severity | Subgroup X | Subgroup Y | Subgroup Z | Total |
| Mild | 2 (6.7%) | 4 (6.3%) | 1 (1.4%) | 7 |
| Moderate | 14 (46.7%) | 23 (35.9%) | 31 (43.1%) | 68 |
| Severe | 14 (46.7%) | 37 (57.8%) | 40 (55.6%) | 91 |
| Total | 30 | 64 | 72 | 166 |

p-value = 0.46 (note: would be better with exact test p-values)

Conclusion: No evidence of association between severity and Subgroup assignments.

## Association with Use of Medication

For the analyses of non-study medication use related to acid reflux, reported drug descriptions in the clinical database were mapped to drug types as follows:

PPIs – PANTOPRAZOLE, PANTOPRAZOL SODIUM, NEXIUM, PRILOSEC, OMEPRAZOLE, OMEPRAZOLE MAGNESIUM, LANSOPRAZOLE, RABEPRAZOLE, RABEBRAZOLE SODIUM, LOSEC, PROTIUM

Histamine H2 Antagonists – PEPCID, PEPCIDIN, FAMOTIDINE, ZANTAC, RANITIDINE

Reported but not assigned to a drug type – ALTACITE, CAL_SUP, CALCICHEW, CARAFATE, GAVISCON, MAALOX, MAGNESIUM OXIDE, OSCAL, ROLAIDS, SIMETHICONE, TUMS

One other covariate associated with acid reflux was examined – gastroesophageal reflux disease reported as an adverse event during the study. No such adverse events were reported during the 166 visits included in these analyses.

### Table S30: Subgroup Association with Use of Medication: Systemic Corticosteroids

(visit-level variable, using non-study medication classification of Charlotte McKee)

|  | Subgroup based on K-means clustering (k=3) of 1079 probesets | | |  |
| --- | --- | --- | --- | --- |
| Any systemic steroid use | Subgroup X | Subgroup Y | Subgroup Z | Total |
| No | 19 (63.3%) | 29 (45.3%) | 34 (47.2%) | 82 |
| Yes | 11 (36.7%) | 35 (54.7%) | 38 (52.8%) | 84 |
| Total | 30 | 64 | 72 | 166 |

p-value = 0.24

Conclusion: No evidence of association between systemic corticosteroid use and Subgroup assignments.

### Table S31: Subgroup Association with Use of Medication: Inhaled Corticosteroids

(visit-level variable, using non-study medication classification of Charlotte McKee)

|  | Subgroup based on K-means clustering (k=3) of 1079 probesets | | |  |
| --- | --- | --- | --- | --- |
| Any inhaled steroid use | Subgroup X | Subgroup Y | Subgroup Z | Total |
| No | 1 (3.3) | 5 (7.8%) | 2 (2.8%) | 8 |
| Yes | 29 (96.7%) | 59 (92.2%) | 70 (97.2%) | 158 |
| Total | 30 | 64 | 72 | 166 |

p-value = 0.36 (would be better with exact test p-value)

Conclusion: No evidence of association between inhaled corticosteroid use and Subgroup assignments.

### Table S32: Subgroup Association with Use of Medication: Association with Use of Intranasal Corticosteroids

(visit-level variable, using non-study medication classification of Charlotte McKee, Medical Monitor)

|  | Subgroup based on K-means clustering (k=3) of 1079 probesets | | |  |
| --- | --- | --- | --- | --- |
| Any intranasal steroid use | Subgroup X | Subgroup Y | Subgroup Z | Total |
| No | 20 (66.7%) | 32 (50.0%) | 39 (54.2%) | 91 |
| Yes | 10 (33.3%) | 32 (50.0%) | 33 (45.8%) | 75 |
| Total | 30 | 64 | 72 | 166 |

p-value = 0.31

Conclusion: No evidence of association between intranasal corticosteroid use and node assignments.

### Table S33: Subgroup Association with Use of Medication: Leukotriene Antagonists

(visit-level variable, using non-study medication classification of Charlotte McKee)

|  | Subgroup based on K-means clustering (k=3) of 1079 probesets | | |  |
| --- | --- | --- | --- | --- |
| Any leukotriene antagonist use | Subgroup X | Subgroup Y | Subgroup Z | Total |
| No | 19 (63.3%) | 48 (75.0%) | 48 (66.7%) | 115 |
| Yes | 11 (36.7%) | 16 (25.0%) | 24 (33.3%) | 51 |
| Total | 30 | 64 | 72 | 166 |

p-value = 0.43

Conclusion: No evidence of association between leukotriene antagonist use and the Subgroup assignments.

### Table S34: Subgroup Association with Use of Medication: Any GI Non-Study Med Use

(visit-level analysis)

|  | Subgroup based on K-means Subgrouping (k=3) of 1079 probesets | | |  |
| --- | --- | --- | --- | --- |
| Any GI non-study med use? | Subgroup X | Subgroup Y | Subgroup Z | Total |
| No | 22 (73.3%) | 43 (67.2%) | 51 (70.8%) | 116 |
| Yes | 8 (26.7%) | 21 (32.8%) | 21 (29.2%) | 50 |
| Total | 30 | 64 | 72 | 166 |

p-value = 0.81

Conclusion: No evidence of association between GI non-study medication use and Subgroup assignments.

### Table S35: Subgroup Association with Use of Medication: Any PPI Non-study Med Use

(visit-level analysis)

|  | Subgroup based on K-means clustering (k=3) of 1079 probesets | | |  |
| --- | --- | --- | --- | --- |
| Any PPI non-study med use? | Subgroup X | Subgroup Y | Subgroup Z | Total |
| No | 22 (73.3%) | 44 (68.8%) | 52 (72.2%) | 118 |
| Yes | 8 (26.7%) | 20 (31.3%) | 20 (27.8%) | 48 |
| Total | 30 | 64 | 72 | 166 |

p-value = 0.87

Conclusion: No evidence of association between PPI non-study medication use and Subgroup assignments.

### Table S36: Subgroup Association with Use of Medication: Association with Any Histamine H2 Antagonist Non-study Med Use

(visit-level analysis)

|  | Subgroup based on K-means clustering (k=3) of 1079 probesets | | |  |
| --- | --- | --- | --- | --- |
| Any H2 antagonist non-study med use? | Subgroup X | Subgroup Y | Subgroup Z | Total |
| No | 30 (100%) | 63 (98.4%) | 72 (100%) | 165 |
| Yes | 0 (0%) | 1 (1.6%) | 0 (0%) | 1 |
| Total | 30 | 64 | 72 | 166 |

p-value = 0.45 (would be better with exact test p-values)

Conclusion: No evidence of association between H2 antagonist non-study medication use and Subgroup assignments.

## Table S37: Subgroup Association with Sex

(donor-level variable)

|  | Subgroup based on K-means clustering (k=3) of 1079 probesets | | |  |
| --- | --- | --- | --- | --- |
| Sex | Subgroup X | Subgroup Y | Subgroup Z | Total |
| F | 17 (56.7%) | 46 (71.9%) | 54 (75.0%) | 117 |
| M | 13 (43.3%) | 18 (28.1%) | 18 (25.0%) | 49 |
| Total | 30 | 64 | 72 | 166 |

p-value = 0.17

Conclusion: No evidence for association between sex and Subgroup assignments.

## Table S38: Subgroup Association with Race

(donor-level variable)

|  | Subgroup based on K-means clustering (k=3) of 1079 probesets | | |  |
| --- | --- | --- | --- | --- |
| Race | Subgroup X | Subgroup Y | Subgroup Z | Total |
| A | 1 (3.3%) | 1 (1.6%) | 3 (4.2%) | 5 |
| B | 5 (16.7%) | 3 (4.7%) | 8 (11.1%) | 16 |
| W | 24 (80.0%) | 60 (93.8%) | 61 (84.7%) | 145 |
| Total | 30 | 64 | 72 | 166 |

p-value = 0.33 (note: would be better with exact test p-values)

Conclusion: No evidence for association between race and Subgroup assignments.

## Table S39: Subgroup Association with Sample Processing Laboratory

(donor-level variable)

|  | Subgroup based on K-means clustering (k=3) of 1079 probesets | | |  |
| --- | --- | --- | --- | --- |
| CRO | Subgroup X | Subgroup Y | Subgroup Z | Total |
| AARI | 6 (20.0%) | 11 (17.2%) | 14 (19.4%) | 31 |
| DeCode | 7 (23.3%) | 18 (28.1%) | 18 (25.0%) | 43 |
| ICON-D | 6 (20.0%) | 14 (21.9%) | 20 (27.8%) | 40 |
| ICON-F | 11 (36.7%) | 21 (32.8%) | 20 (27.8%) | 52 |
| Total | 30 | 64 | 72 | 166 |

p-value = 0.95

Conclusion: No evidence for association between CRO and Subgroup assignments.

## Table S40: Subgroup Association with Country

(donor-level variable)

|  | Subgroup based on K-means clustering (k=3) of 1079 probesets | | |  |
| --- | --- | --- | --- | --- |
| Country | Subgroup X | Subgroup Y | Subgroup Z | Total |
| AUS | 6 (20.0%) | 11 (17.2%) | 14 (19.4%) | 31 |
| GBR | 1 (3.3%) | 7 (10.9%) | 11 (15.2%) | 19 |
| IRL | 5 (16.7%) | 7 (10.9%) | 9 (12.5%) | 21 |
| ISL | 7 (23.3%) | 18 (28.1%) | 18 (25.0%) | 43 |
| USA | 11 (36.7%) | 21 (32.8%) | 20 (27.8%) | 52 |
| Total | 30 | 64 | 72 | 166 |

p-value = 0.84

Conclusion: No evidence for association between country and Subgroup assignments.

## Table S41: Subgroup Association with Atopy Status

(donor-level variable)

|  | Subgroup based on K-means clustering (k=3) of 1079 probesets | | |  |
| --- | --- | --- | --- | --- |
| Atopy Status | Subgroup X | Subgroup Y | Subgroup Z | Total |
| Atopic | 19 (63.3%) | 41 (64.1%) | 54 (75.0%) | 114 |
| Non-atopic | 7 (23.3%) | 18 (28.1%) | 11 (15.3%) | 36 |
| Unknown | 4 (13.3%) | 5 (7.8%) | 7 (9.7%) | 16 |
| Total | 30 | 64 | 70 | 166 |

p-value = 0.40

Conclusion: No evidence of association between atopy status and Subgroup assignments.

## Table S42: Subgroup Association with Fasting Status

(visit-level variable)

|  | Subgroup based on K-means clustering (k=3) of 1079 probesets | | |  |
| --- | --- | --- | --- | --- |
| Fasting status | Subgroup X | Subgroup Y | Subgroup Z | Total |
| Unknown | 0 (0.0%) | 9 (14.6%) | 2 (2.8%) | 11 |
| Fasting/Yes | 9 (30.0%) | 14 (21.9%) | 21 (29.2%) | 44 |
| Non-fasting/No | 21 (70.0%) | 41 (64.1%) | 49 (68.1%) | 111 |
| Total | 30 | 64 | 72 | 166 |

p-value = 0.042 (would be better with exact test p-values)

Conclusion: Evidence of association between fasting status and Subgroup assignments.

## Table S43: Subgroup Association with IgE

|  | **Subgroup based on K-means clustering (k=3) of 1079 probesets** | | |
| --- | --- | --- | --- |
|  | **Exacerbation** | | |
| **Statistic** | **Subgroup X** | **Subgroup Y** | **Subgroup Z** |
| N | 10 | 21 | 24 |
| Mean | 317.1 | 348.1 | 113.7 |
| Median | 38.0 | 126.0 | 64.5 |
| S.D. | 541.7 | 572.5 | 174.4 |
| CV | 170.8 | 164.4 | 153.5 |
| 5^th^ percentile | 10 | 40 | 10 |
| 95^th^ percentile | 1500 | 992 | 326 |
| Missing values | 20 | 43 | 48 |

Conclusions:

1. Poorly behaved variable from a statistical point of view – very skewed, lots of variability.
2. Large amount of missing data makes if questionable how much we’ll get out of this.

## Table S44: Subgroup Association with Medical History of Acid Reflux

(donor-level variable)

|  | Subgroup based on K-means clustering (k=3) of 1079 probesets | | |  |
| --- | --- | --- | --- | --- |
| Any history? | Subgroup X | Subgroup Y | Subgroup Z | Total |
| No | 20 (66.7%) | 39 (60.9%) | 45 (62.5%) | 104 |
| Yes | 10 (33.3%) | 25 (39.1%) | 27 (37.5%) | 62 |
| Total | 30 | 64 | 72 | 166 |

p-value = 0.87

Conclusion: No evidence for association between medical history of acid reflux and Subgroup assignments.

## Table S45: Association with BMI (based on screening height and weight)

(donor-level variable)

For the days after quiet visits and BMI analyses, the p-value is from a one-way analysis of variance (ANOVA).

|  | **Cluster based on 1079 probeset clustering** | | |
| --- | --- | --- | --- |
| **Statistic** | **Cluster X** | **Cluster Y** | **Cluster Z** |
| N | 30 | 64 | 53 |
| Mean | 28.4 | 32.4 | 30.2 |
| Median | 28.0 | 32.5 | 29.3 |
| S.D. | 6.2 | 6.6 | 6.2 |
| CV | 22.0 | 20.5 | 20.6 |

p-value from test for differences of means among clusters = 0.015

Conclusion: Some evidence of differences among clusters in mean BMI. Mean BMI is statistically significantly lower (p=0.006) in cluster 1 than cluster 2, and is statistically suggestively lower (p=0.0501) in cluster 3 than cluster 2.

## Table S46: Subgroup Association with Days Since Quiet Visit

|  | **Subgroup based on 1079 probeset clustering** | | |
| --- | --- | --- | --- |
| **Statistic** | **Subgroup X** | **Subgroup Y** | **Subgroup Z** |
| N | 30 | 64 | 71 |
| Mean | 48.4 | 62.7 | 79.6 |
| Median | 40.5 | 40 | 69 |
| S.D. | 45.3 | 55.3 | 64.0 |
| CV | 93.6 | 88.3 | 80.5 |
| 5^th^ percentile | 9 | 7 | 11 |
| 95^th^ percentile | 91 | 181 | 211 |
| Missing values | 0 | 0 | 1 |

p-value from test for differences of means among Subgroups = 0.03

Conclusions:

1. Large variability, some skewing, not many missing observations. May be a variable with some statistical utility.
2. Interesting differences between nodes in mean number of days since quiet visit. Exacerbation visits occur sooner after a quiet in some nodes than in others. Statistically significant difference between Subgroups 1 and 3 (p=0.014), difference between Subgroups 2 and 3 is statistically suggestive (p=0.091).

##

When subjects with or without exacerbations were compared within an asthma severity category, the difference between those who had never experienced an *exacerbation* and those who had at least 1 *exacerbation* was significant only in the mild asthma group (*P*=0.0479). Mean FEV_1_ values were similar regardless of exacerbation status if asthma severity was moderate or severe.

# References

1. Whalen KA, Legault H, Hang C, Hill A, Kasaian M, Donaldson D, Bensch GW, Bensch G, Baker J, Reddy PS, et al: **In vitro allergen challenge of peripheral blood induces differential gene expression in mononuclear cells of asthmatic patients: inhibition of cytosolic phospholipase A2alpha overcomes the asthma-associated response.** *Clin Exp Allergy* 2008, **38:**1590-1605.

2. Shi L, Reid LH, Jones WD, Shippy R, Warrington JA, Baker SC, Collins PJ, de Longueville F, Kawasaki ES, Lee KY, et al: **The MicroArray Quality Control (MAQC) project shows inter- and intraplatform reproducibility of gene expression measurements.** *Nat Biotechnol* 2006, **24:**1151-1161.

3. Thorell K, Bergman A, Caren H, Nilsson S, Kogner P, Martinsson T, Abel F: **Verification of genes differentially expressed in neuroblastoma tumours: a study of potential tumour suppressor genes.** *BMC Med Genomics* 2009, **2:**53.

4. Wang Y, Barbacioru C, Hyland F, Xiao W, Hunkapiller KL, Blake J, Chan F, Gonzalez C, Zhang L, Samaha RR: **Large scale real-time PCR validation on gene expression measurements from two commercial long-oligonucleotide microarrays.** *BMC Genomics* 2006, **7:**59.

5. Hartigan JA, Wong MA: **A K-means clustering algorithm.** *Applied Statistics* 1979, **28:**100-108.

6. Kluger Y, Basri R, Chang JT, Gerstein M: **Spectral biclustering of microarray data: coclustering genes and conditions.** *Genome Res* 2003, **13:**703-716.

7. Rousseeuw PJ: **Silhouettes: A graphical aid to the interpretation and validation of cluster analysis.** *J Comput Appl Math* 1987, **20:**53-65.

8. McShane LM, Radmacher MD, Freidlin B, Yu R, Li MC, Simon R: **Methods for assessing reproducibility of clustering patterns observed in analyses of microarray data.** *Bioinformatics* 2002, **18:**1462-1469.
